# Supplementary material for: A neutralizing epitope on the SD1 domain of SARS-CoV-2 spike targeted following infection and vaccination
Source: Cell Rep. 2022 Aug 11;40(8):111276. doi: 10.1016/j.celrep.2022.111276 (PMC9365860; doi:10.1016/j.celrep.2022.111276)
Supplement: Document S1. Figures S1–S8 and Tables S1 and S2 [file mmc1.pdf]

Cell Reports, Volume *40*

Supplemental information

A neutralizing epitope on the SD1 domain of SARS-CoV-2 spike targeted following infection and vaccination

Jeffrey Seow, Hataf Khan, Annachiara Rosa, Valeria Calvaresi, Carl Graham, Suzanne Pickering, Valerie E. Pye, Nora B. Cronin, Isabella Huettner, Michael H. Malim, Argyris Politis, Peter Cherepanov, and Katie J. Doores

A neutralizing epitope on the SD1 domain of SARS-CoV-2 Spike targeted following infection and vaccination.

Jeffrey Seow[1]*, Hataf Khan[1]*, Annachiara Rosa[2]*, Valeria Calvaresi[3]*, Carl Graham[1], Suzanne Pickering[1], Valerie E. Pye[2], Nora B. Cronin[4], Isabella Huettner[1], Michael H. Malim[1], Argyris Politis[3]#, Peter Cherepanov[2,5]#, Katie J. Doores[1,6]#

[1] Department of Infectious Diseases, School of Immunology & Microbial Sciences, King's College London, London, UK.
[2]Chromatin Structure and Mobile DNA Laboratory, The Francis Crick Institute, London, UK
[3] Department of Chemistry, King's College London, London, UK.
[4]LonCEM Facility, The Francis Crick Institute, London, UK;
[5]Department of Infectious Disease, St-Mary's Campus, Imperial College London, London, UK.
[6]Lead author

***** These authors contributed equally

# To whom correspondence should be addressed (katie.doores@kcl.ac.uk, Peter.Cherepanov@crick.ac.uk, argyris.politis@kcl.ac.uk)

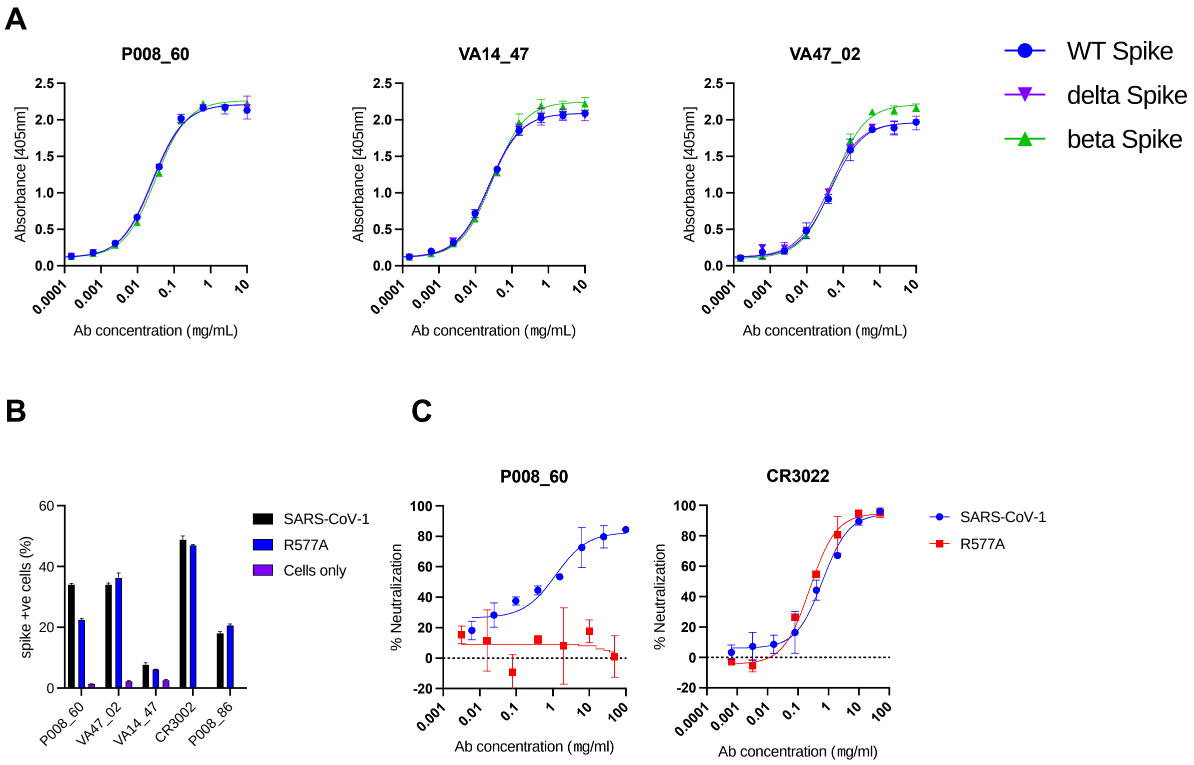

Figure S1. SD1 mAb binding to SARS-CoV-2 variants of concern and SARS-CoV-1. A) Binding of P008_60, VA14_47 and VA47_2 to WT, delta and beta recombinant Spike by ELISA. **B)** SD1 mAbs bind to SARS-CoV-1 Spike and R577A mutant expressed on the surface of HEK 293T cells. CR3022 and P008_86 were used as controls. **C)** Neutralization of SARS-CoV-1 WT and R577A pseudotyped particles by P008_60. CR3022 is used as a control. Experiments were performed in duplicate and performed at least twice. Representative data sets are shown. Error bars represent the range of the values for experiments performed in duplicate (not shown when smaller than symbol size). Related to **Figure 1**.

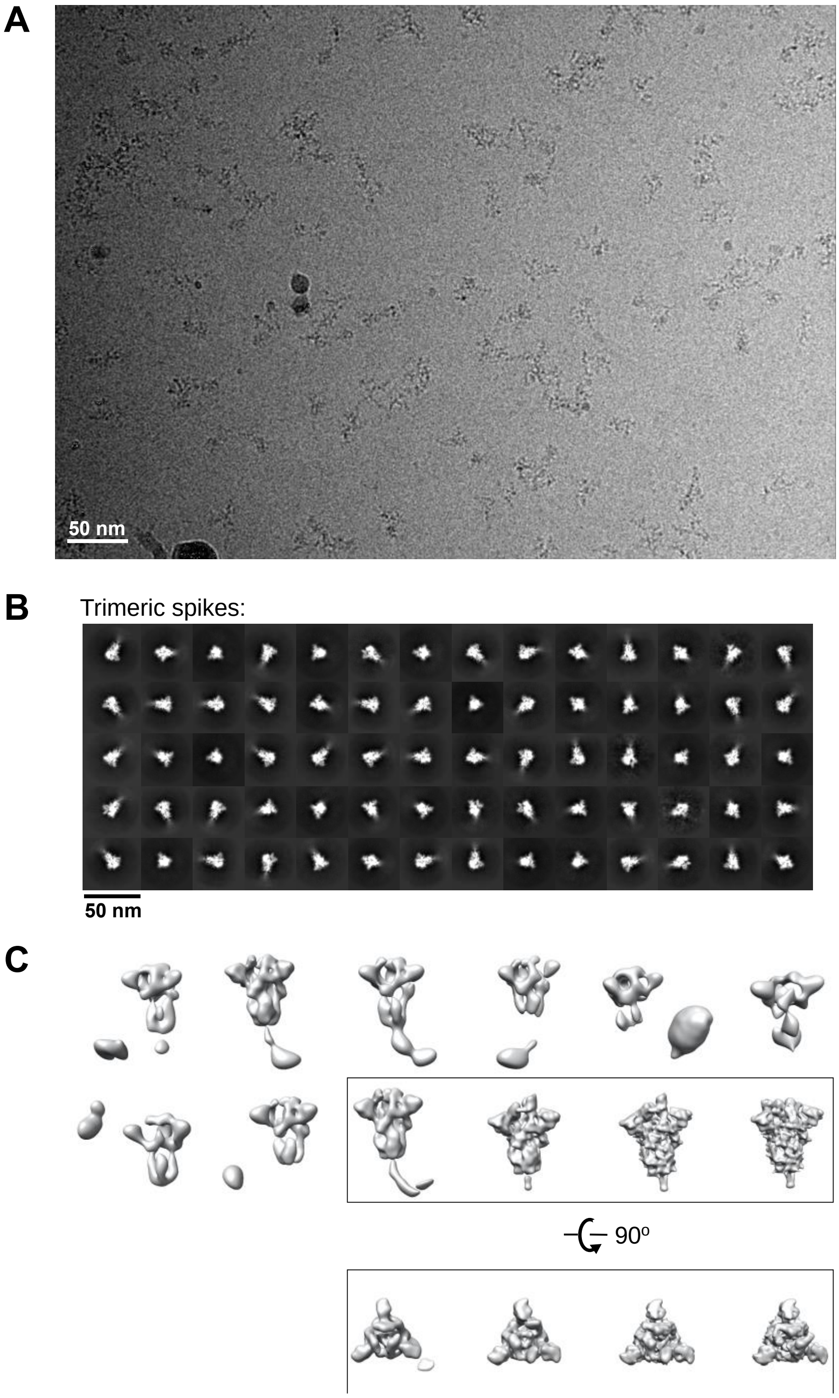

Figure S2. Preliminary cryo-EM data analysis. (A) Example of a micrograph of a vitrified sample containing SARS-CoV-2 spike supplemented with excess Fab P008_60; the scale bar is 50 nm. **(B)** 2D class averages corresponding to trimeric spike ectodomain **(C)** Result of 3D classification of trimeric spikes into 12 classes. The best 4 classes (boxed) are shown in two orthogonal views. Note that features corresponding to bound Fab molecules are absent in 2D and 3D class averages. Related to **Figure 2**.

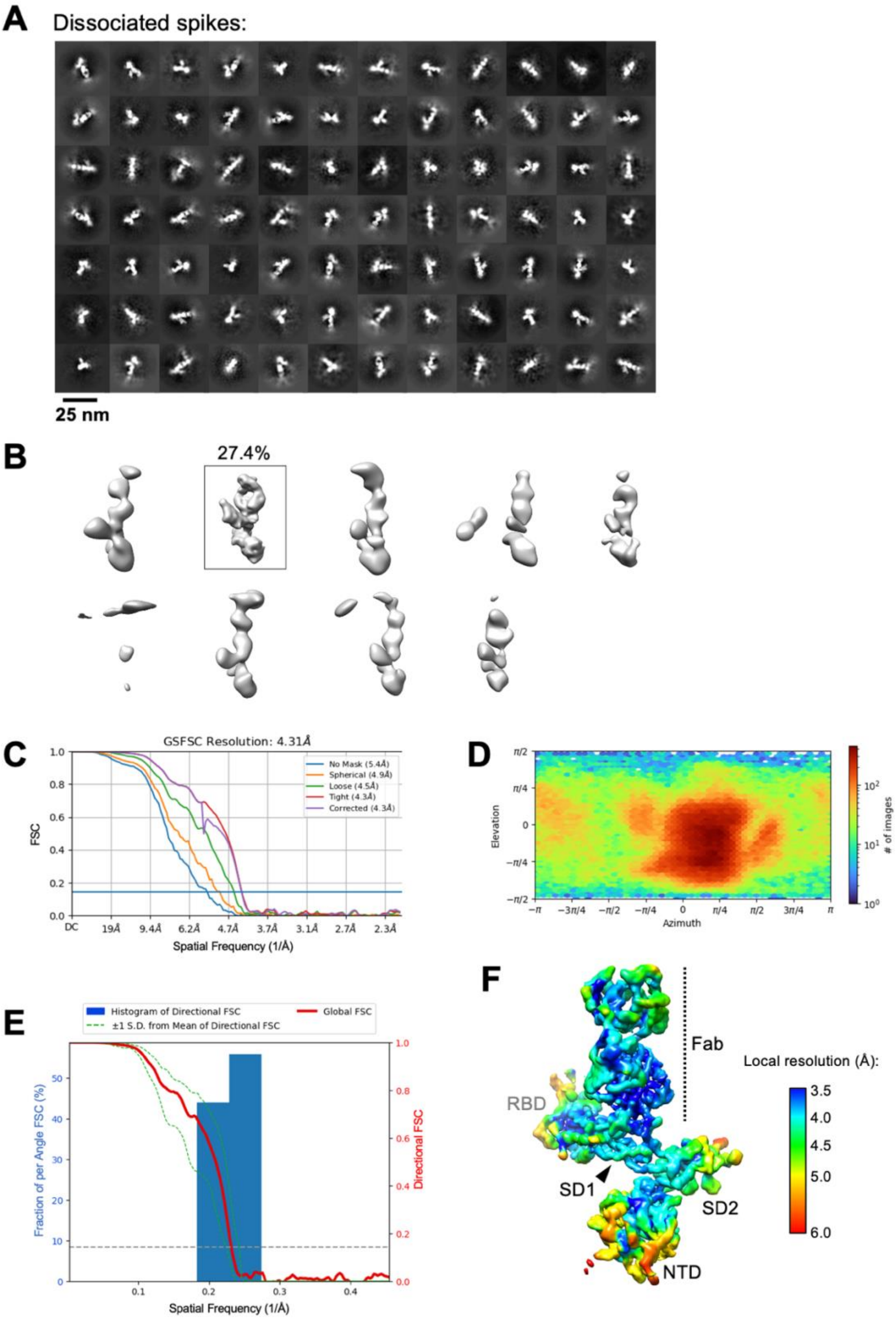

Figure S3. Cryo-EM reconstruction of the S1-Fab P008_60 complex. (A) 2D class averages corresponding to dissociated spikes. **(B)** Results of 3D classification into 9 classes. Particles contributing to the single well-defined 3D class (boxed, 27.4% particles) were retained for downstream processing. **(C, D)** Half-map Fourier shell correlations (FSCs, C) and distribution of the refined particle orientations for the final reconstruction, as implemented in cryoSPARC (D). **(E)** Directional resolution metrics generated by 3DFSC software (12) showing the global FSC curve (thick red line), boundaries of the directional FSCs (± 1 standard deviation, dotted green

lines), and a histogram of directional FSC values (blue bars). **(E)** The final 3D reconstruction, colored by local resolution; the color scheme corresponds to the key on the right. Related to **Figure 2**.

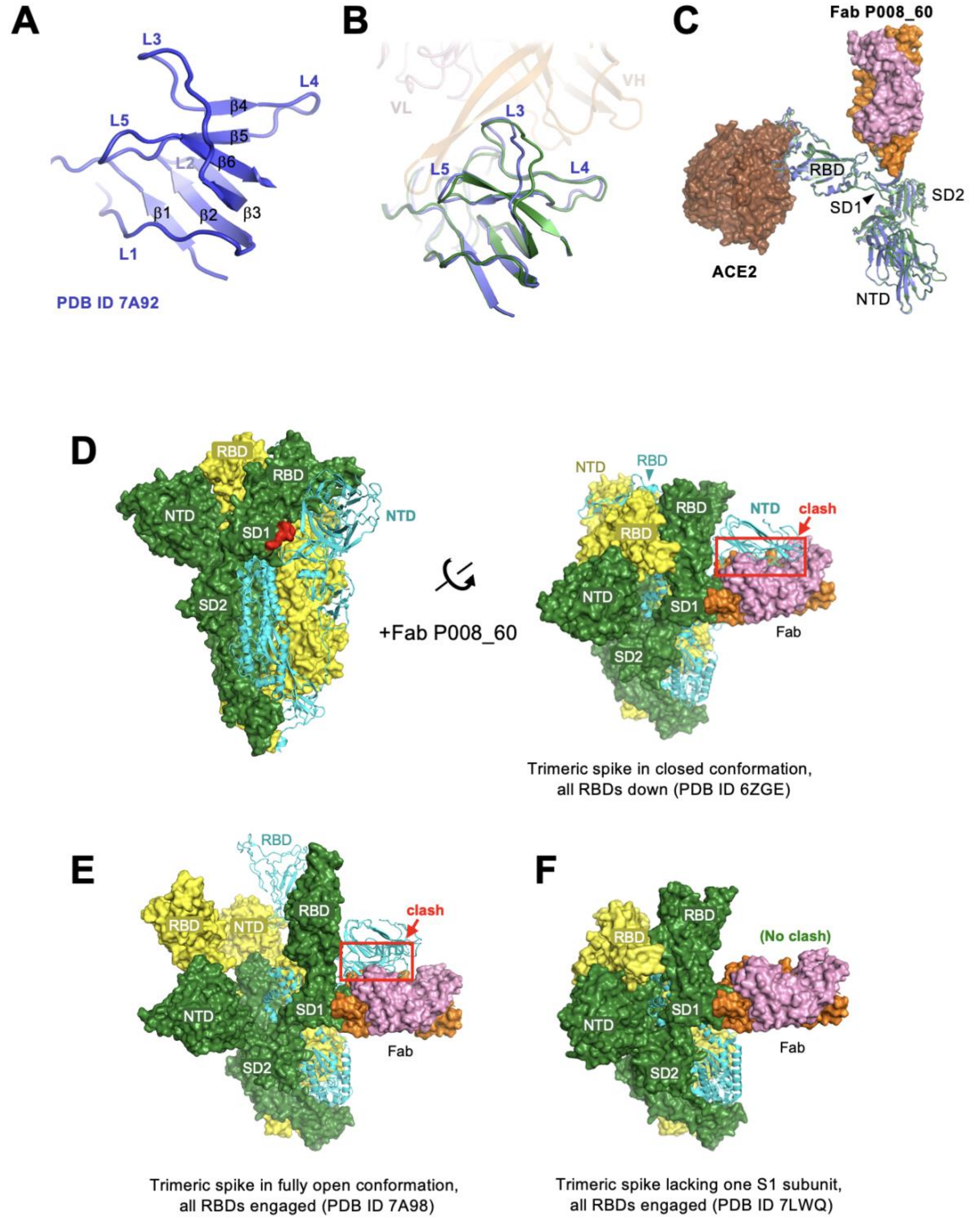

Figure S4. (A) Structure of SD1 shown in cartoons, with individual β-strands and loops indicated; the structure shown is from PDB entry 7A92 (3). **(B)** Superposition of SD1 structures without (blue, PDB entry 7A92 (3)) and with (green) Fab P008_60 bound; the Fab is shown as semi-transparent cartoons. **(C)** Superposition of the ACE2- and P008_60 Fab- bound S1 structures. Fab (pink and orange) and ACE2 (yellow) are shown as surface representations, and S1 as cartoons. **(D)** Left: models of the trimeric spike in fully closed (left, PDB entry 6ZGE (4)) in the orientation and colors as in **Fig. 2C**, with two spike subunits shown as green and yellow surface representations (including S2 portions) and one as cyan cartoons. Right: model of the closed trimeric spike trimer in complex with P008_60, bound to the SD1 of the green spike subunit. Note the extensive clashes (indicated with red box and arrowhead) with the NTD of the neighboring (cyan) S1 subunit. **(E,F)** Models of

fully open trimeric spike **(E)** (PDB entry 7A98 (3)) and partially dissociated trimeric spike, lacking one of the S1 subunits **(F)** (PDB entry 7LWQ (21)) in complex with P008_60. For clarity, ACE2 molecules (present in PDB entry 7A98) are hidden; Fab and two spike subunits are shown as surface representation and one spike chain is shown as cartoons. Related to **Figure 2**.

A

	VH	%	IGHJ	%	IGHD	CDRH3	VK	%	IGKJ	%	CDRL3
**P008_60**	IGHV3-30	95.5	IGHJ4	97.9	IGHD1-14	CARDTPDLETYYFDCW	IGKV3-20	97.9	IGKJ2	87.2	CQQYGDSPRGSF
**VA14_47**	IGHV3-30	97.9	IGHJ6	90.3	IGHD3-3	CTKADYYDFWSGYQKTYYYYMDVW	IGKV3-20	97.9	IGKJ5	100.0	CQQYGSSPQITF
**VA47_2**	IGHV4-61	94.2	IGHJ3	94.0	IGHD3-22	CARVDPYYYSSSGYWTNAFDIW	IGKV3-20	95.0	IGKJ1	94.7	CHQYDNLWTF

B

Heavy chain alignment

```
47_2          QVQLQESGPGLVKPSQTLSLTCTVSGGSISNTNYFWNWIRQPAGKGLEWIGHTYTSGS-T 59
14_47         EVQLVESGGGVVQPGRSLRLSCGGSGFTFS--SHAMHWVRQAPGKGLEWVAVISYDGSYQ 58
8_60          EVQLVESGGGVVQPGRSLRLTCAASGFIFS--SYGMHWVRQAPGKGLEWVAVISYDGSYK 58
              :*** *** *:*:*.::* *:*  **  :*  .:  :*:**  ******:.    .**

47_2          NYNPSLKSRVTISIDTSRNQFSLKLSSVTATDTAVYYCARVDPYYYSS--SGYWTNAFDI 117
14_47         YYADSVKGRFTISRDNSKNALYLQMNSLRVEDTAIYYCARDT--------PDLETYYFDC 110
8_60          YYADSVKGRFTISRDNSKNTLYLQMNSLRAEDTAVYYCTKADYYDFWSGYQKTYYYYMDV 118
               *  *:*.*.*** *.*:* : *::.*: . ***:***::                 :*

47_2          WGQGTMVTVSS  128
14_47         WGQGTLVTVSS  121
8_60          WGKGTTVTISS  129
              **:** **:**
```

Light chain alignment

```
14_47         EIVMTQSPGTLSLSPGERATLSCRASQSVSSSYLAWYQQKPGQAPRLLIYSACSRATGIP 60
8_60          DIQLTQSPGTLSLSPGERATLSCRASQSVSSSYLAWYQQKPGQAPRLLIYGTSSRATGIP 60
47_02         EIVMTQSPGTLXSSPGERVTLSCRASQSVSSNYLAWYQQKPGQAPRLLIYGASKRATGIP 60
              :* :*******  *****.************.*****************.:..******

14_47         DRFSGSGSGTDFTLTISRLEPEDFAVYYCQQYGDSPRGSFGQGTKVDIK    109
8_60          DRFSGSGSGTDFTLTISRLEPEDFAVYYCQQYGSSPQITFGQGTRLEIK    109
47_02         DRFSGGGSGTDFTLTISRLEPEDFAIYYCHQYDN--LWTFGQGTKLEIK    107
              *****.******************:***:**..    :*****:::**
```

Figure S5: SD1 mAb germline characteristics. A) Germline gene usage and level of somatic hypermutation**. B)** SD1-specific mAb heavy and light chain alignment. Related to **Figure 1** and **Figure 2**.

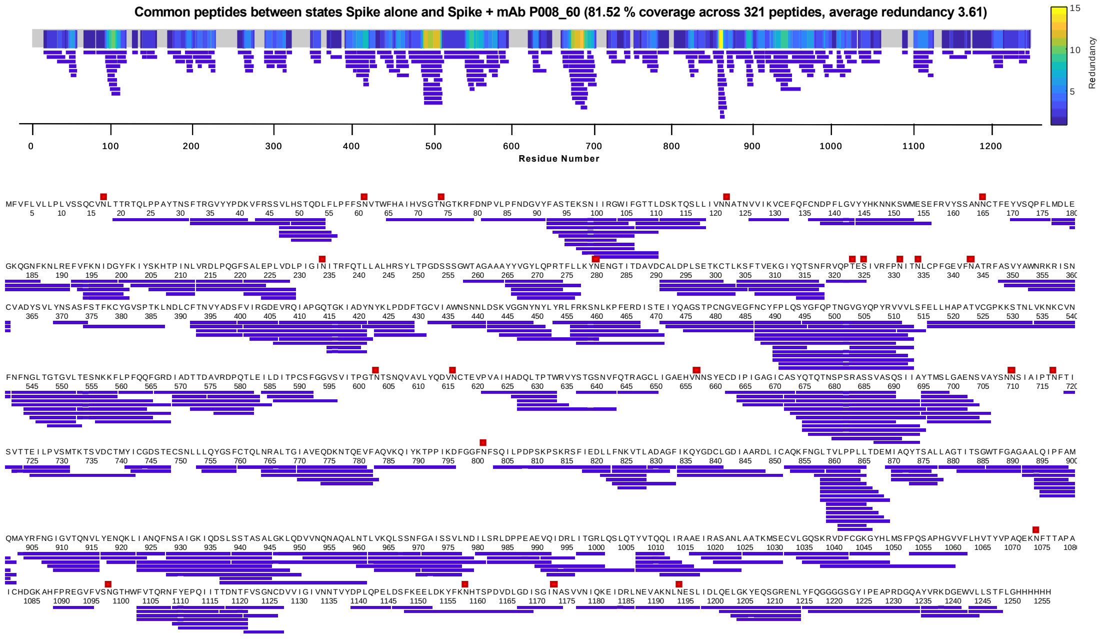

Fig. S6. Sequence coverage of peptides followed by HDX-MS. The blue segments indicate the peptides followed and the red squares indicate the glycosylation sites. Related to **Figure 3**.

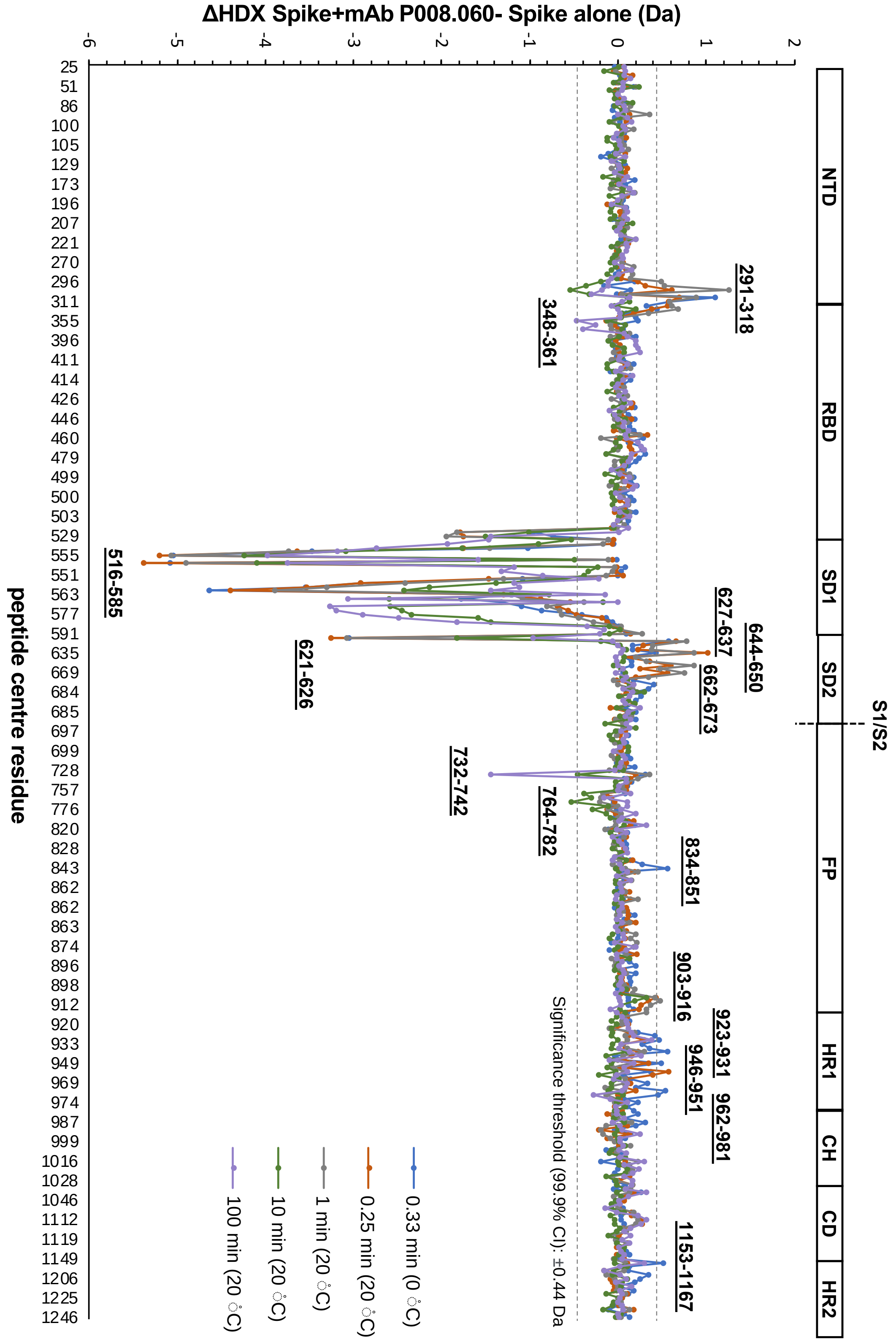

Fig. S7. Difference plot illustrates the differences in HDX over the measured time points between Spike + mAb P008_60 and Spike alone. Residues comprising a region with a statistically significant difference in HDX are indicated. The peptides are arranged according to their position from N- to C-terminus. The various subdomains of the Spike protein are indicated. NTD: N-terminal domain, RBD: receptor binding domain, SD1: subdomain 1, SD2: subdomain 2, FP: fusion peptide, HR1: heptad repeat 1, CH: central helix, CD: connector domain, HR2: heptad repeat 2. A dotted line denotes the furin cleavage site (separating S1 and S2 domains). Related to **Figure 3**.

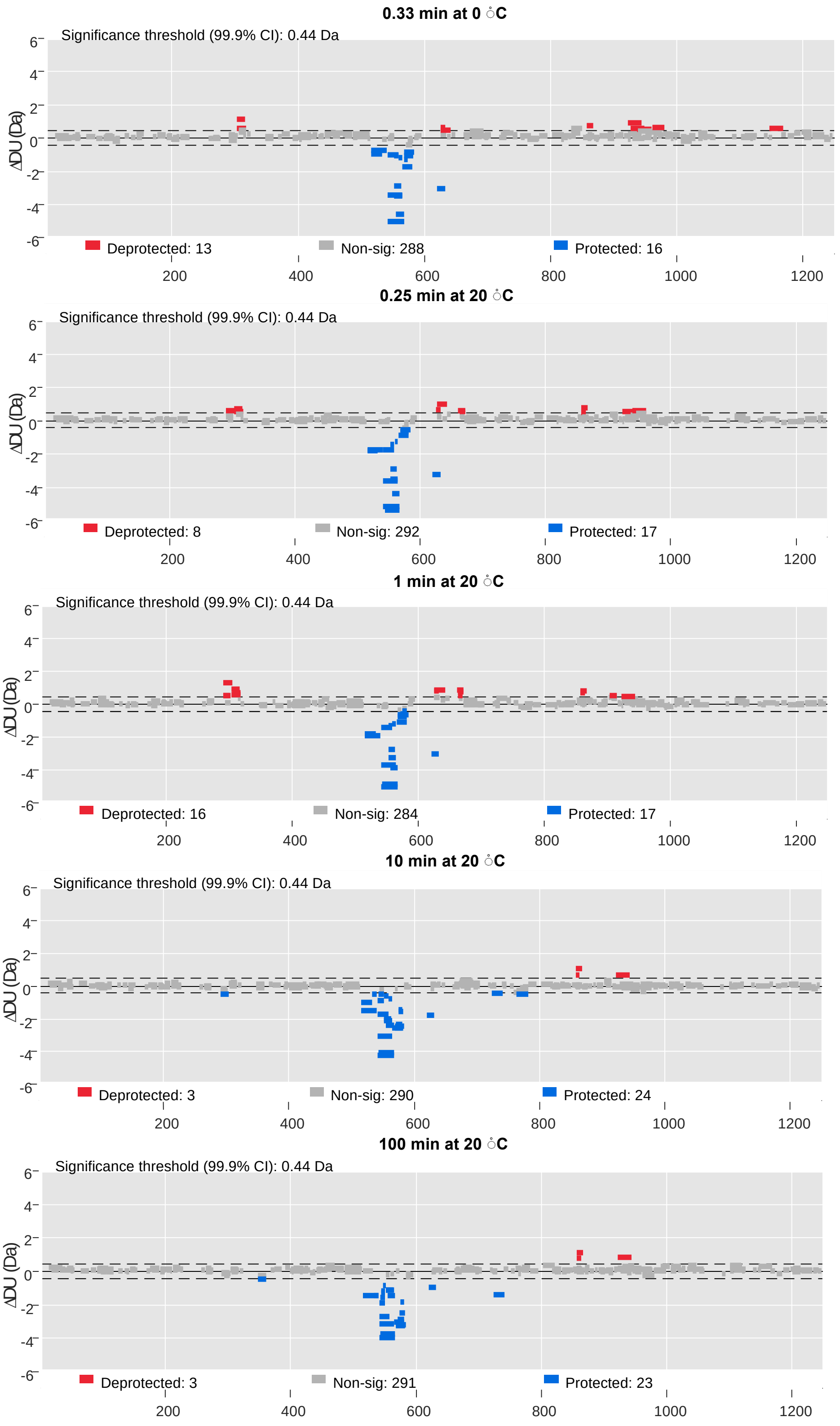

Fig S8. Statistically significant and not significant peptides grouped by time point and arranged according to their position from the N- to the C-terminus. Figures generated by Deuteros software (version 2.0). Related to **Figure 3**.

Table S1. Cryo-EM data collection, 3D volume reconstruction, and model refinement. Related to **Figure 2**.

**Database accession codes**	
EMDB	EMDB-14591
RCSB	7ZBU
**Data collection**	
Microscope	Titan Krios G3i
Operating voltage (kV)	300
Detector	Gatan K3
Physical pixel size (Å)	1.1
Defocus range (μm)	-0.7-3.6
Number of frames per movie	40
Total electron dose (e/Å^2)	50
Total movies acquired/used	16,624/15,980
Movie alignment software	MotionCor2
**3D Reconstruction**	
Software for 2D classification	cryoSPARC-2
Software for 3D classification	Relion-3.1
Software for reconstruction	Relion-3.1
Number of extracted particles	3,772,722
Number of refined particles	166,619
Symmetry imposed	C1
Map resolution (Å) [a]	4.31
Map 3D FSC sphericity	0.885
**Model refinement**	
Software for real-space refinement	Phenix version dev-4213
Number of atoms	8,529
Real-space correlation coefficient	0.68
Mean B-factor (Å^2)	133
R.m.s. deviations	
Bonds (Å)	0.003
Angles (°)	0.679
Validation	
MolProbity score	1.73
Clash score	10.09
Rotamers outliers (%)	0.21
Ramachandran plot quality (%)	
Favored	96.69
Disallowed	0

[a] Based on the FSC of 0.143 between half-sets.

Table S2: HDX summary table. Related to **Figure 3**.

States	Spike protein alone; Spike protein + mAb P008_60
HDX reaction details	PBS buffer (137 mM NaCl, 2.7 mM KCl, 8 mM Na_2HPO_4, and 2 mM KH_2PO_4); pH/D_{read}=7.6; 83.3% D_2O
HDX time course (min)	0.33 at 0 ºC (on ice); 0.25, 1, 10 and 100 at 20 ºC
HDX control samples	Maximally labelled sample
Back-exchange (mean / IQR)	29.12% / 10.55%
# of Peptides	321
Sequence coverage	81.52%
Average peptide length / redundancy	11.55 / 3.61
Replicates	3 (technical)
Repeatability (average SD)	0.0169 (Spike protein alone); 0.0151 (Spike protein + mAb P008_60)
