# Supplementary figures and images for: A neutralizing epitope on the SD1 domain of SARS-CoV-2 spike targeted following infection and vaccination
Source: Cell Rep. 2022 Aug 11;40(8):111276. doi: 10.1016/j.celrep.2022.111276 (PMC9365860; doi:10.1016/j.celrep.2022.111276)

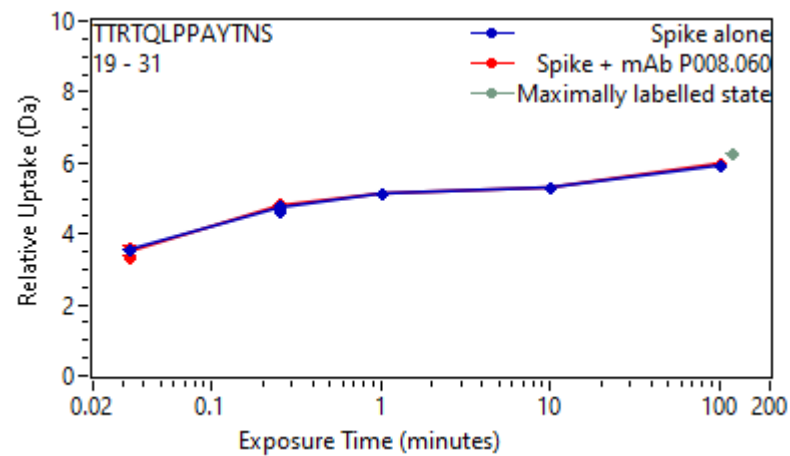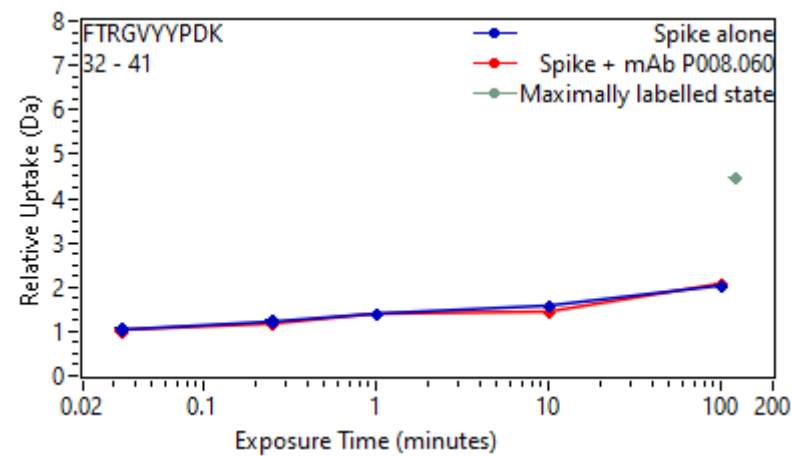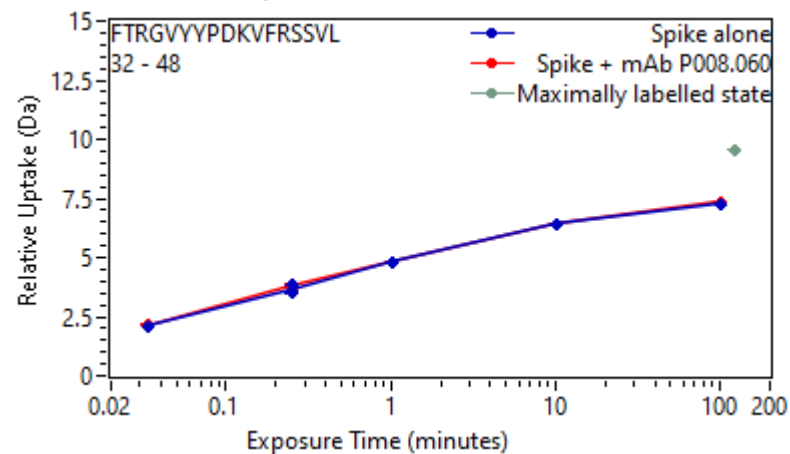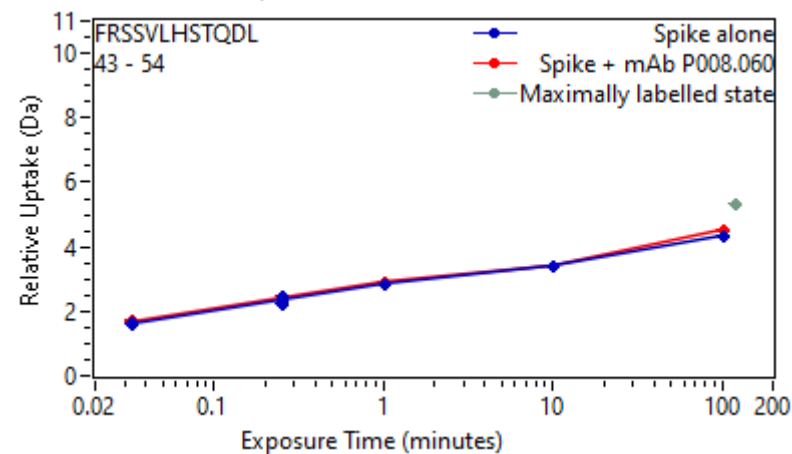

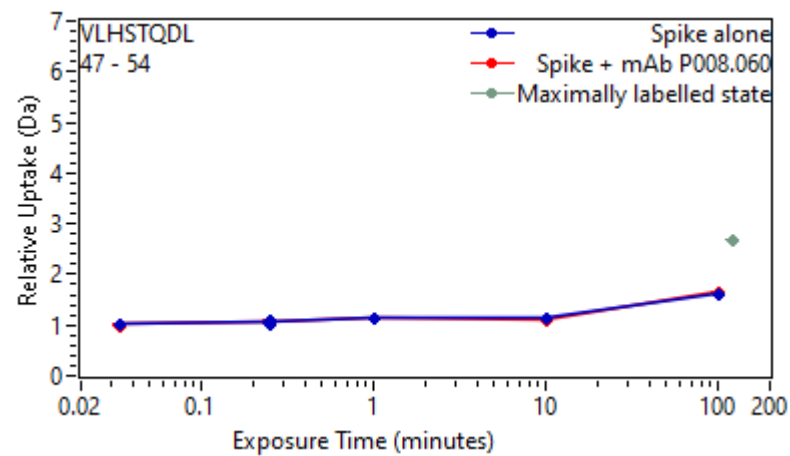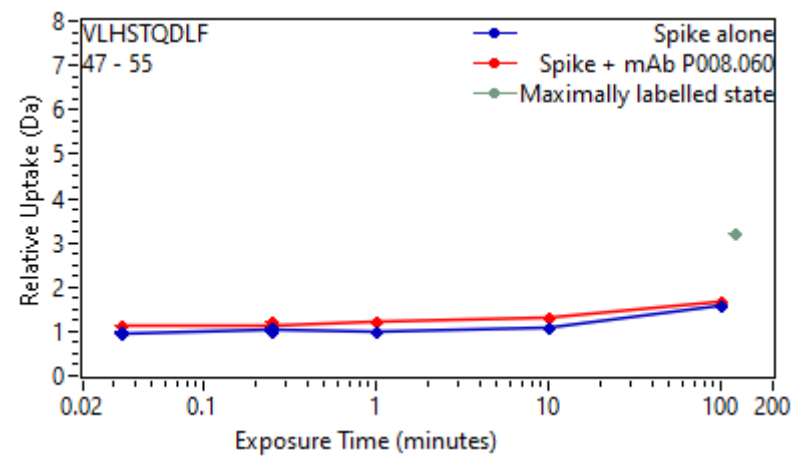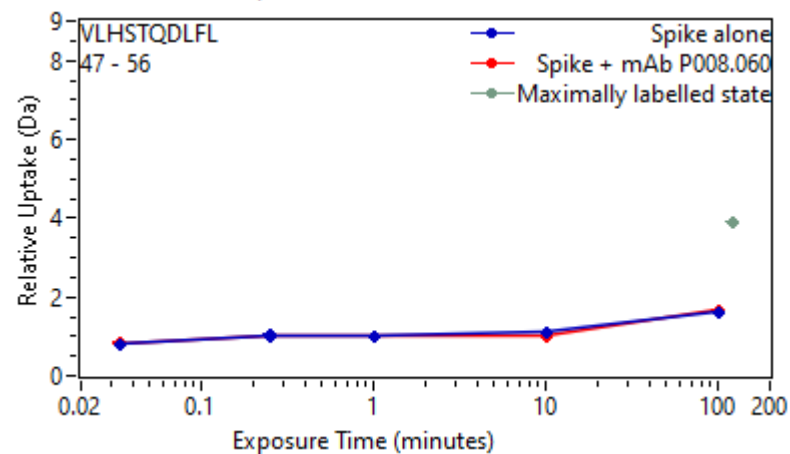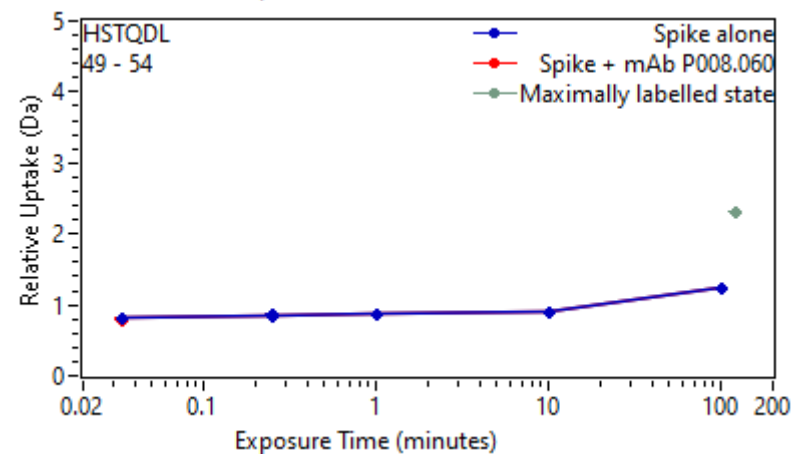

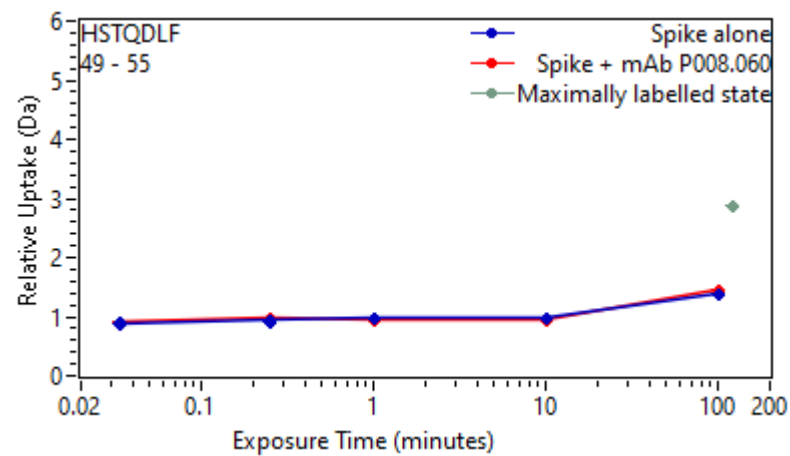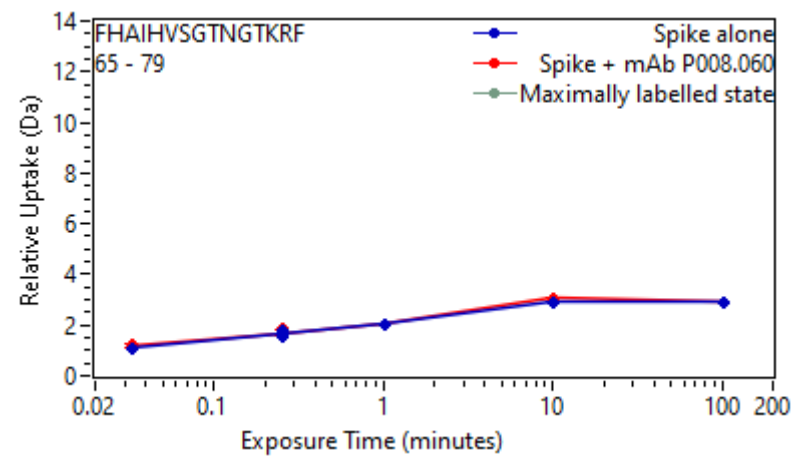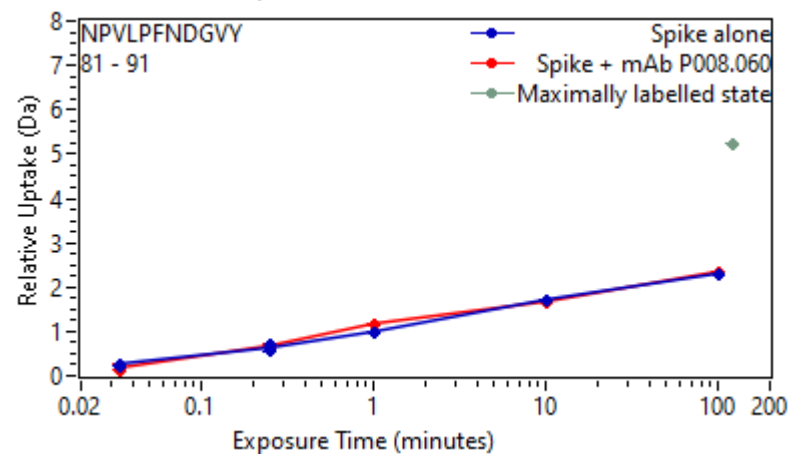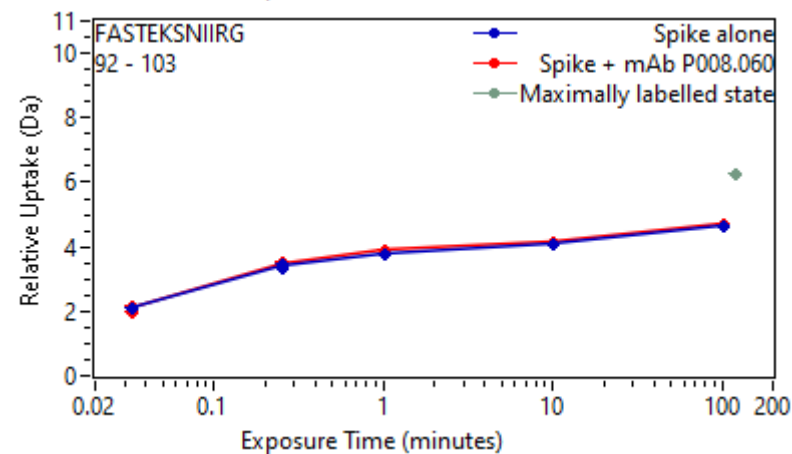

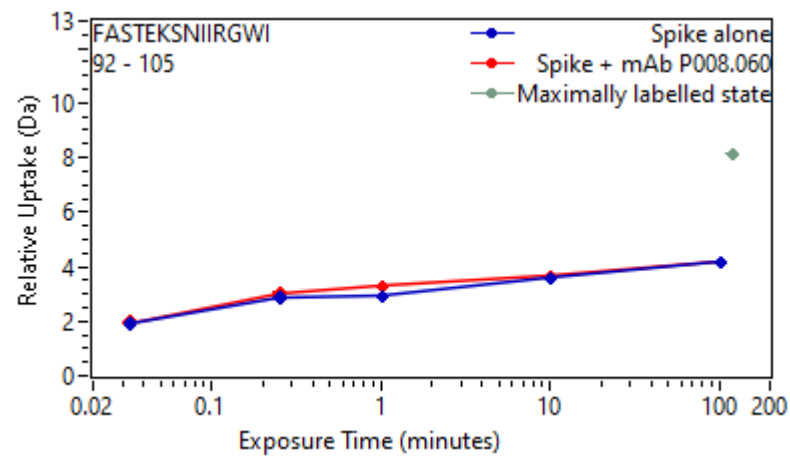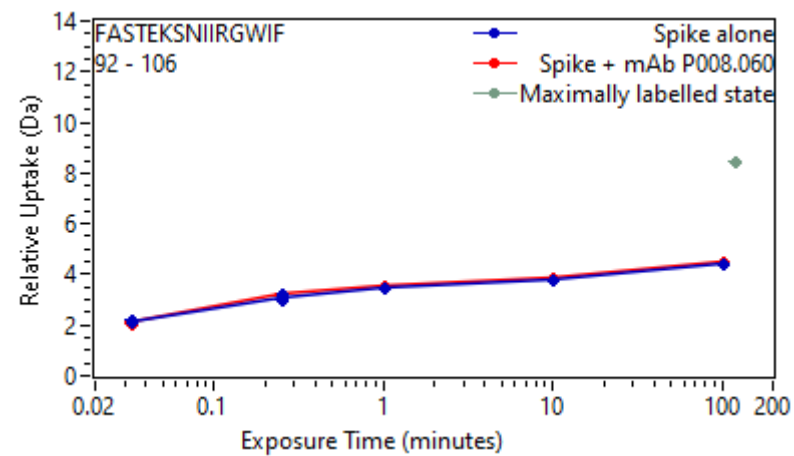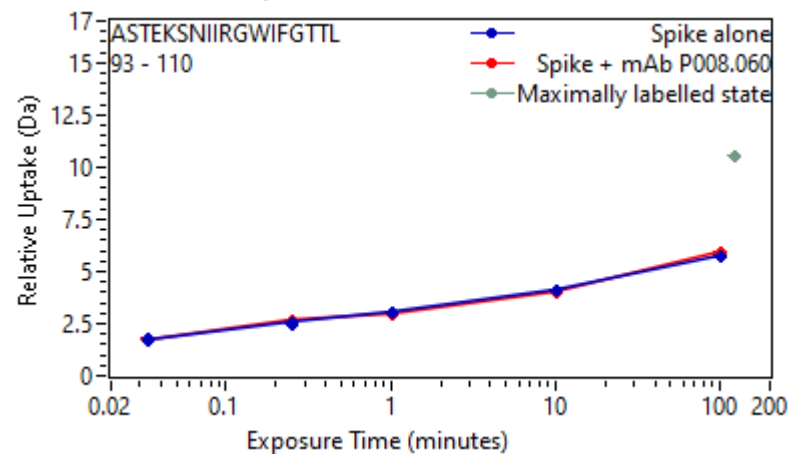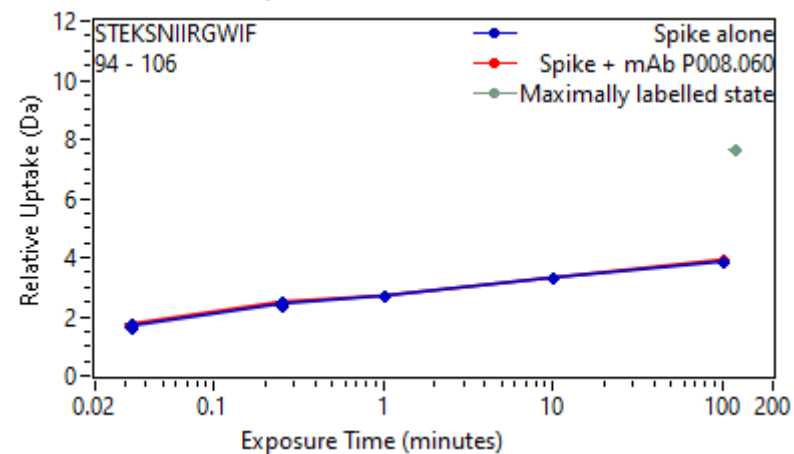

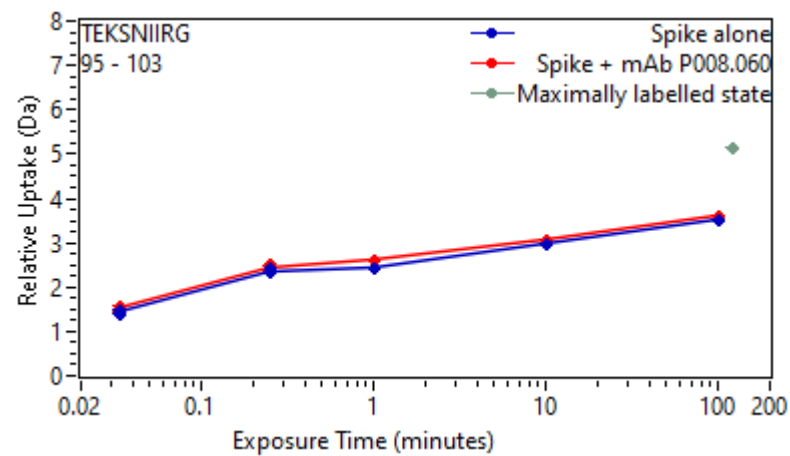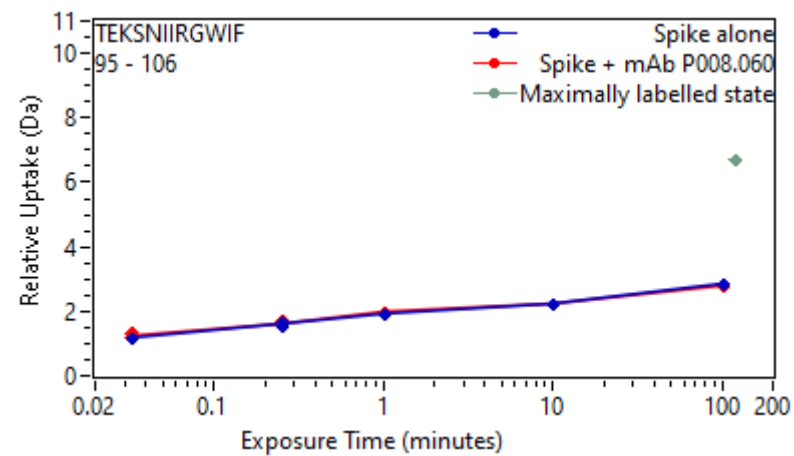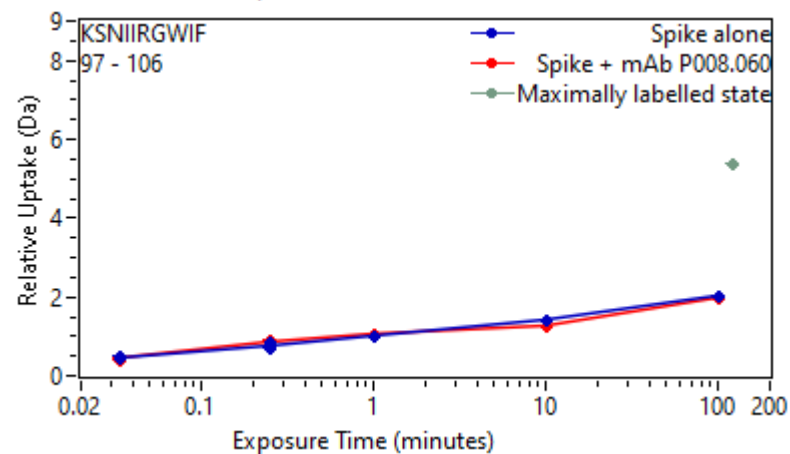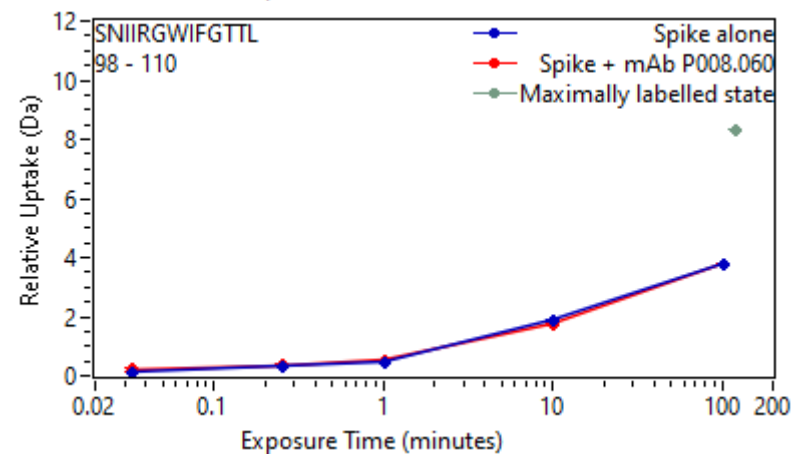

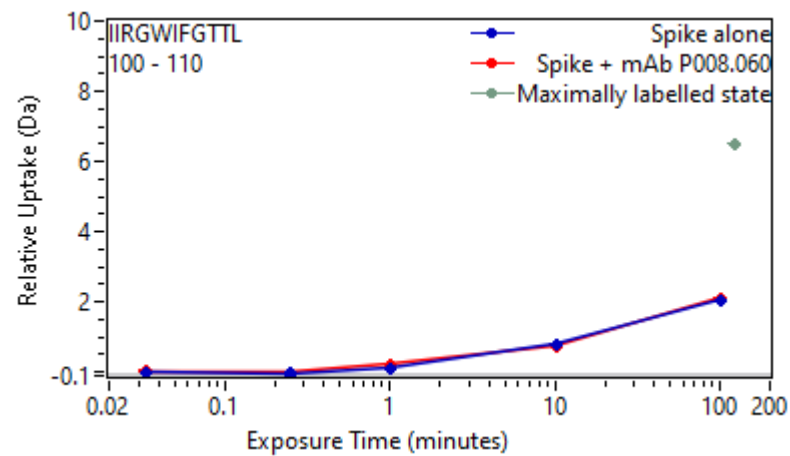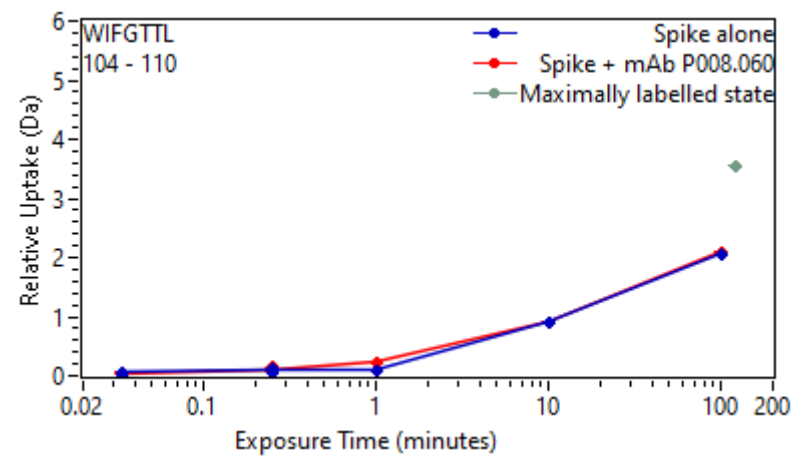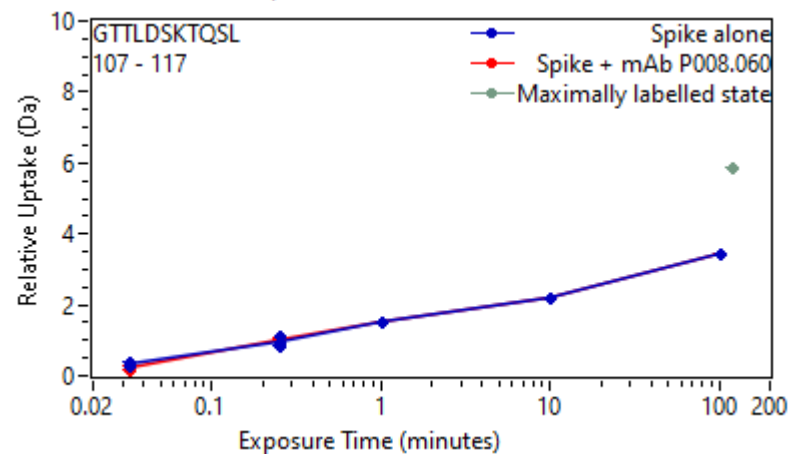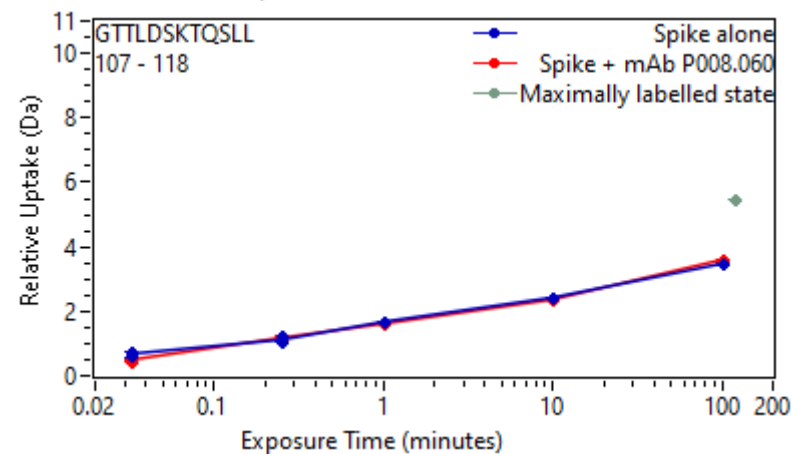

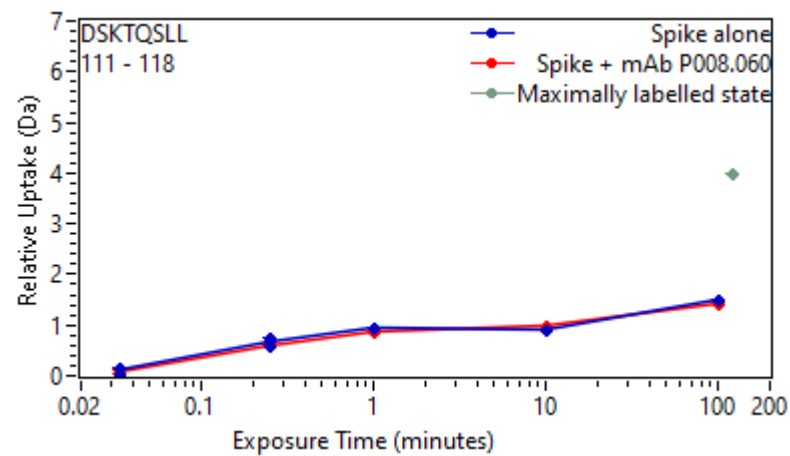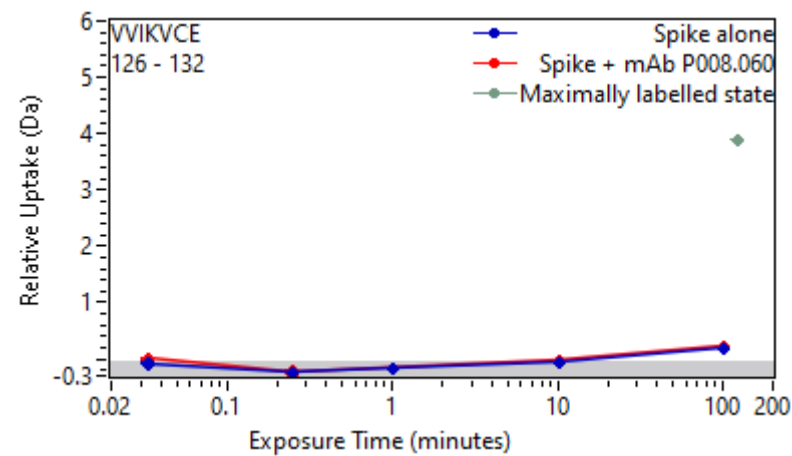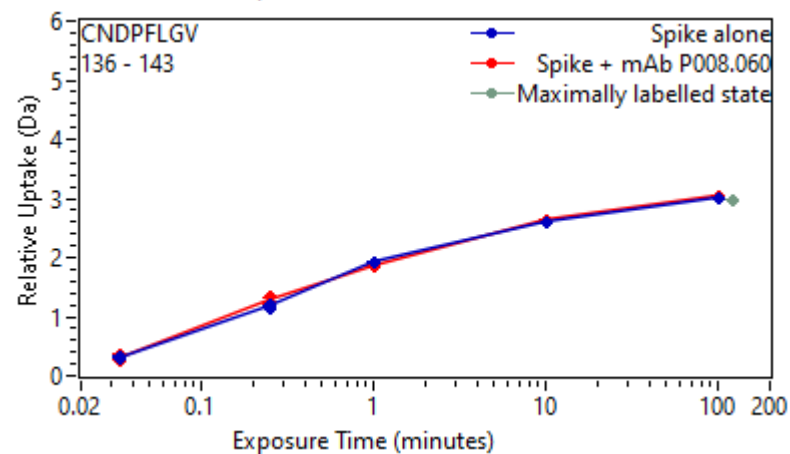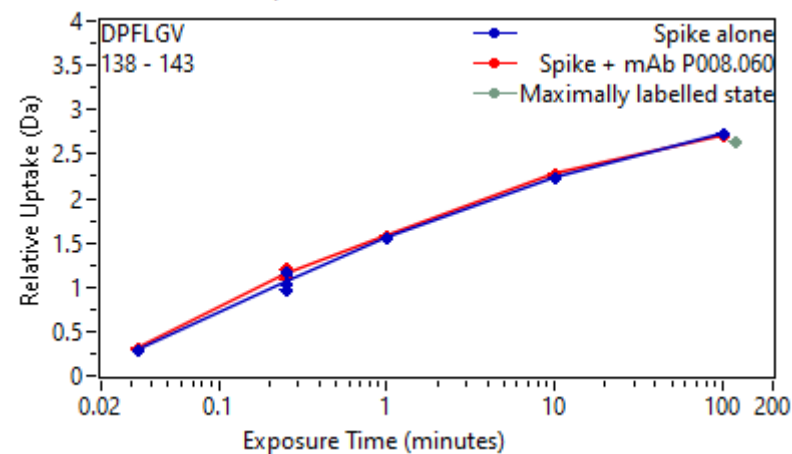

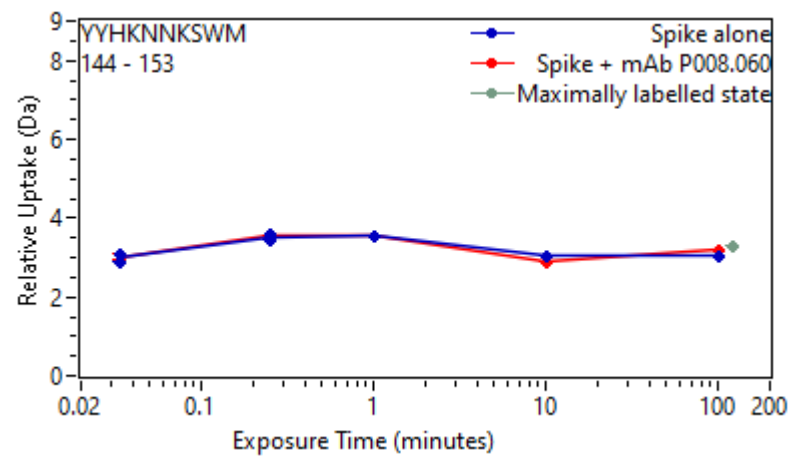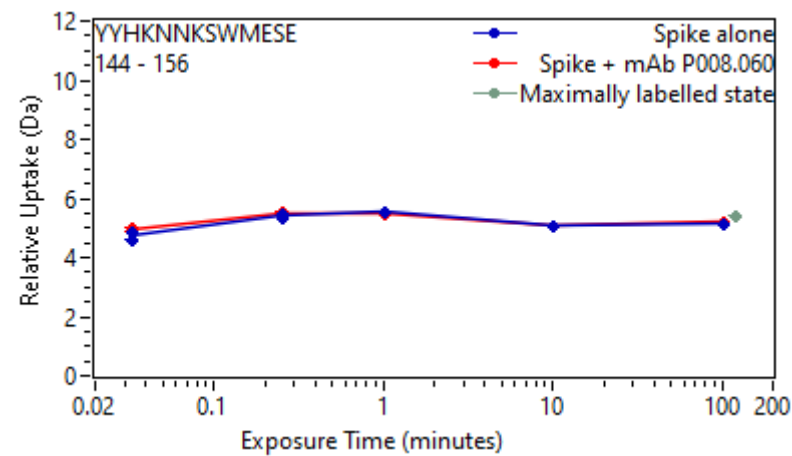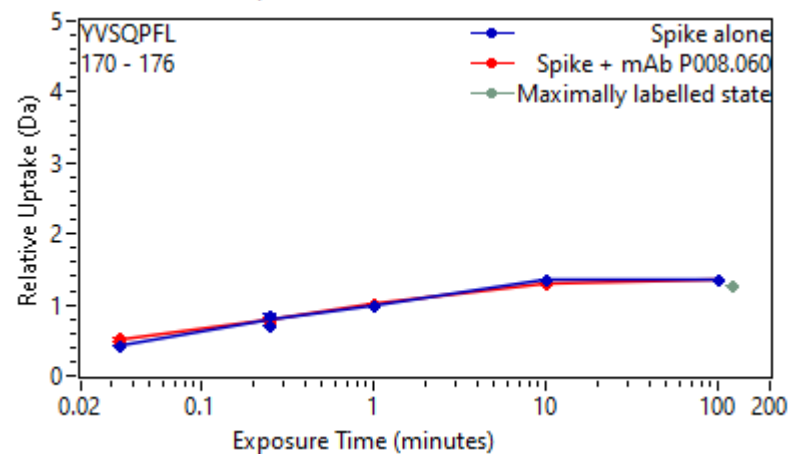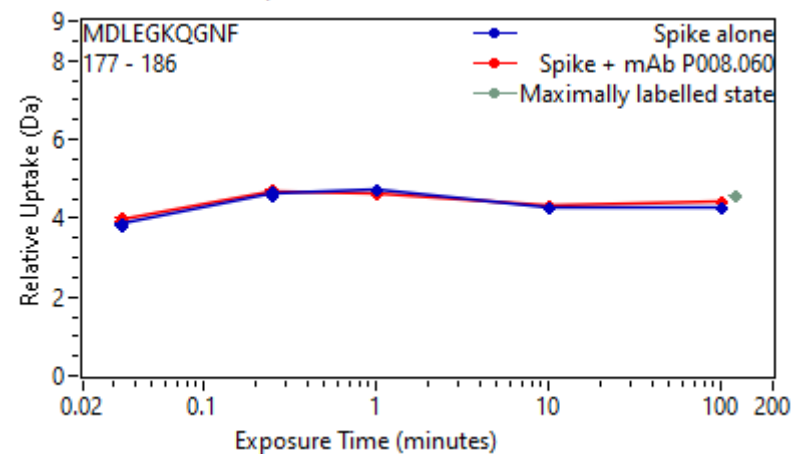

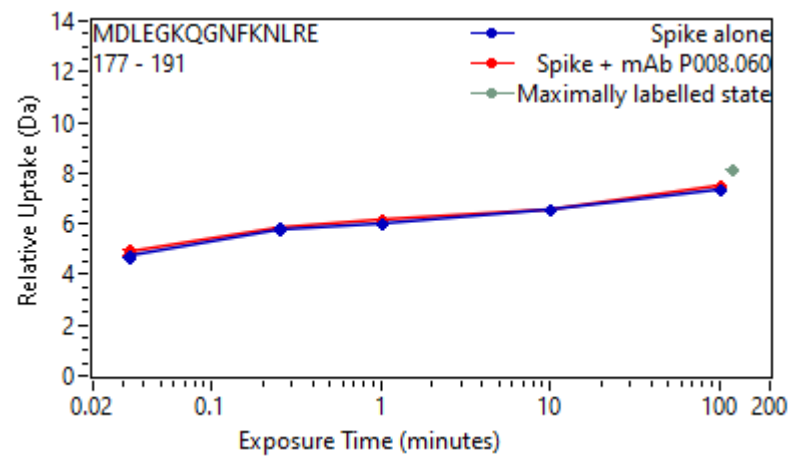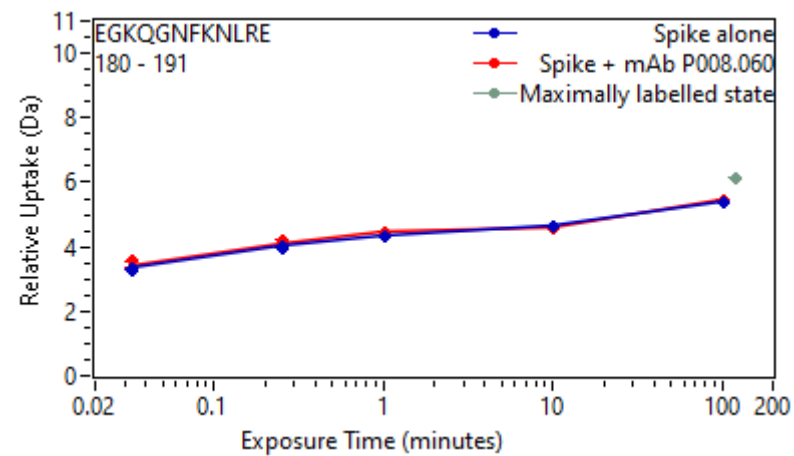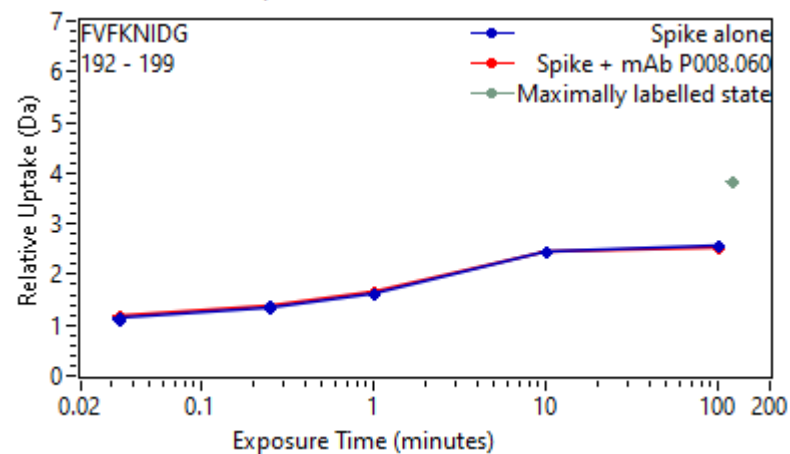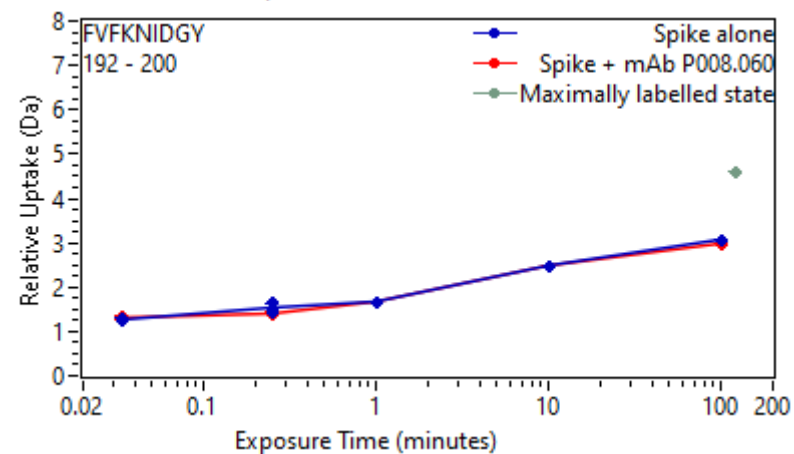

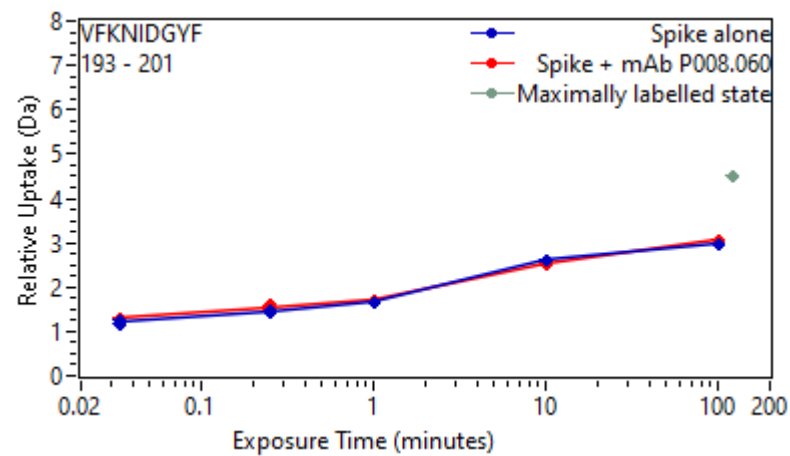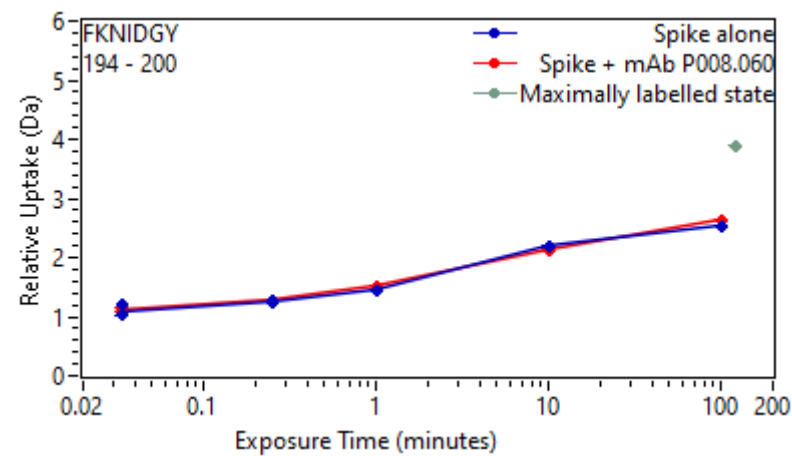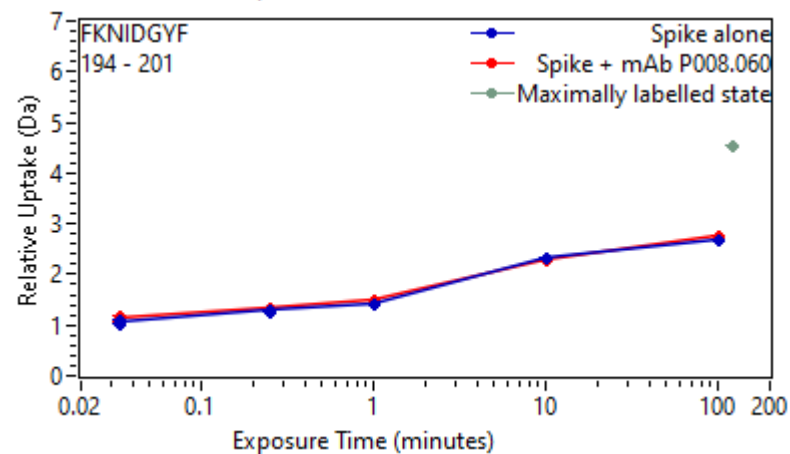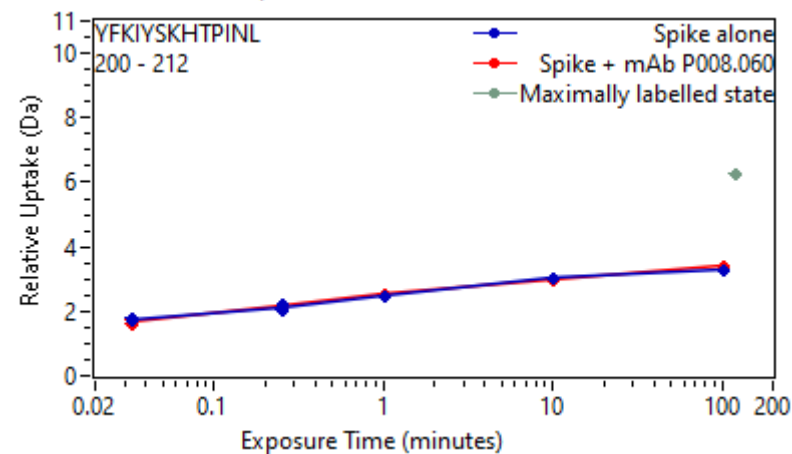

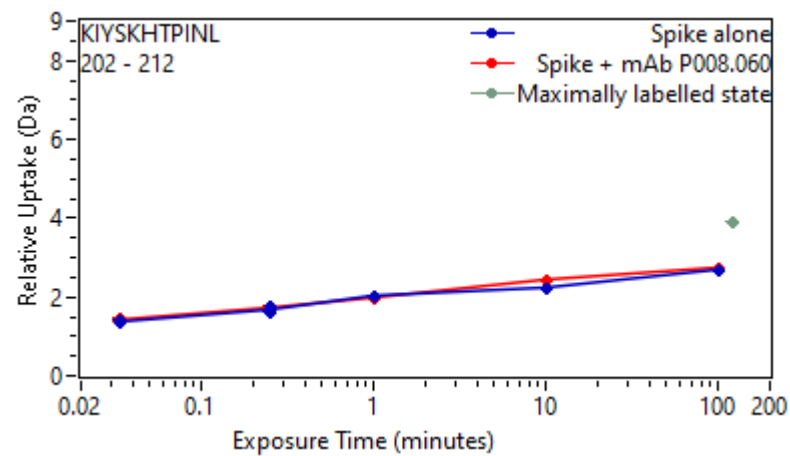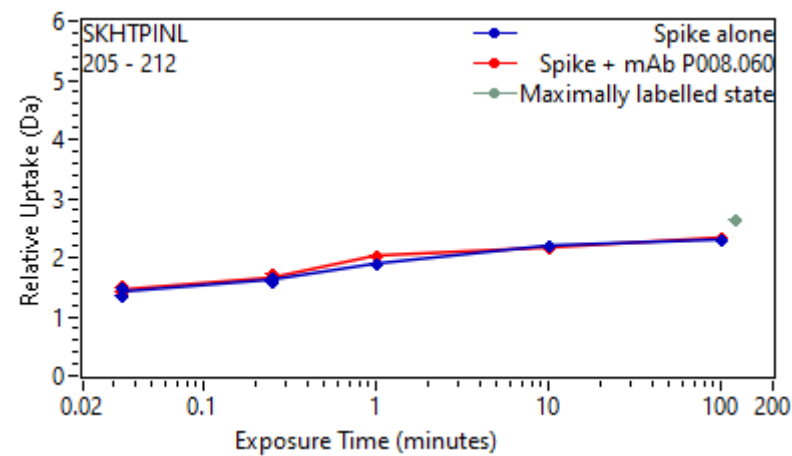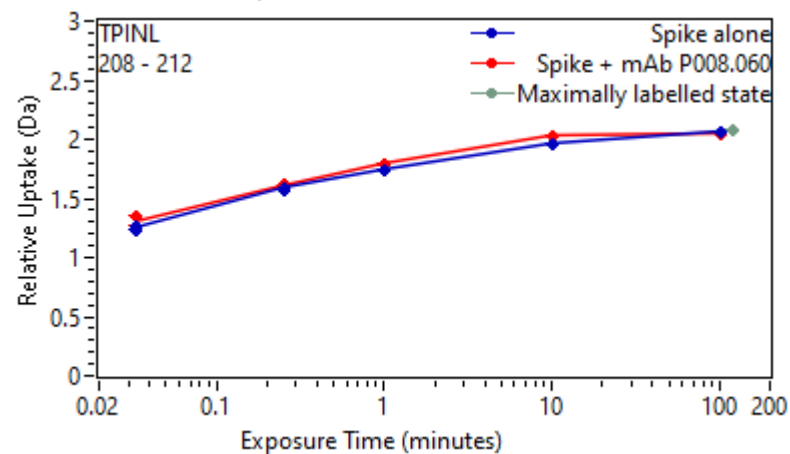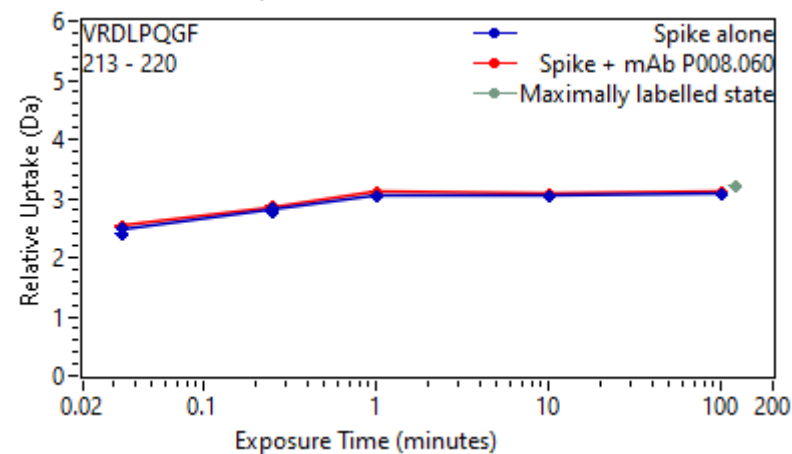

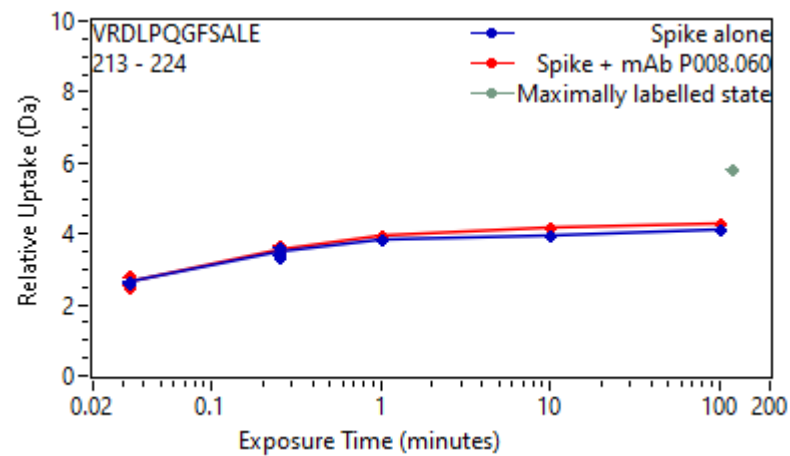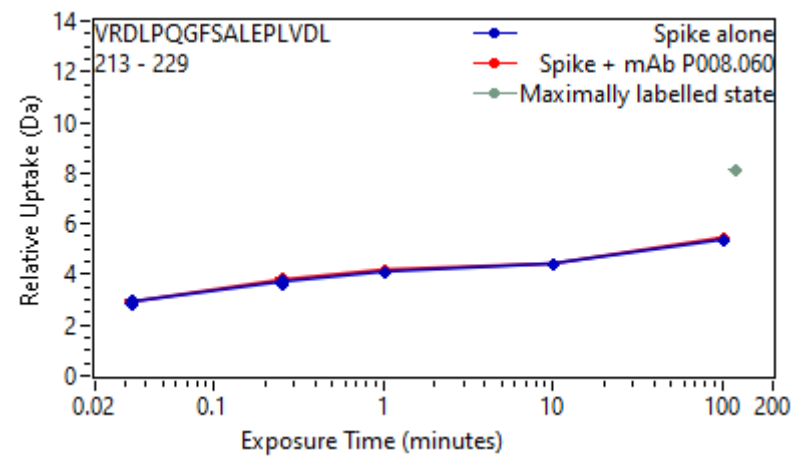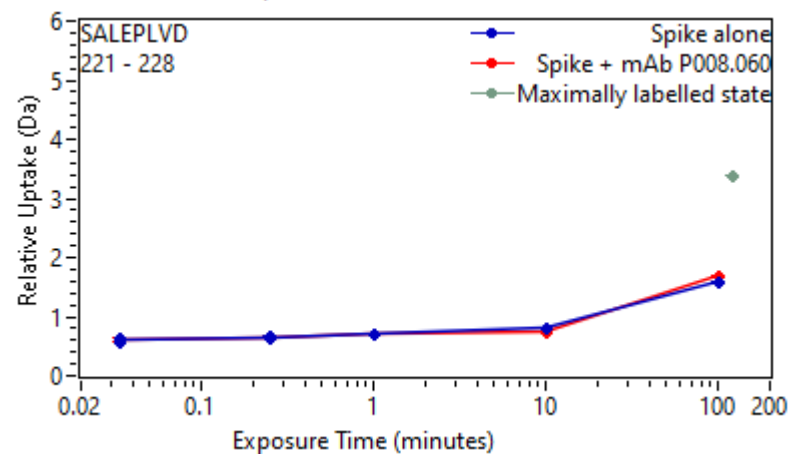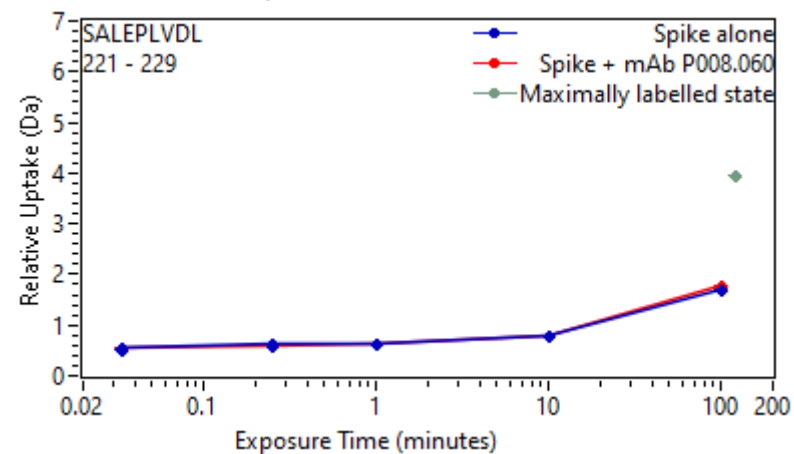

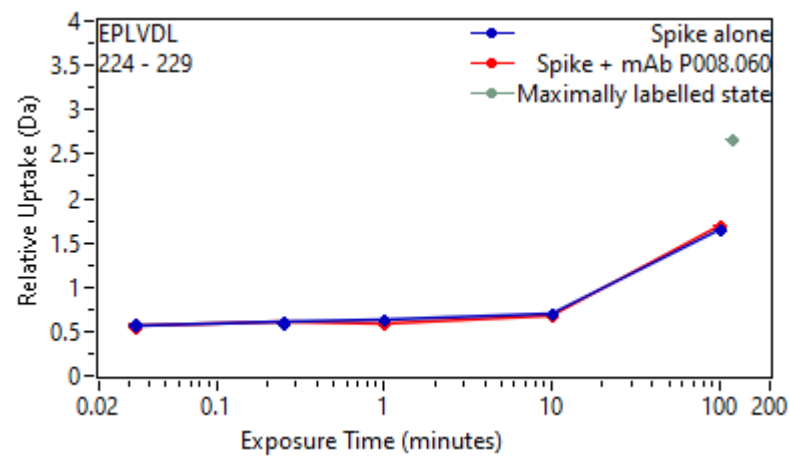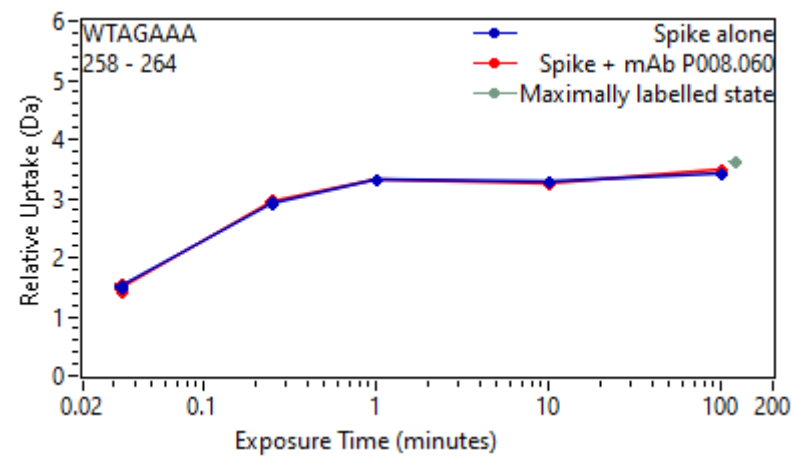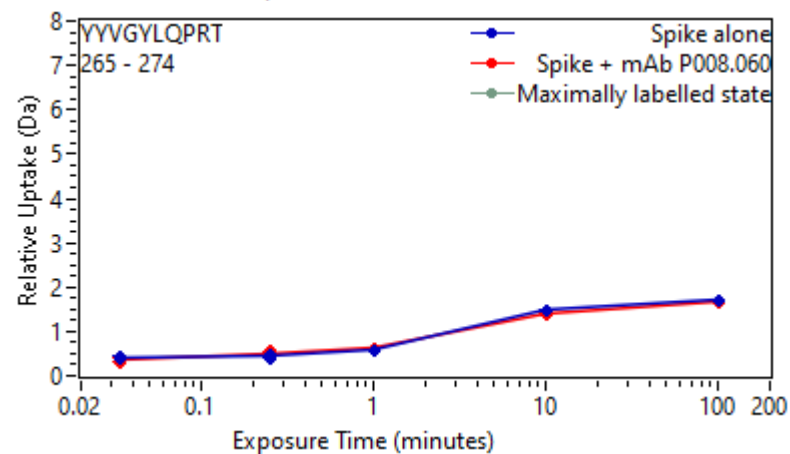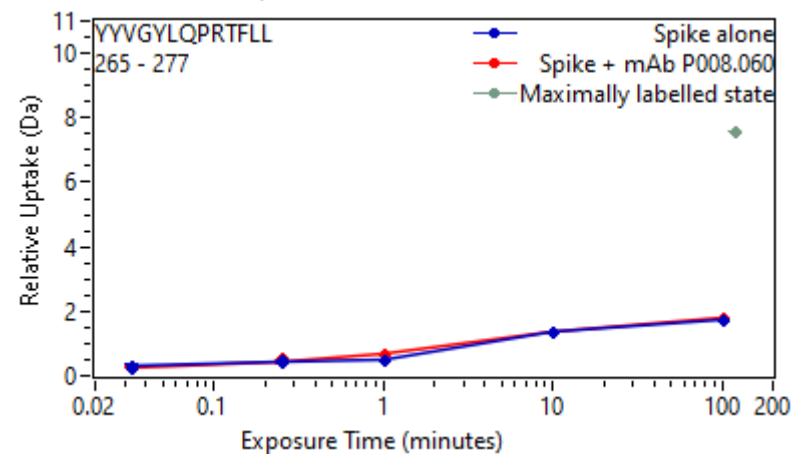

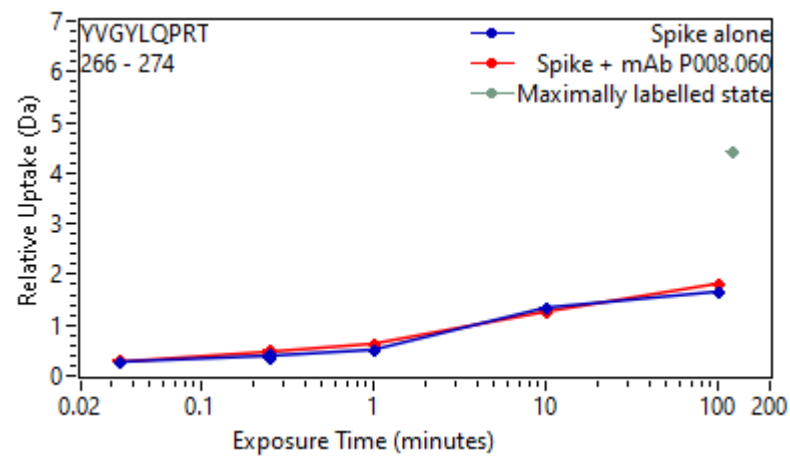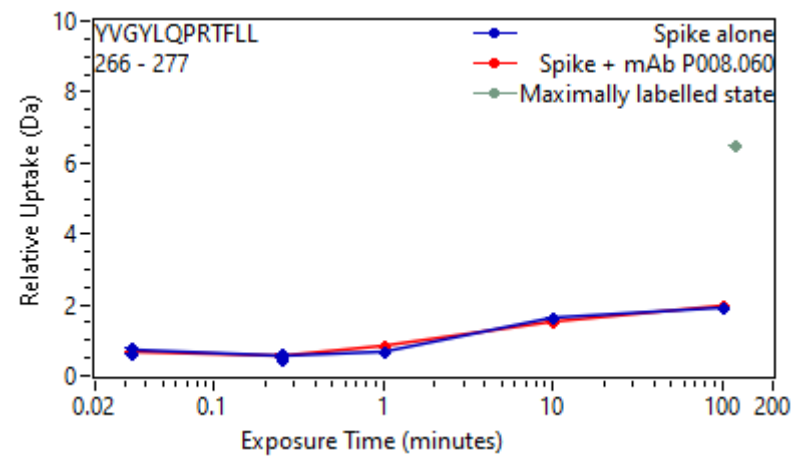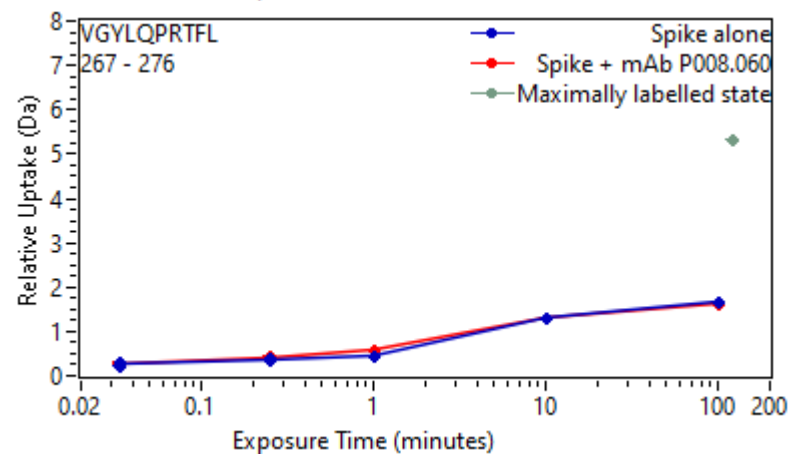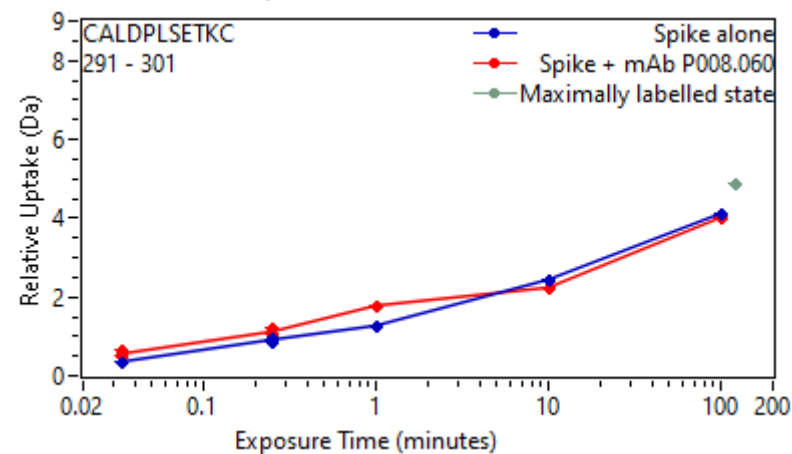

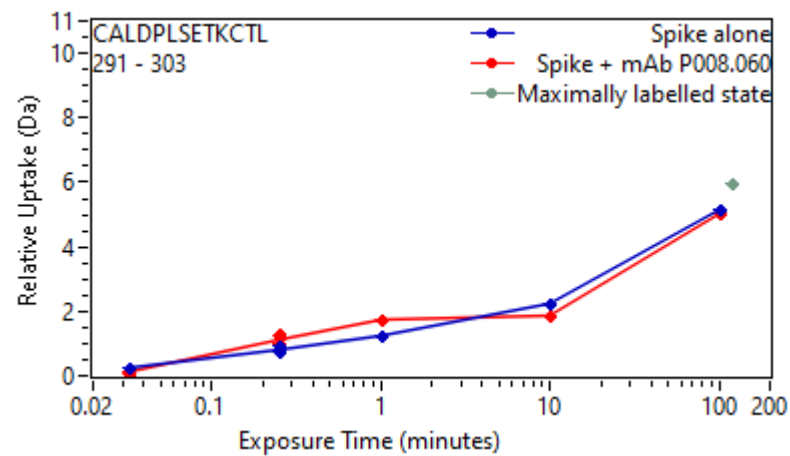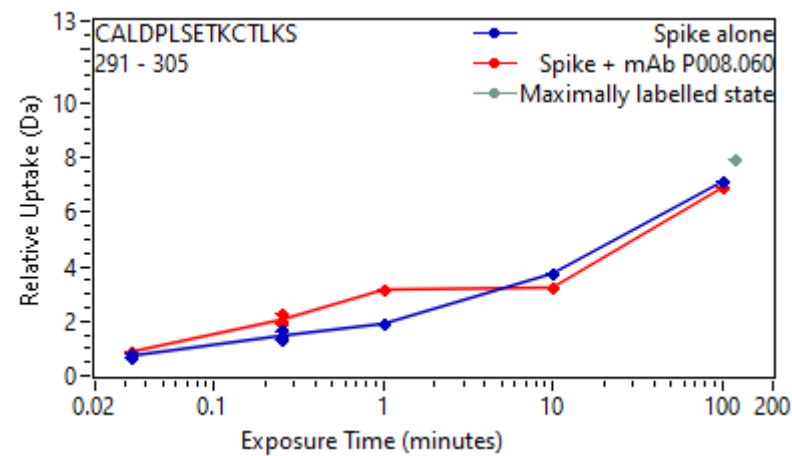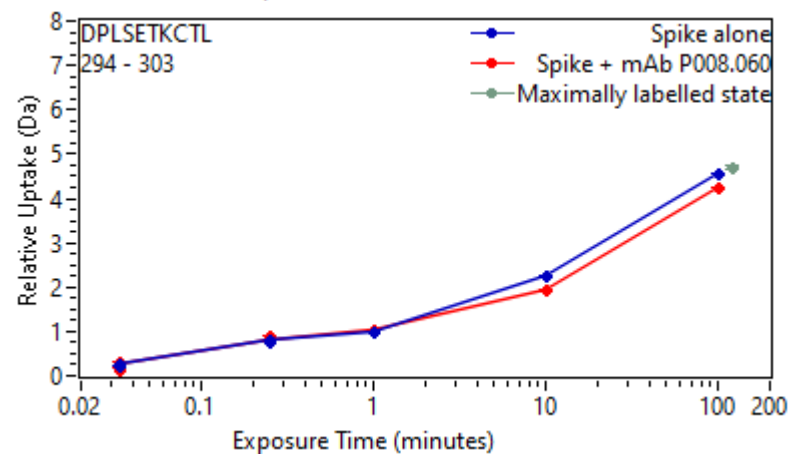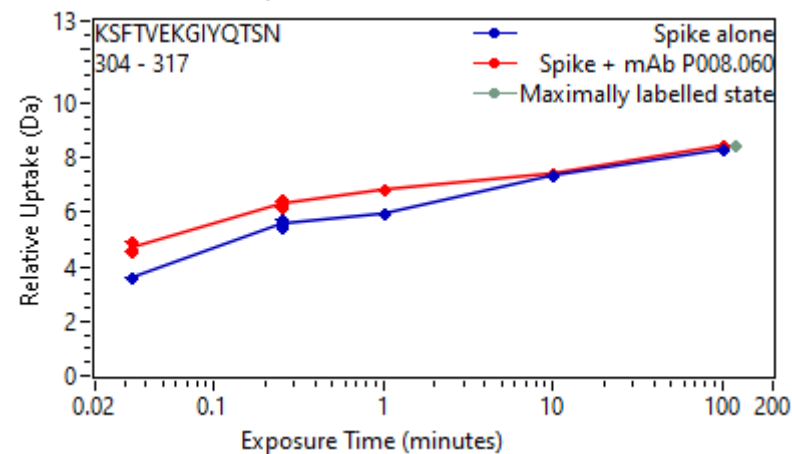

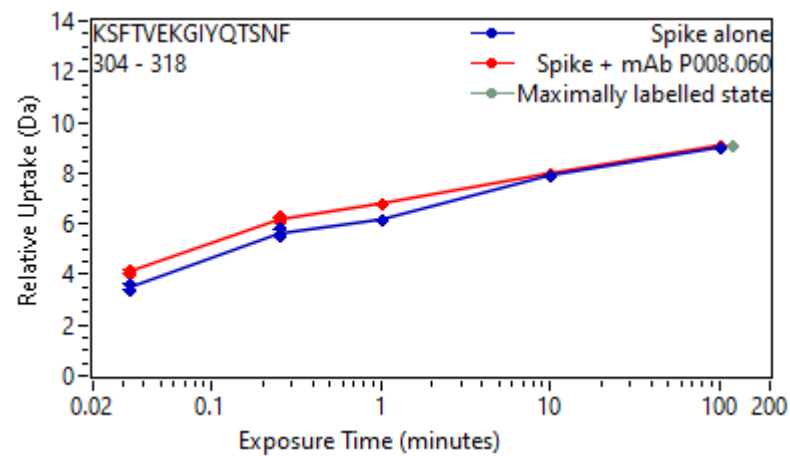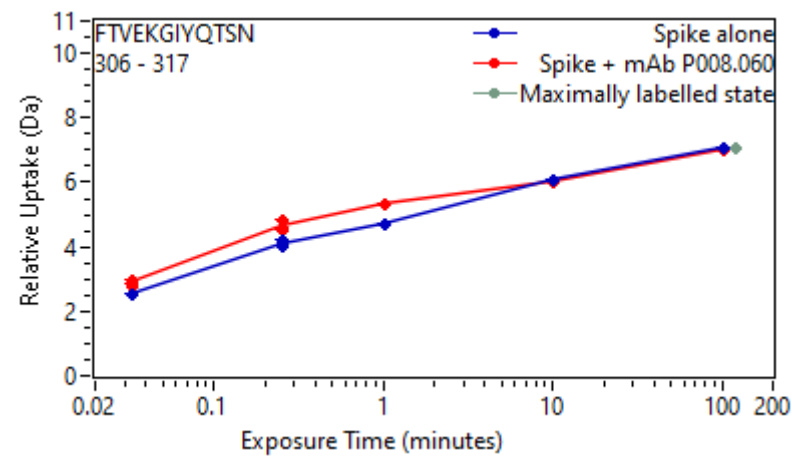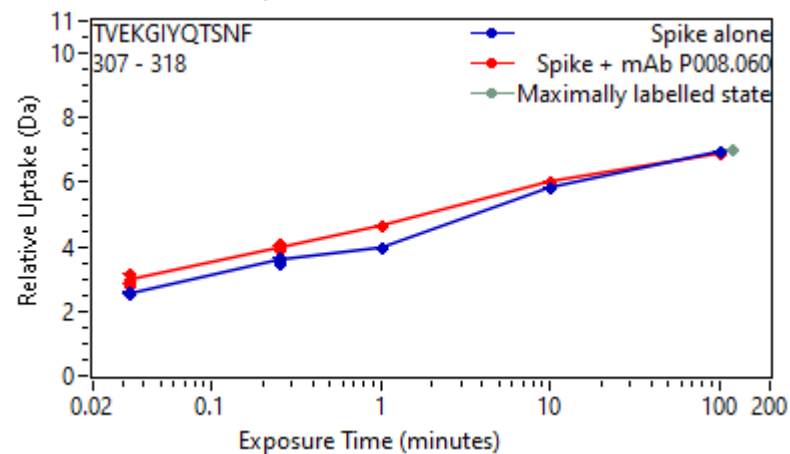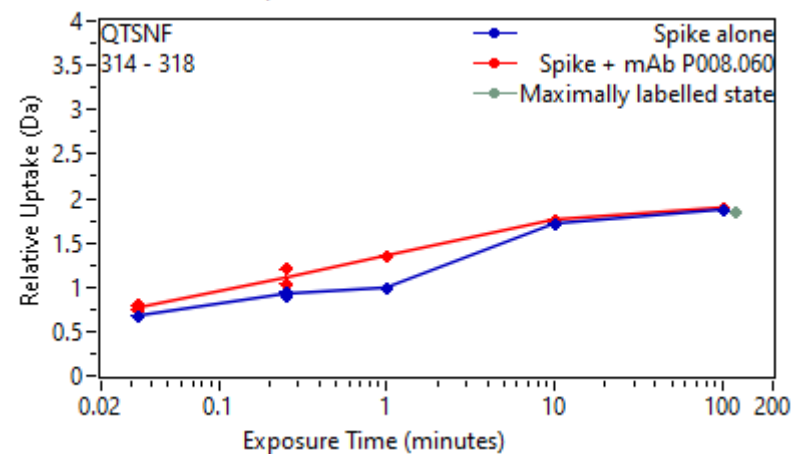

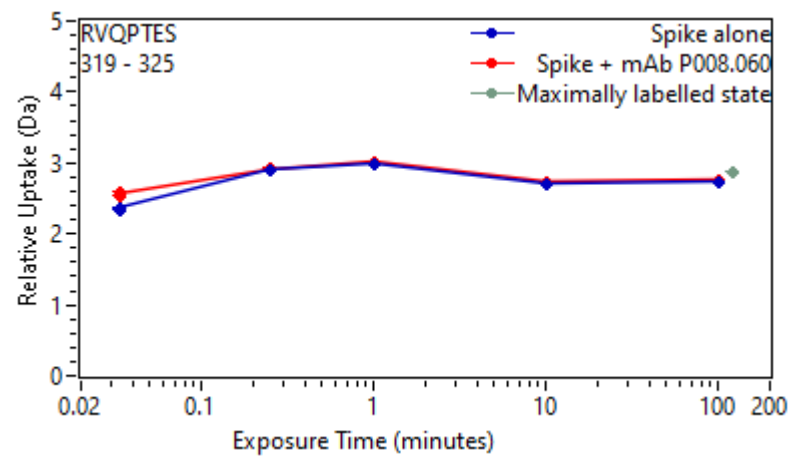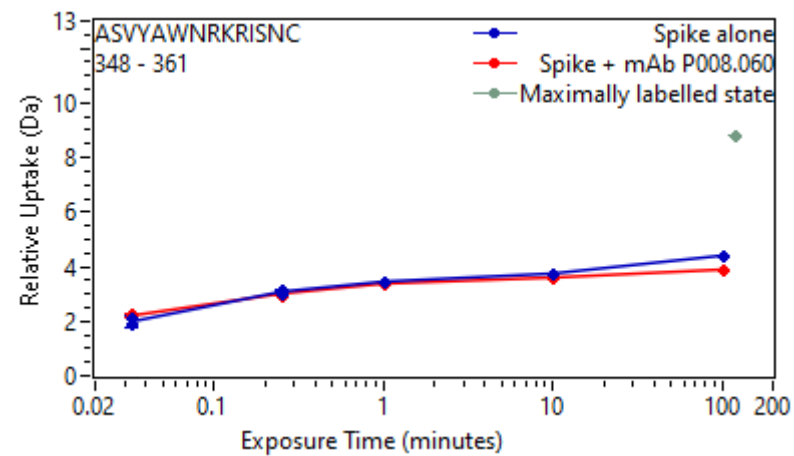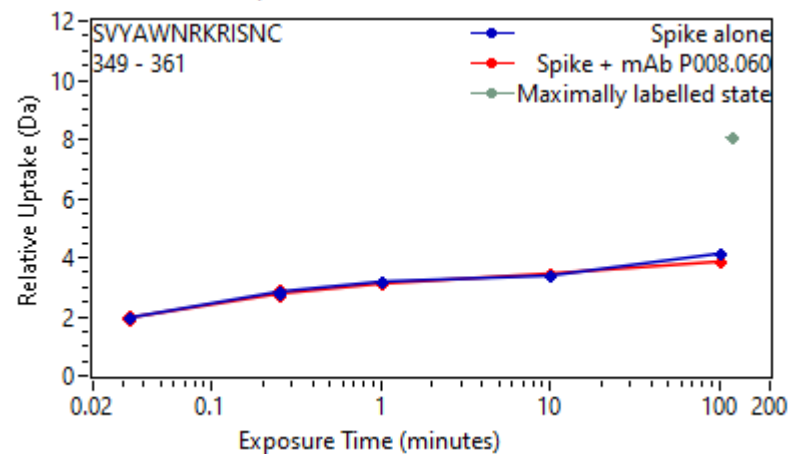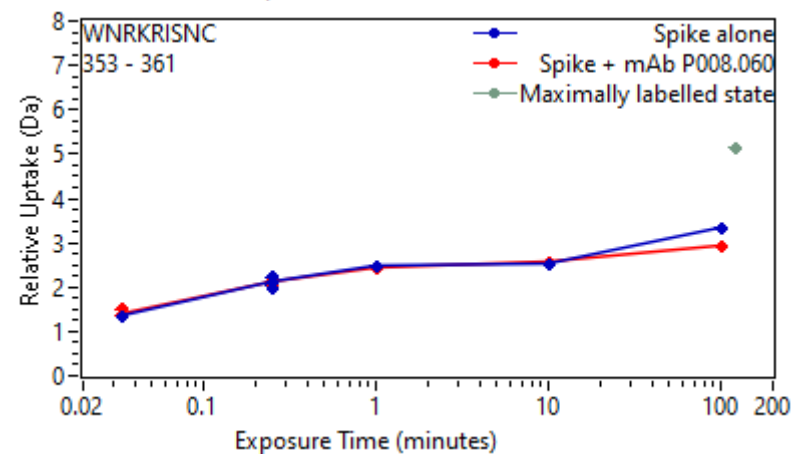

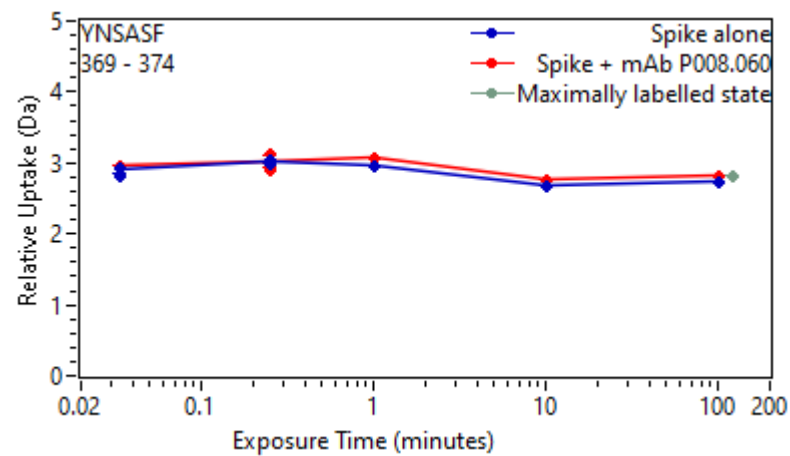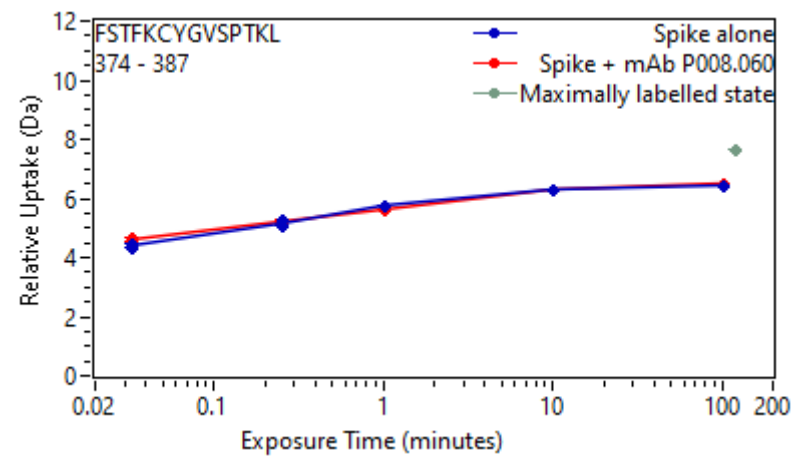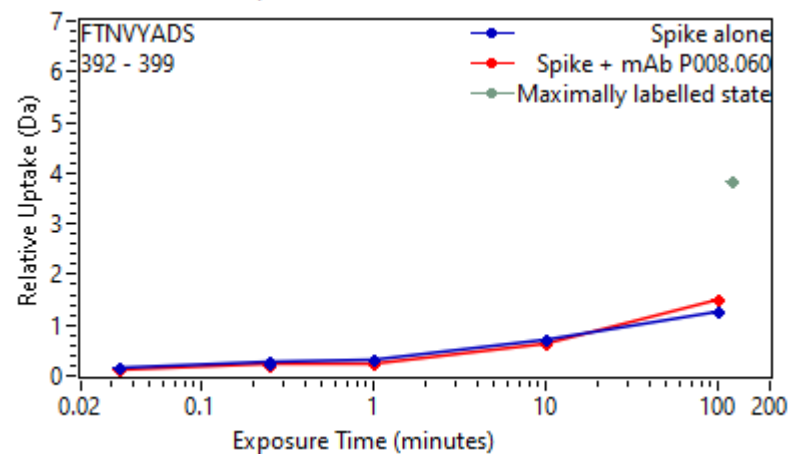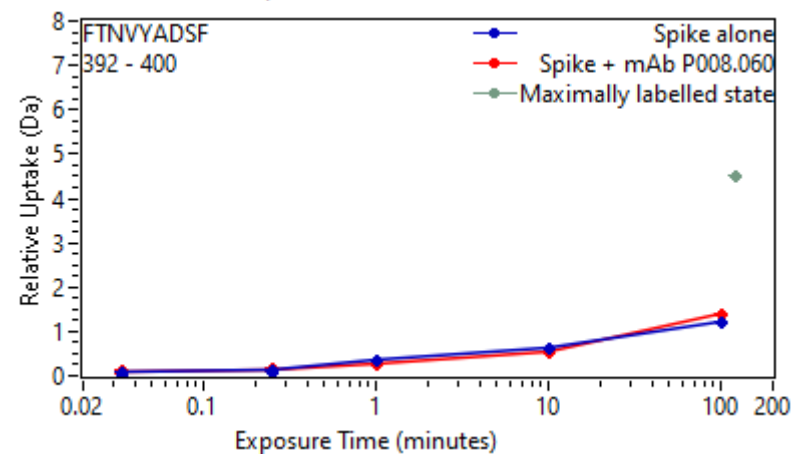

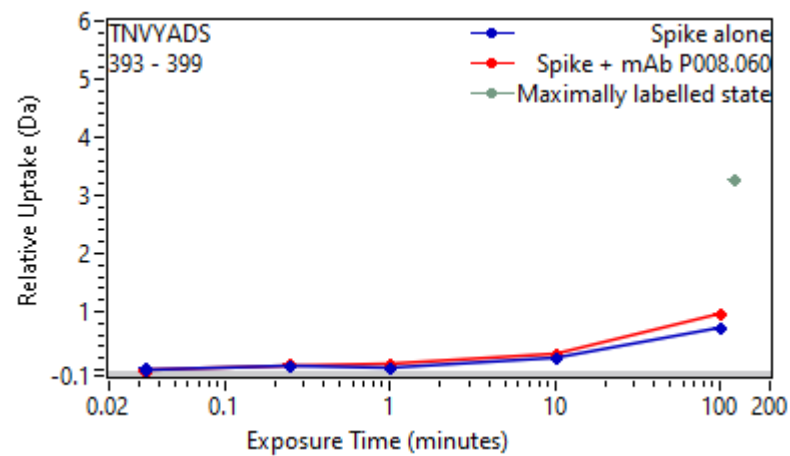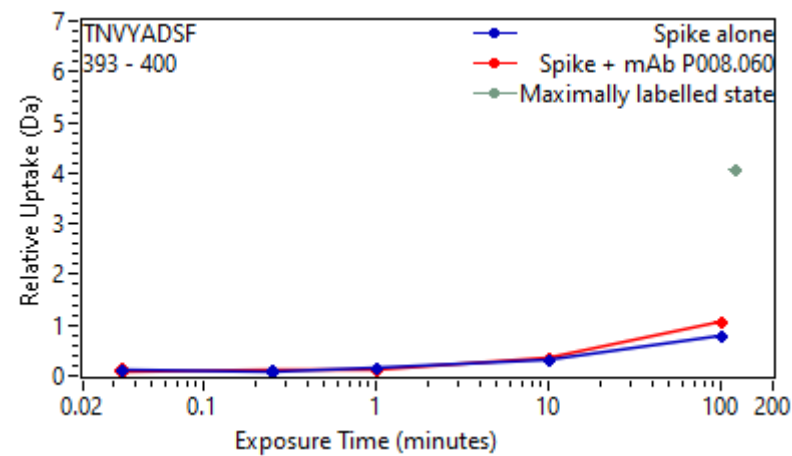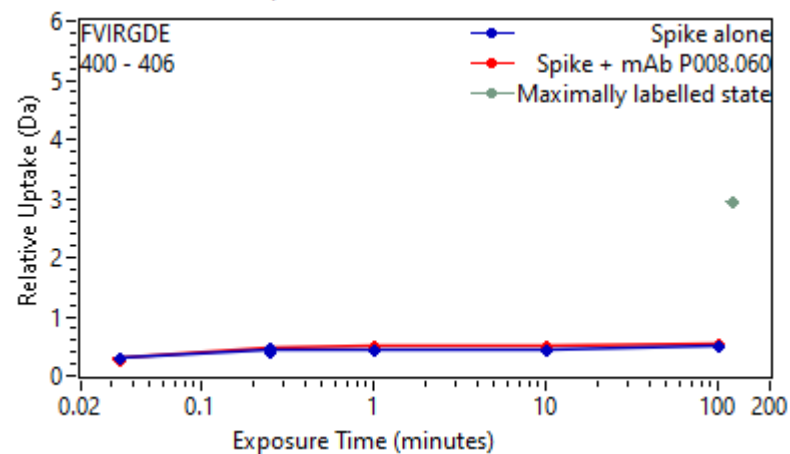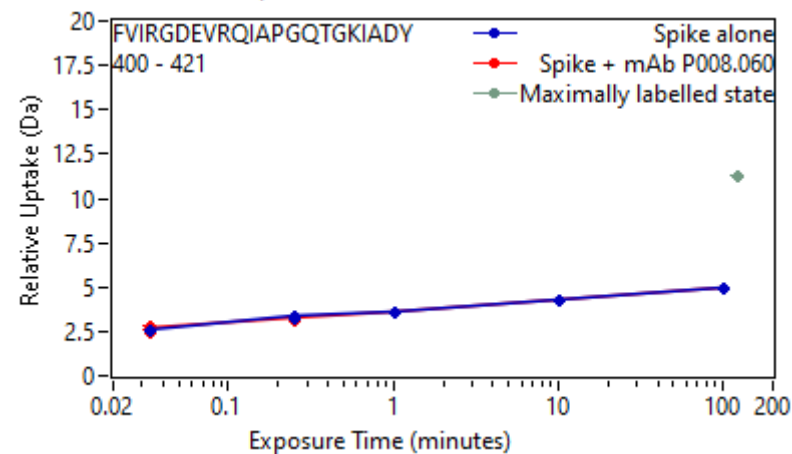

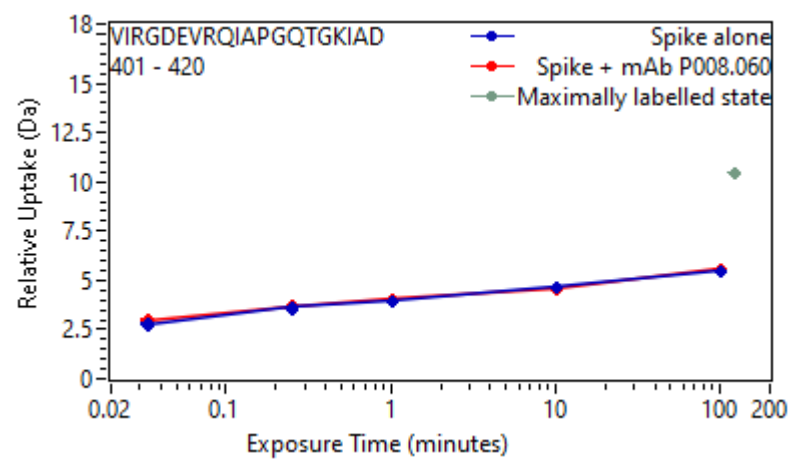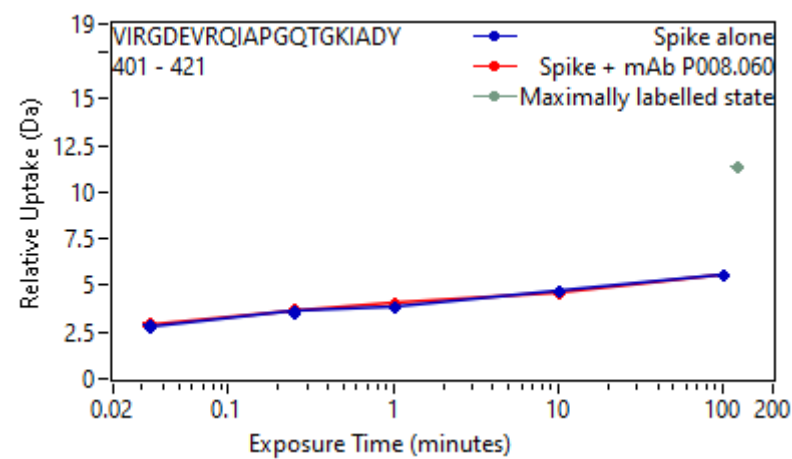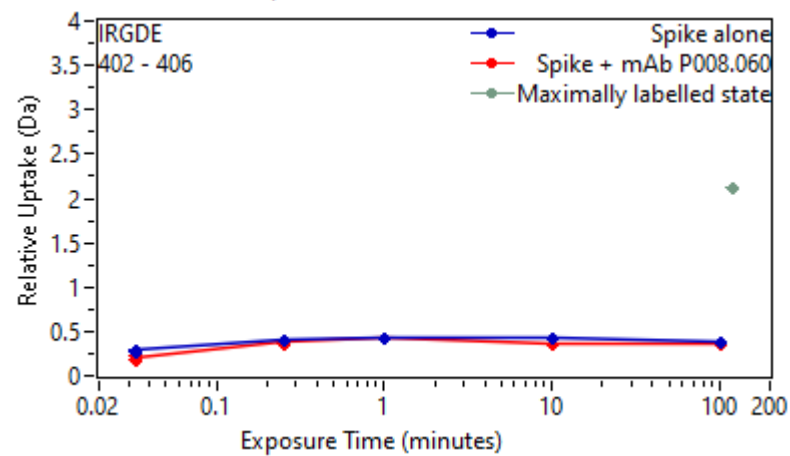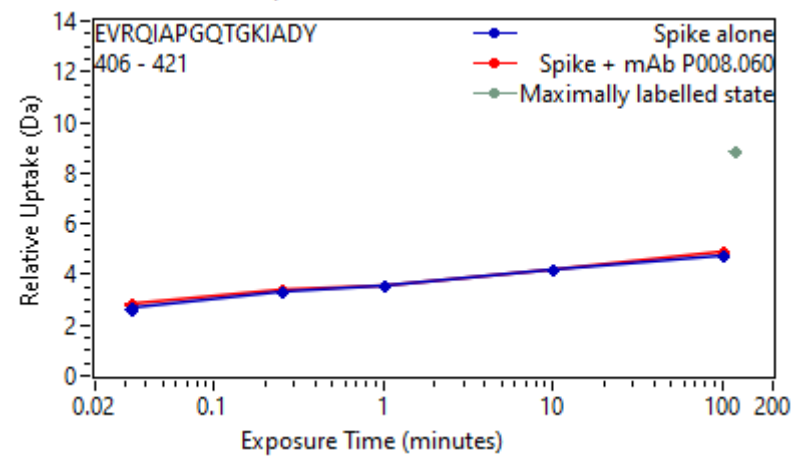

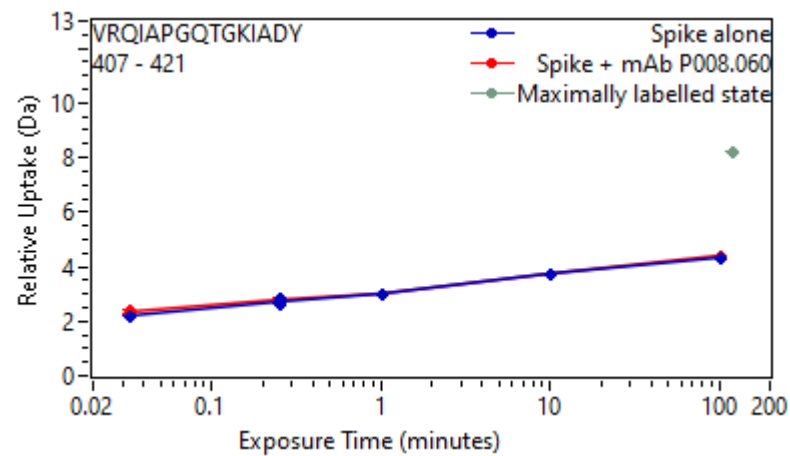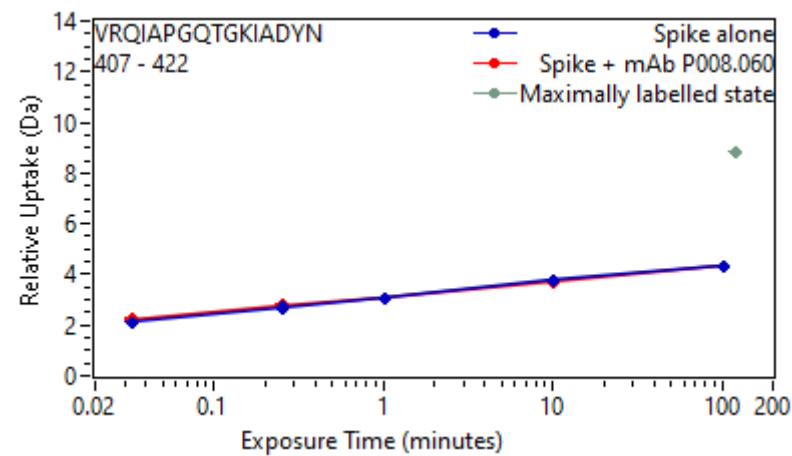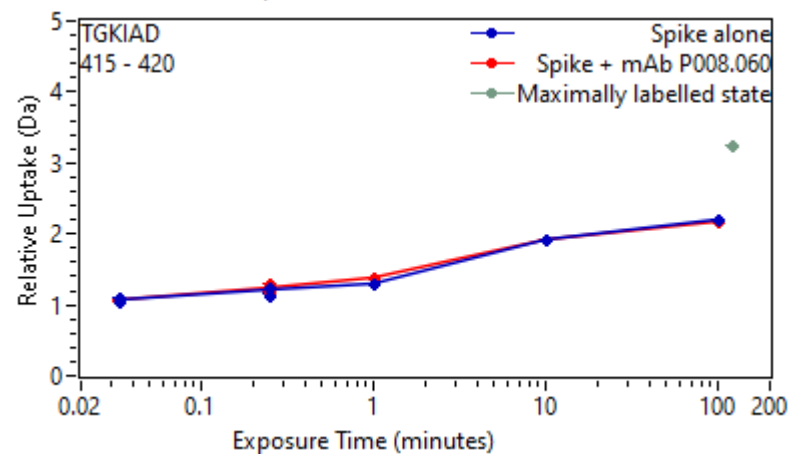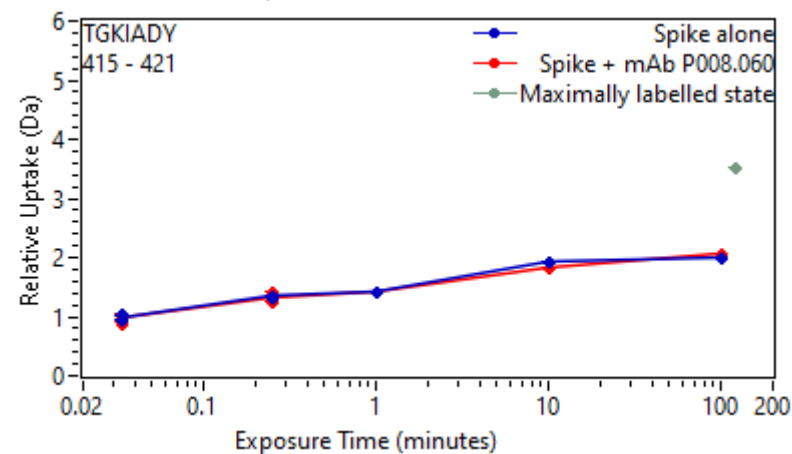

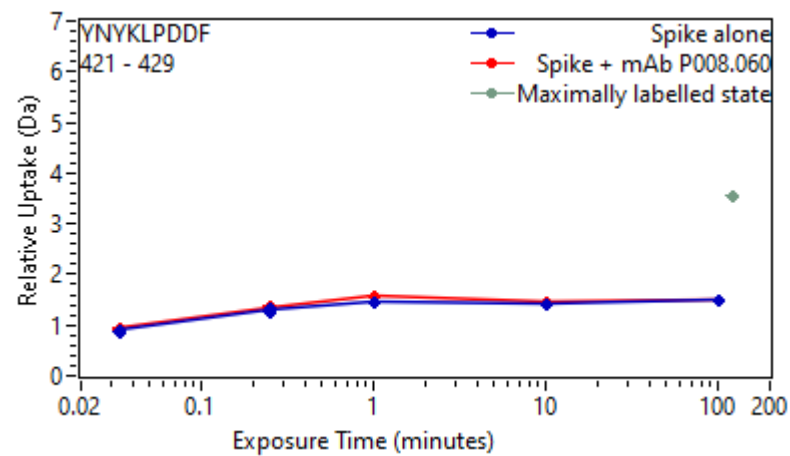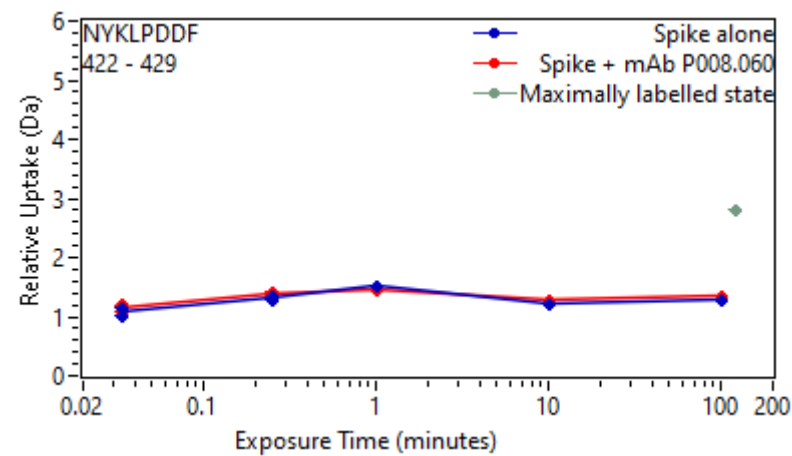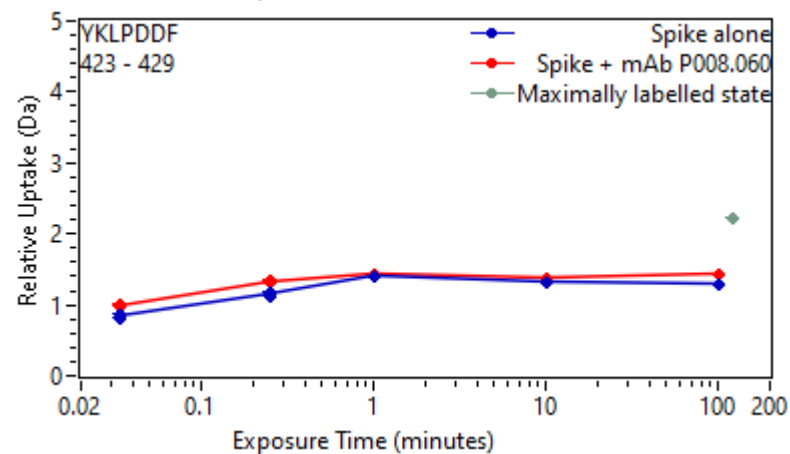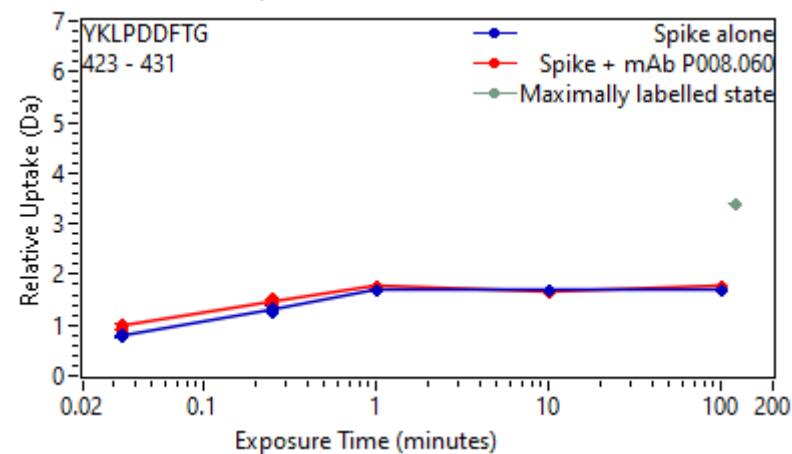

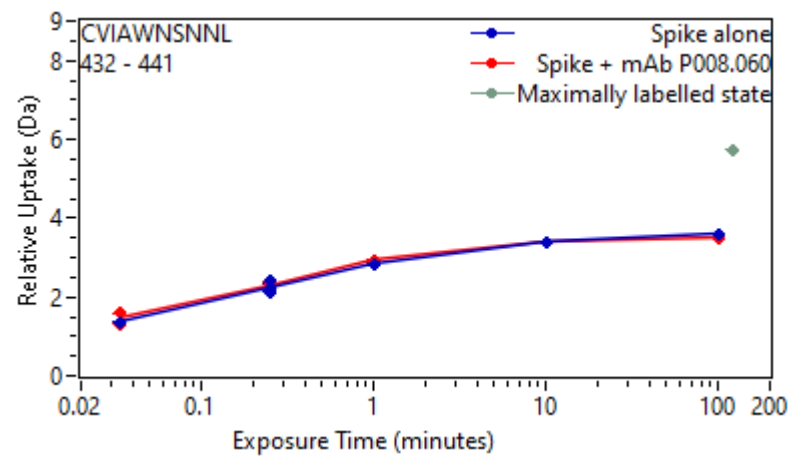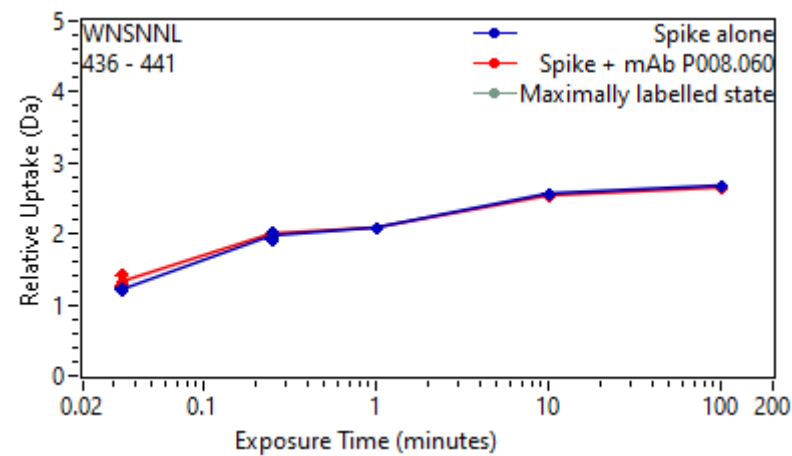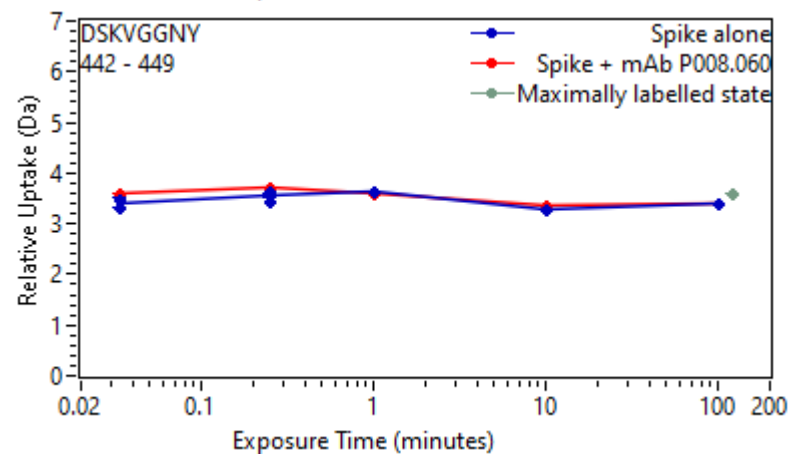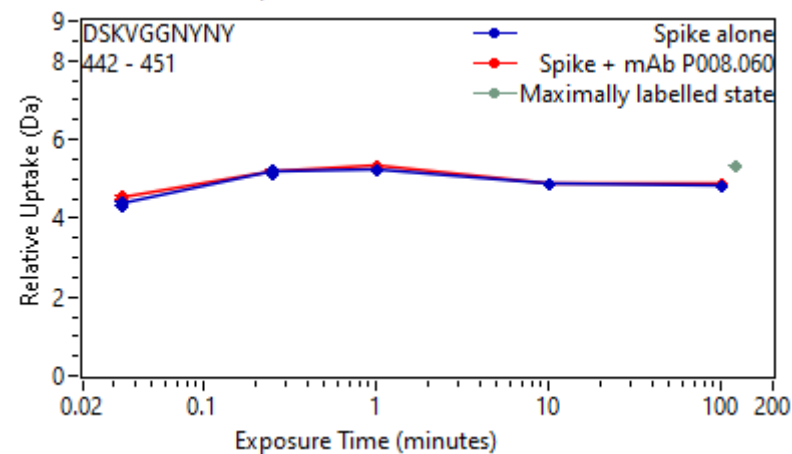

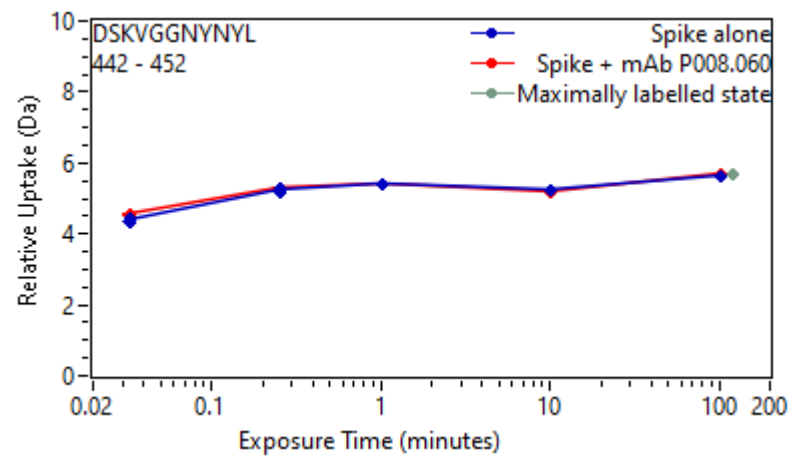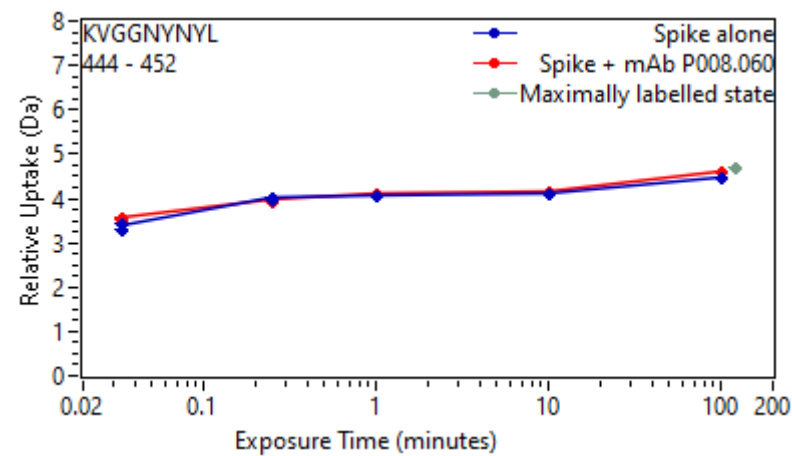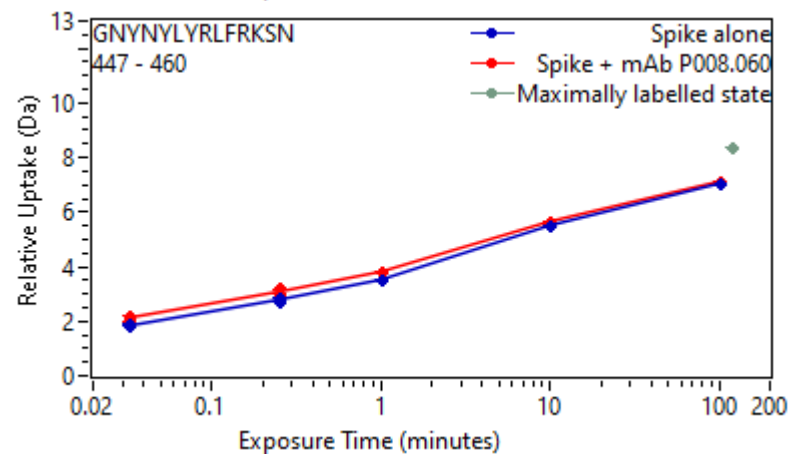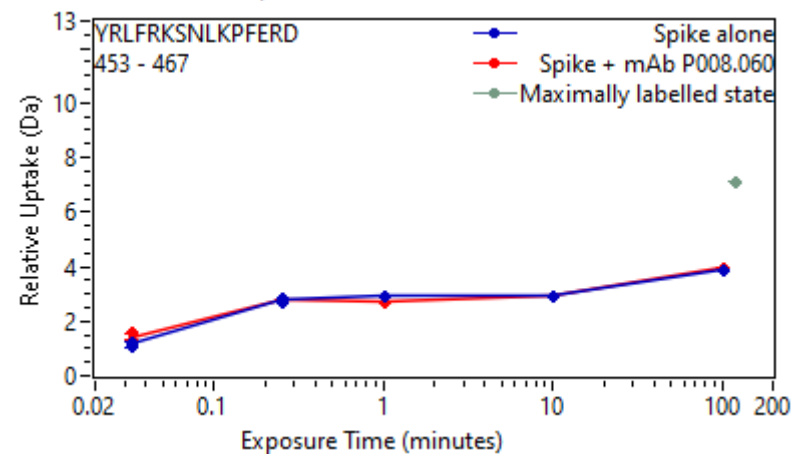

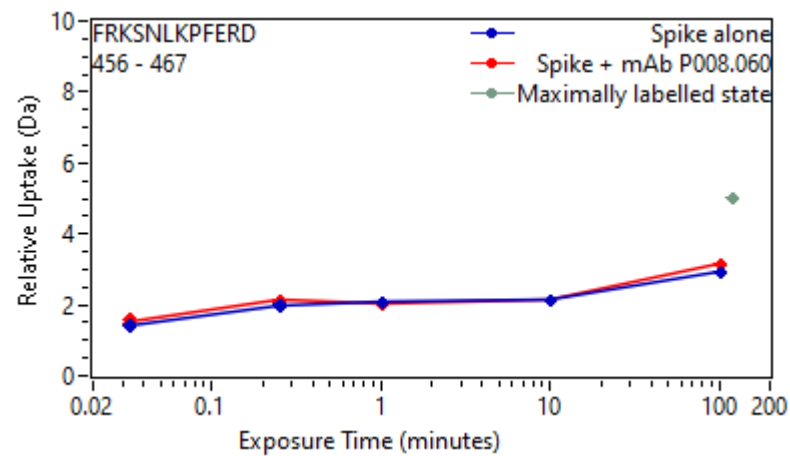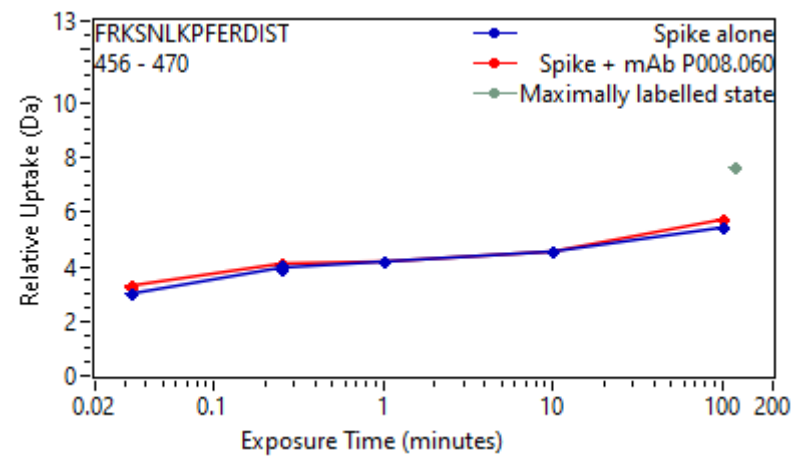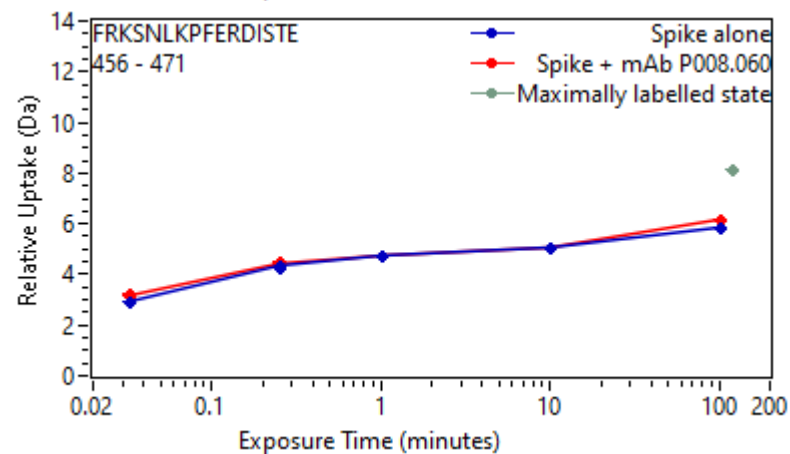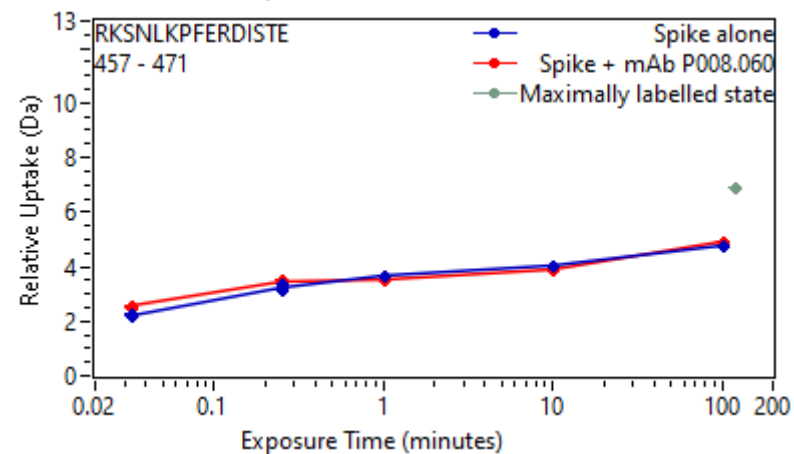

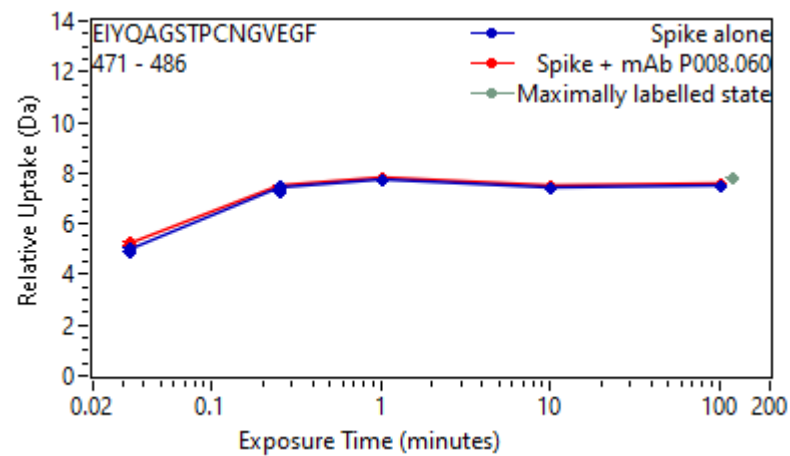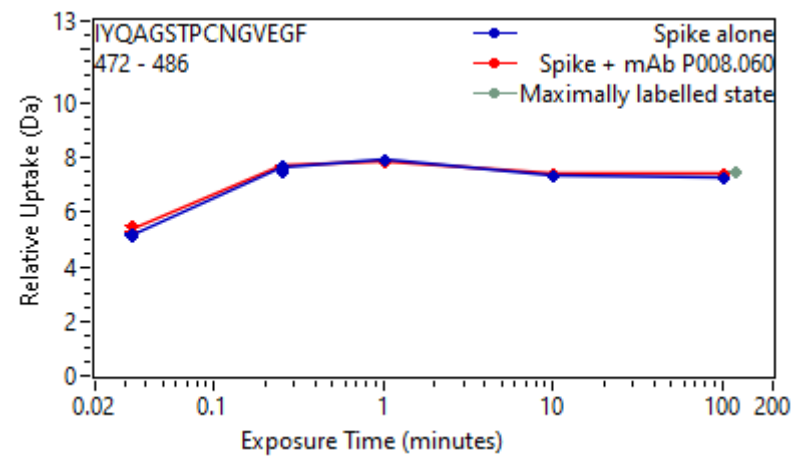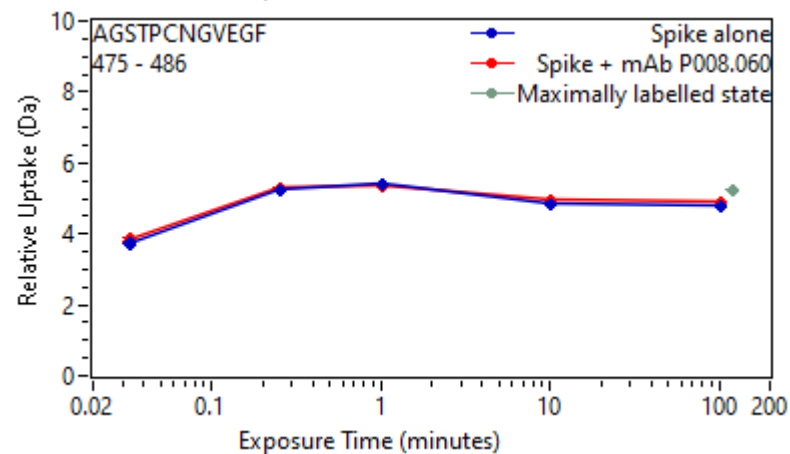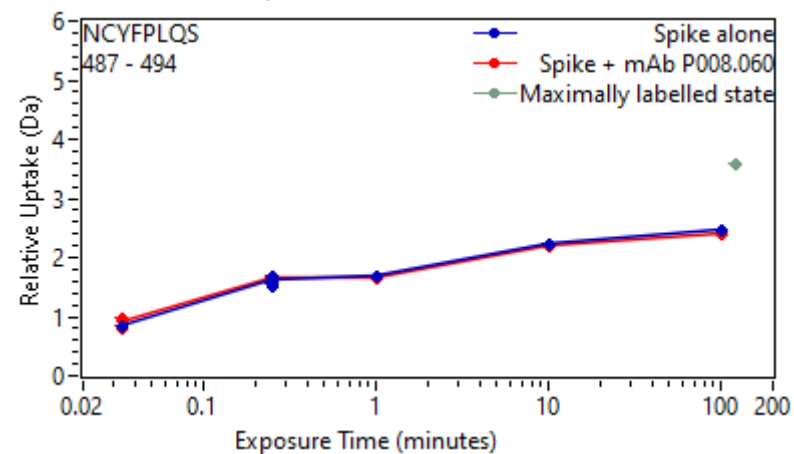

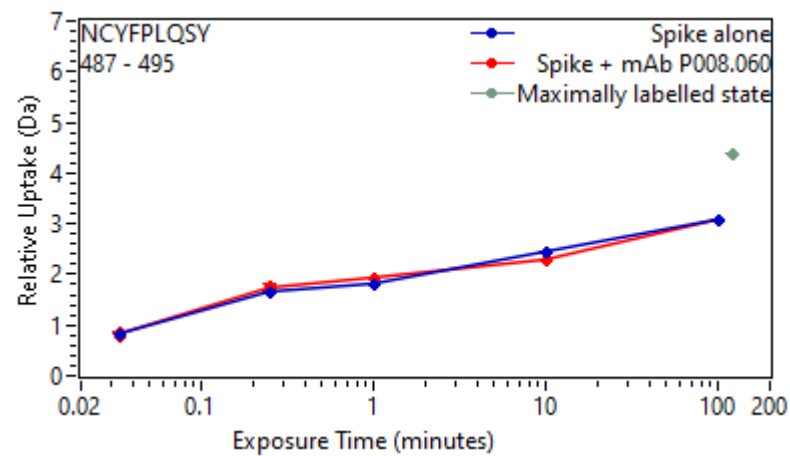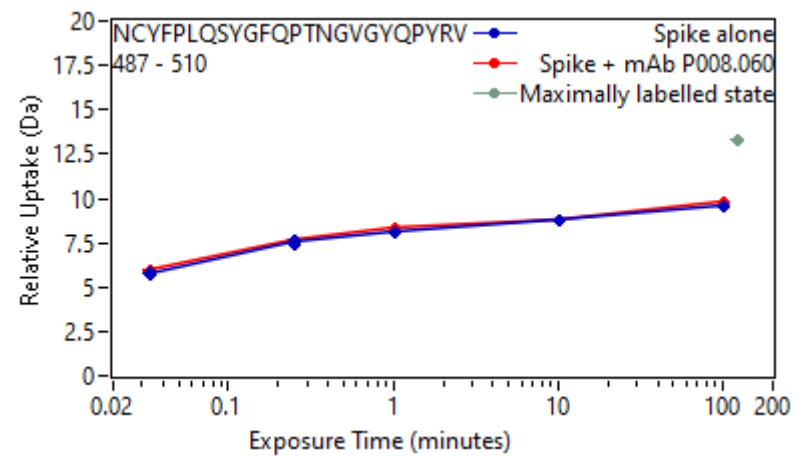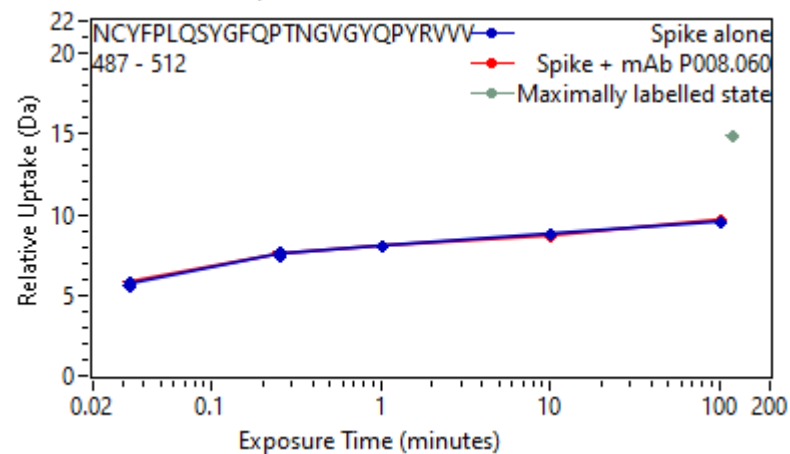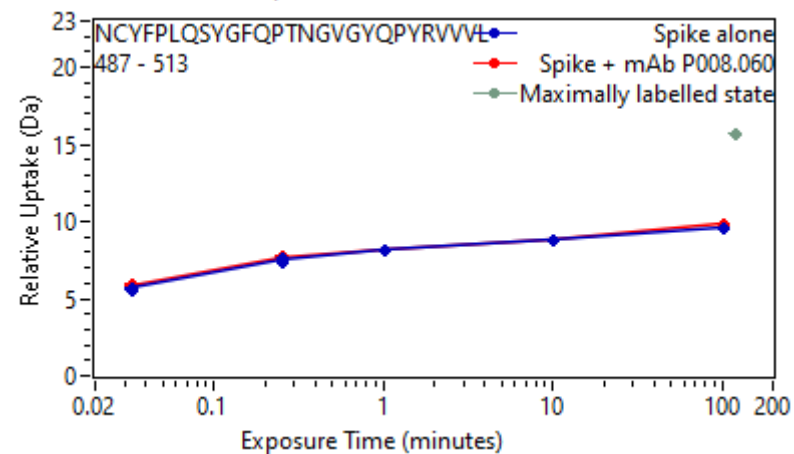

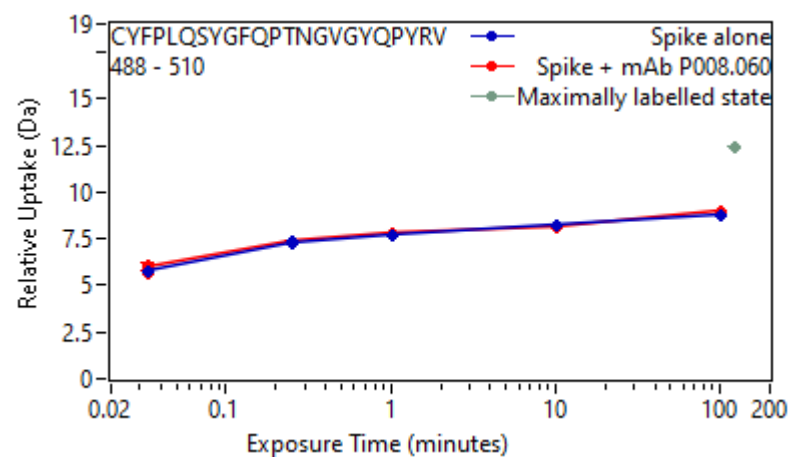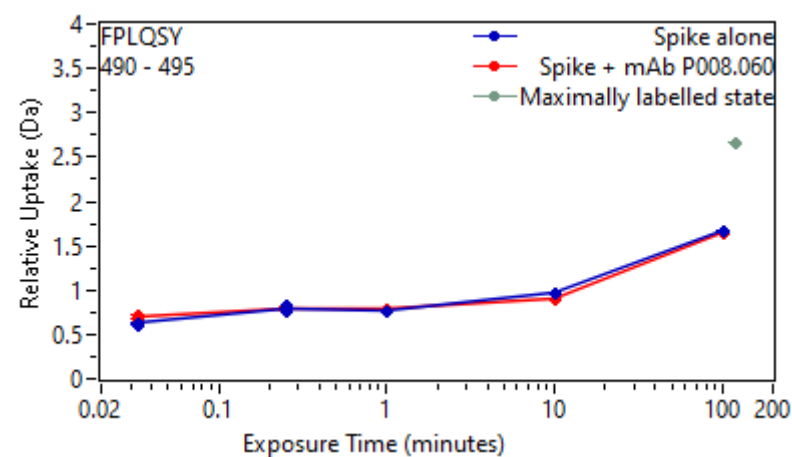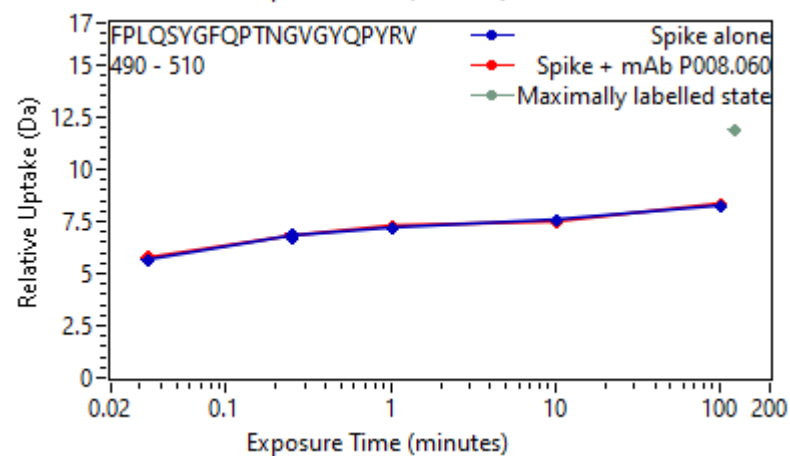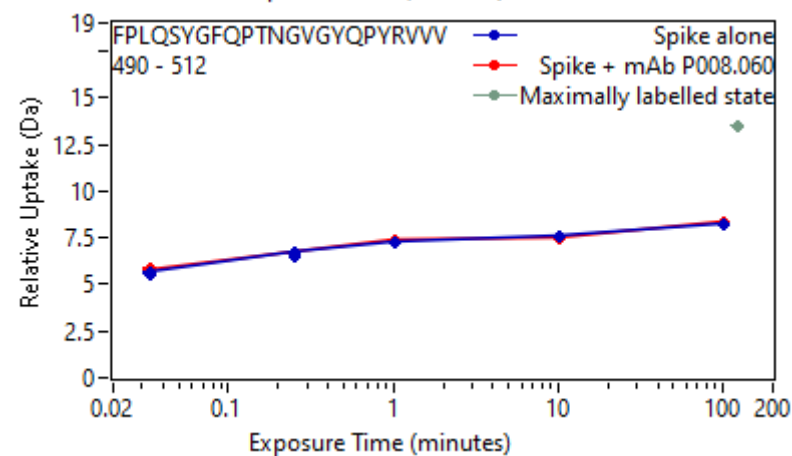

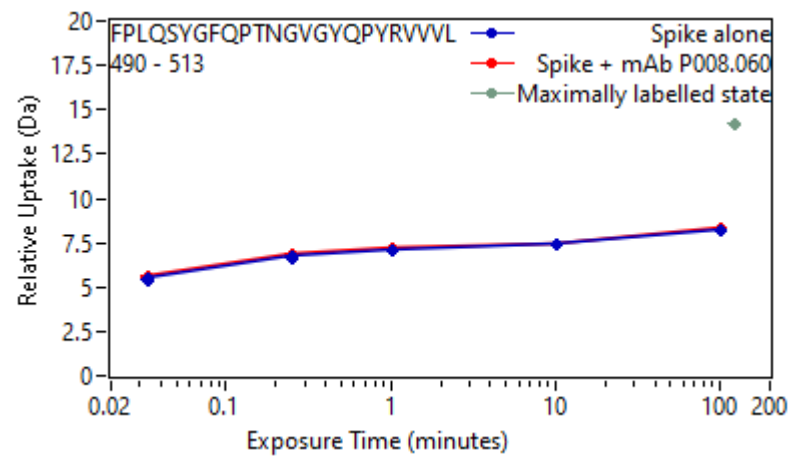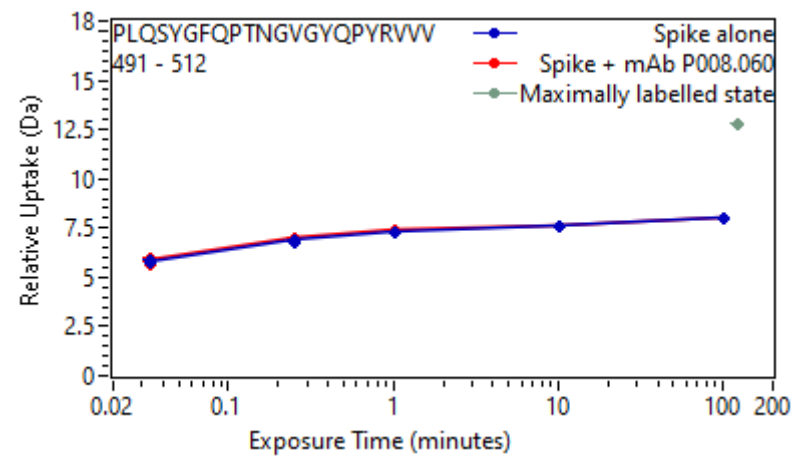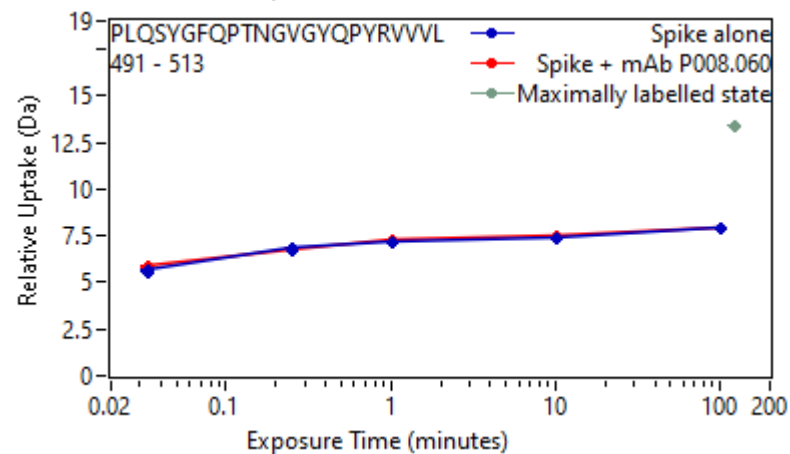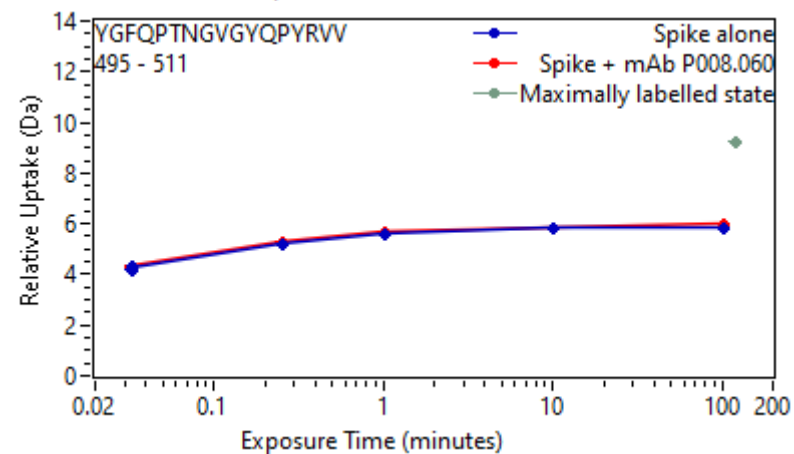

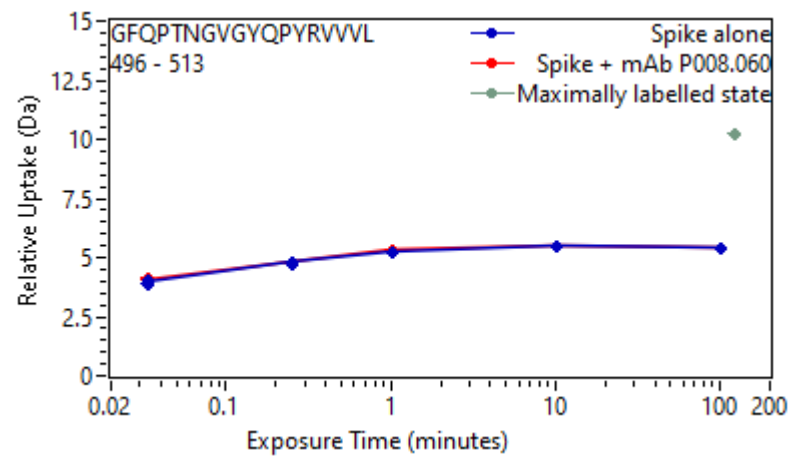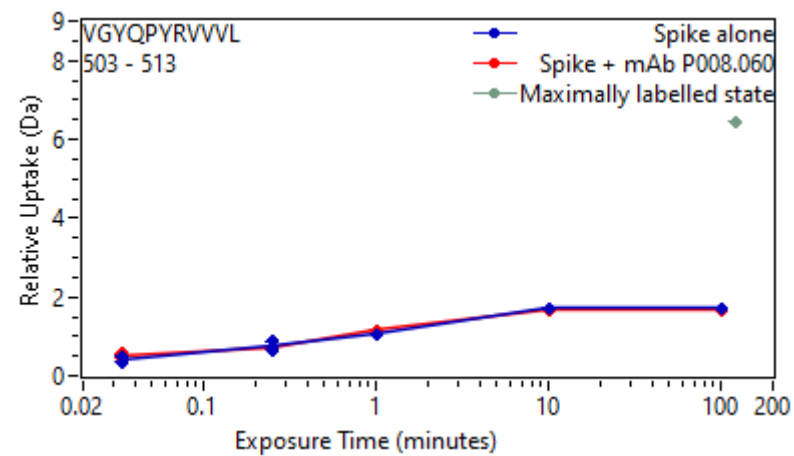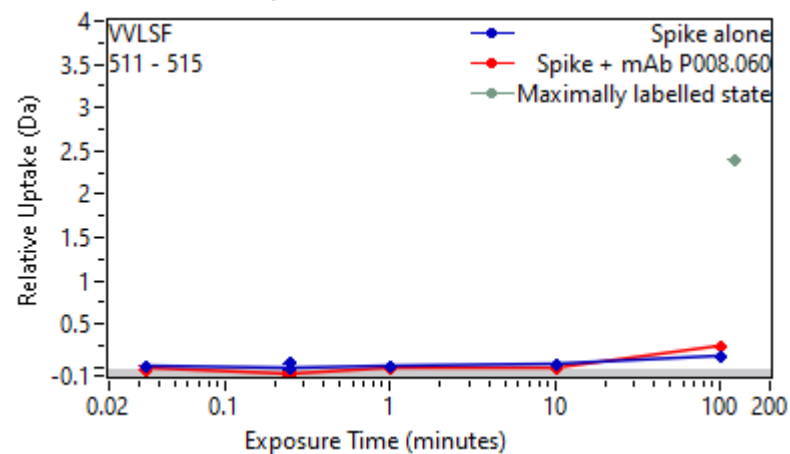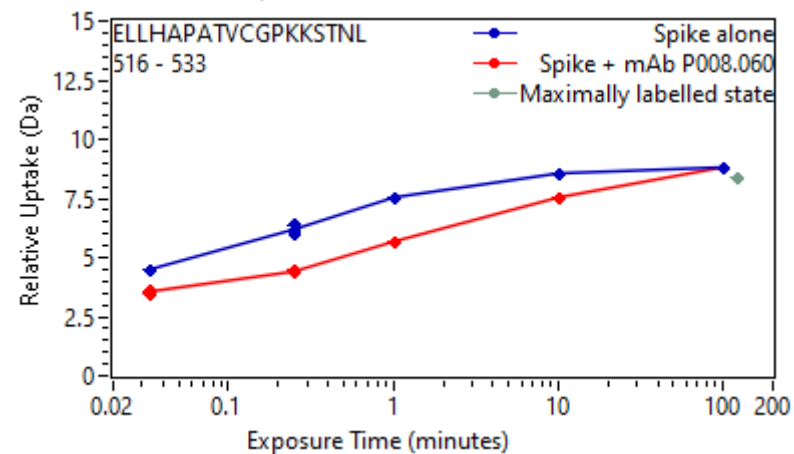

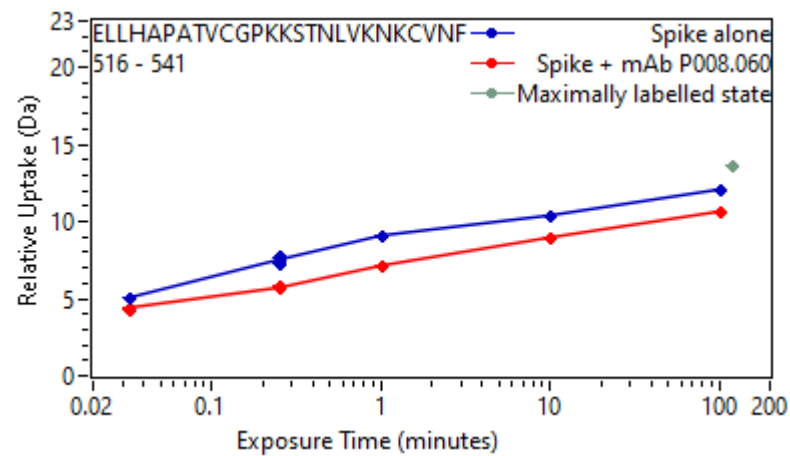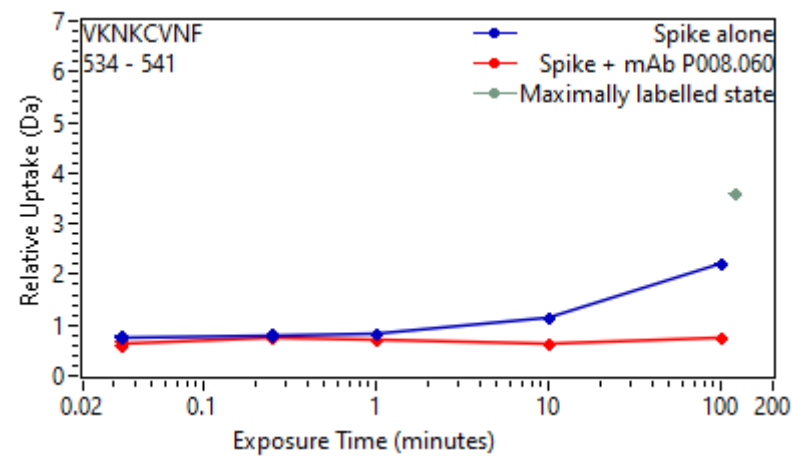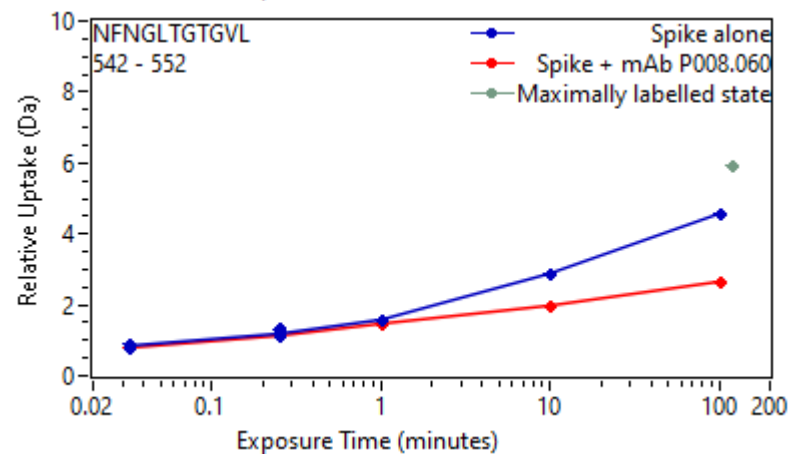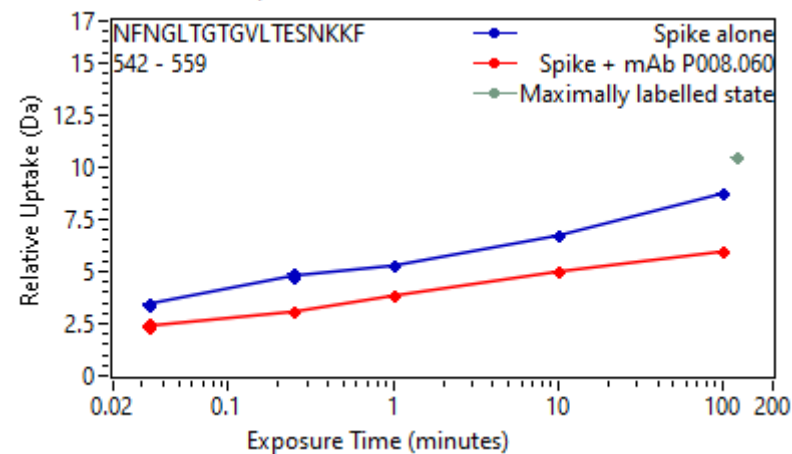

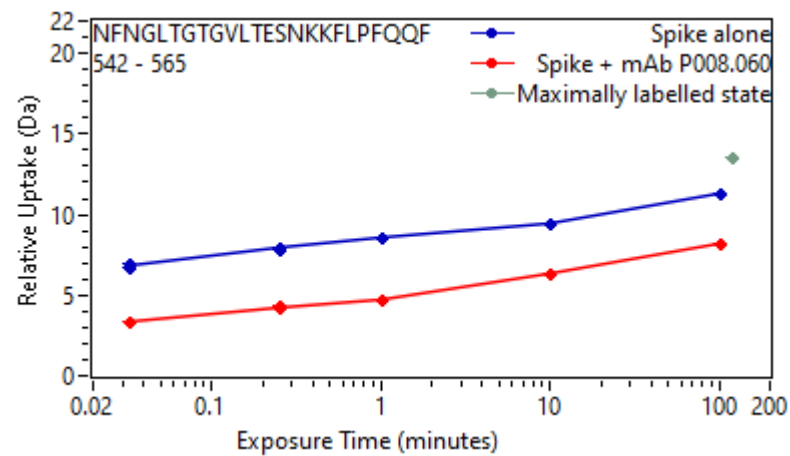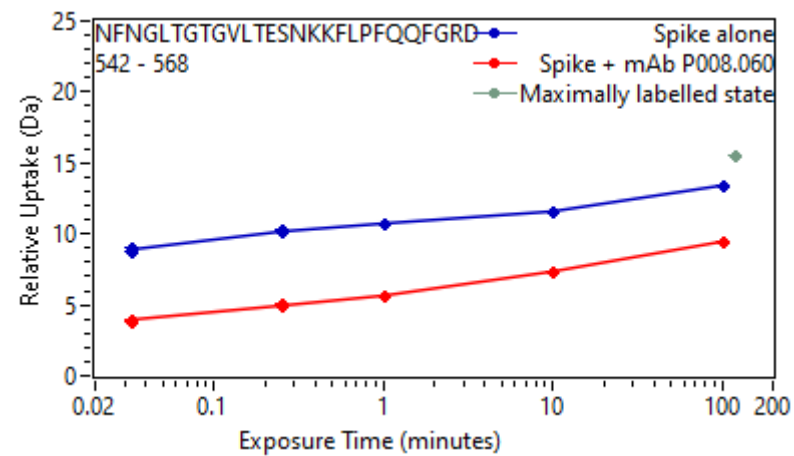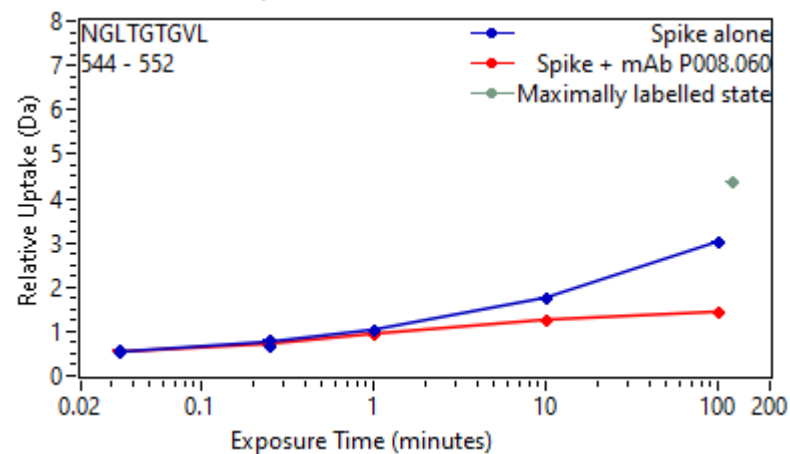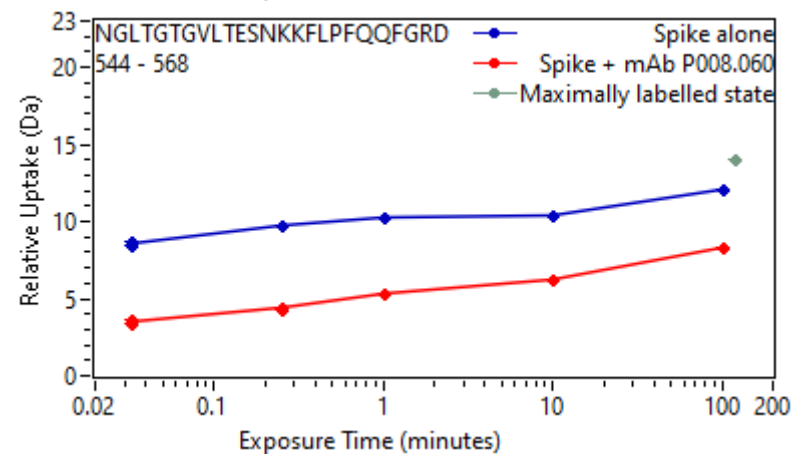

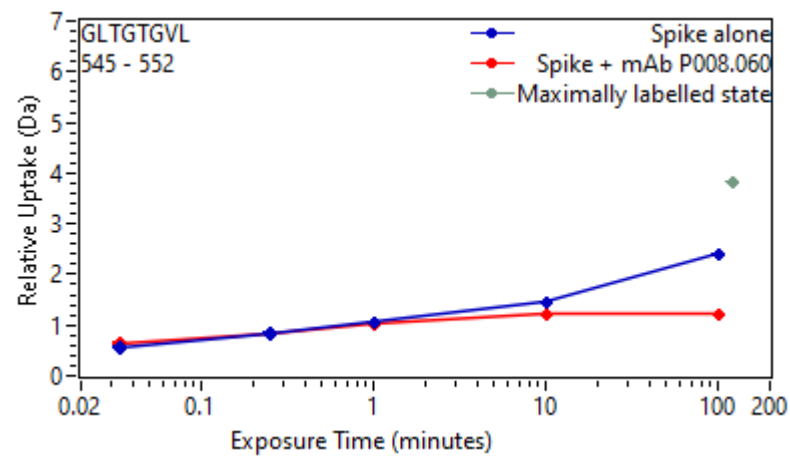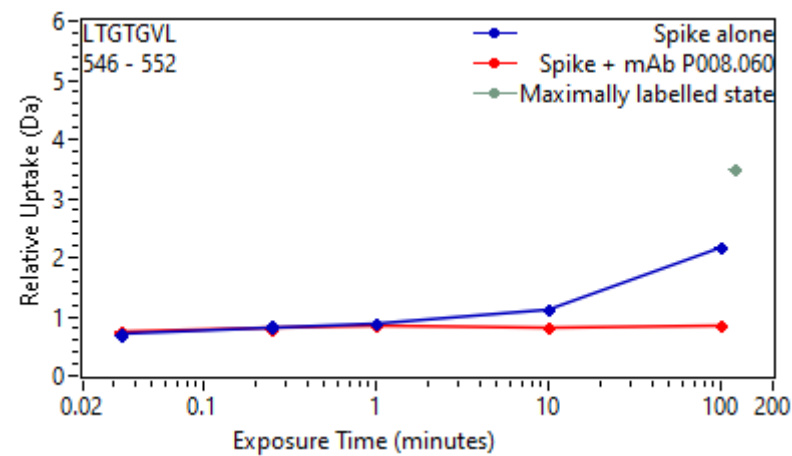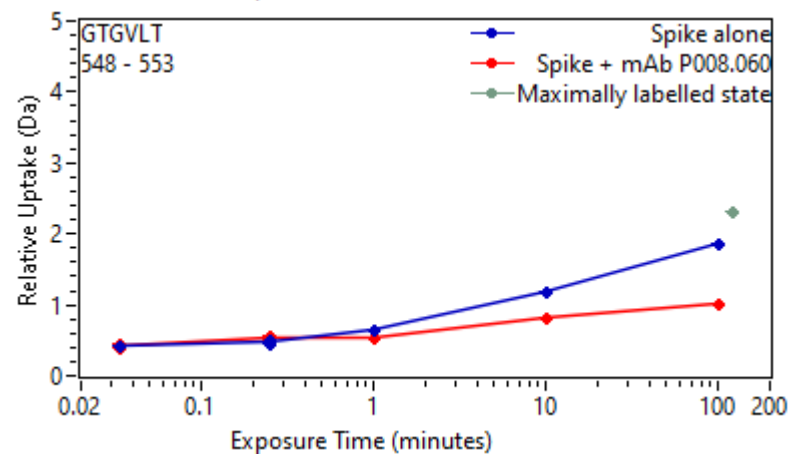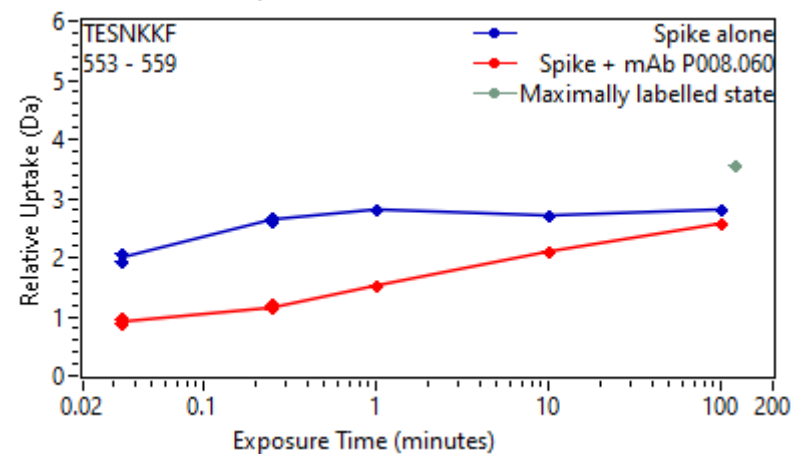

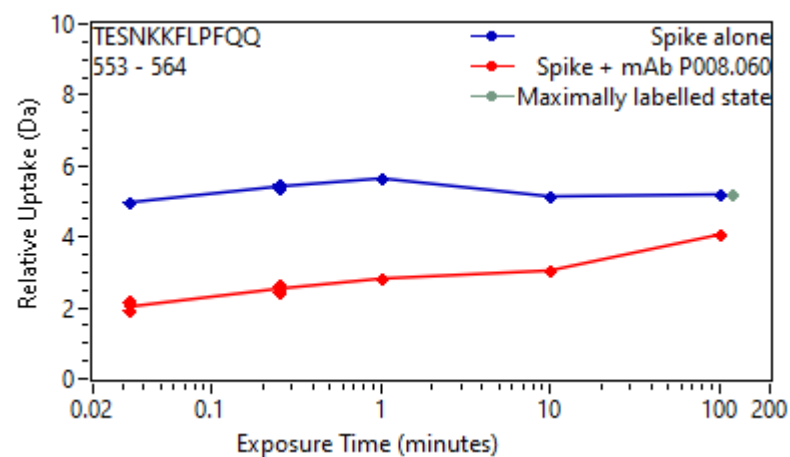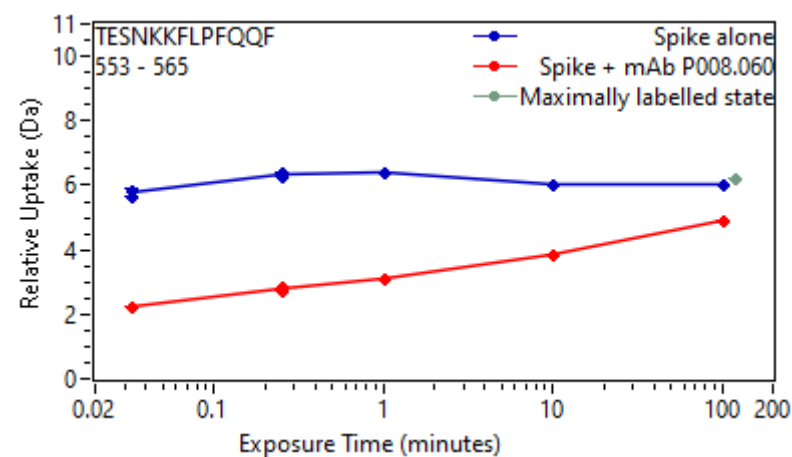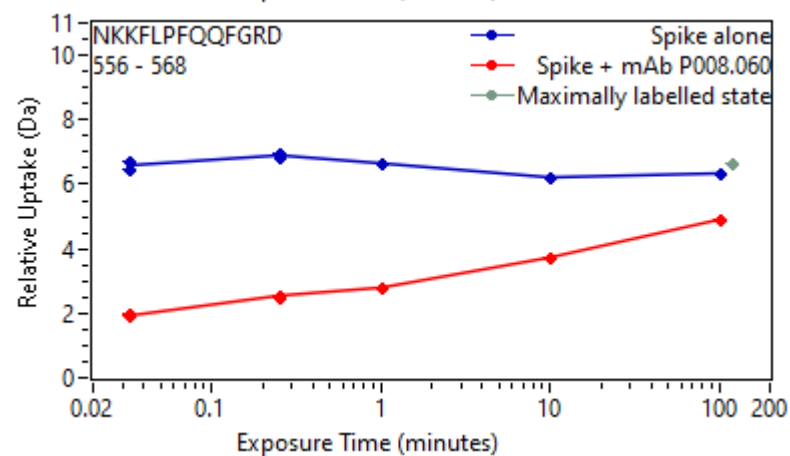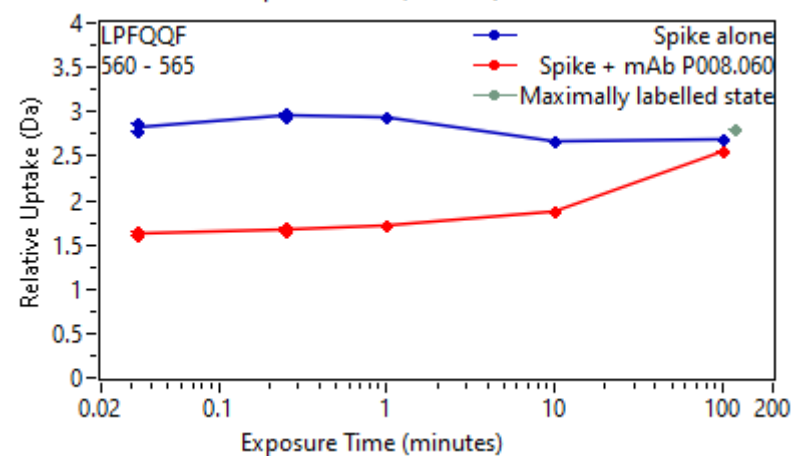

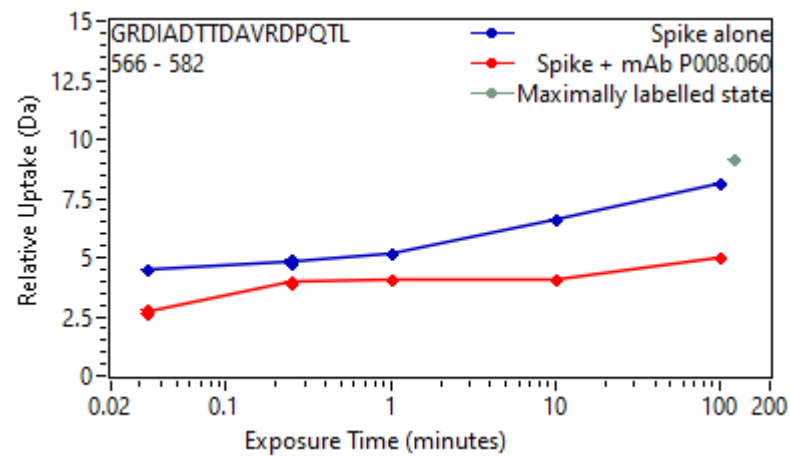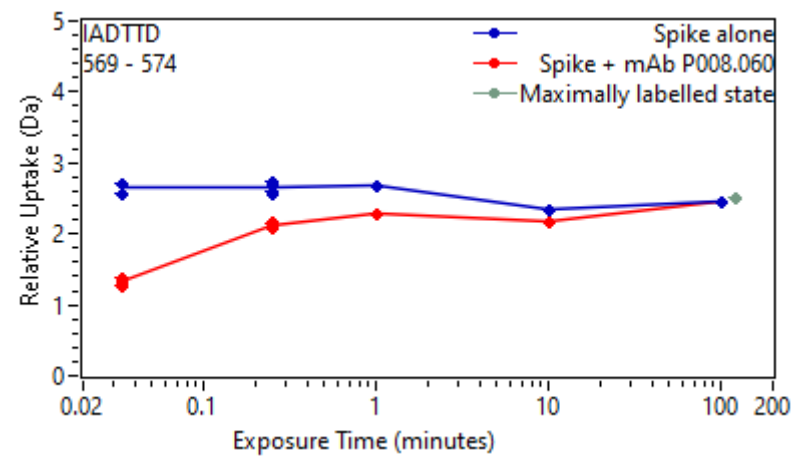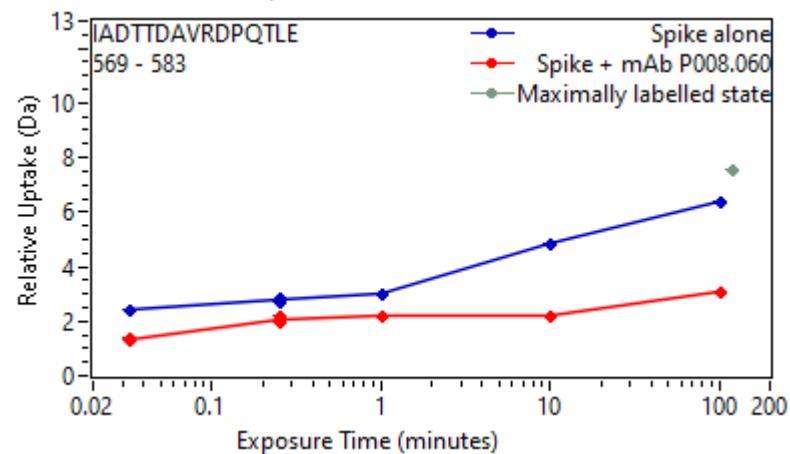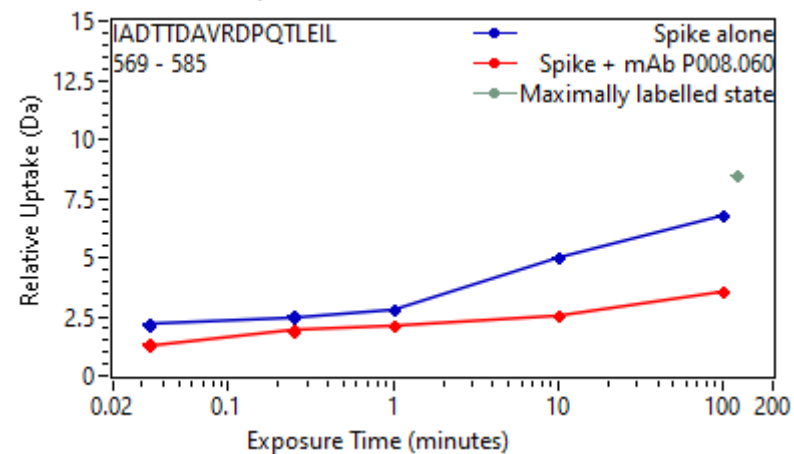

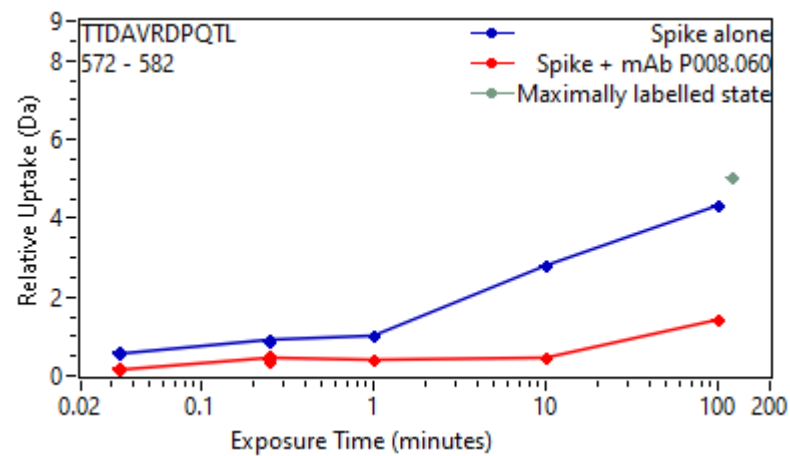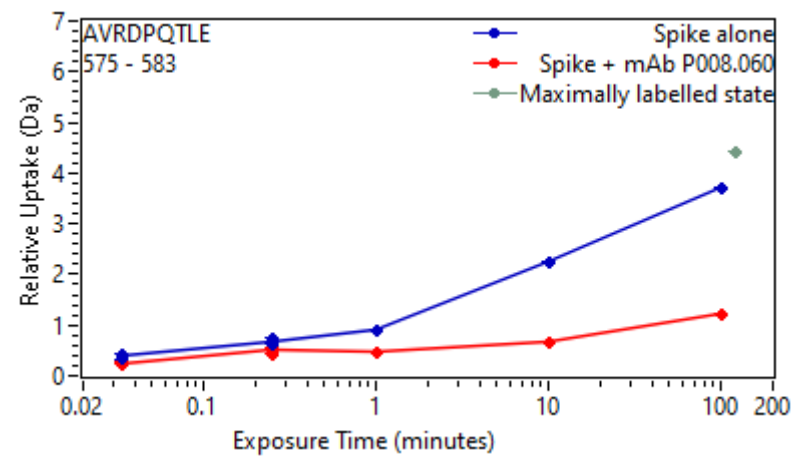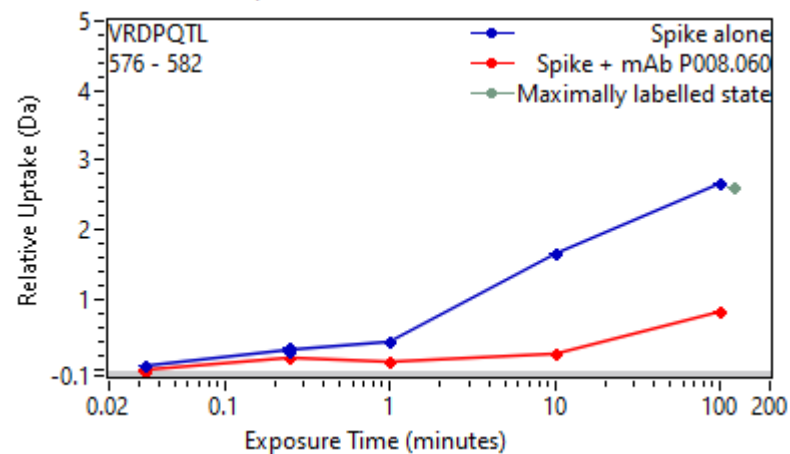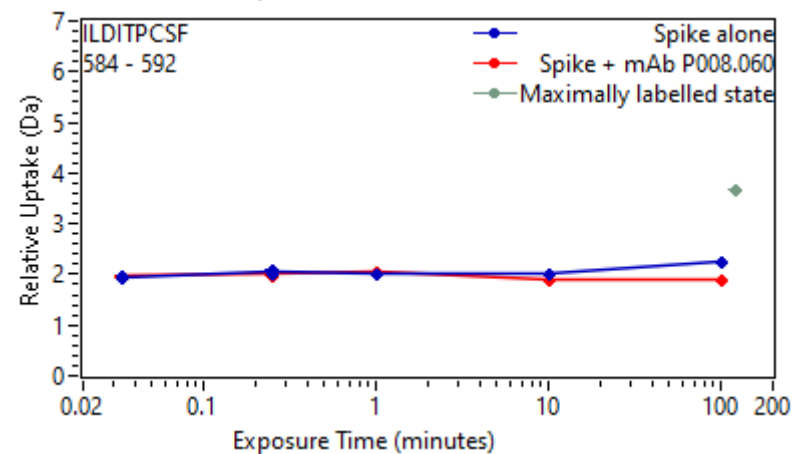

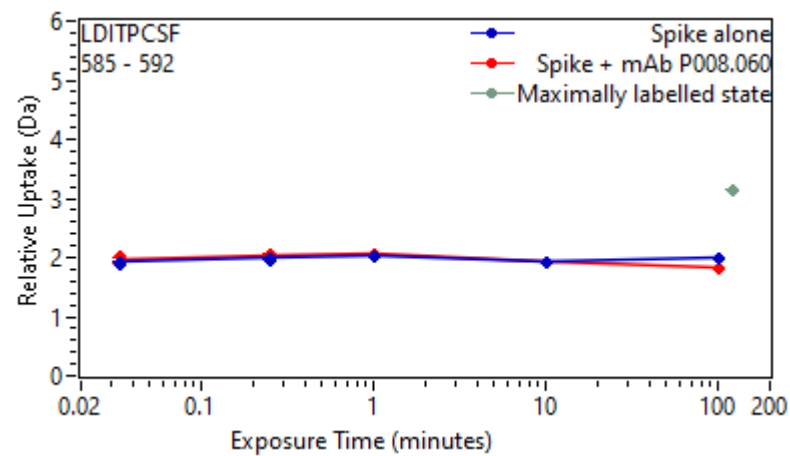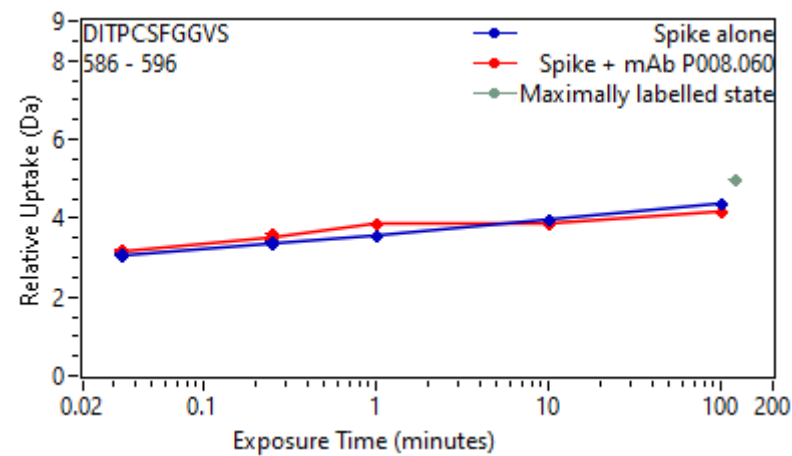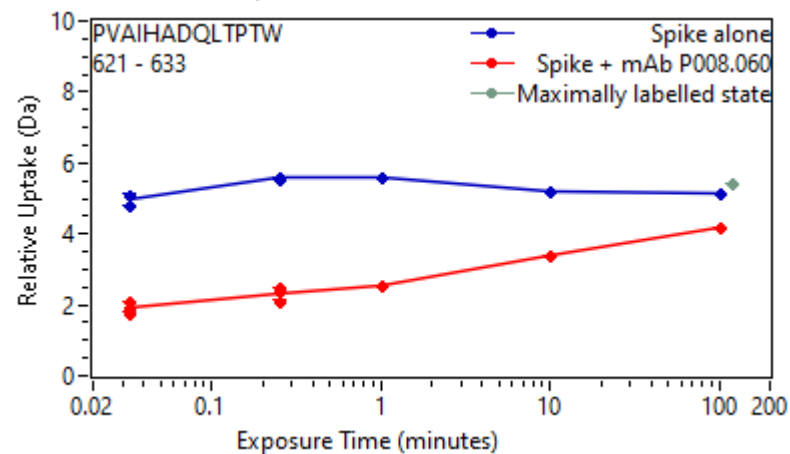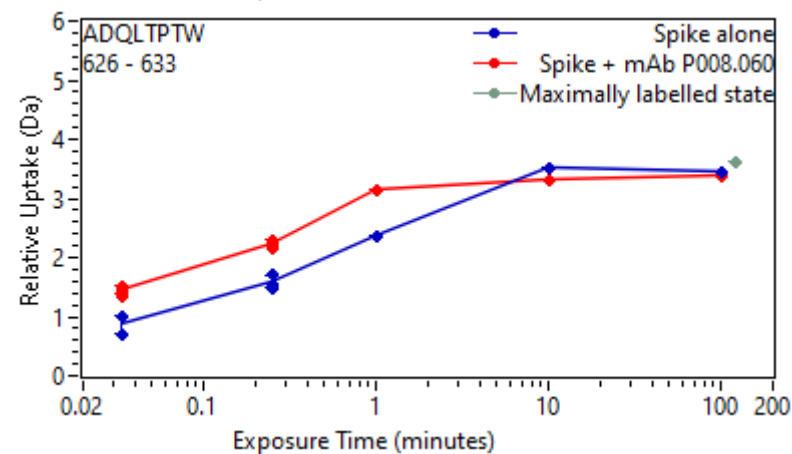

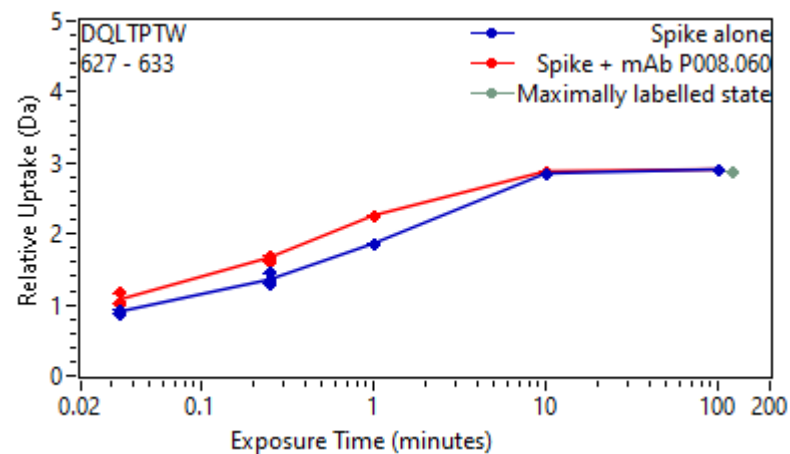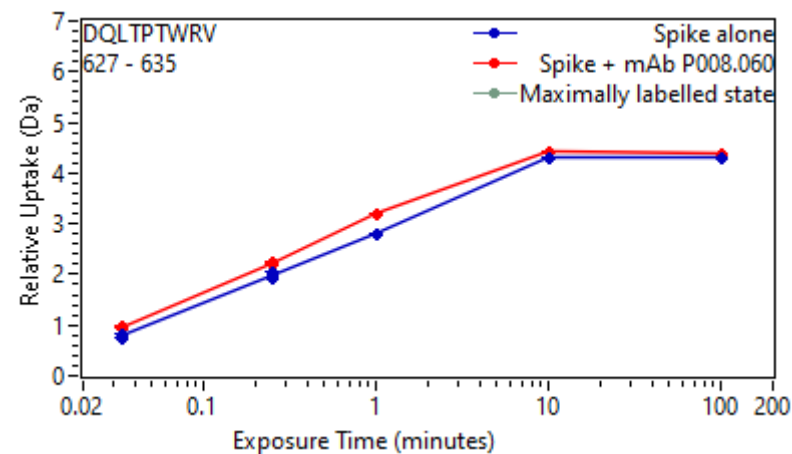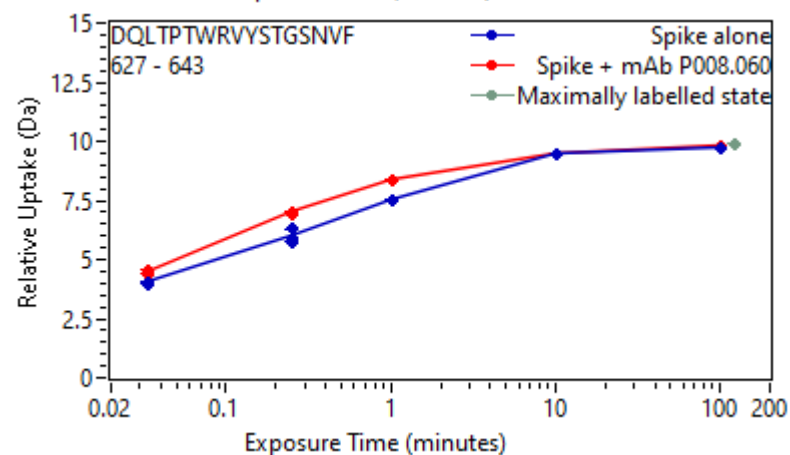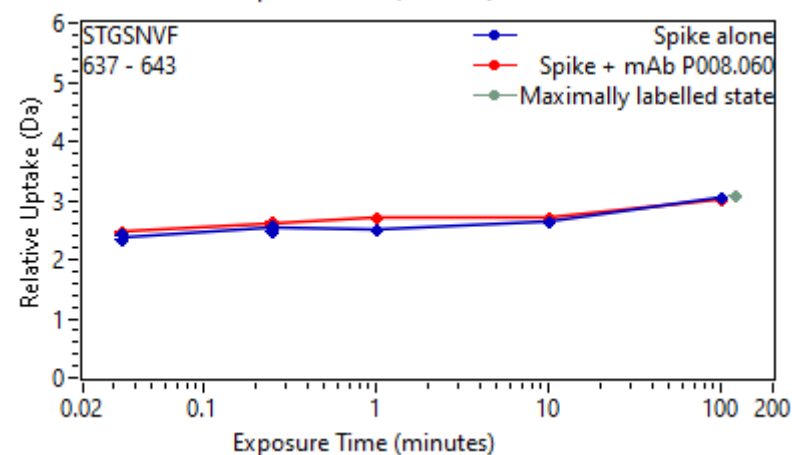

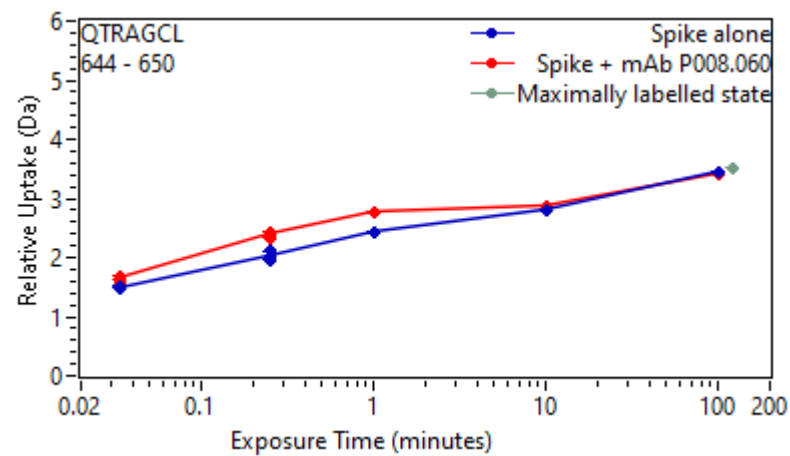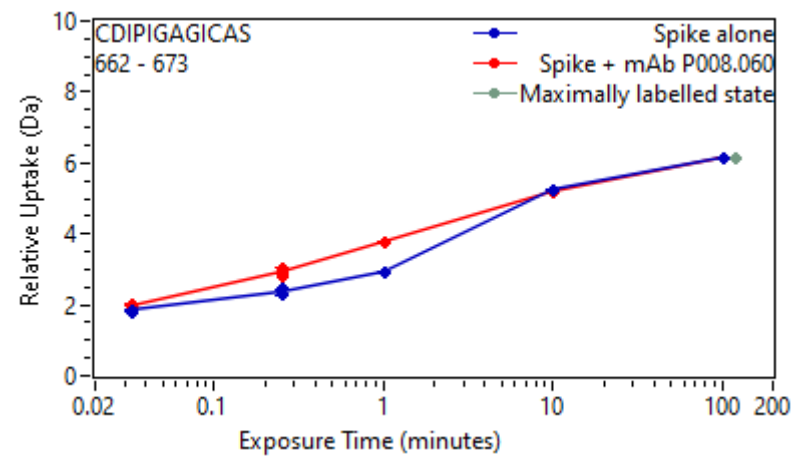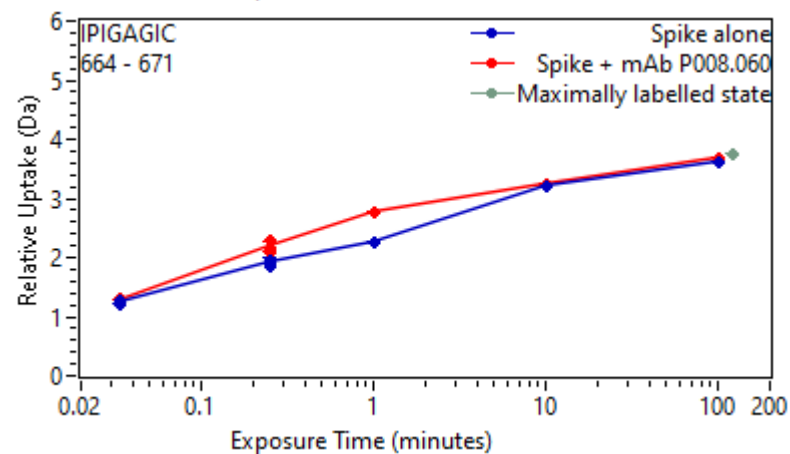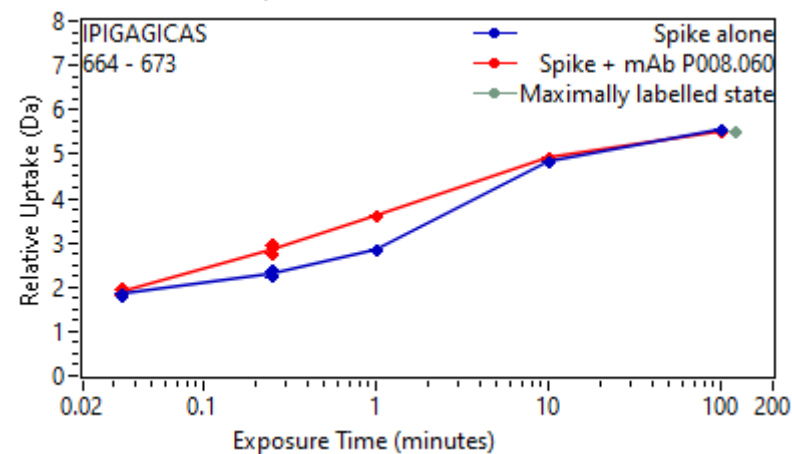

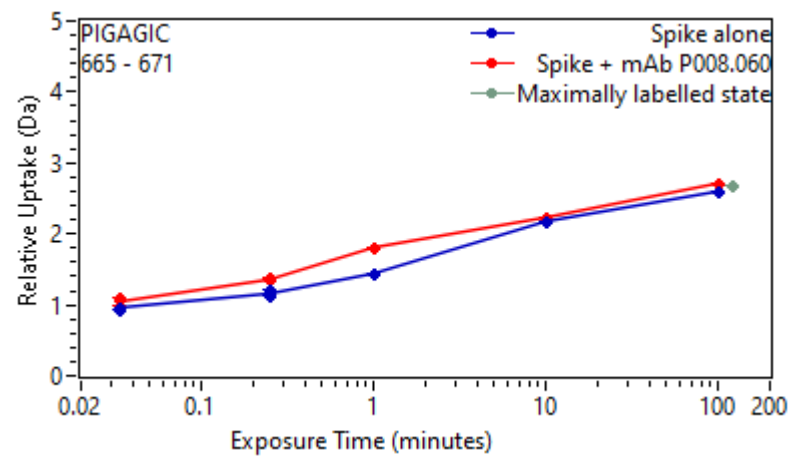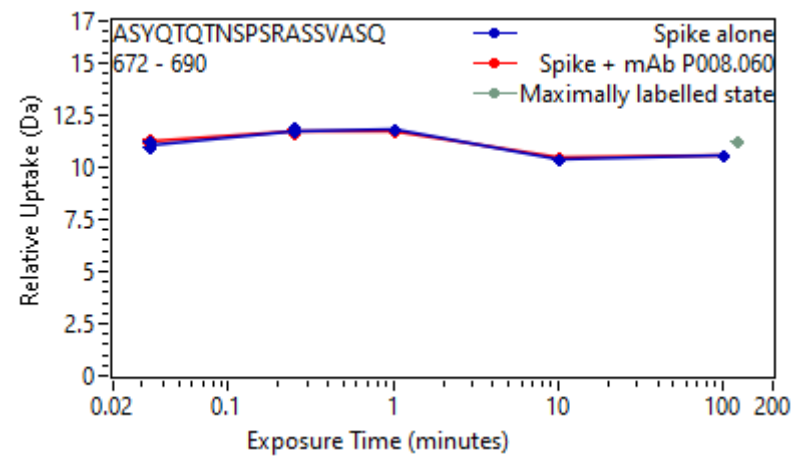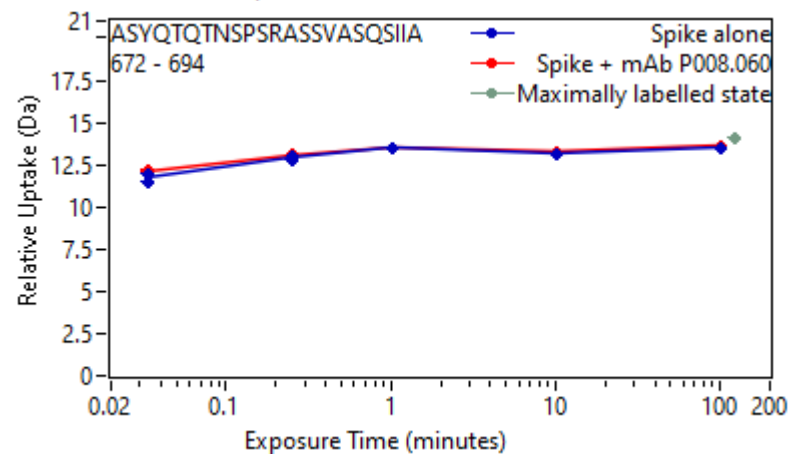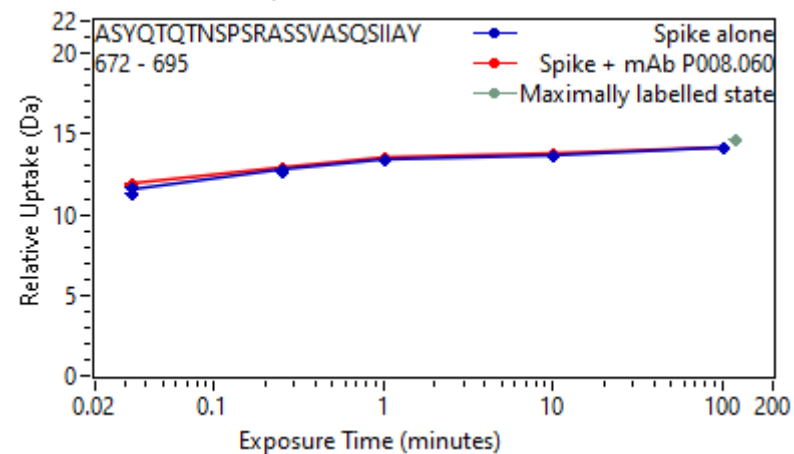

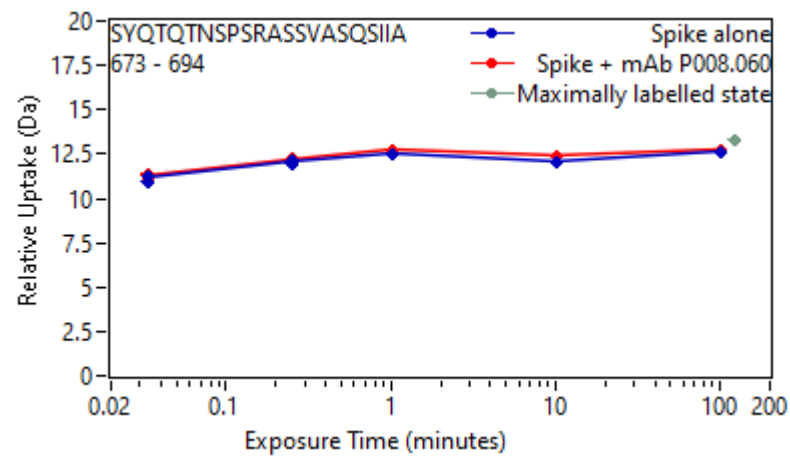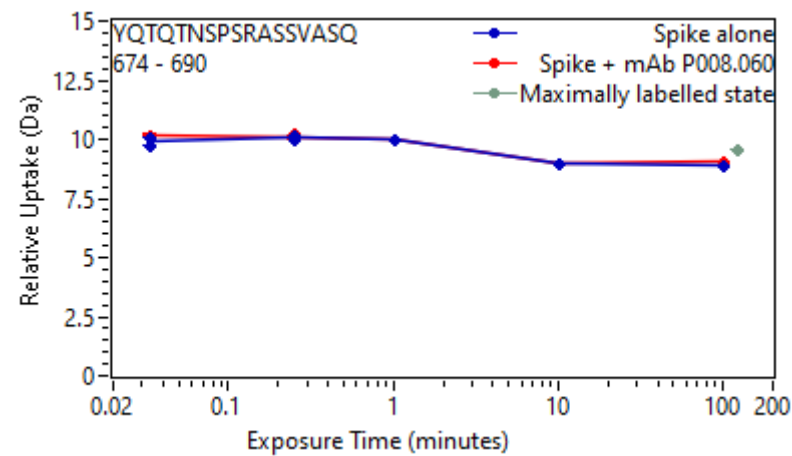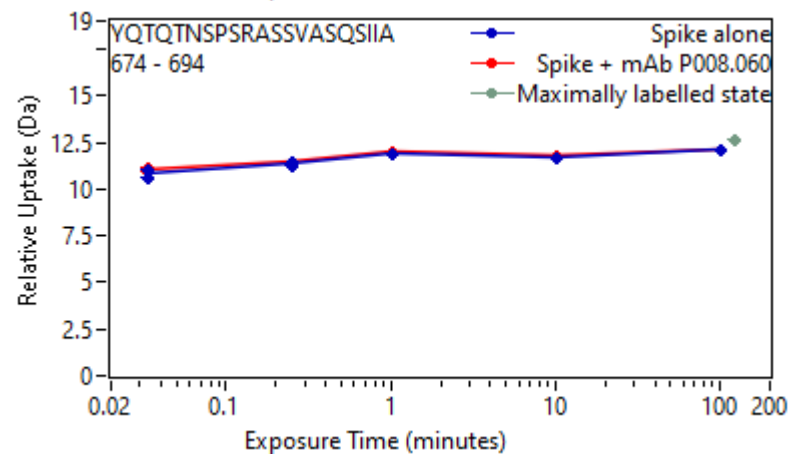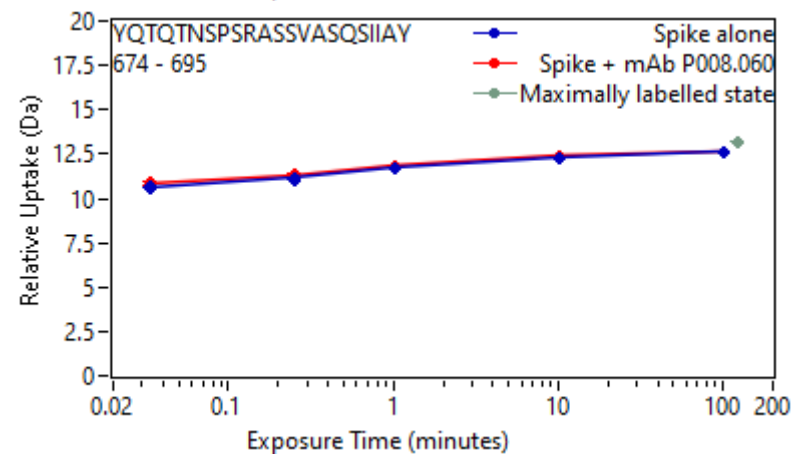

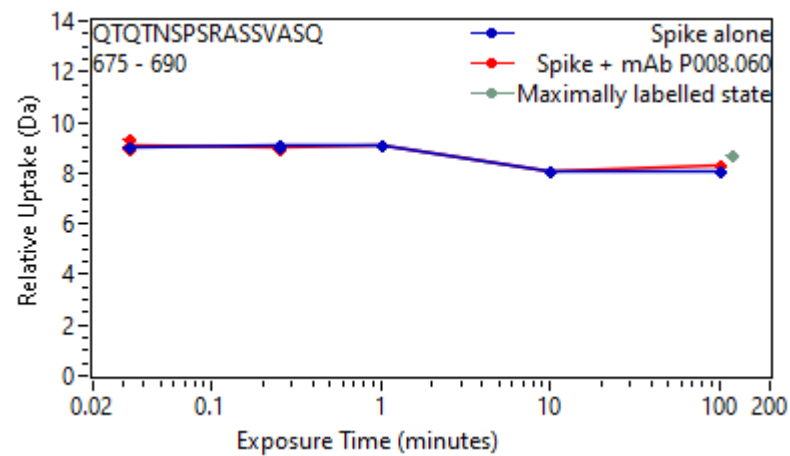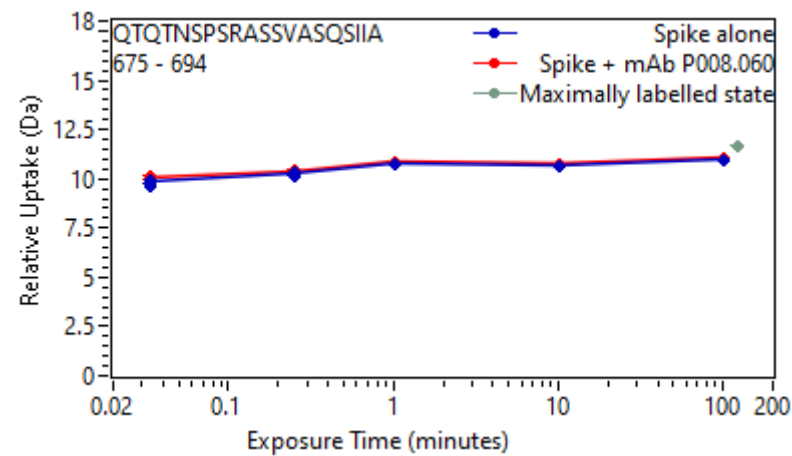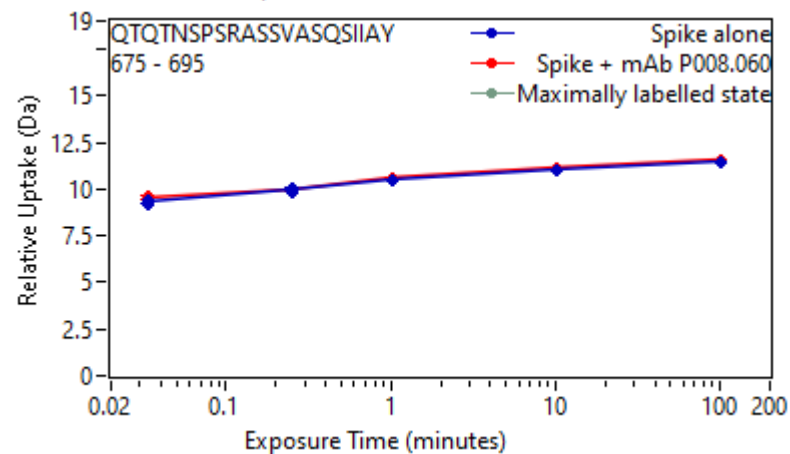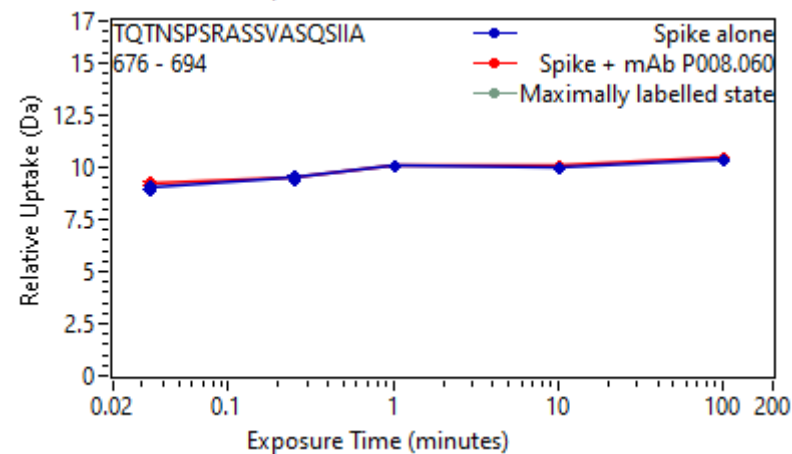

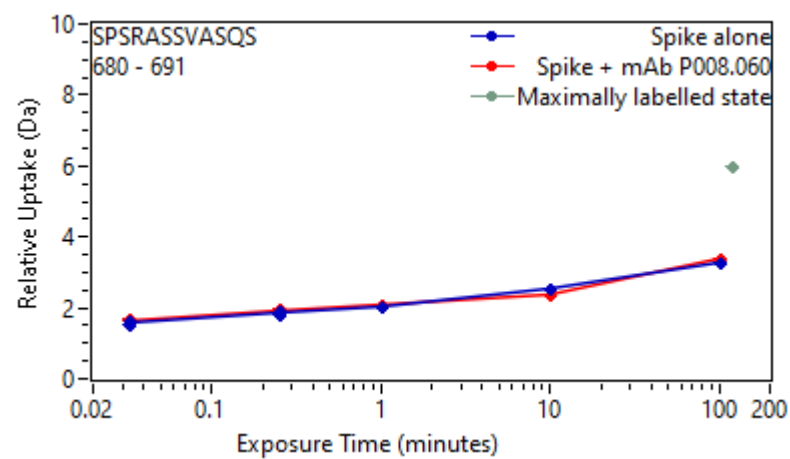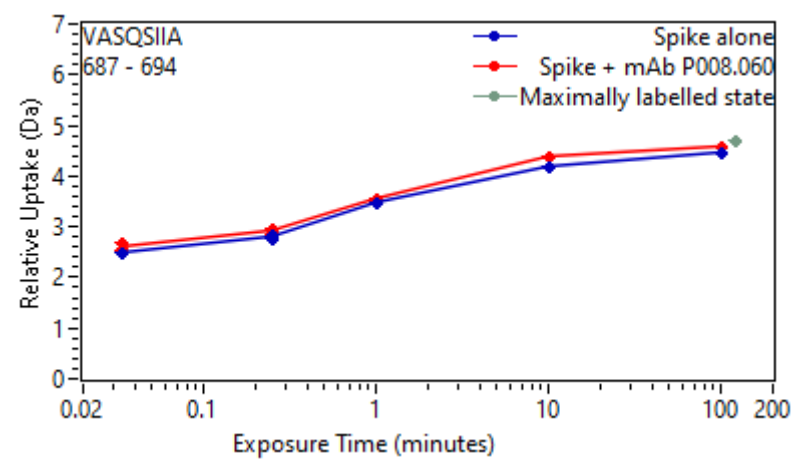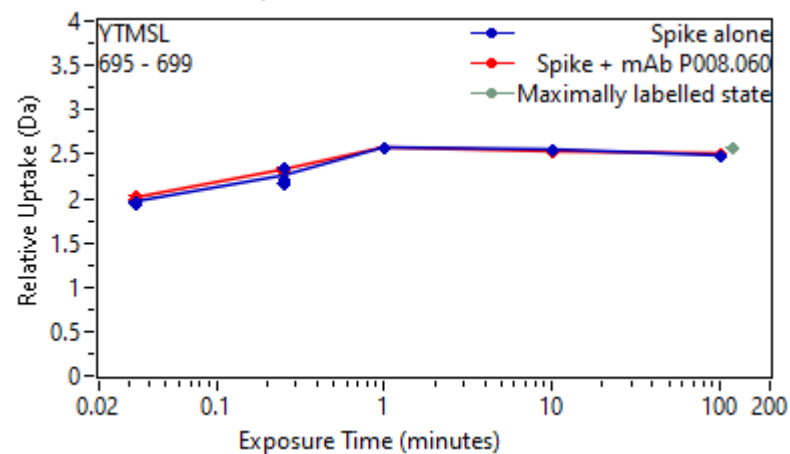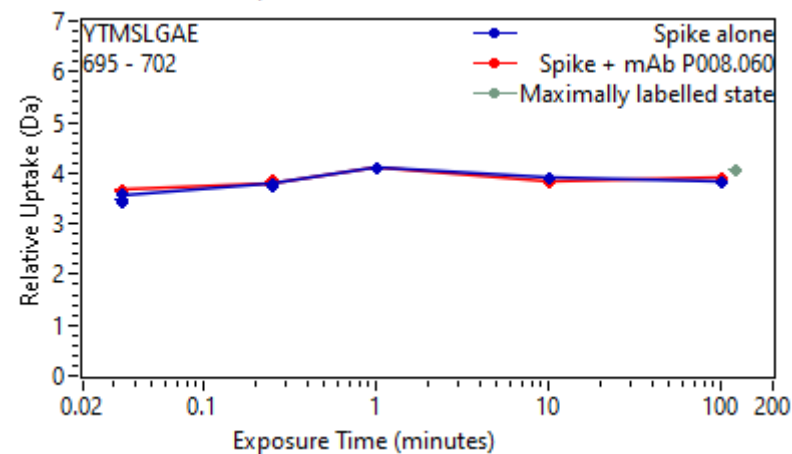

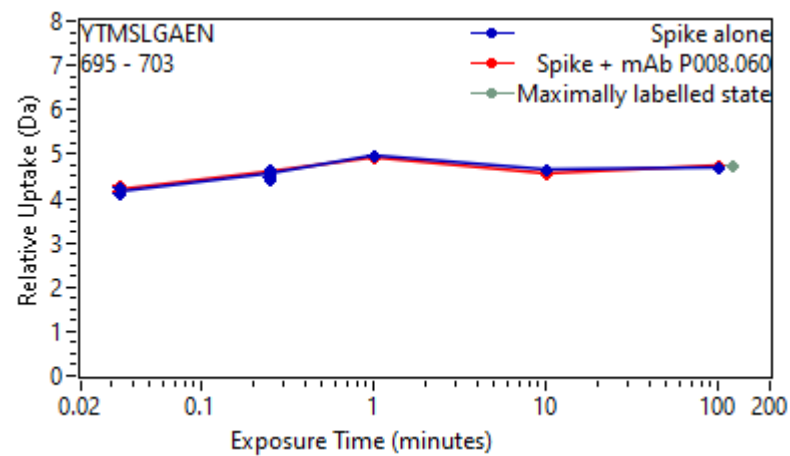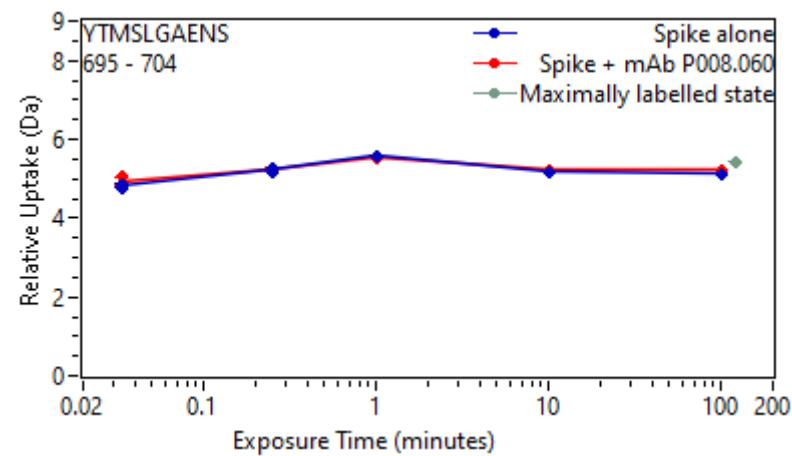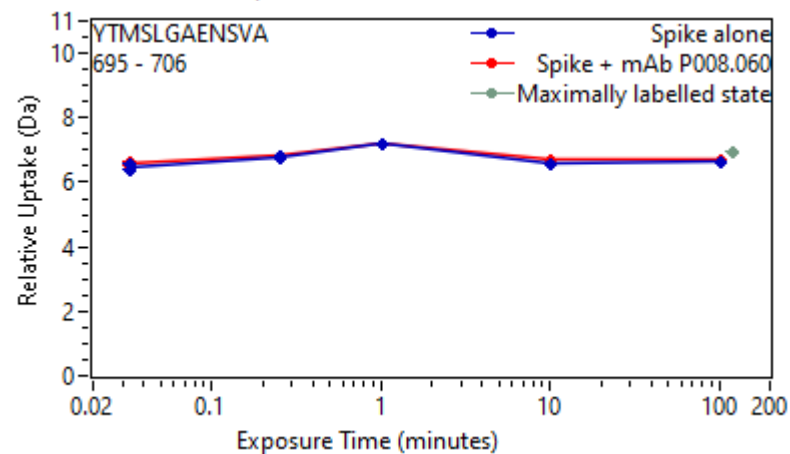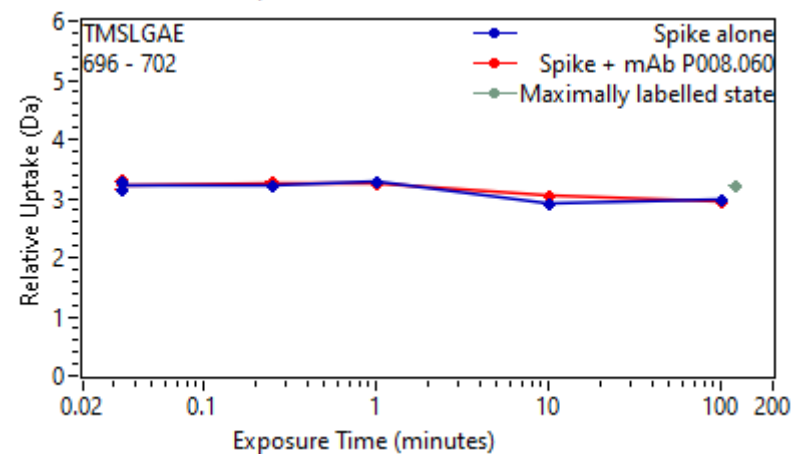

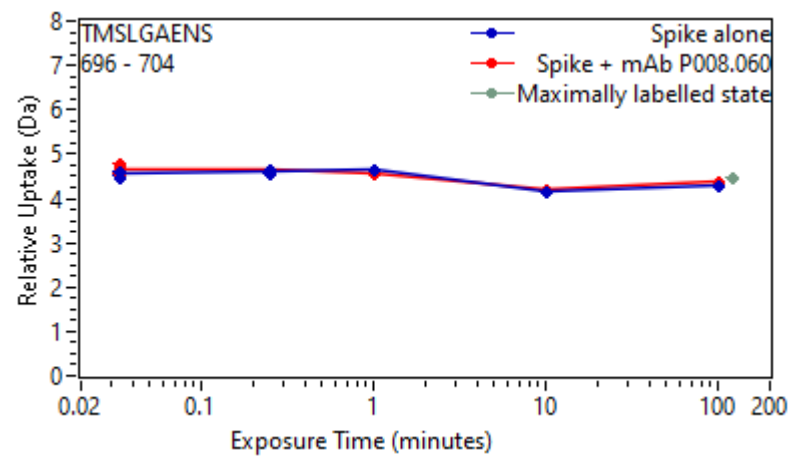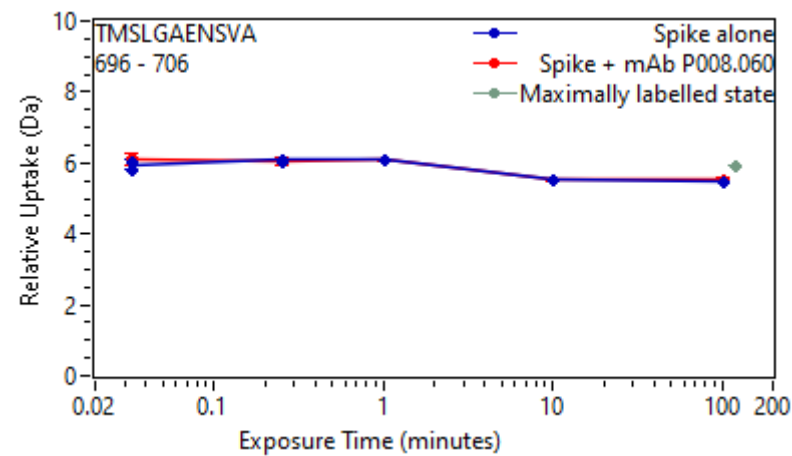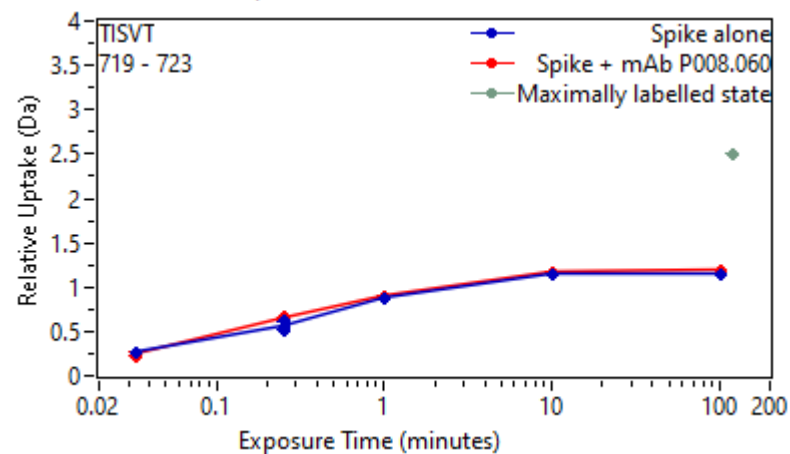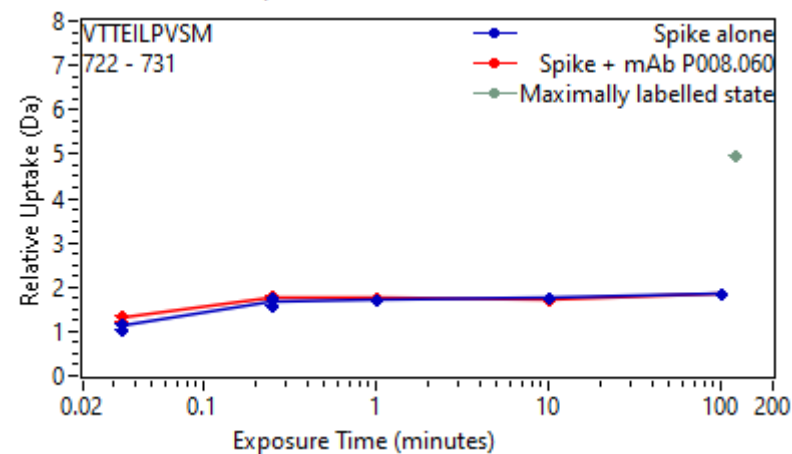

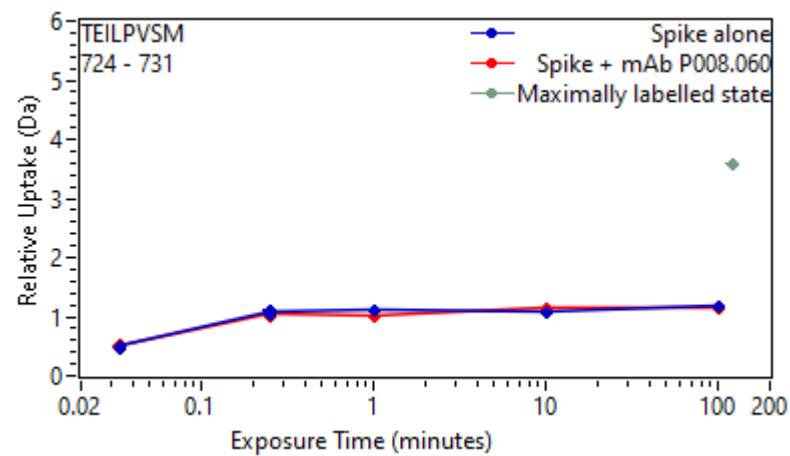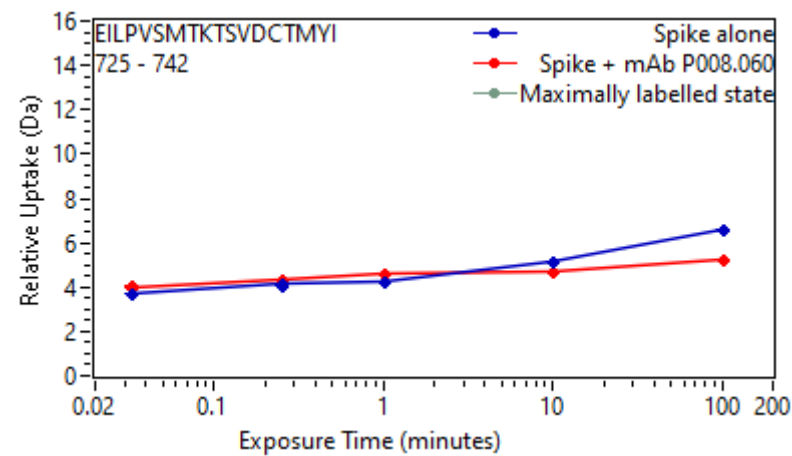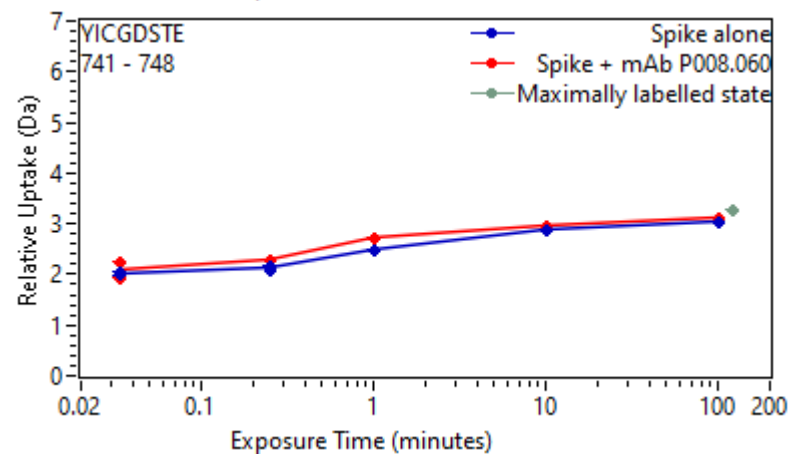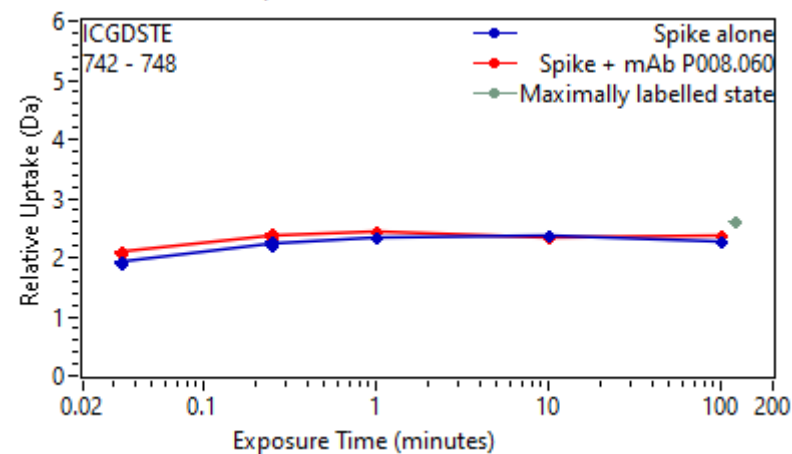

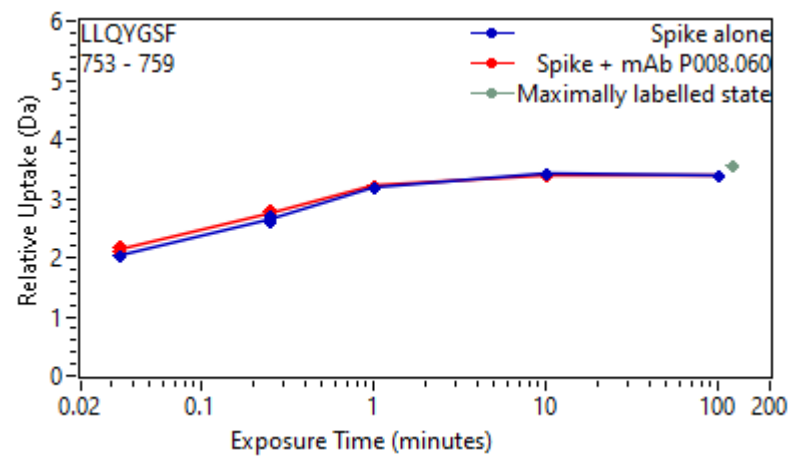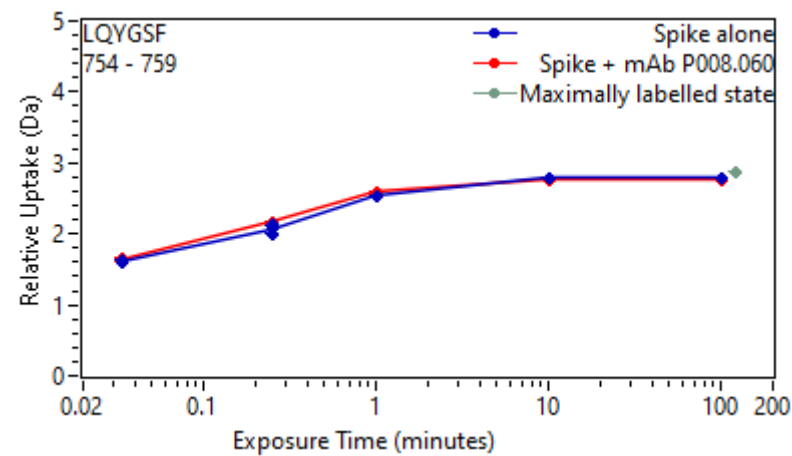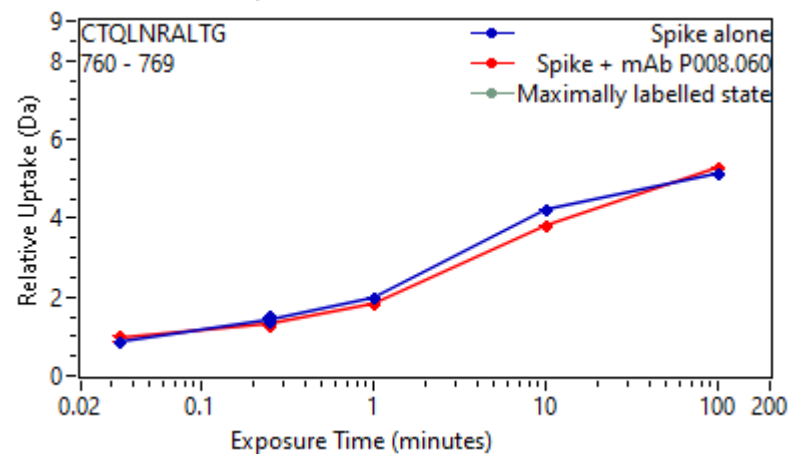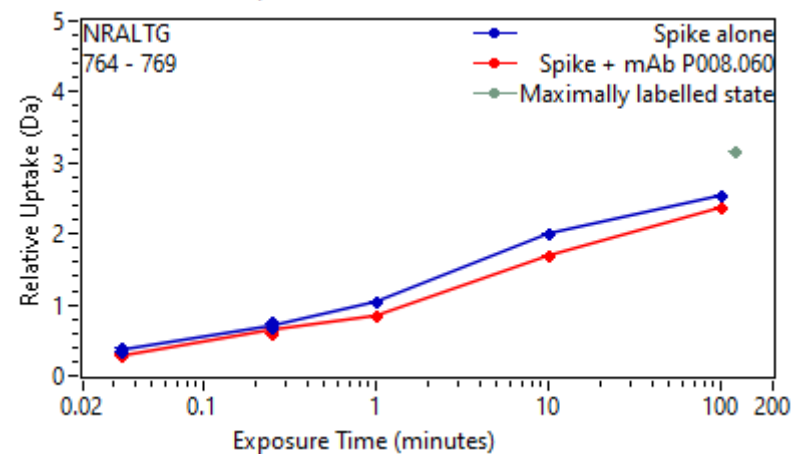

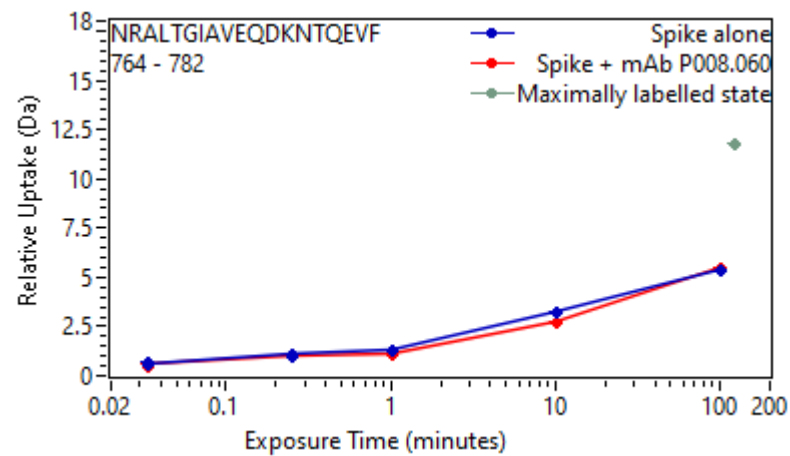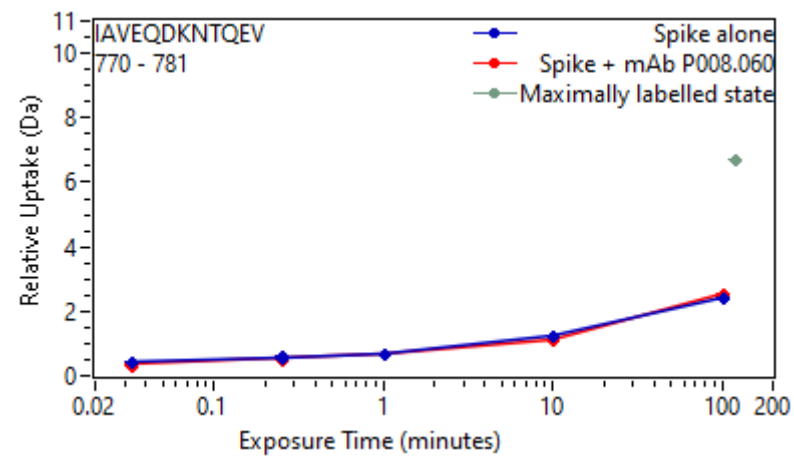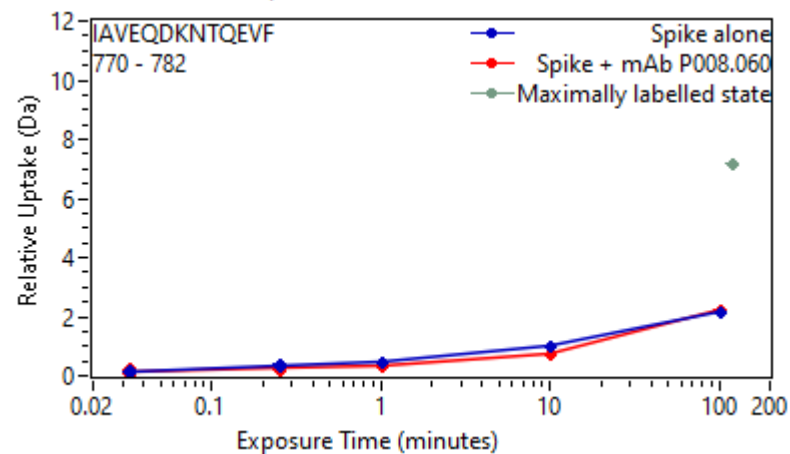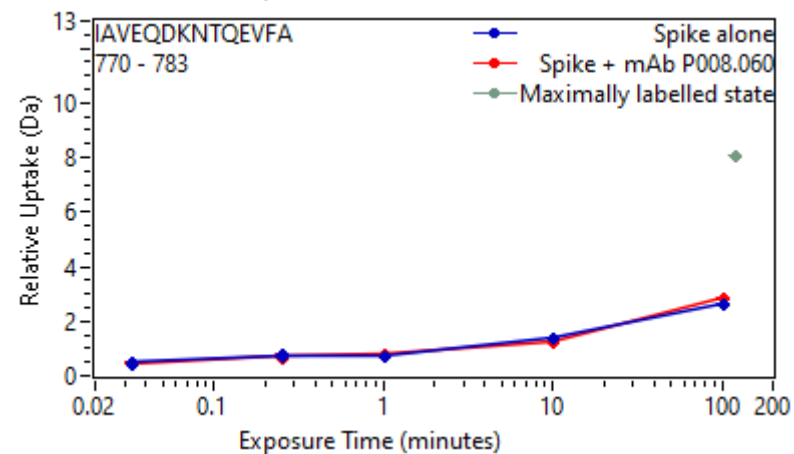

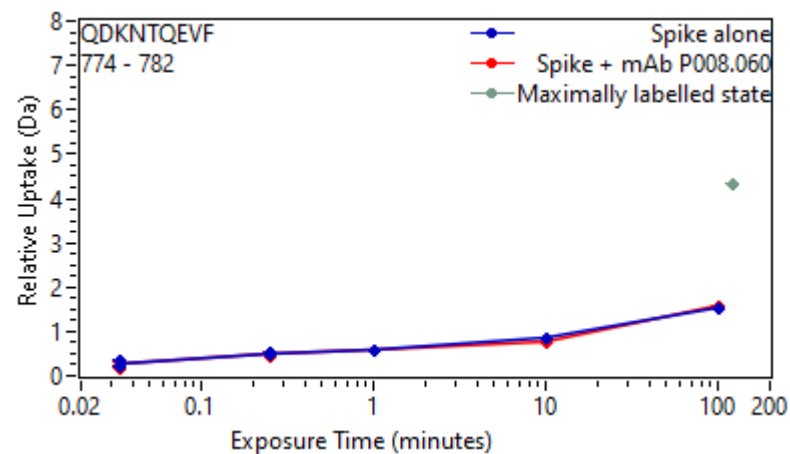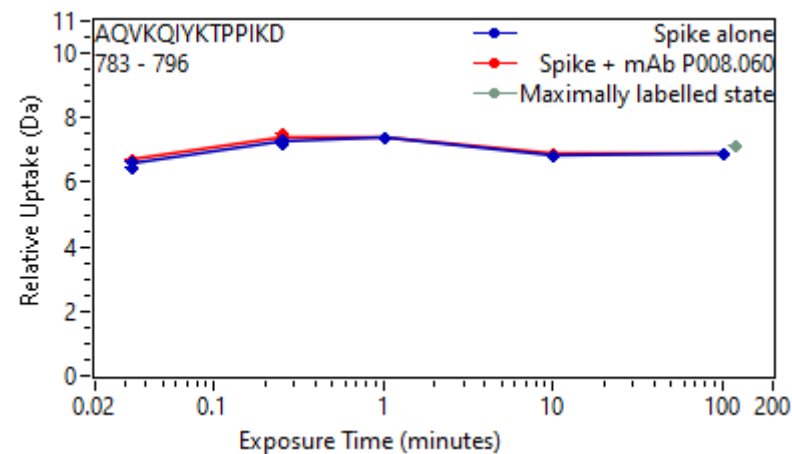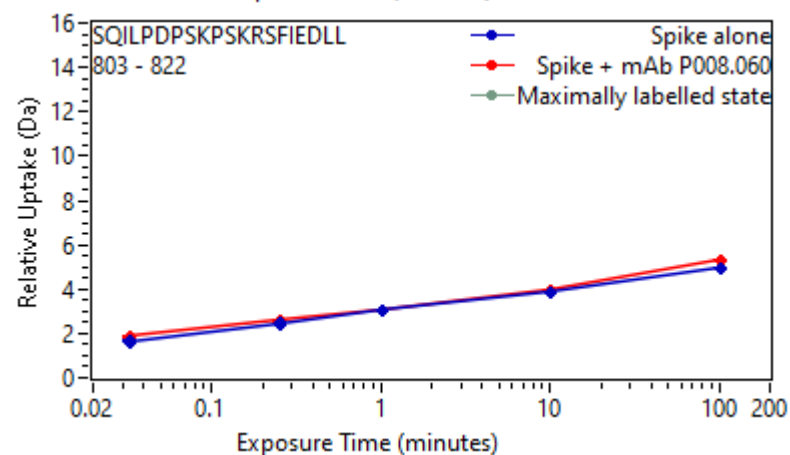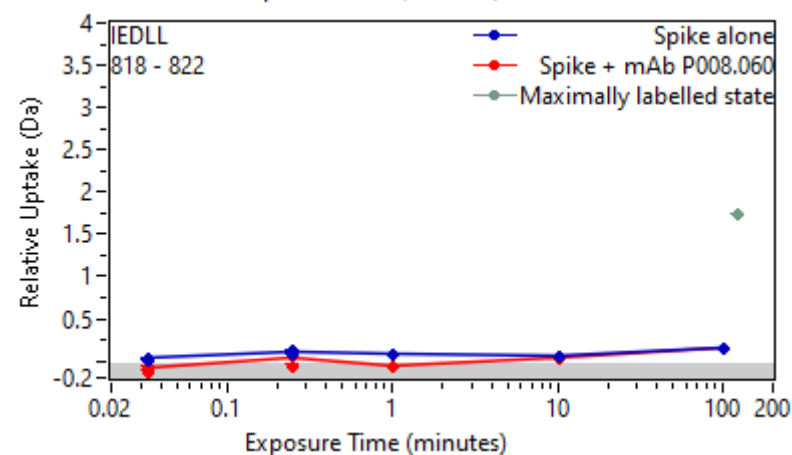

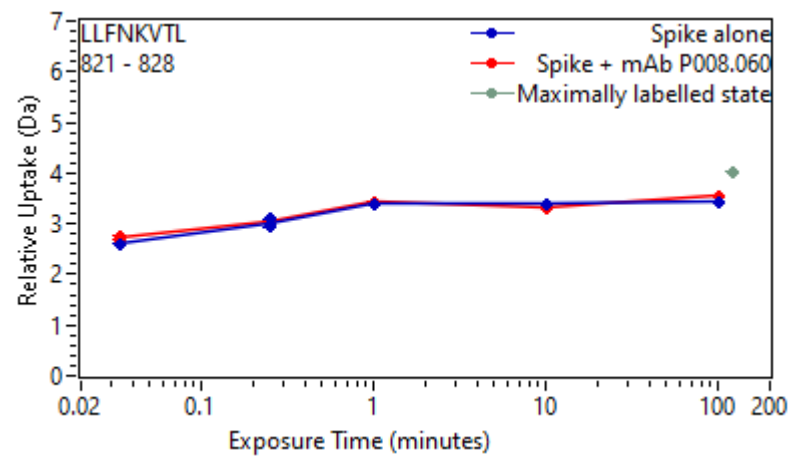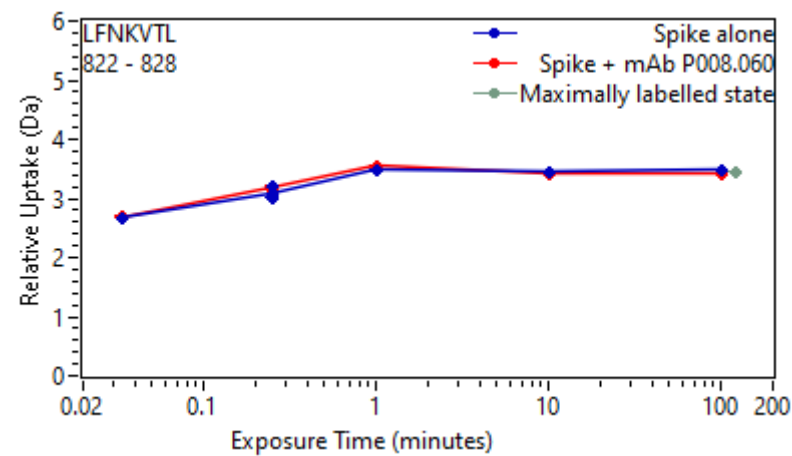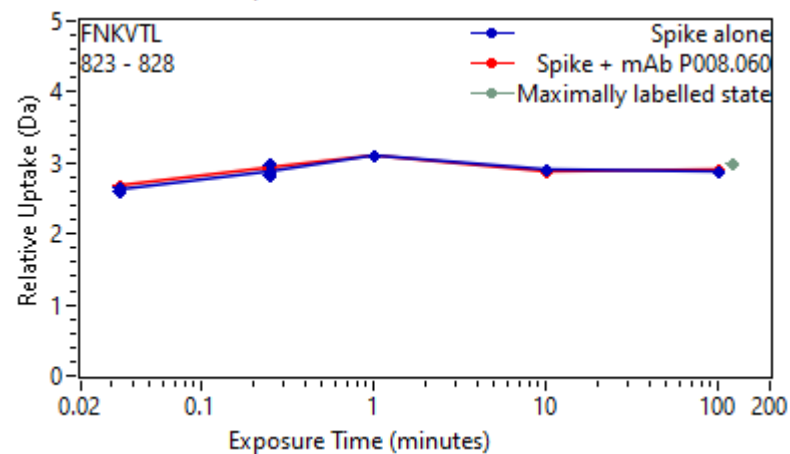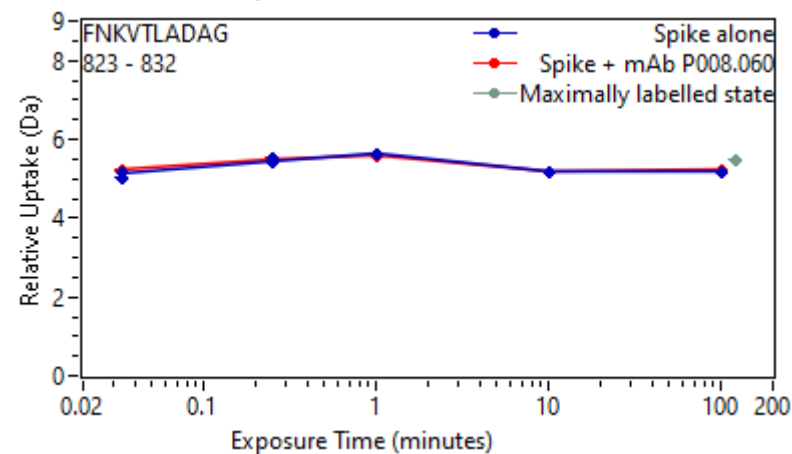

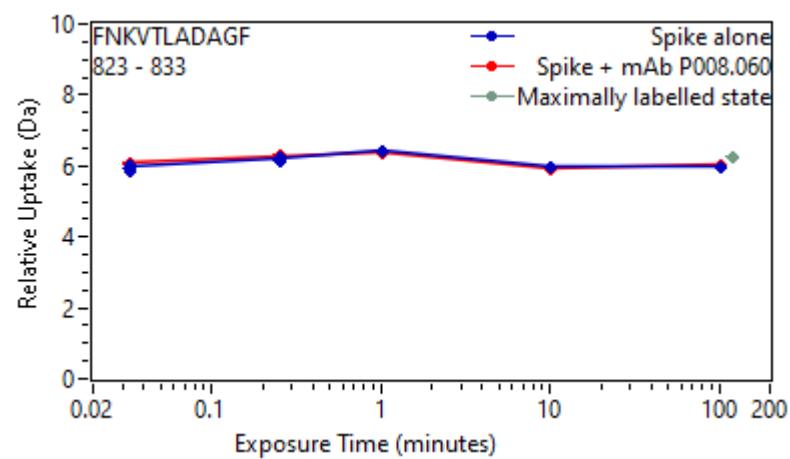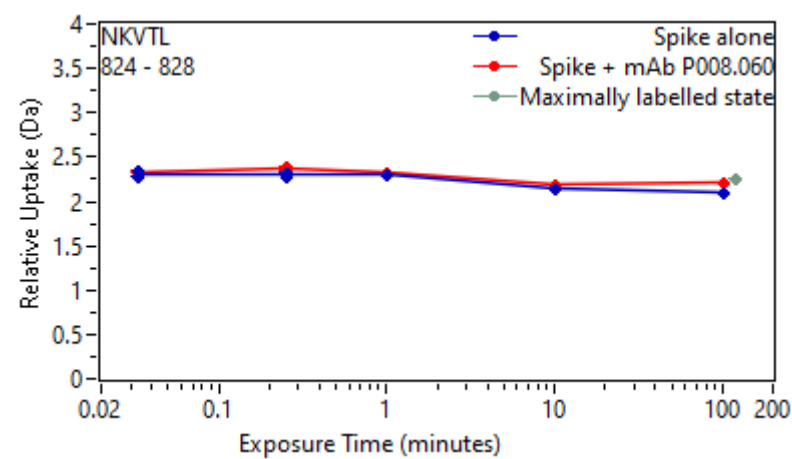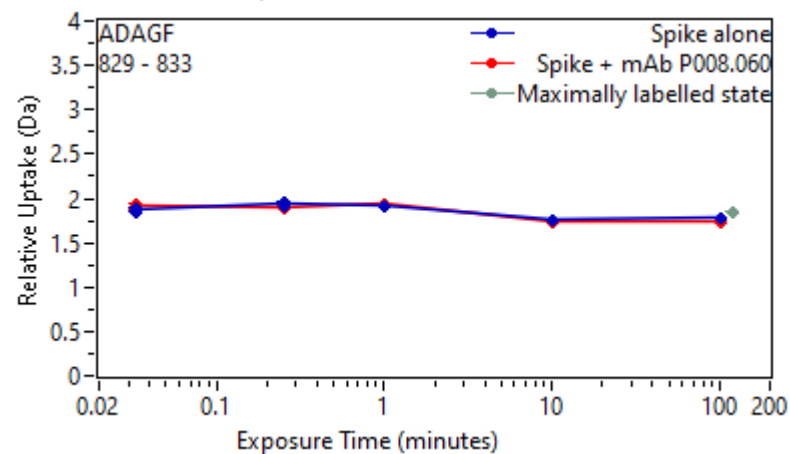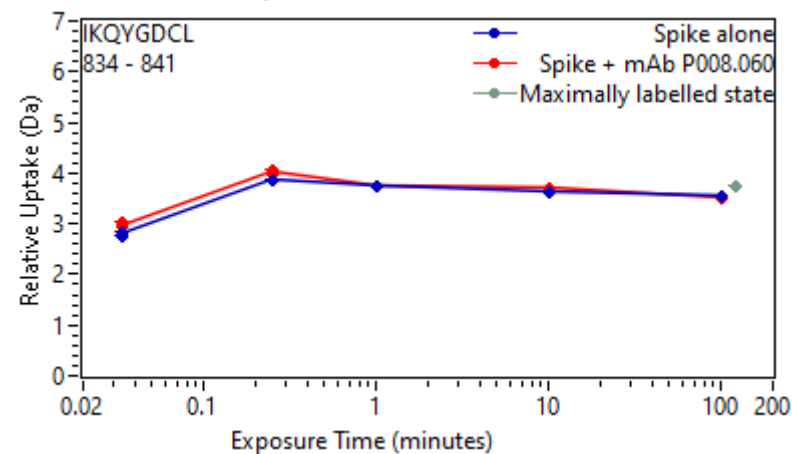

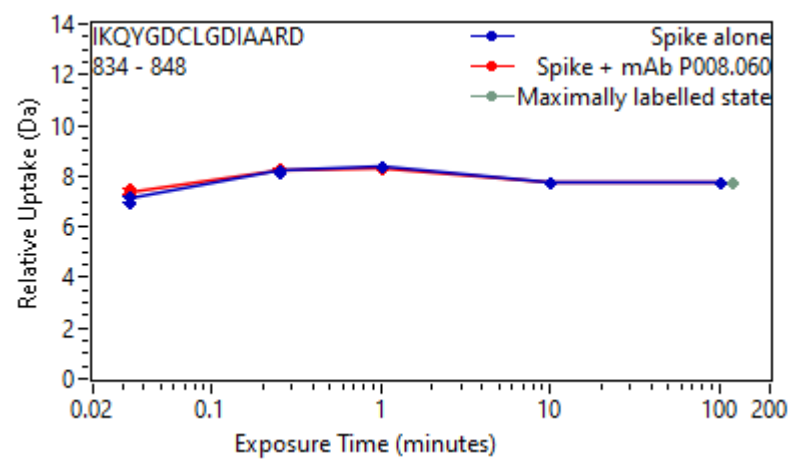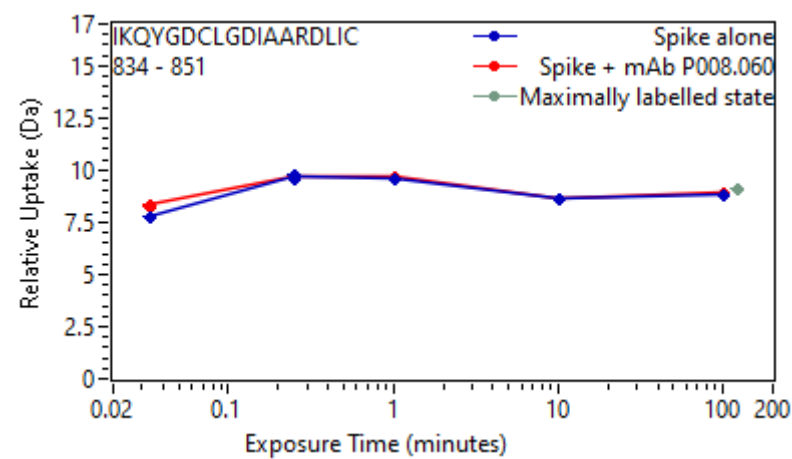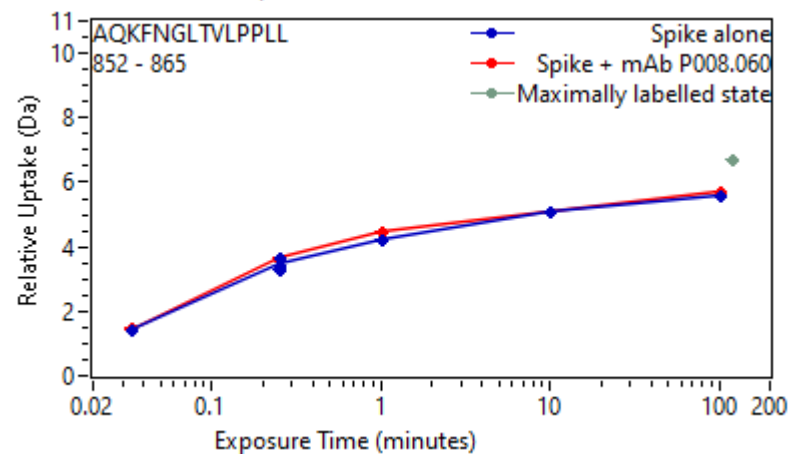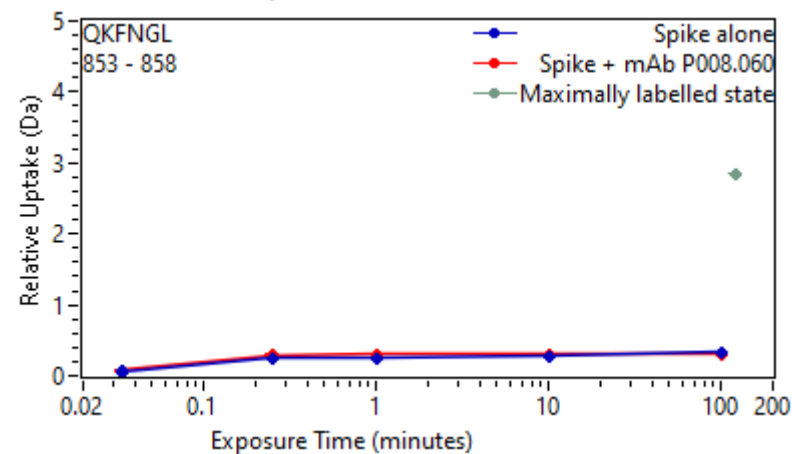

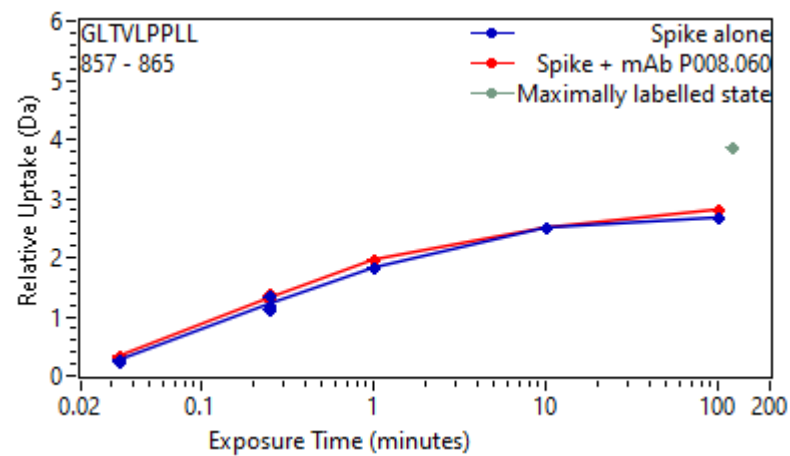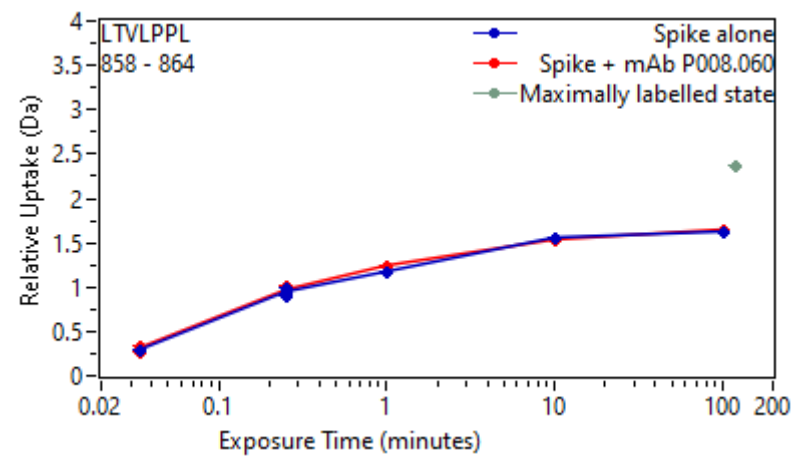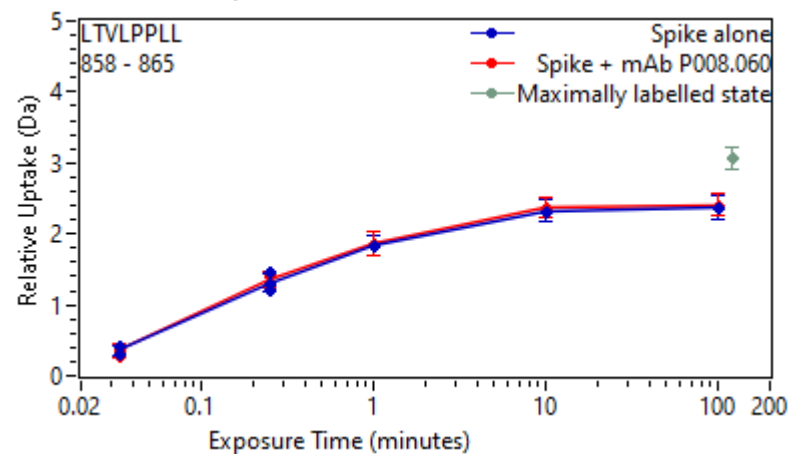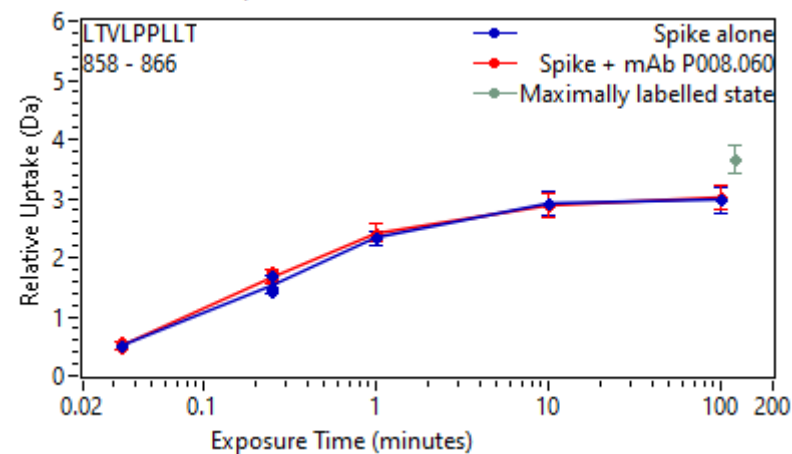

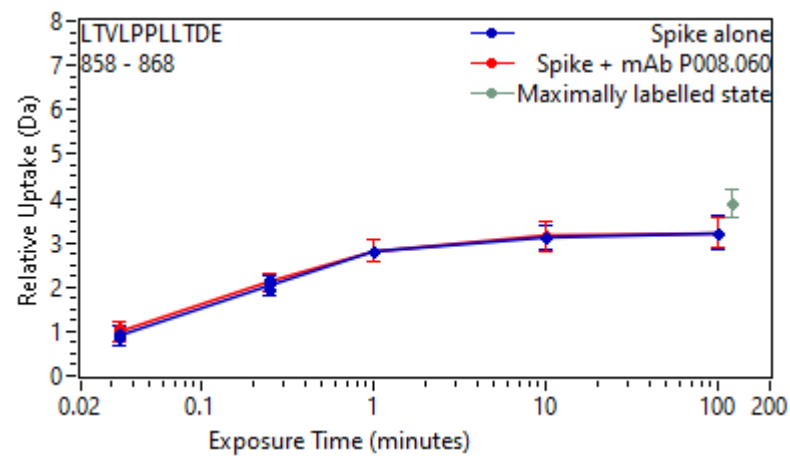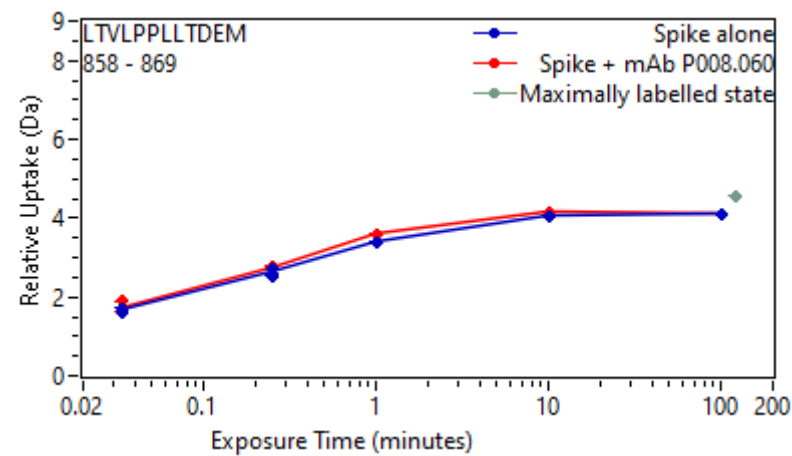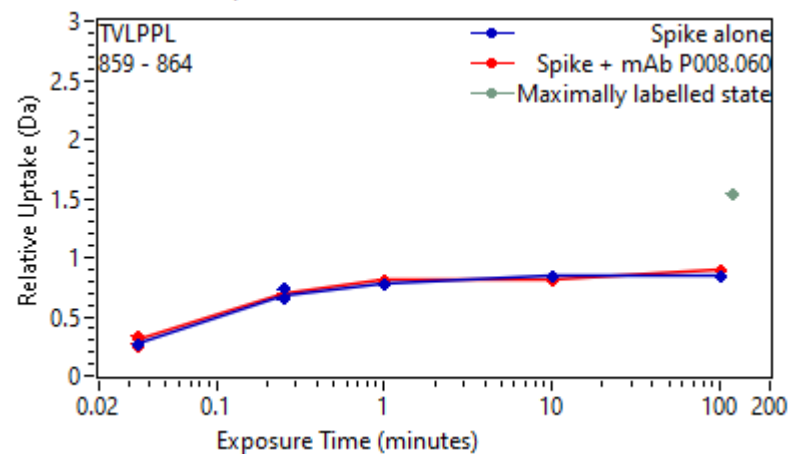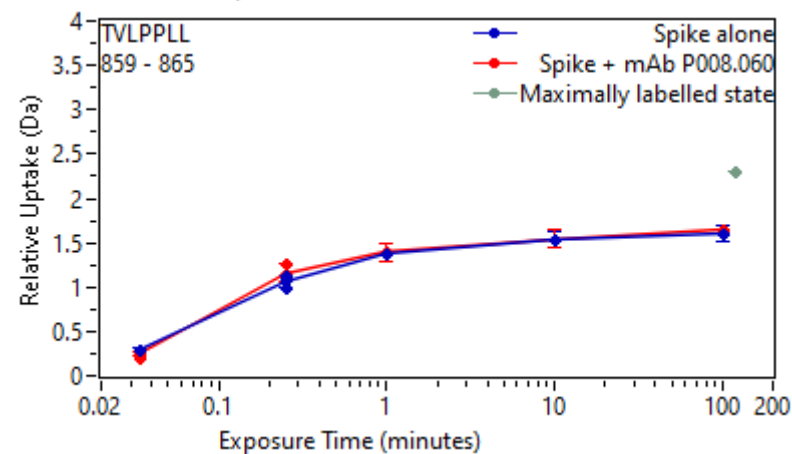

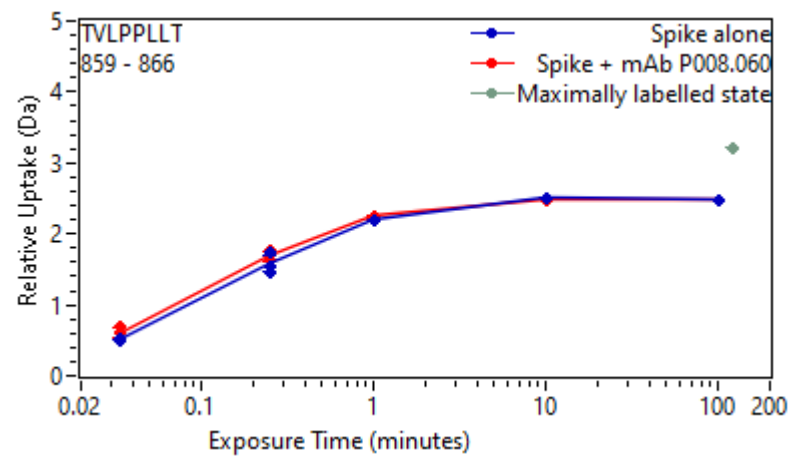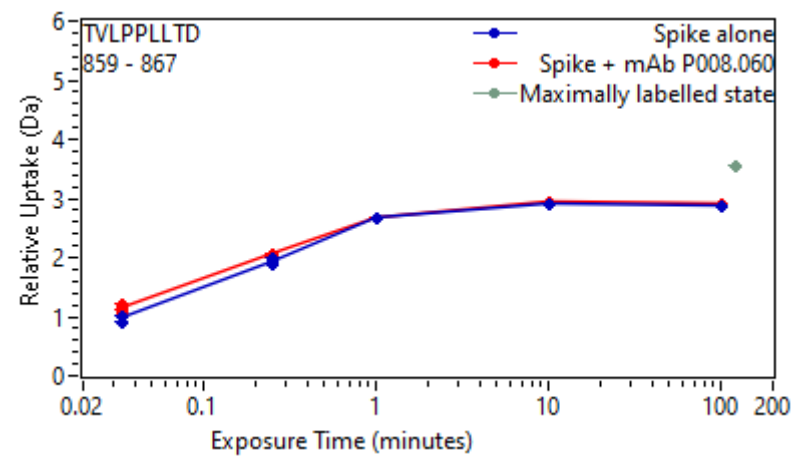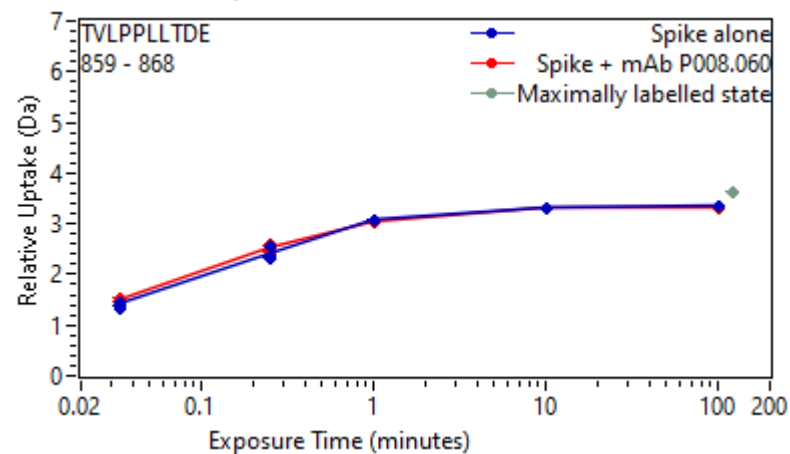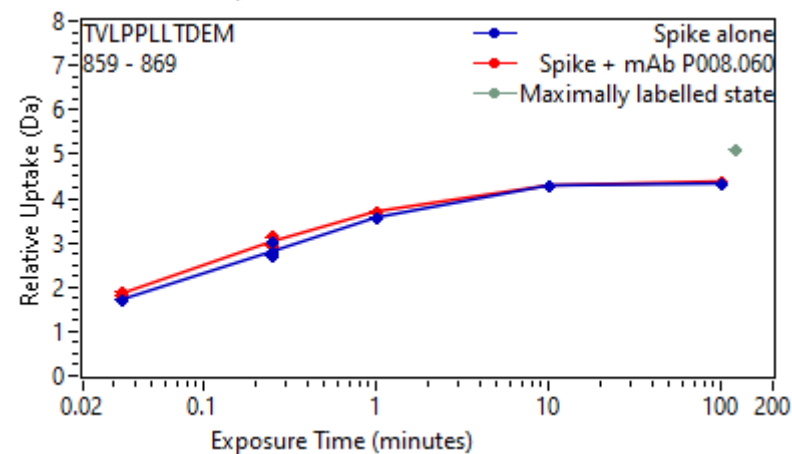

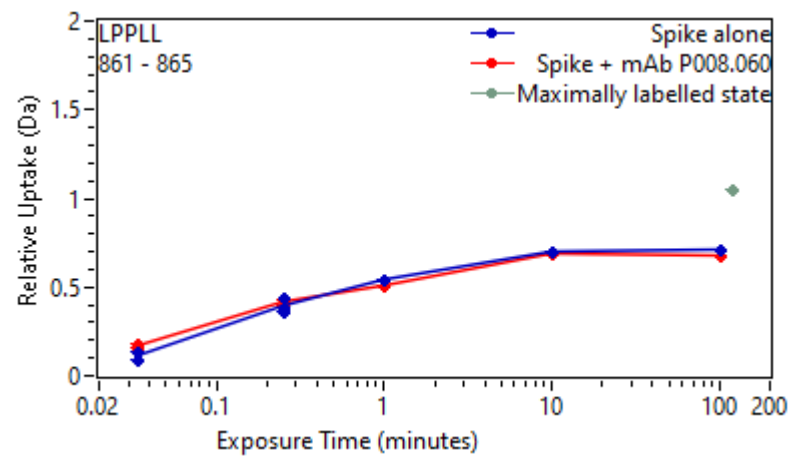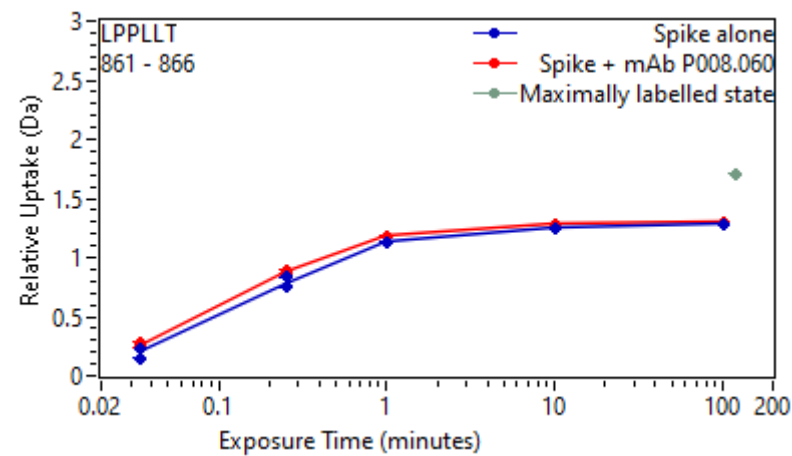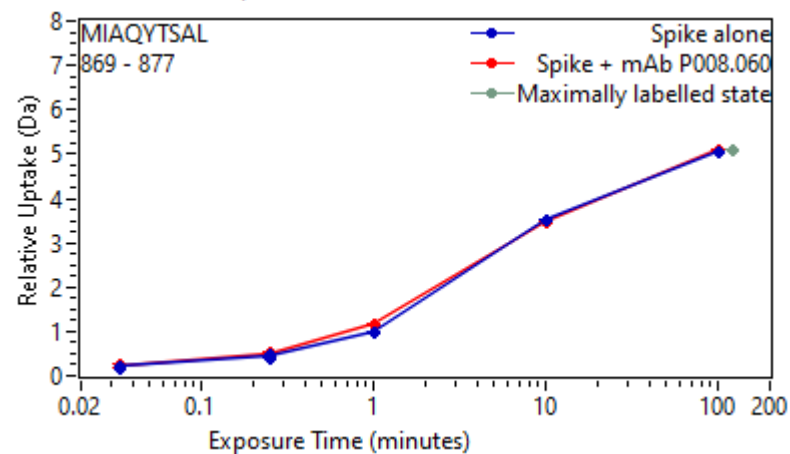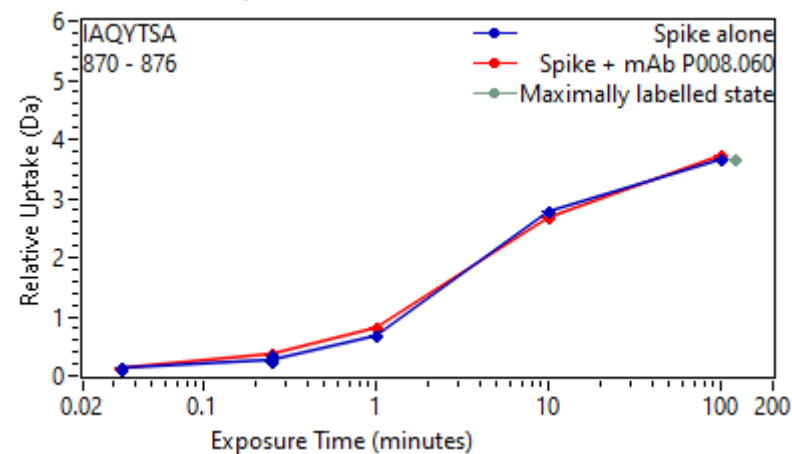

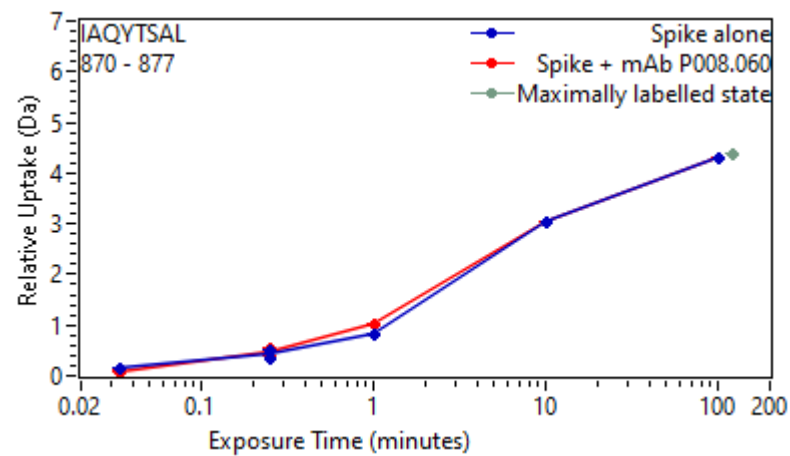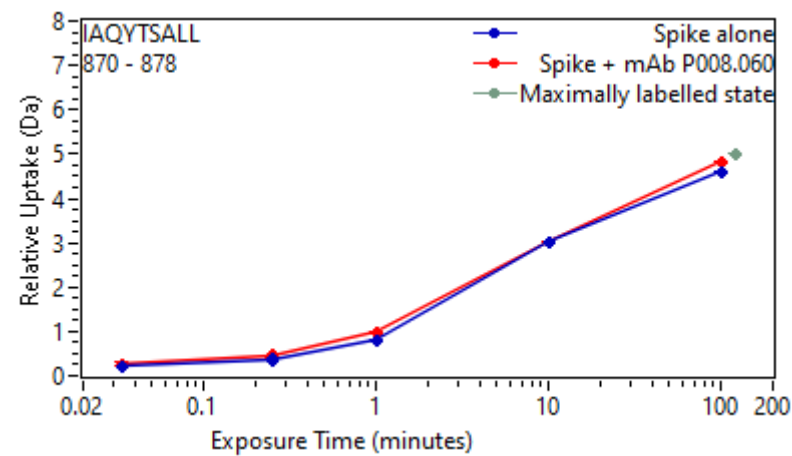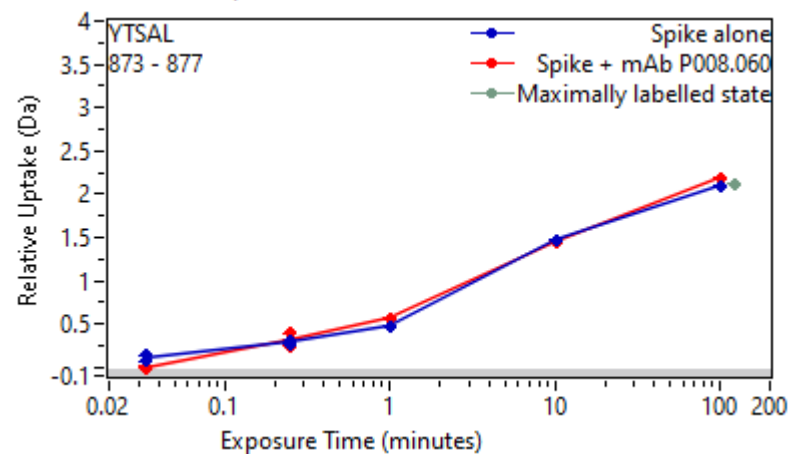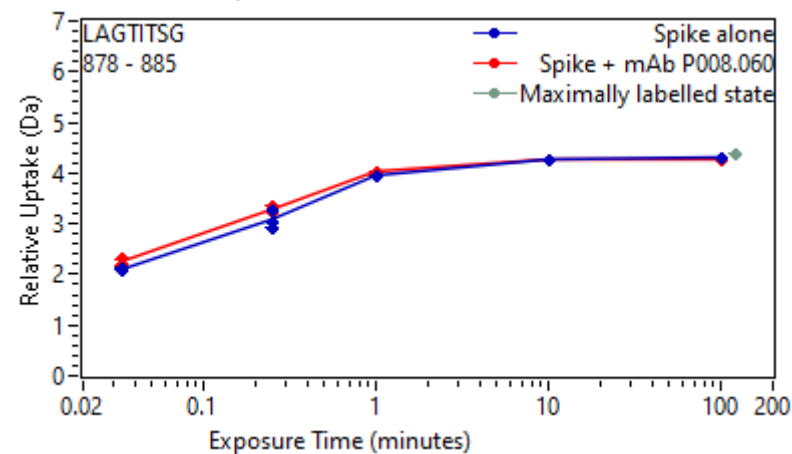

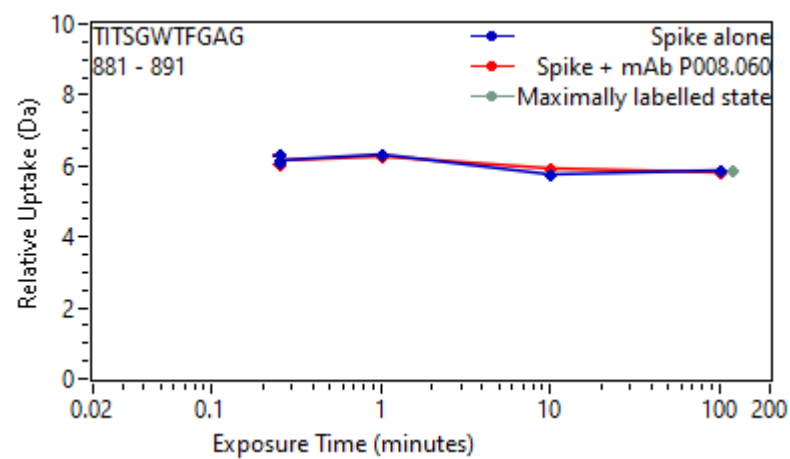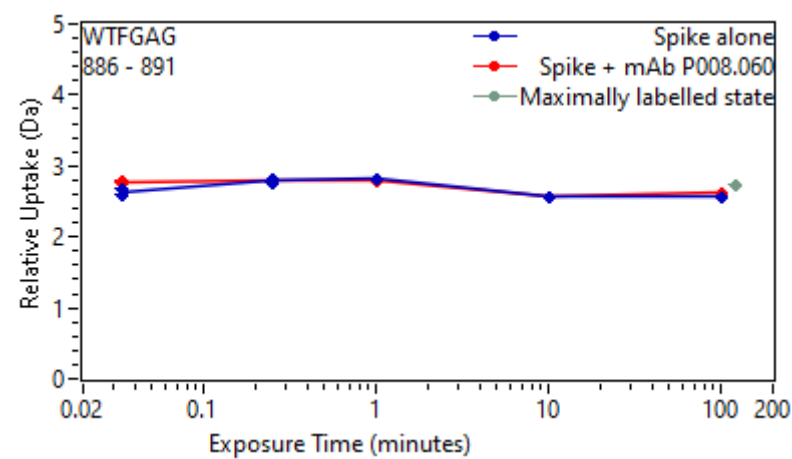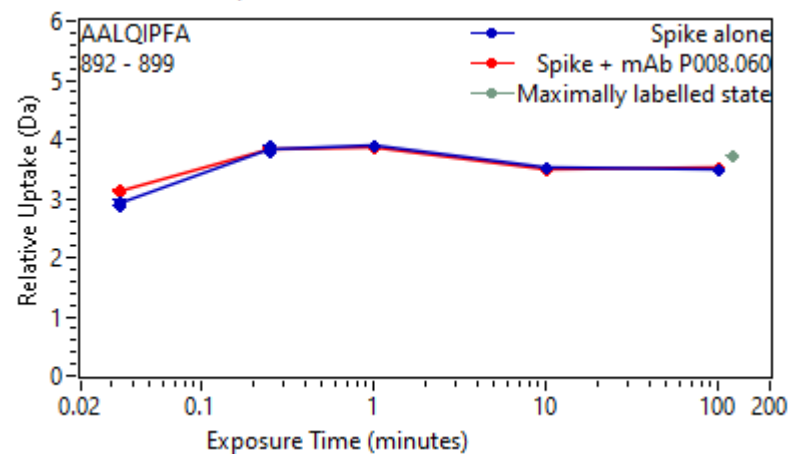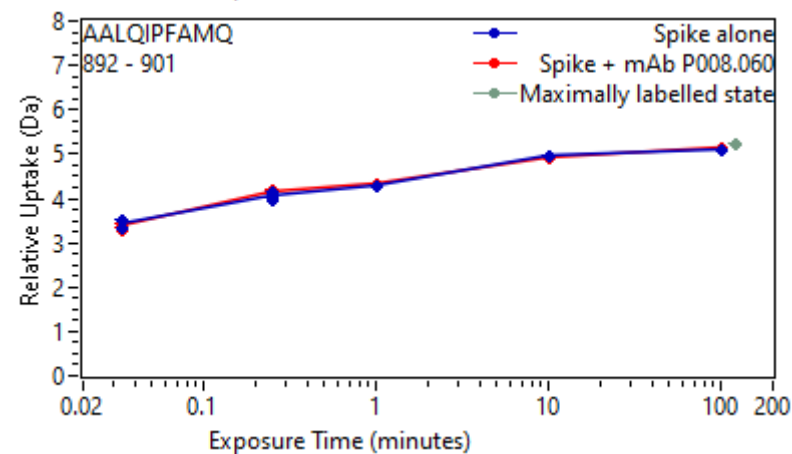

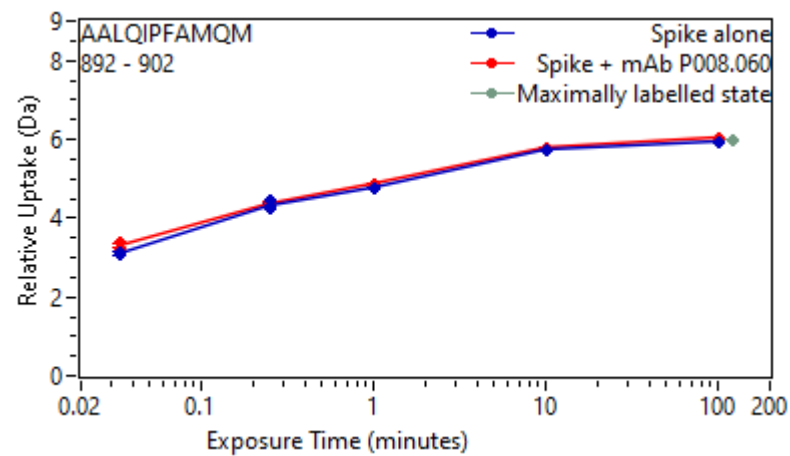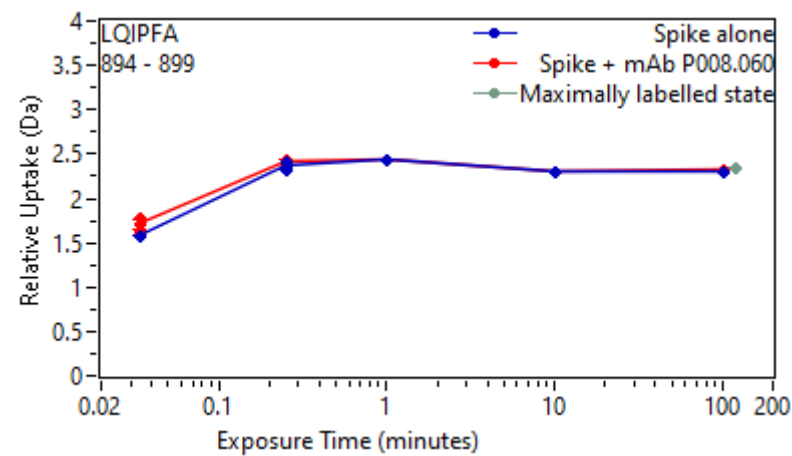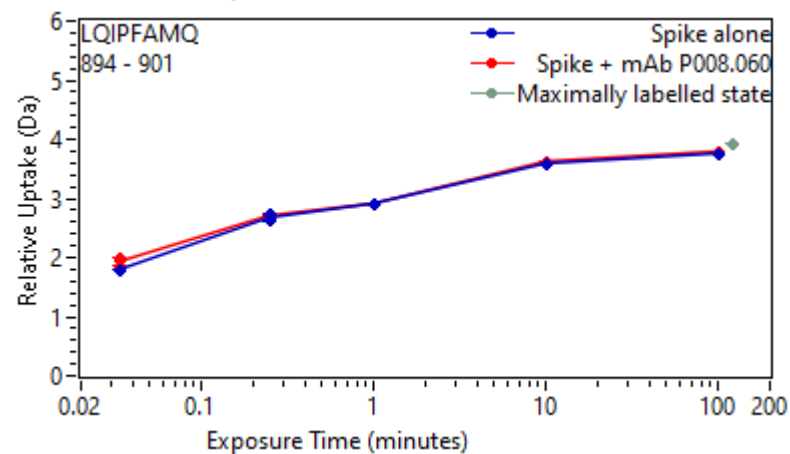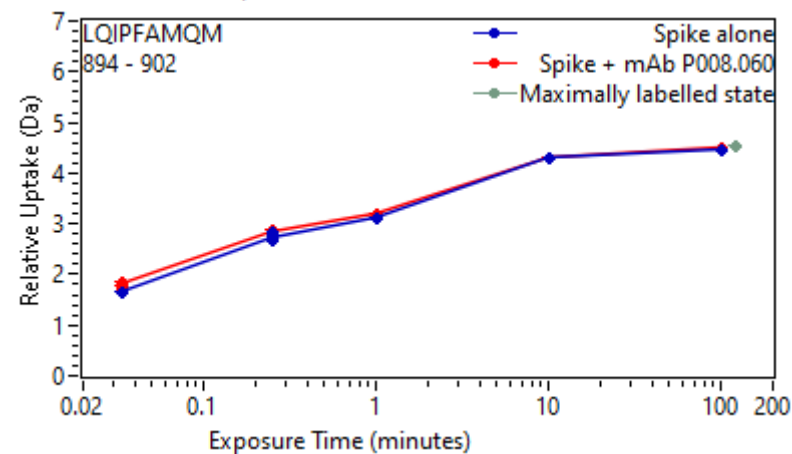

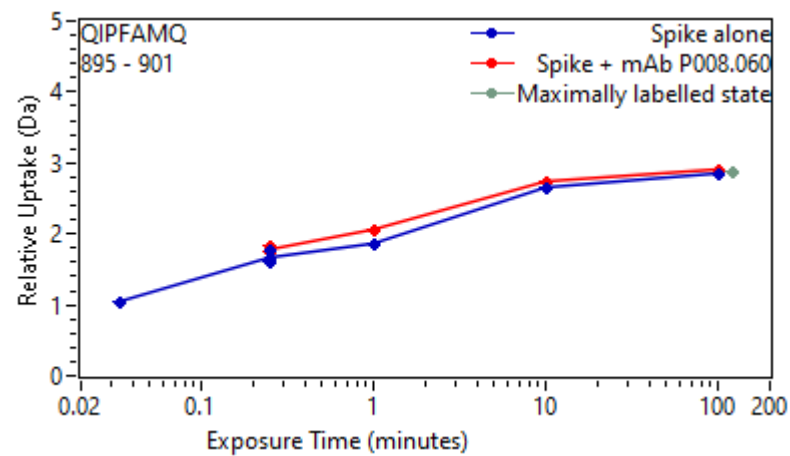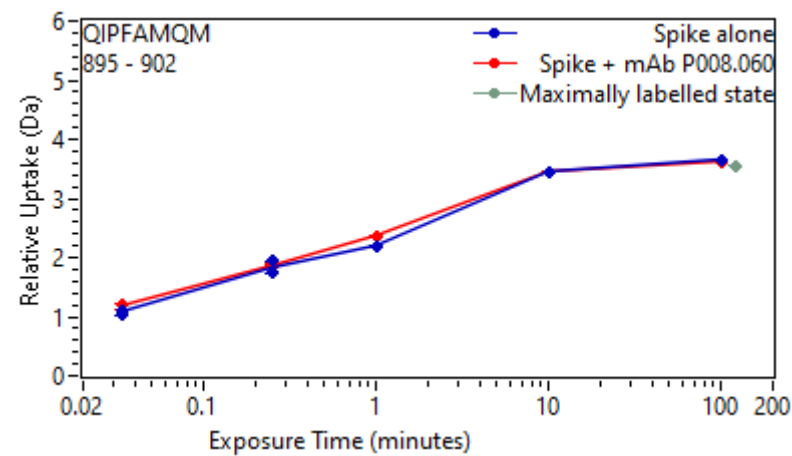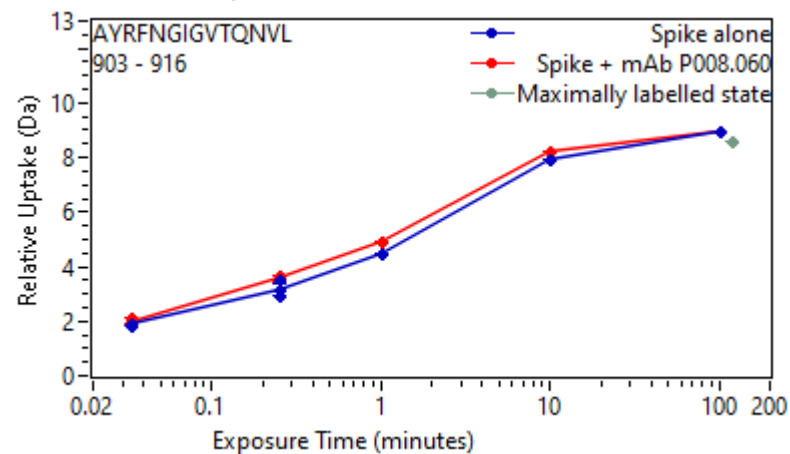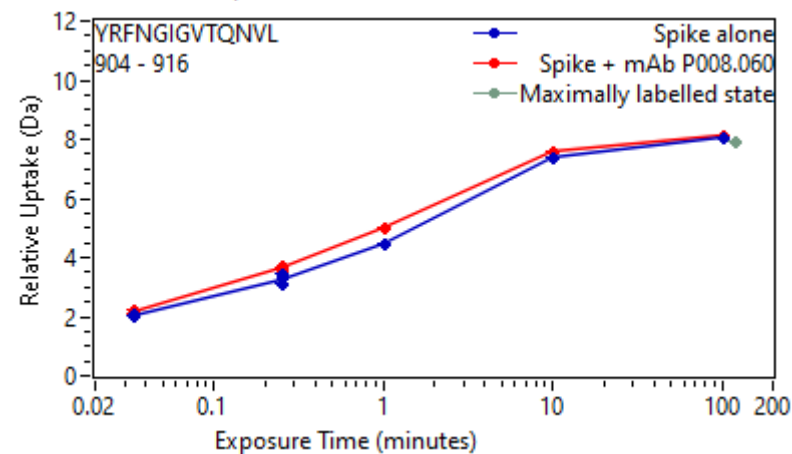

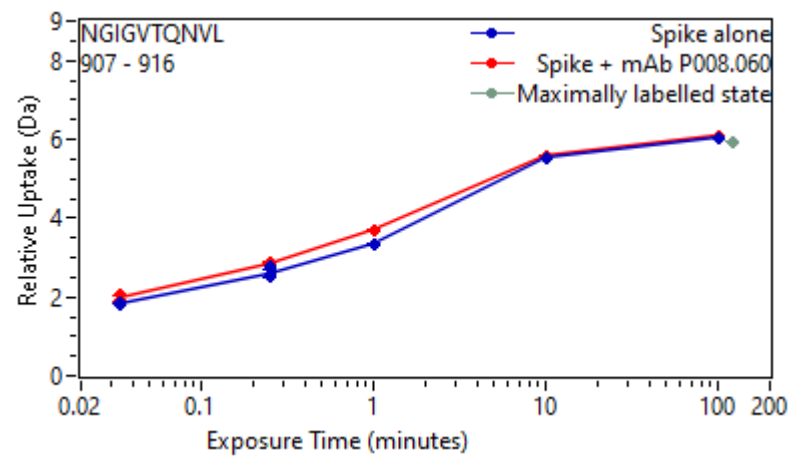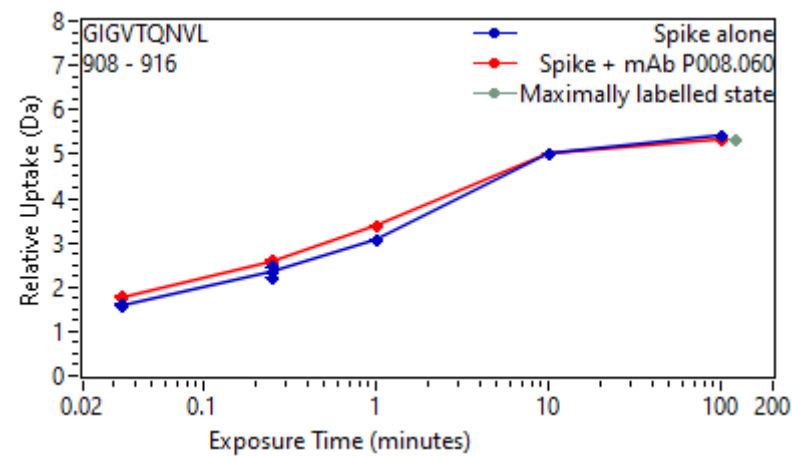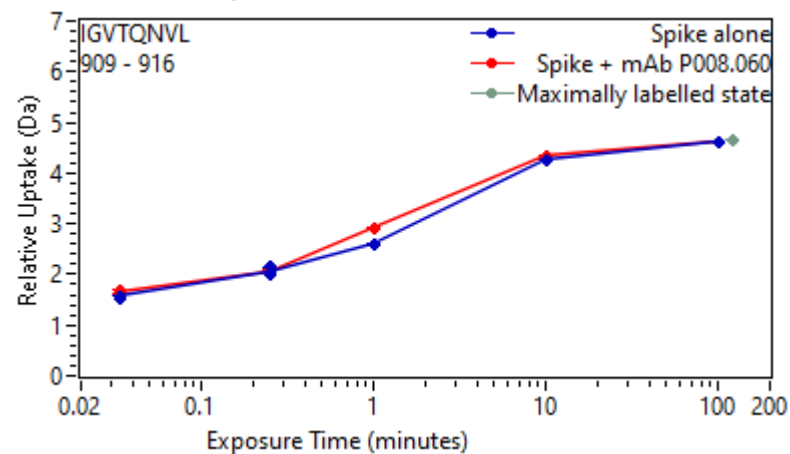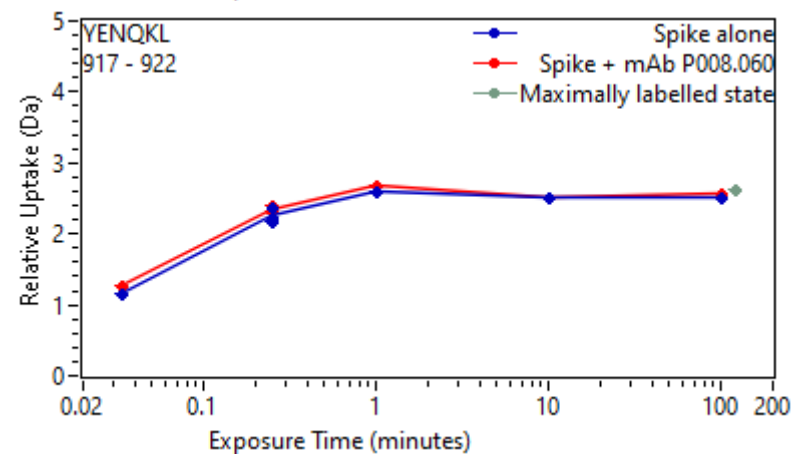

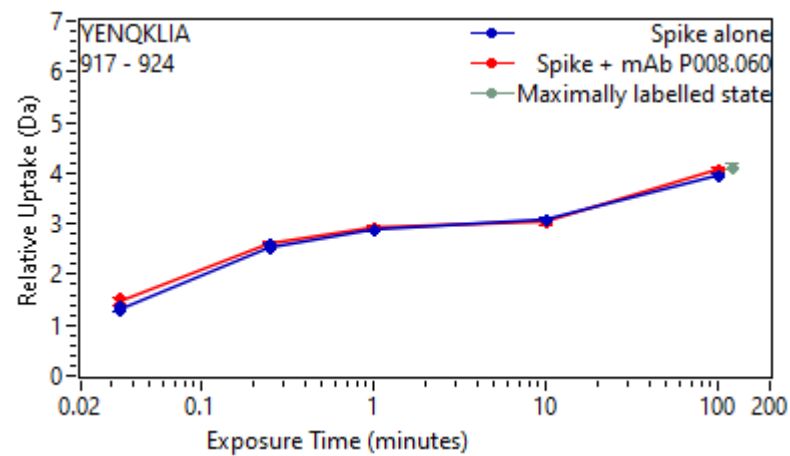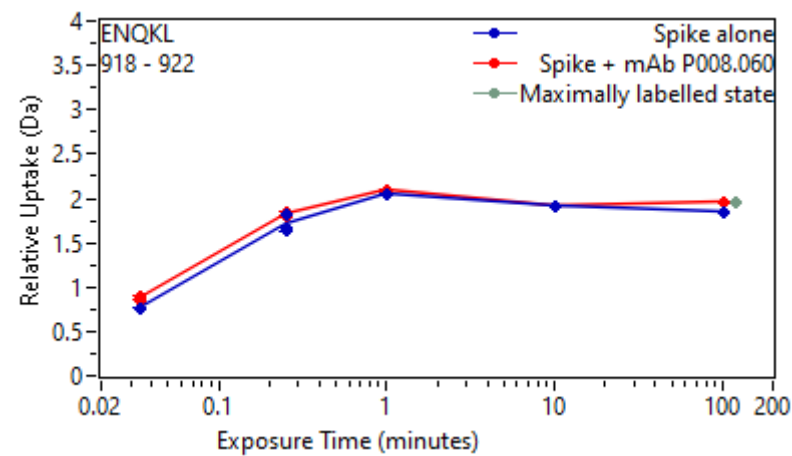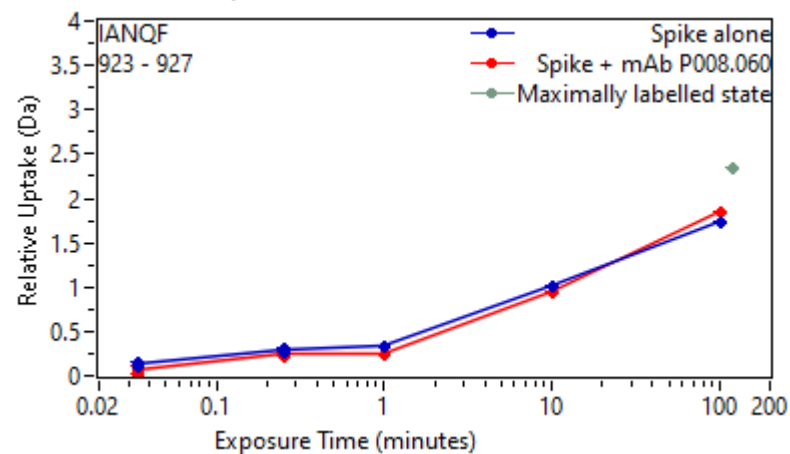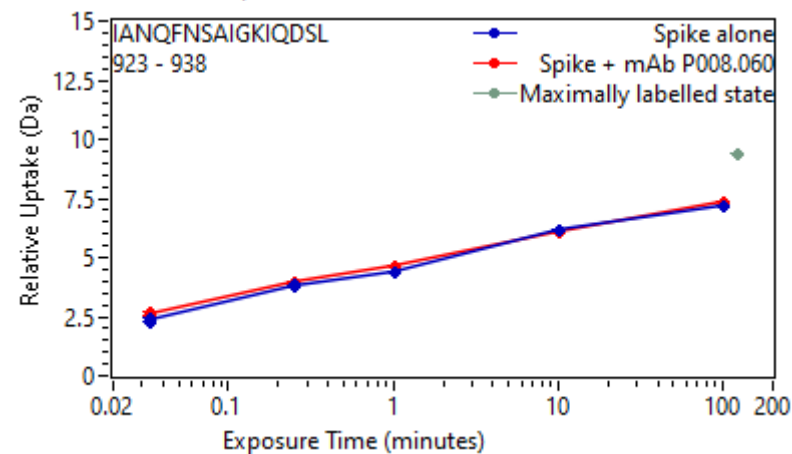

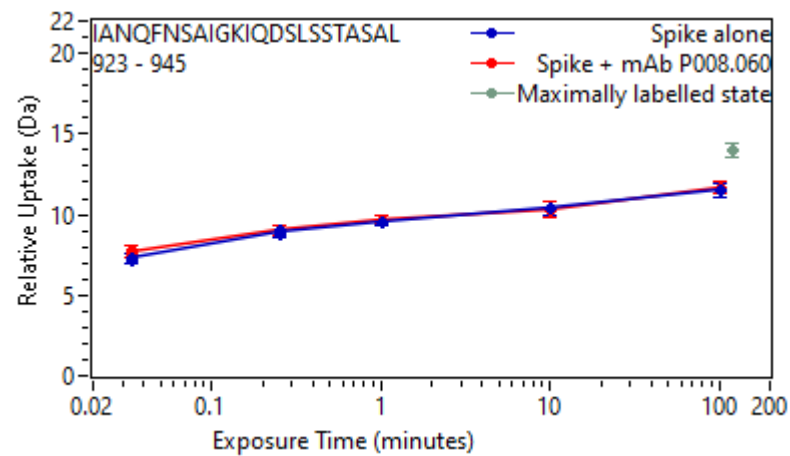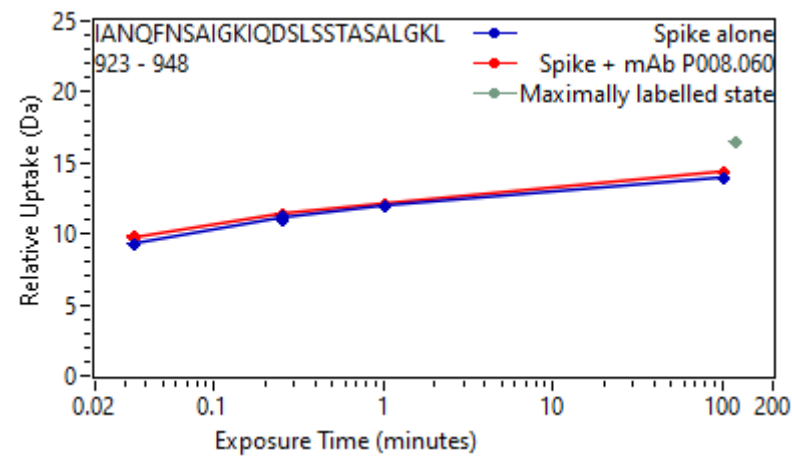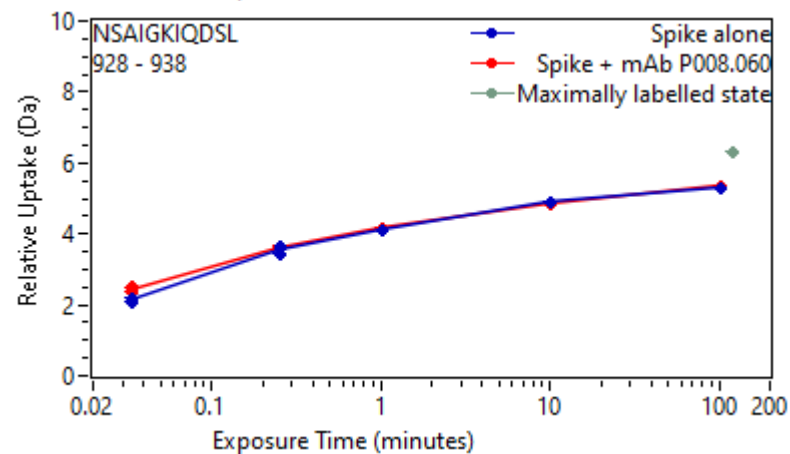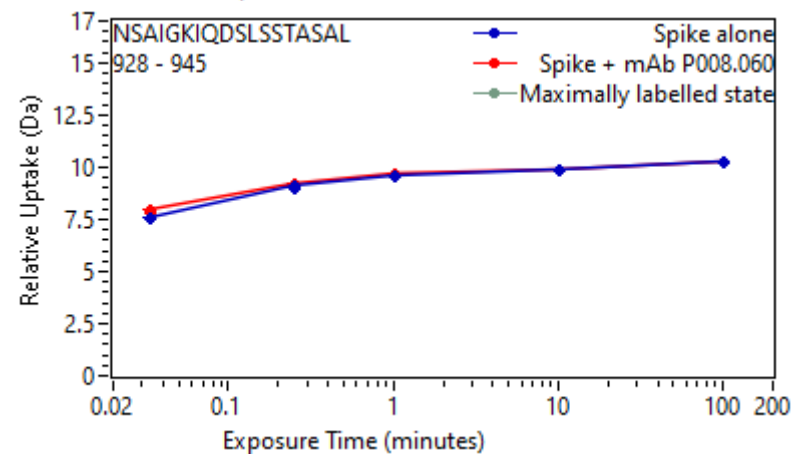

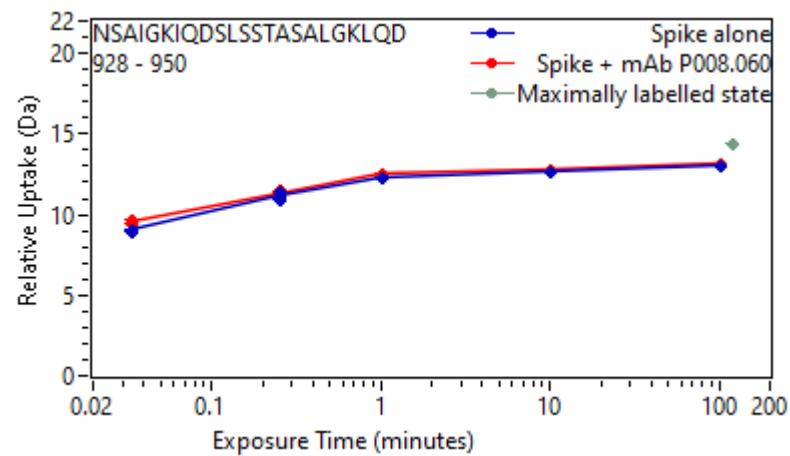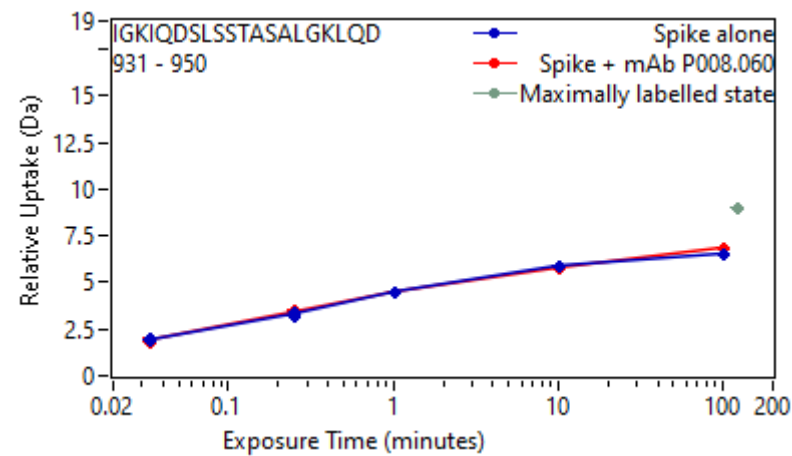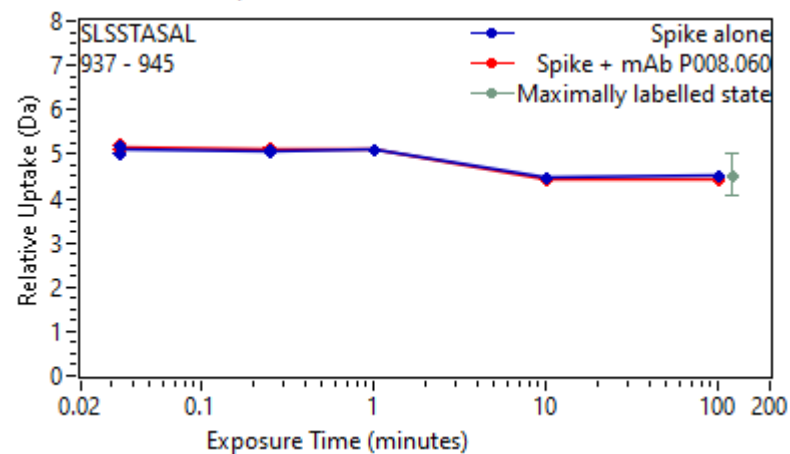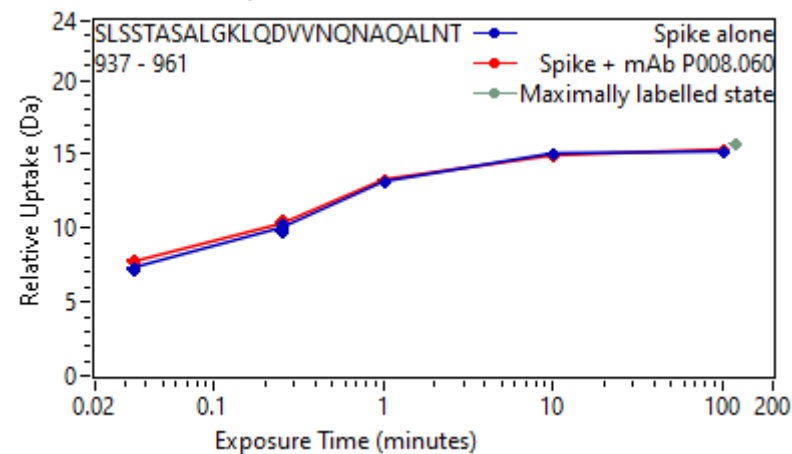

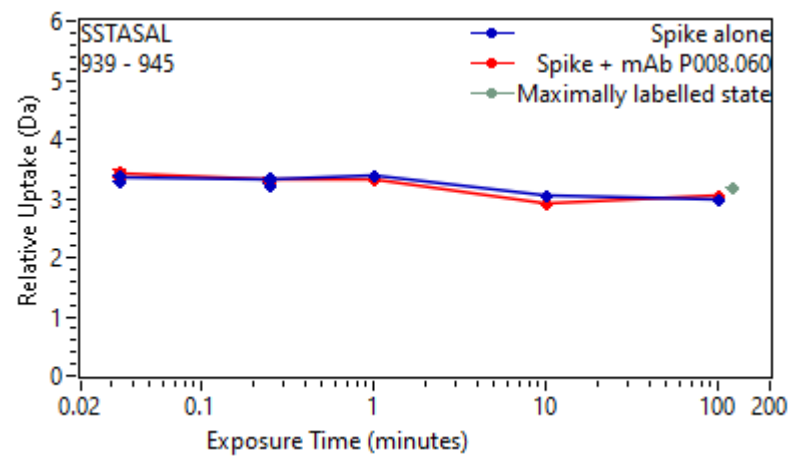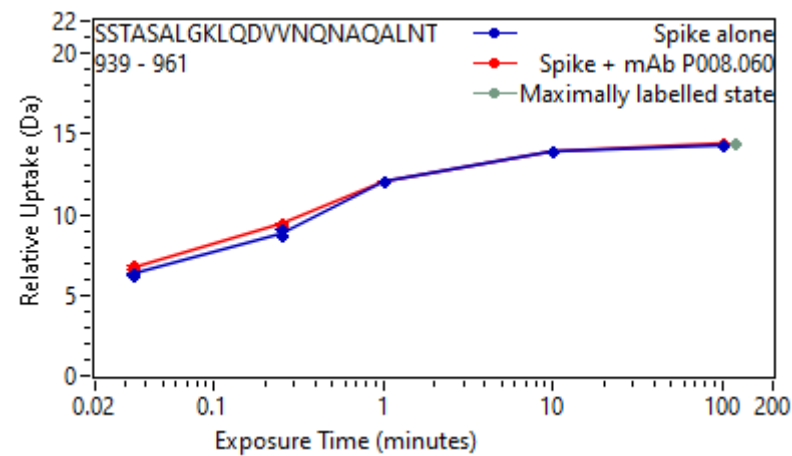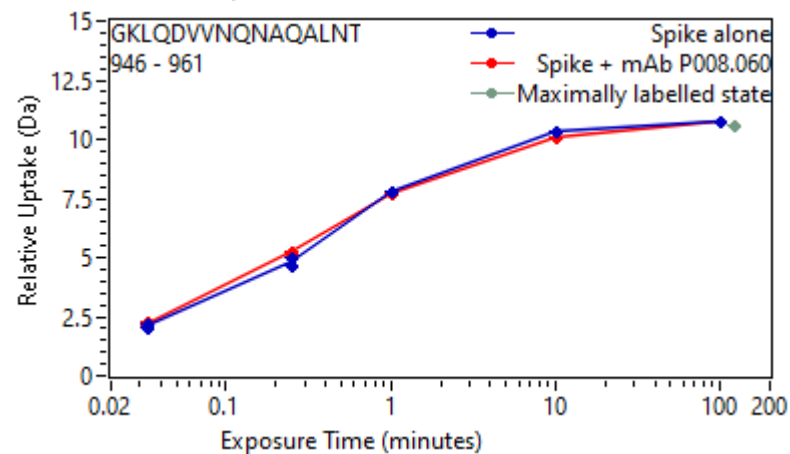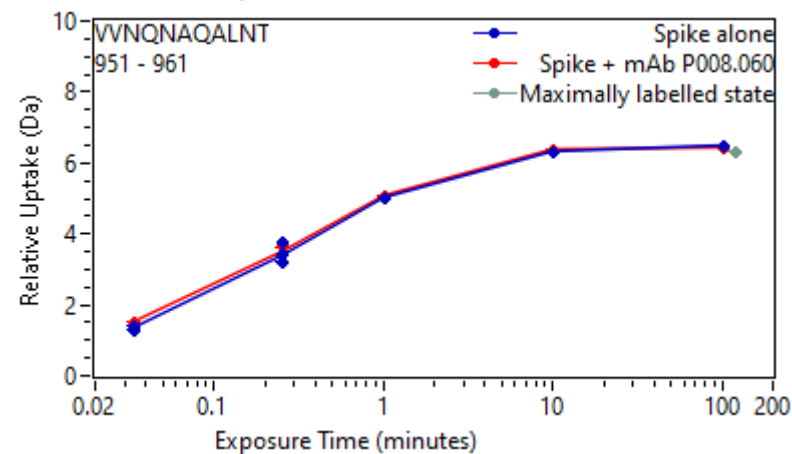

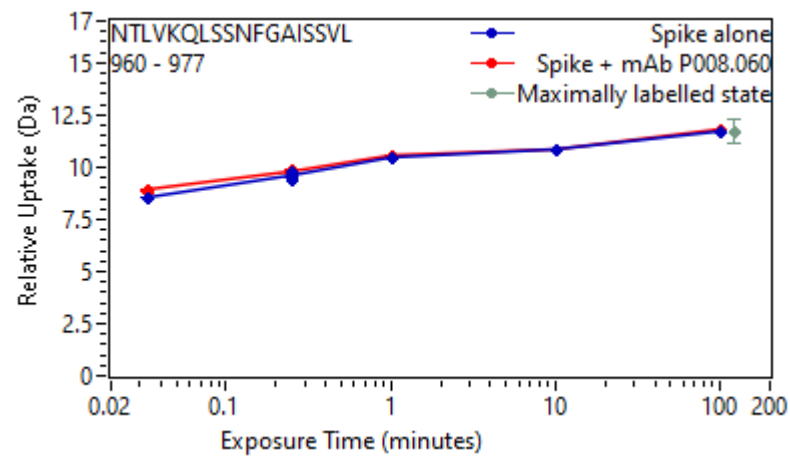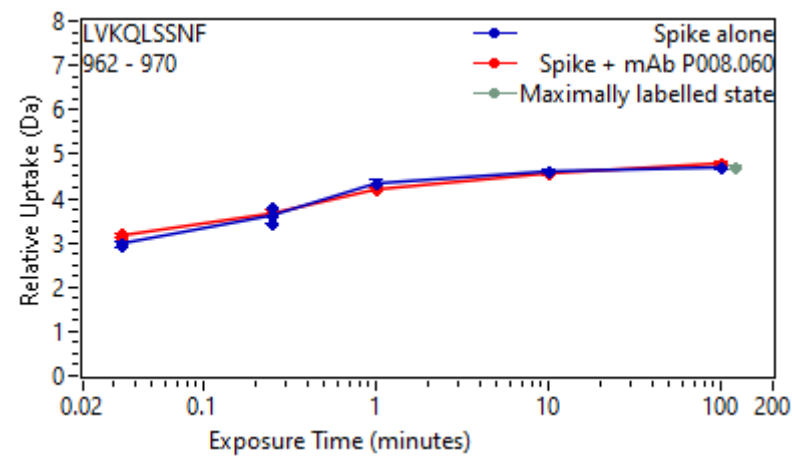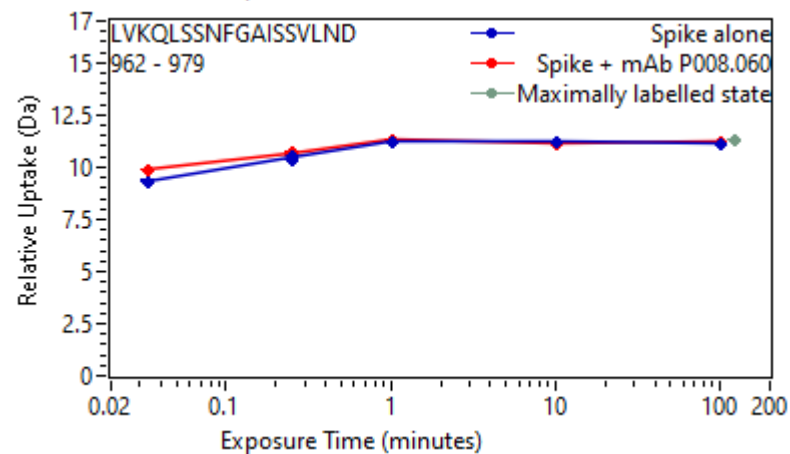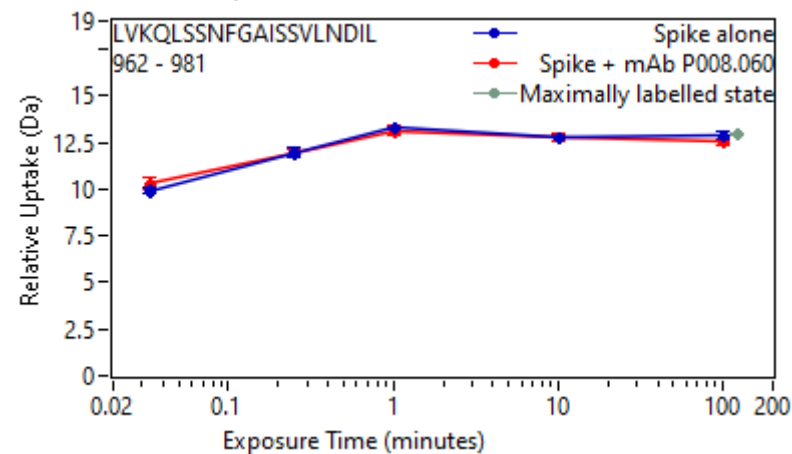

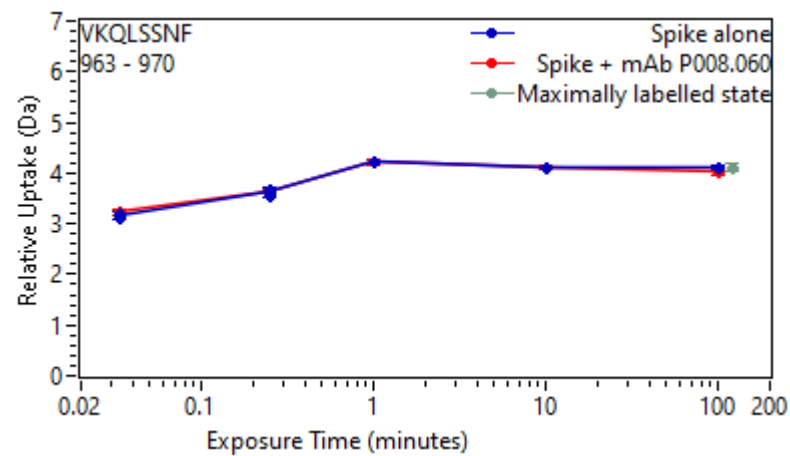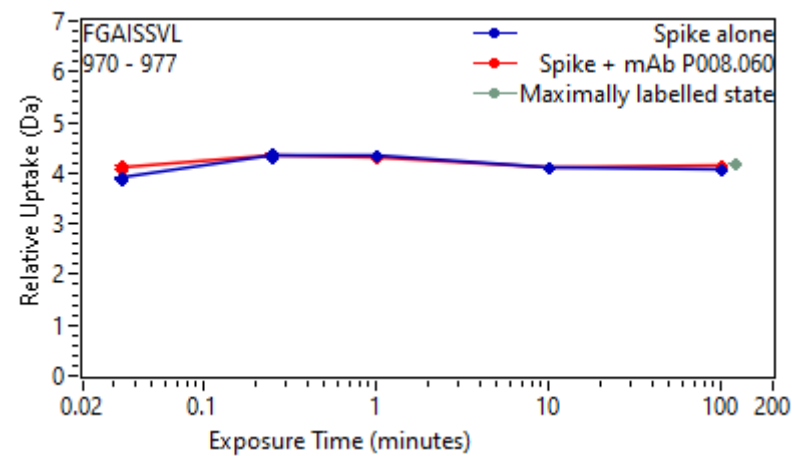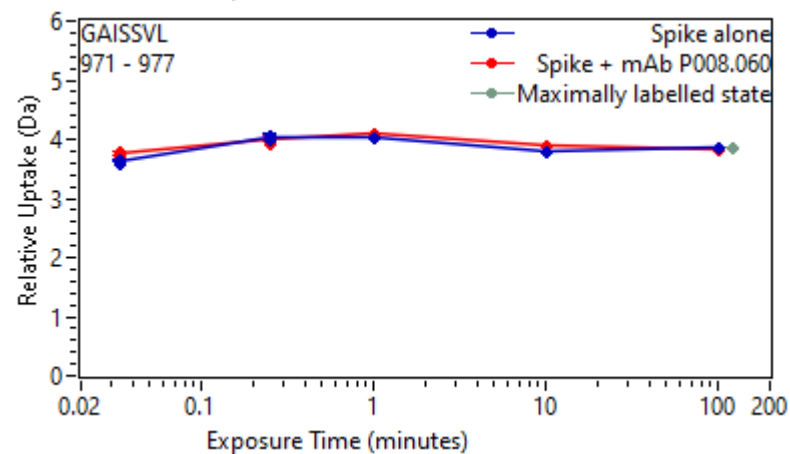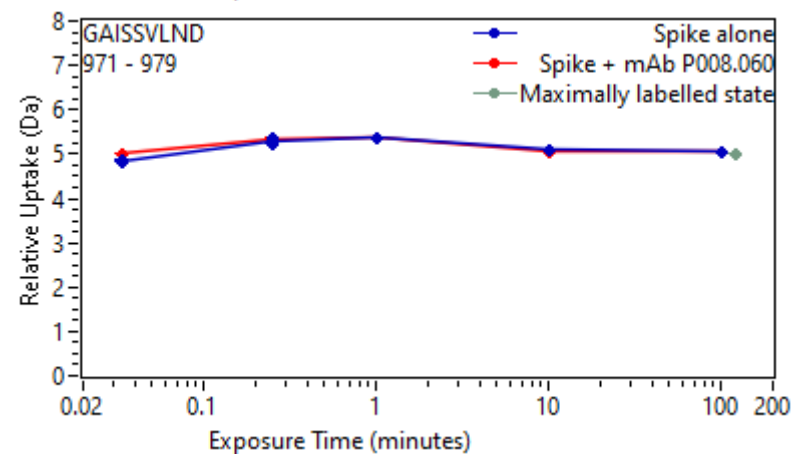

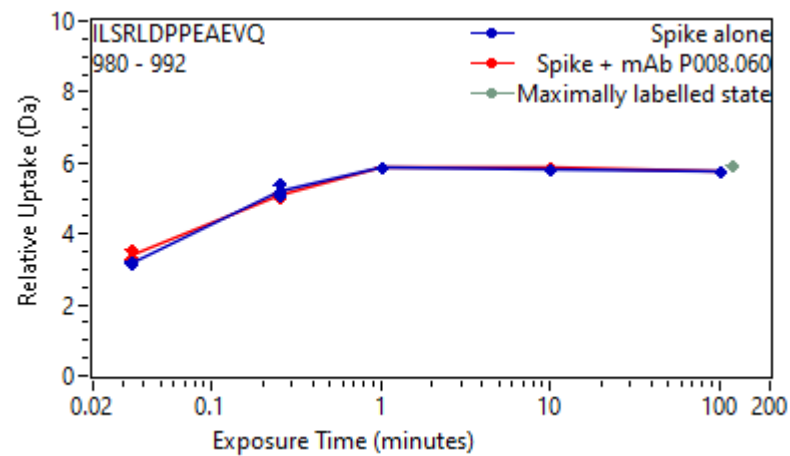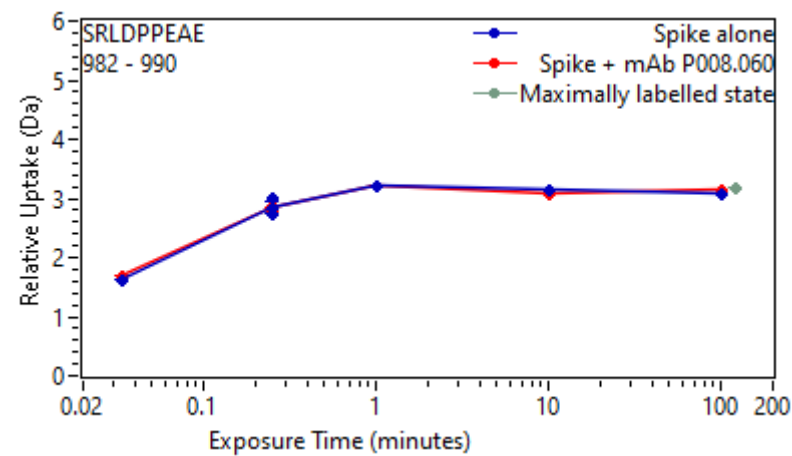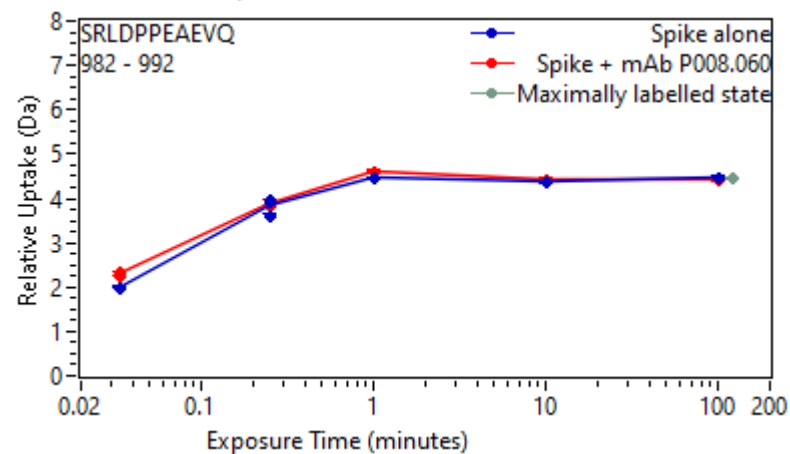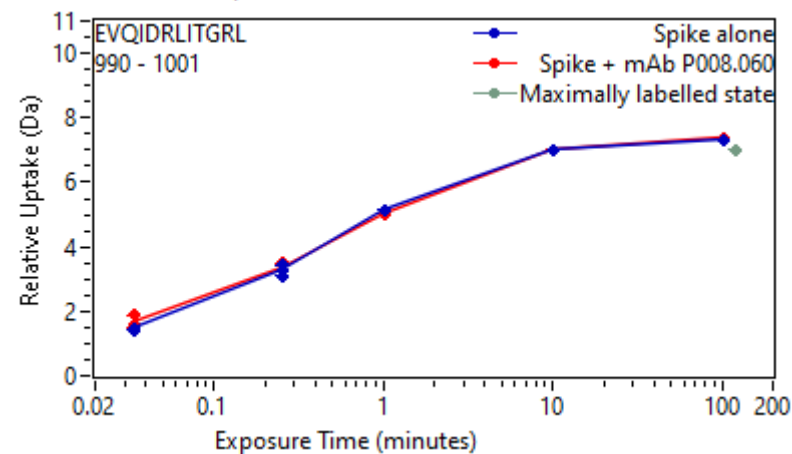

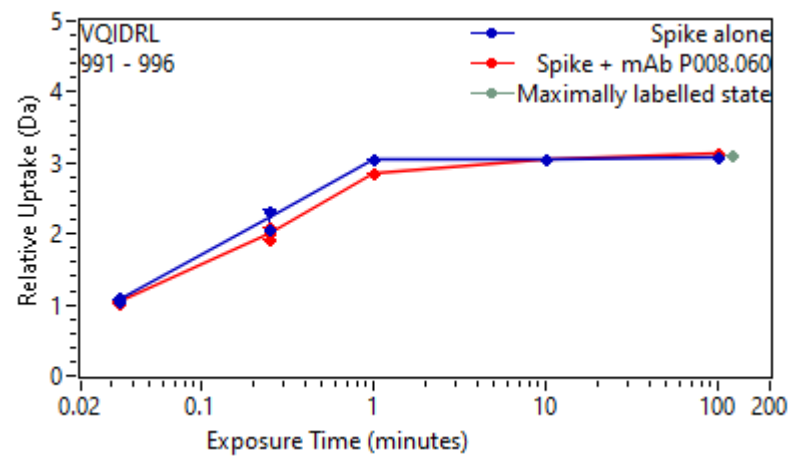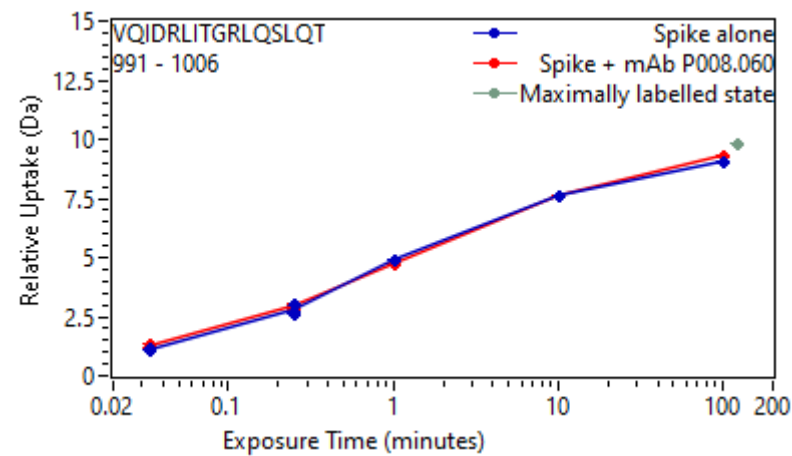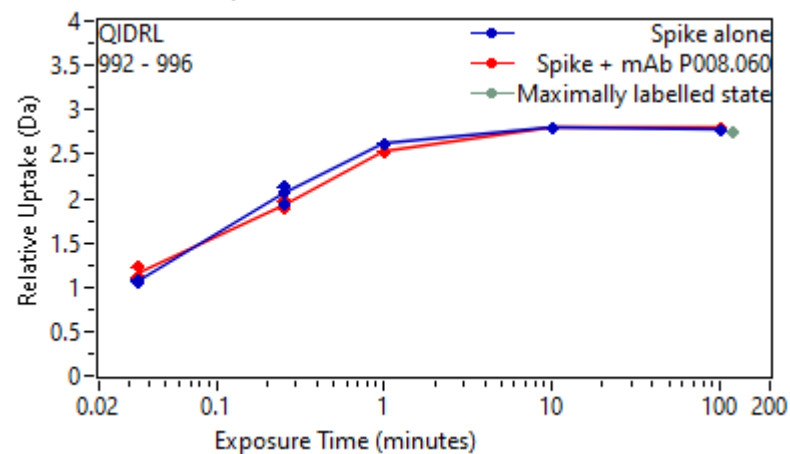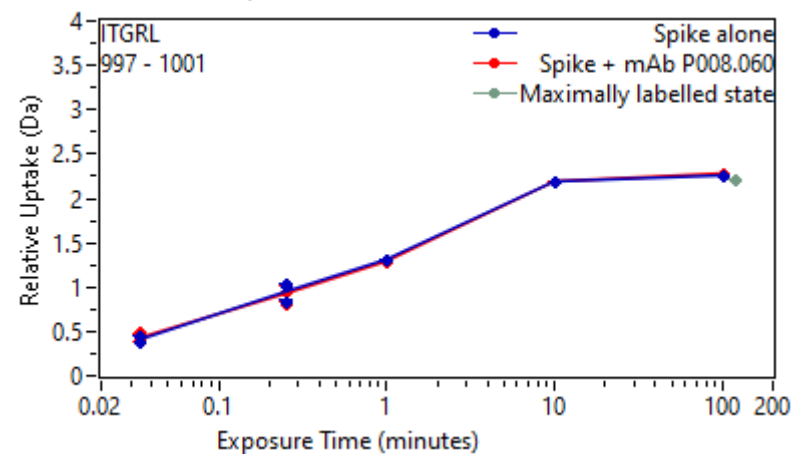

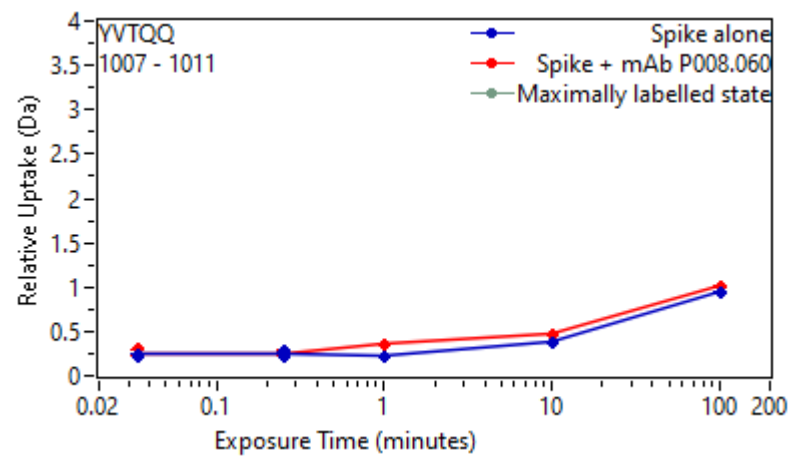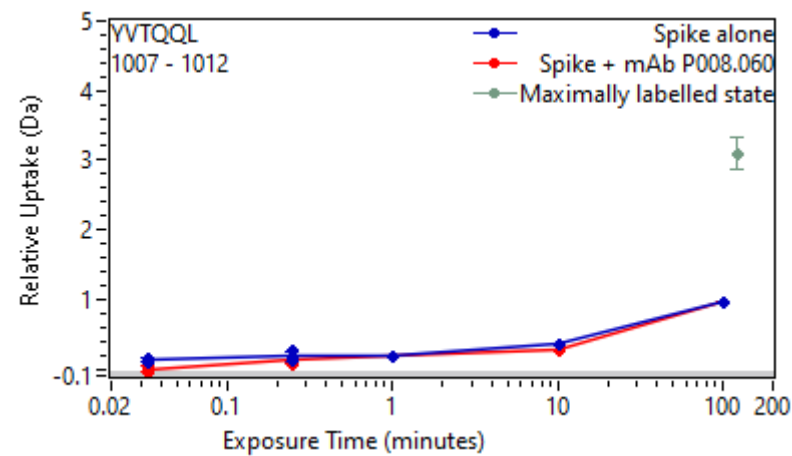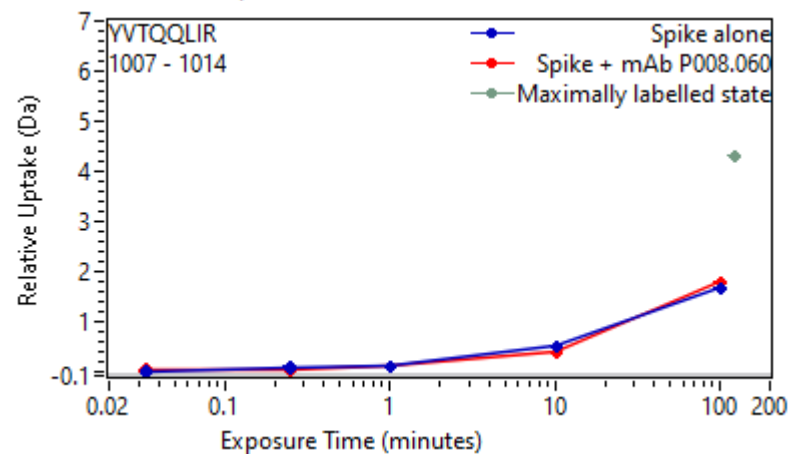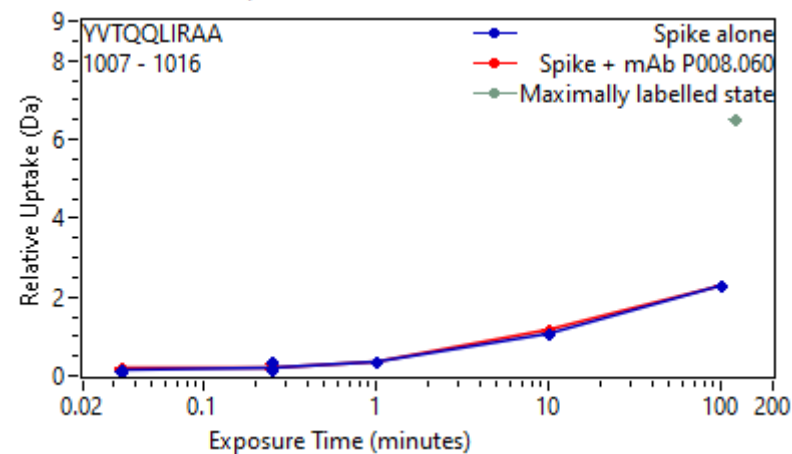

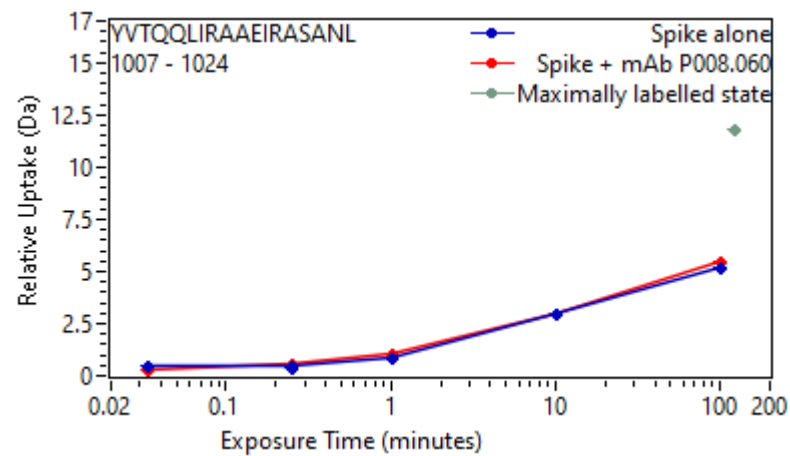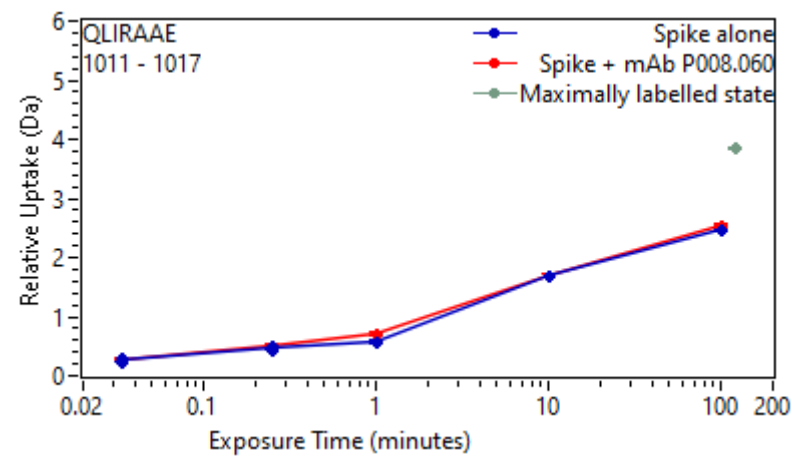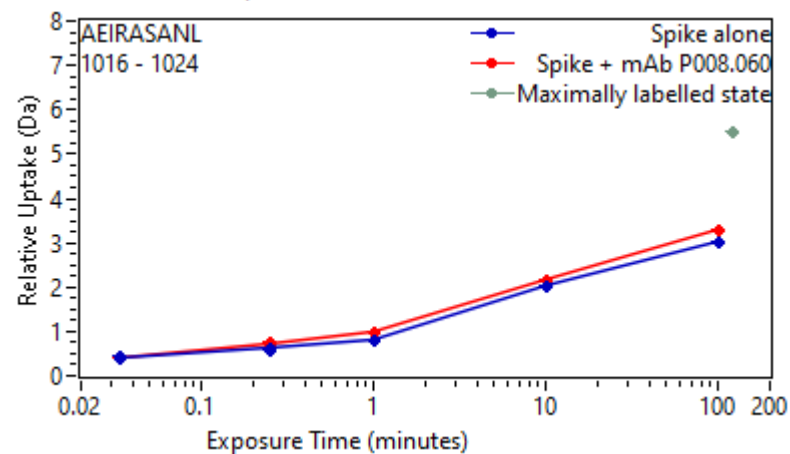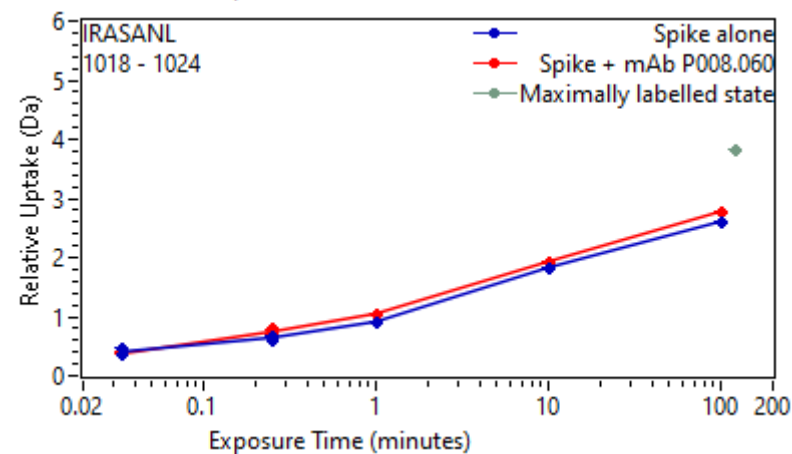

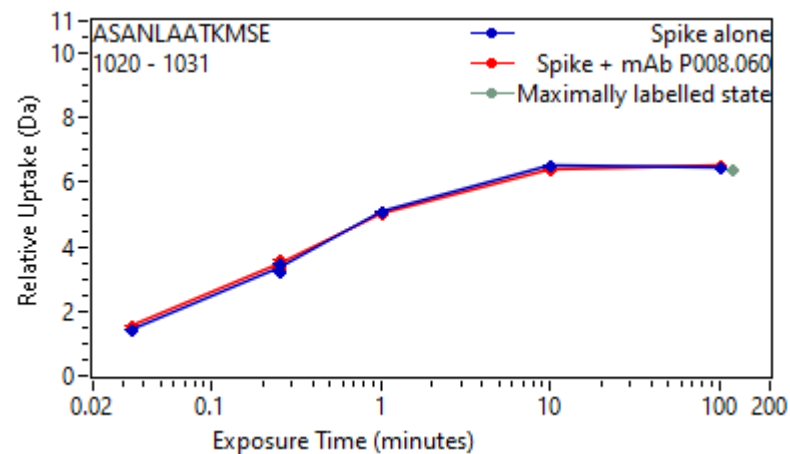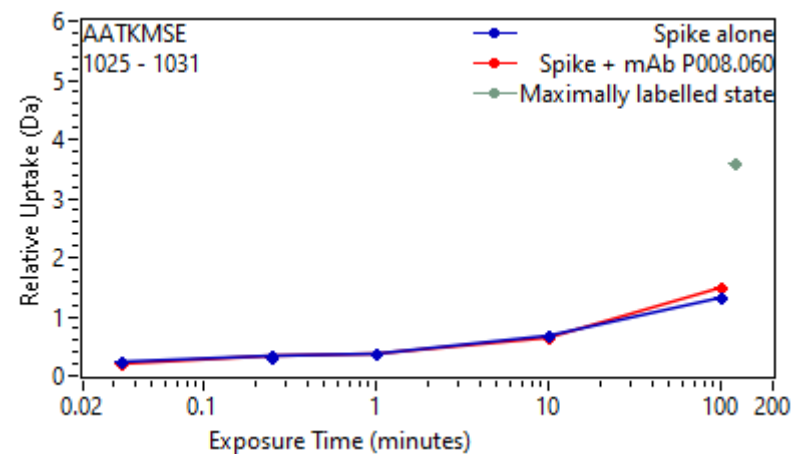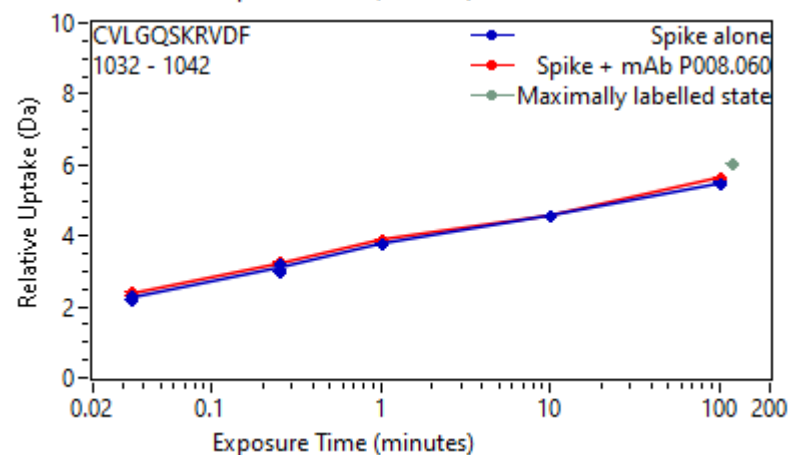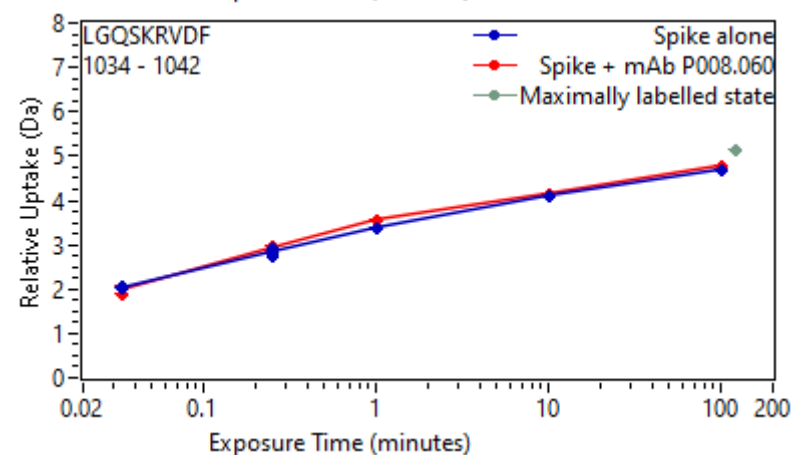

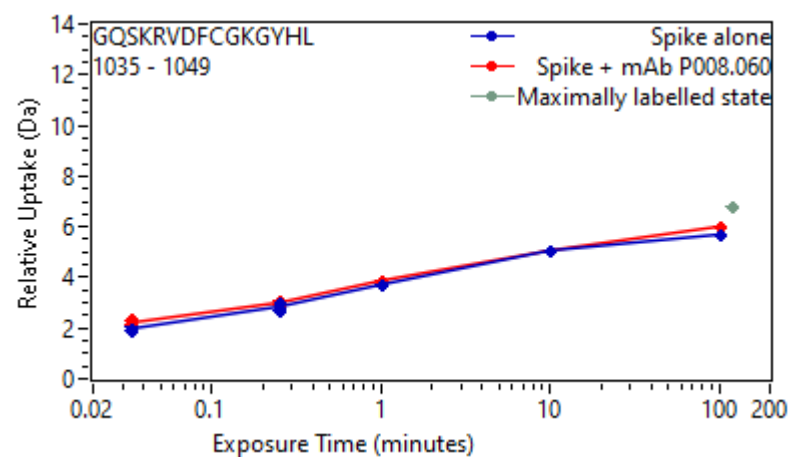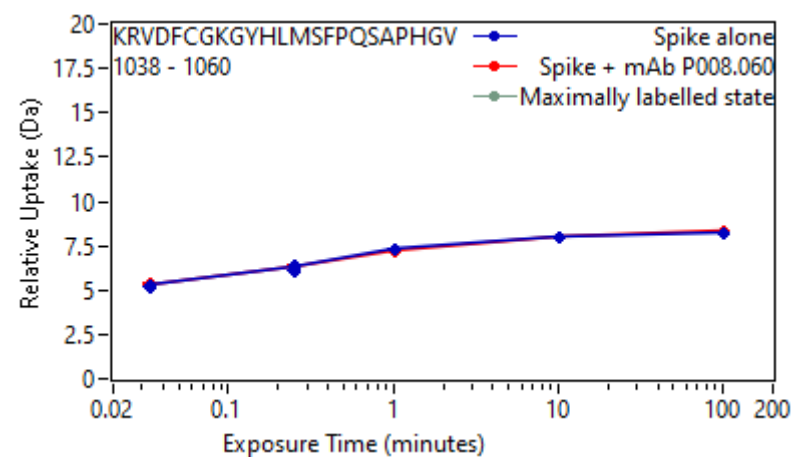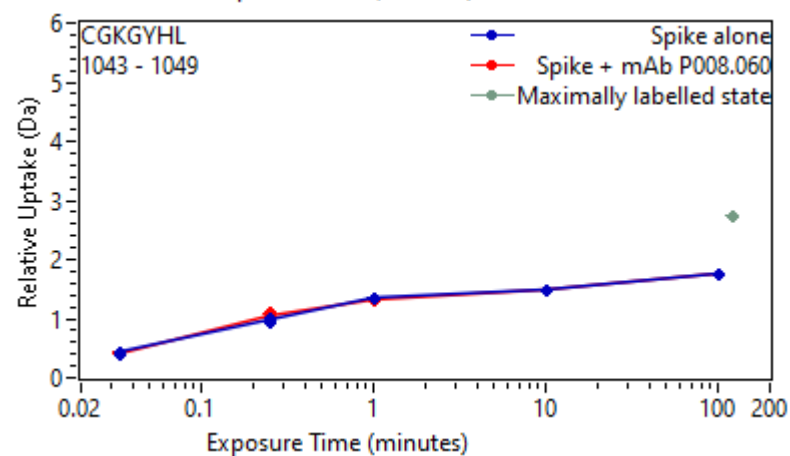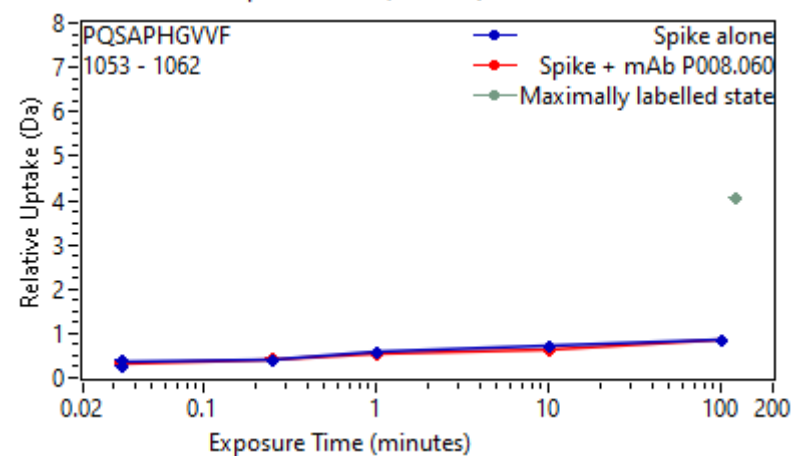

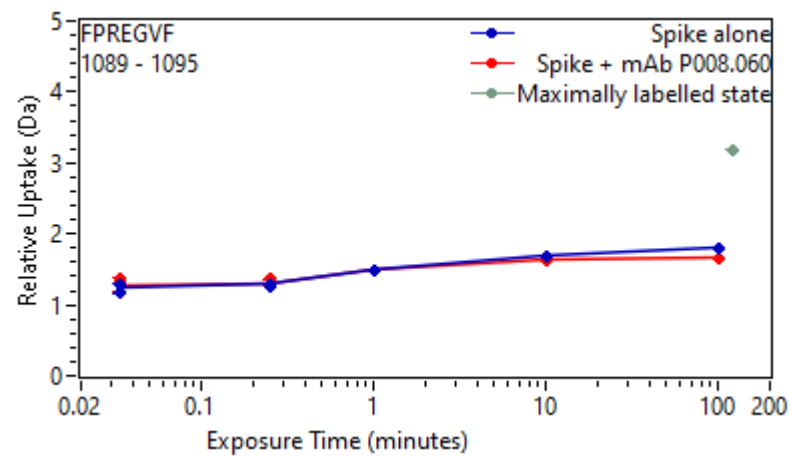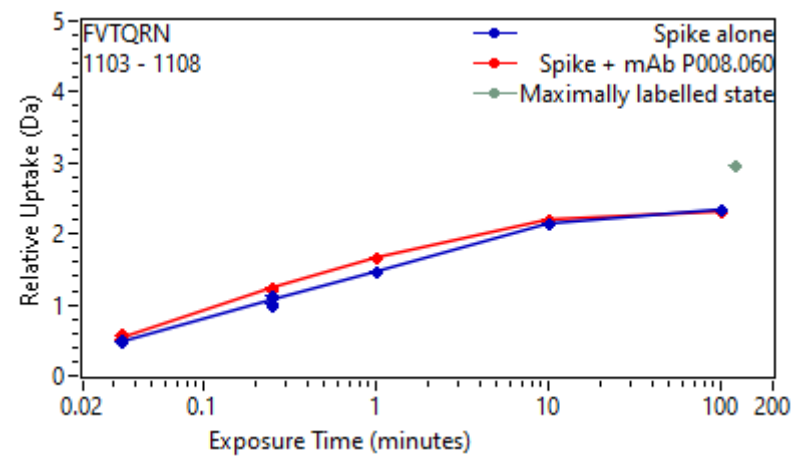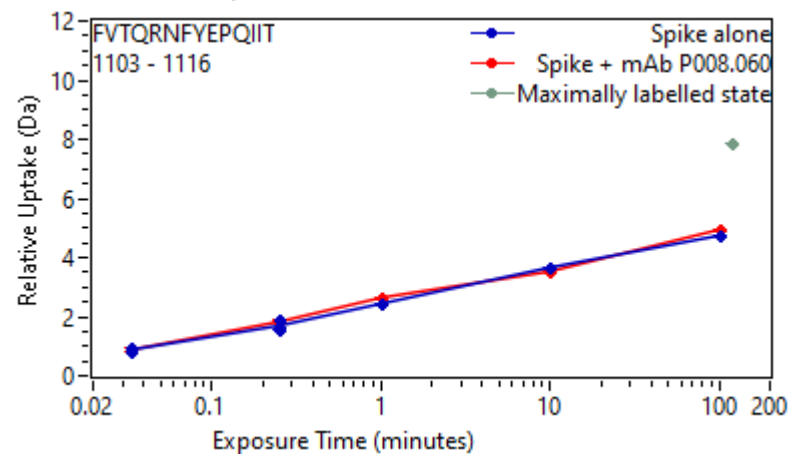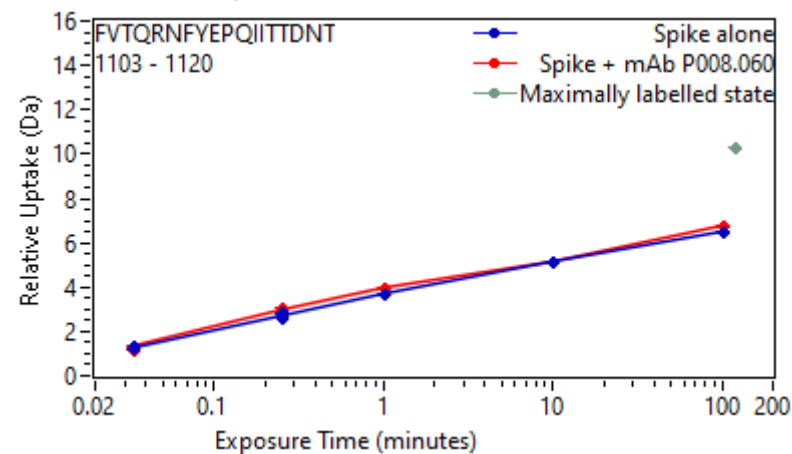

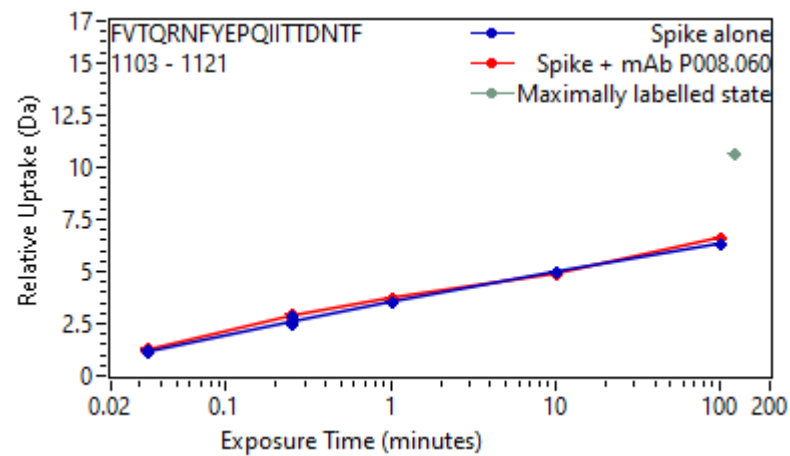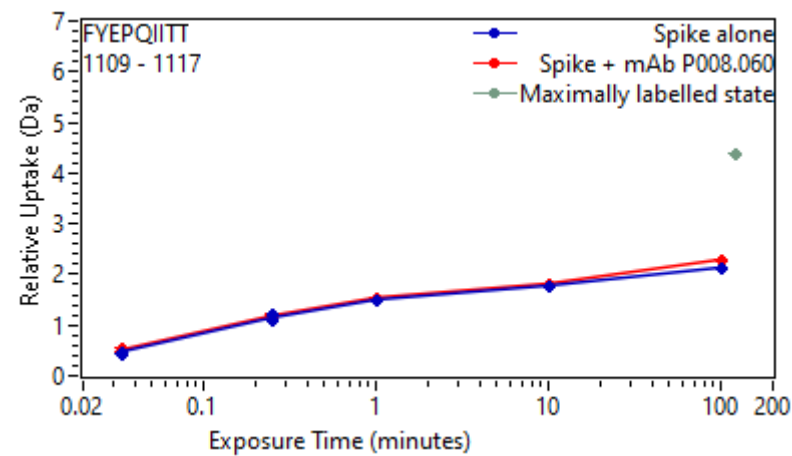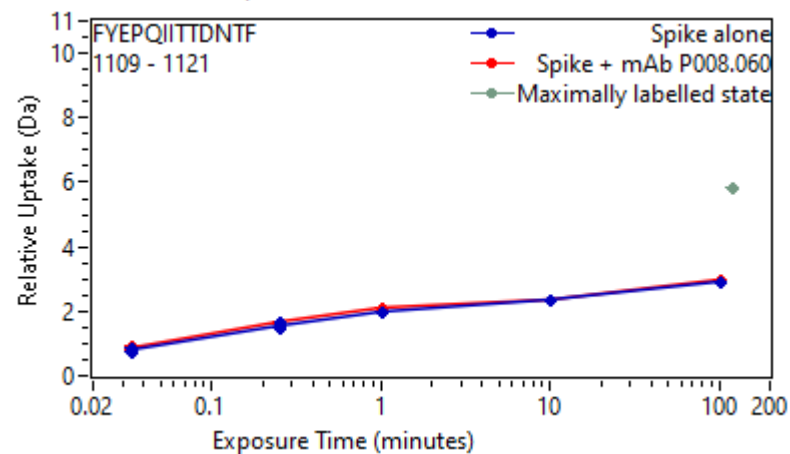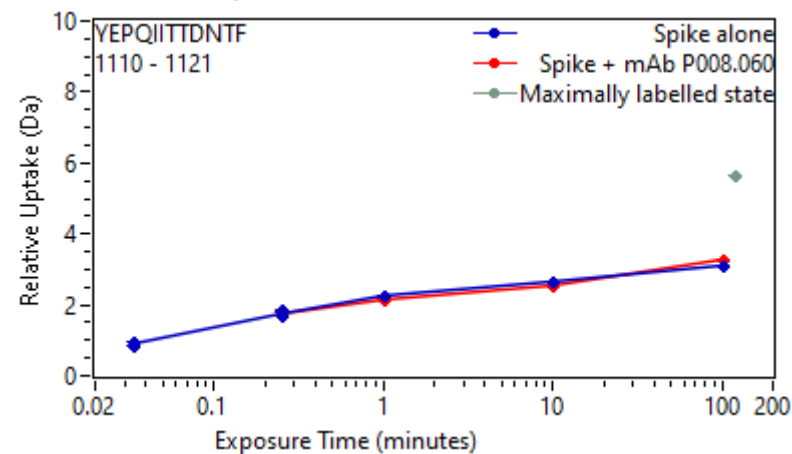

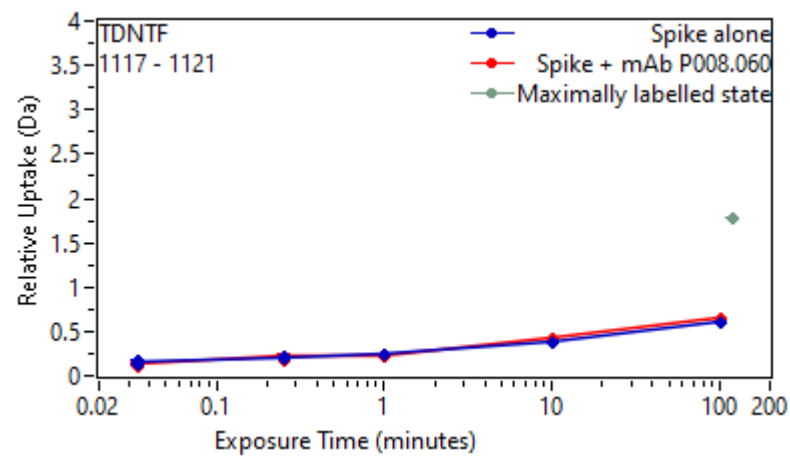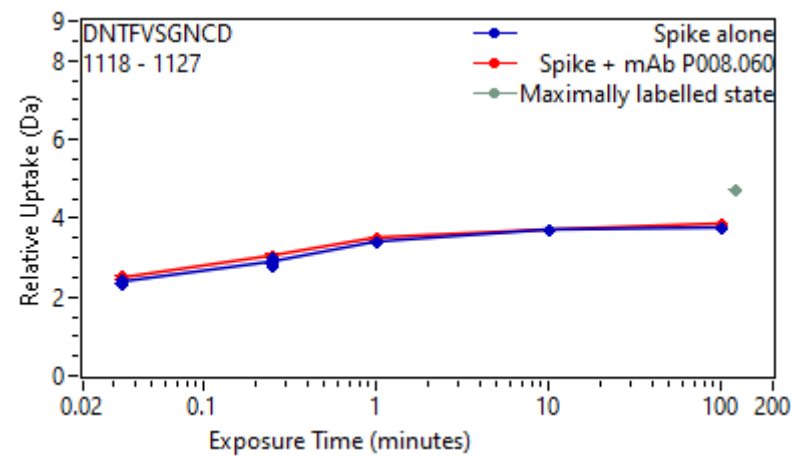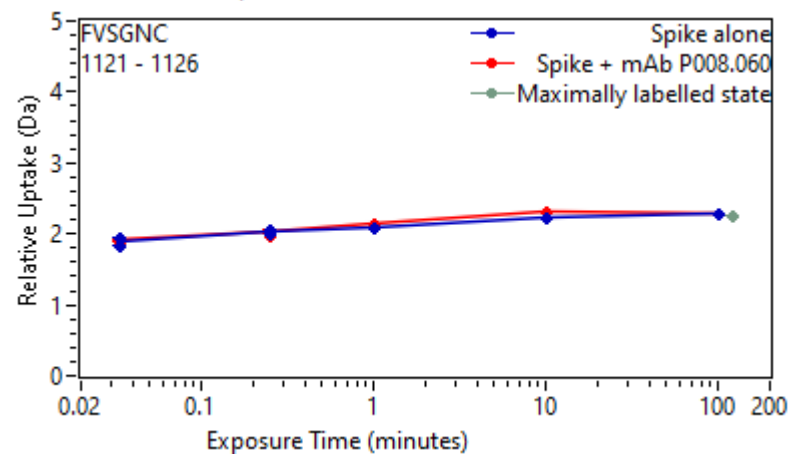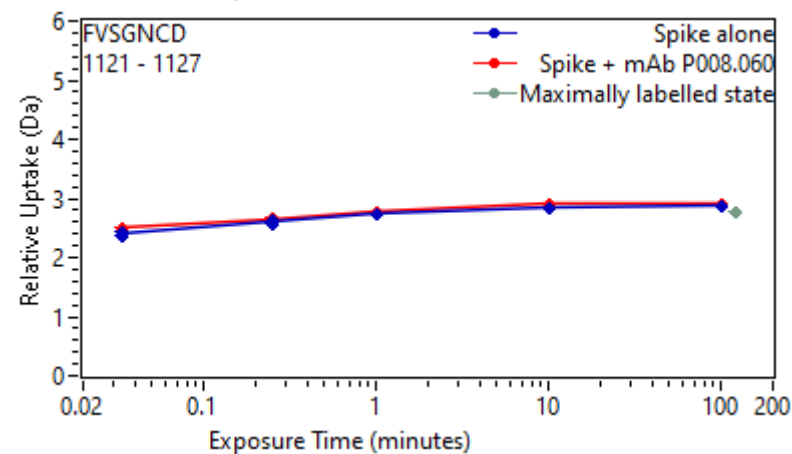

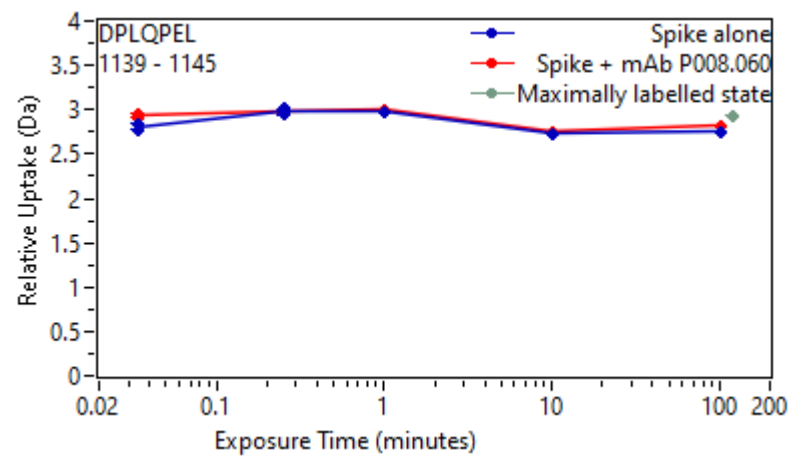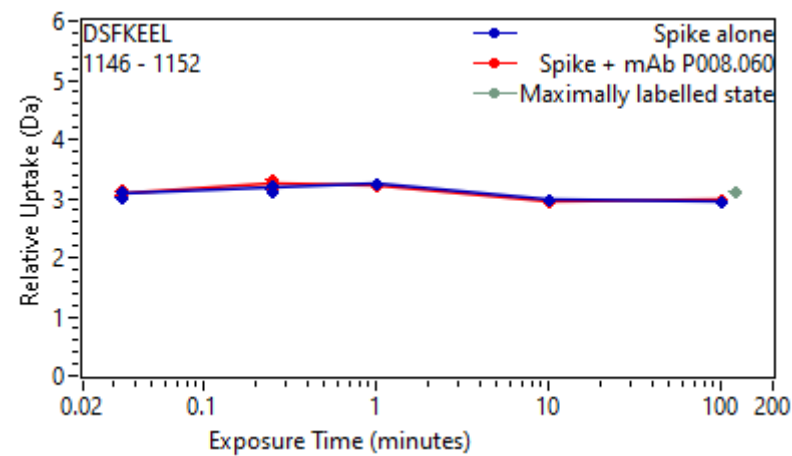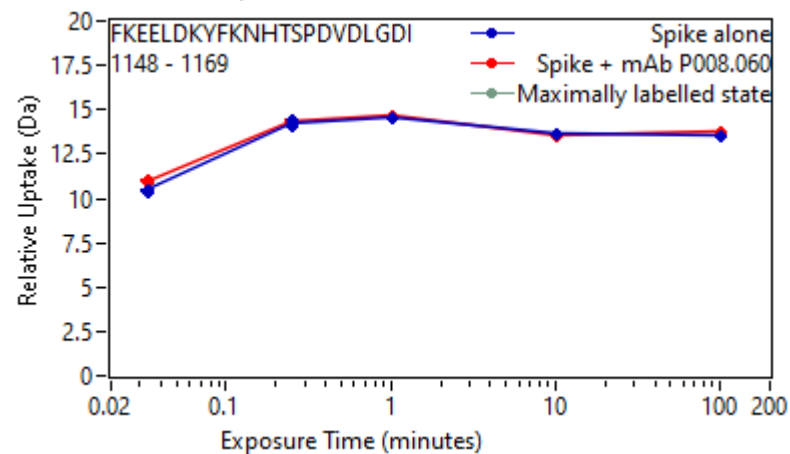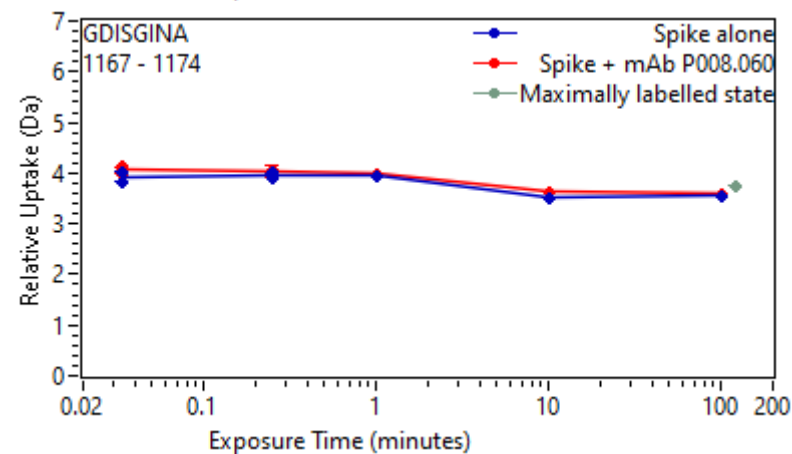

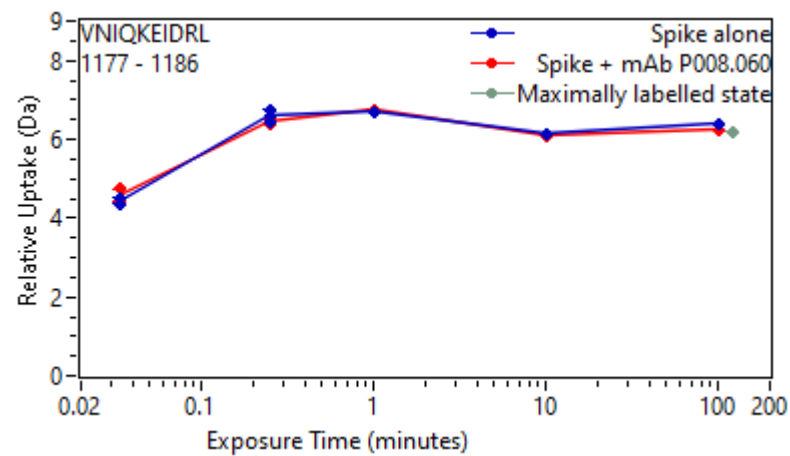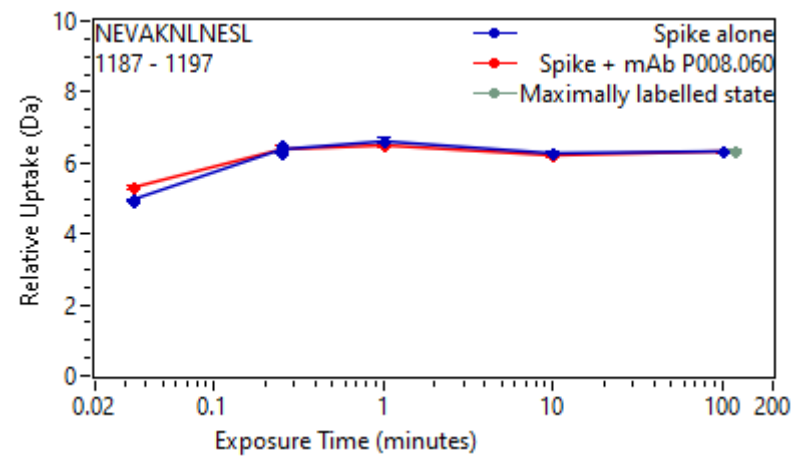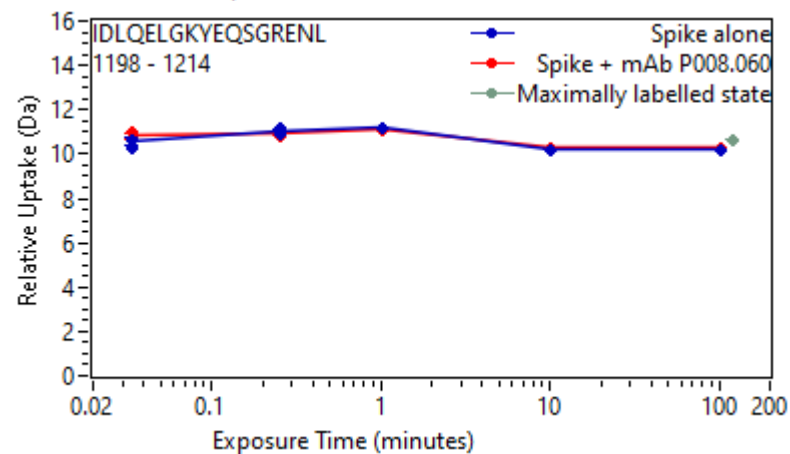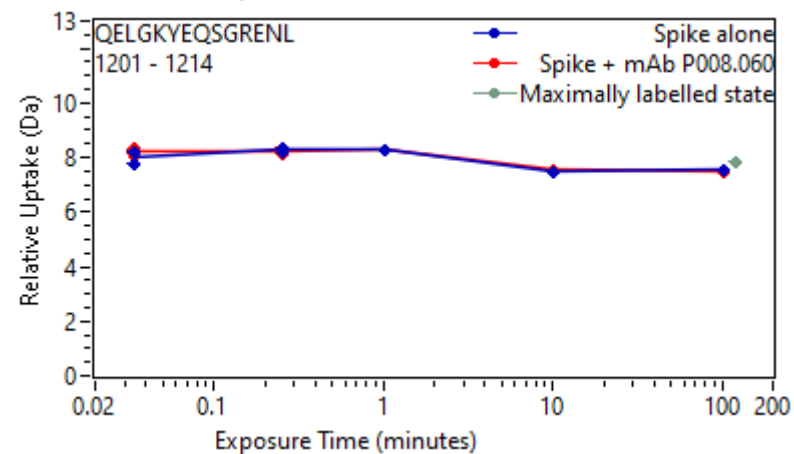

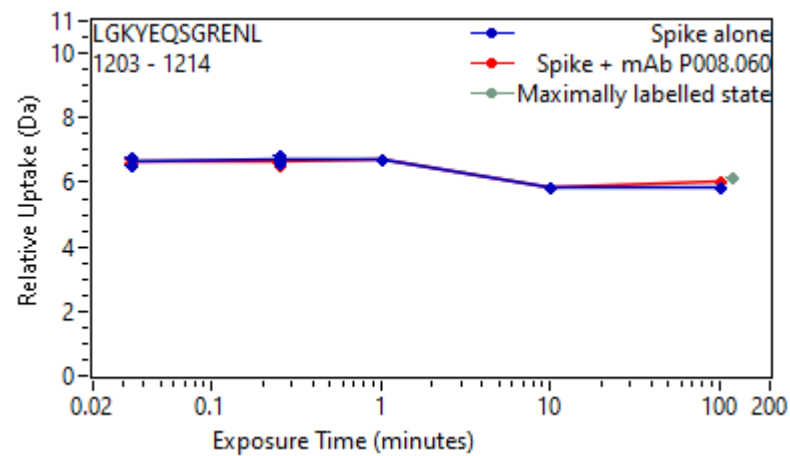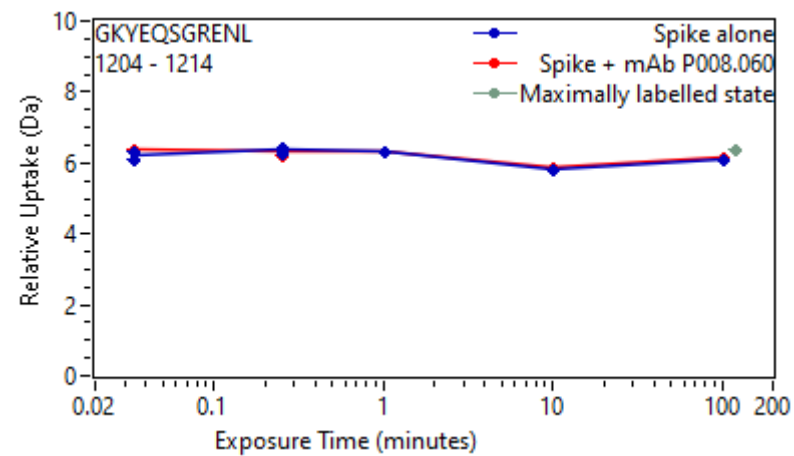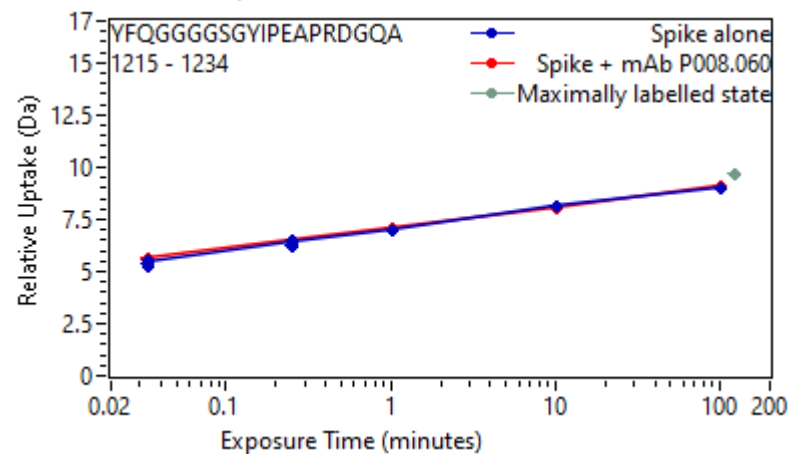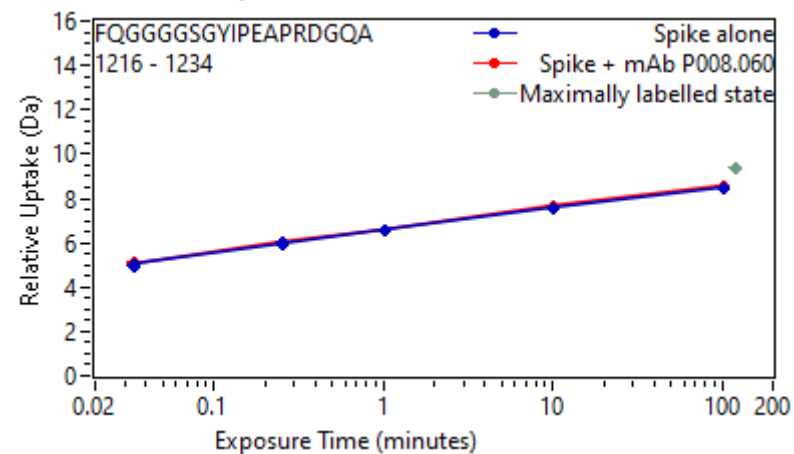

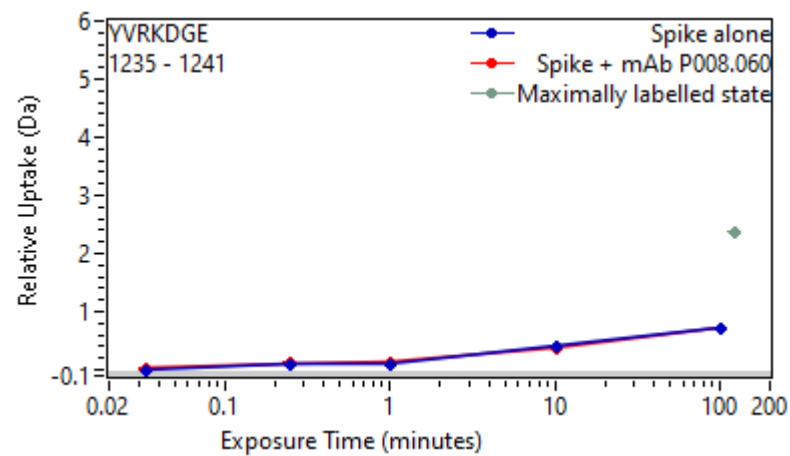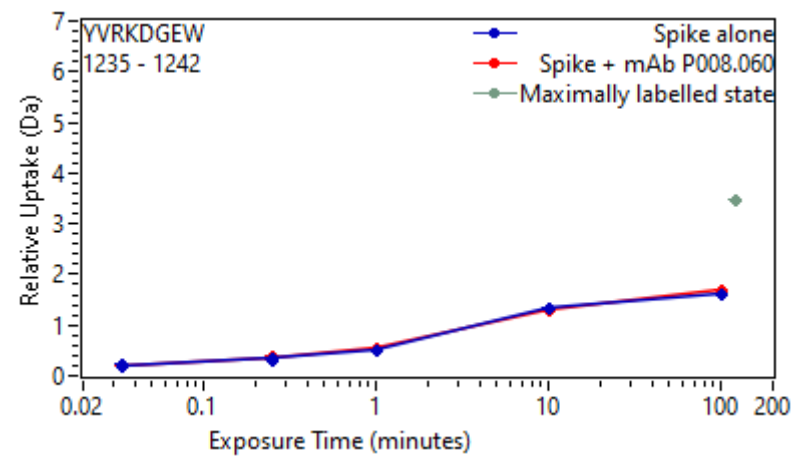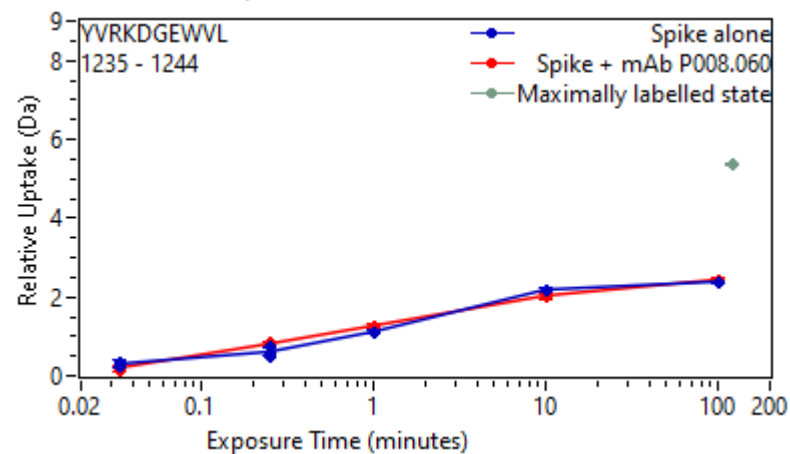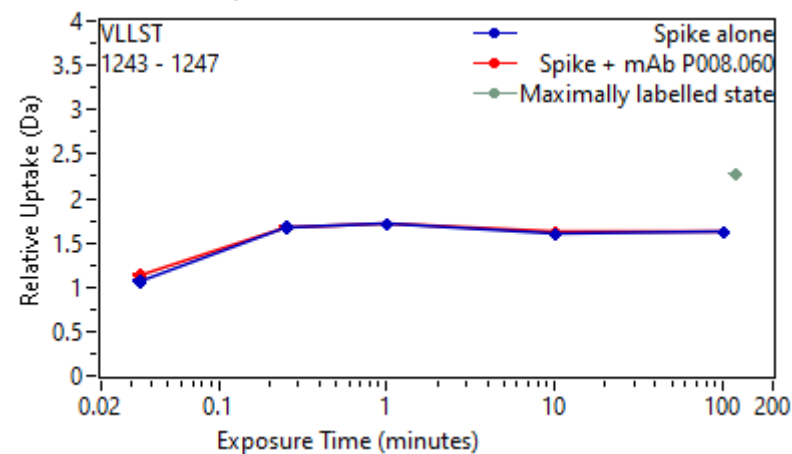

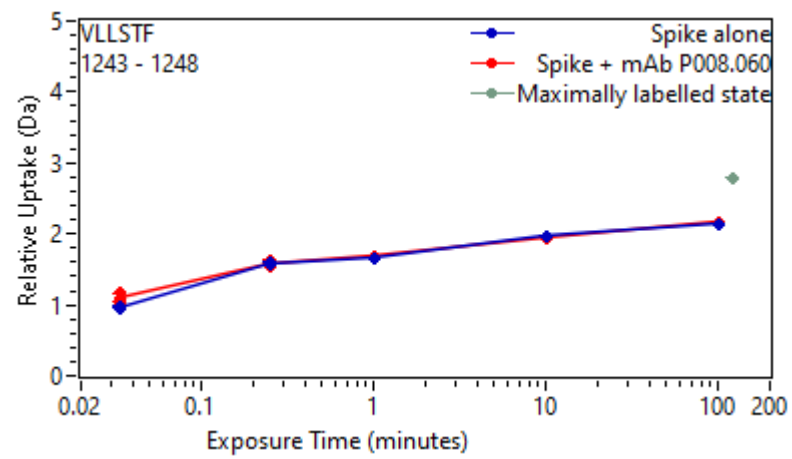

Supplement: Data S2. Deuterium uptake plots for peptides whose HDX was followed, related to Figure 3 [file mmc3.pdf]
