# Supplementary material for: A neutralizing epitope on the SD1 domain of SARS-CoV-2 spike targeted following infection and vaccination
Source: Cell Rep. 2022 Aug 11;40(8):111276. doi: 10.1016/j.celrep.2022.111276 (PMC9365860; doi:10.1016/j.celrep.2022.111276)
Supplement: Document S2. Article plus supplemental information [file mmc4.pdf]

Cell Reports

Article

A neutralizing epitope on the SD1 domain of SARS-CoV-2 spike targeted following infection and vaccination

Graphical abstract

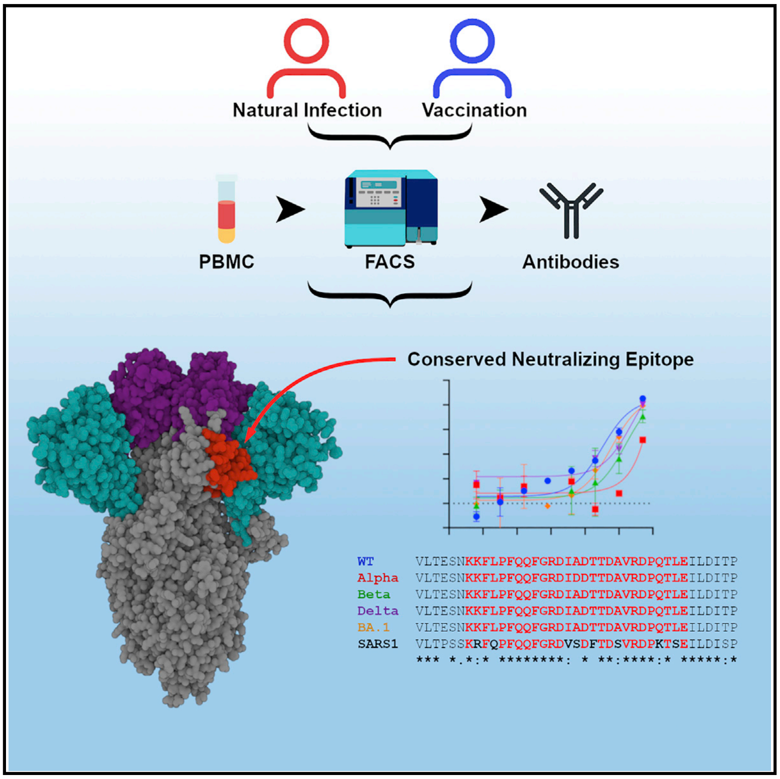

Authors

Jeffrey Seow, Hataf Khan, Annachiara Rosa, ..., Argyris Politis, Peter Cherepanov, Katie J. Doores

Correspondence

argyris.politis@kcl.ac.uk (A.P.), peter.cherepanov@crick.ac.uk (P.C.), katie.doores@kcl.ac.uk (K.J.D.)

In brief

Seow et al. identify a class of broadly neutralizing antibodies that bind a conserved epitope on the spike subdomain 1 (SD1) and that are elicited following infection and vaccination. The SD1 epitope is occluded on spike prefusion structures, suggesting binding to a conformational state of spike.

Highlights

- A neutralizing epitope on spike subdomain 1 (SD1) is identified
- The SD1 epitope is conserved between current SARS-CoV-2 variants and SARS-CoV
- SD1 antibodies arise from infection and vaccination
- Cryo-EM reveals the SD1 epitope is occluded on many SARS-CoV-2 spike structures

Seow et al., 2022, Cell Reports *40*, 111276

August 23, 2022 © 2022 The Author(s).

https://doi.org/10.1016/j.celrep.2022.111276

CellPress

Cell Reports

CellPress
OPEN ACCESS

Article

A neutralizing epitope on the SD1 domain of SARS-CoV-2 spike targeted following infection and vaccination

Jeffrey Seow,[1,7] Hataf Khan,[1,7] Annachiara Rosa,[2,6,7] Valeria Calvaresi,[3,7] Carl Graham,[1] Suzanne Pickering,[1] Valerie E. Pye,[2] Nora B. Cronin,[4] Isabella Huettner,[1] Michael H. Malim,[1] Argyris Politis,[3,*] Peter Cherepanov,[2,5,*] and Katie J. Doores[1,8,*]

[1]Department of Infectious Diseases, School of Immunology & Microbial Sciences, King's College London, London, UK
[2]Chromatin Structure and Mobile DNA Laboratory, The Francis Crick Institute, London, UK
[3]Department of Chemistry, King's College London, London, UK
[4]LonCEM Facility, The Francis Crick Institute, London, UK
[5]Department of Infectious Disease, St-Mary's Campus, Imperial College London, London, UK
[6]Present address: Faculty of Medicine, Wolfson Education Center, Hammersmith Campus, Imperial College London, Du Cane Road, London W12 0NN, UK
[7]These authors contributed equally
[8]Lead contact
*Correspondence: argyris.politis@kcl.ac.uk (A.P.), peter.cherepanov@crick.ac.uk (P.C.), katie.doores@kcl.ac.uk (K.J.D.)
https://doi.org/10.1016/j.celrep.2022.111276

SUMMARY

Severe acute respiratory syndrome coronavirus 2 (SARS-CoV-2) spike is the target for neutralizing antibodies elicited following both infection and vaccination. While extensive research has shown that the receptor binding domain (RBD) and, to a lesser extent, the N-terminal domain (NTD) are the predominant targets for neutralizing antibodies, identification of neutralizing epitopes beyond these regions is important for informing vaccine development and understanding antibody-mediated immune escape. Here, we identify a class of broadly neutralizing antibodies that bind an epitope on the spike subdomain 1 (SD1) and that have arisen from infection or vaccination. Using cryo-electron microscopy (cryo-EM) and hydrogen-deuterium exchange coupled to mass spectrometry (HDX-MS), we show that SD1-specific antibody P008_60 binds an epitope that is not accessible within the canonical prefusion states of the SARS-CoV-2 spike, suggesting a transient conformation of the viral glycoprotein that is vulnerable to neutralization.

INTRODUCTION

The severe acute respiratory syndrome coronavirus 2 (SARS-CoV-2)-encoded spike glycoprotein is the key target for neutralizing antibodies generated following natural infection or vaccination. Spike proteins assemble into homotrimers on the viral membrane, with each spike monomer comprising two functional subunits, S1 and S2. The S1 subunit contains the N-terminal domain (NTD), the receptor binding domain (RBD), and the subdomains 1 and 2 (SD1 and SD2) (Wrapp et al., 2020). The RBD contains the receptor binding motif (RBM) that directly contacts the host receptor human angiotensin-converting enzyme 2 (ACE2) (Lan et al., 2020). The S2 subunit, containing the fusion peptide (FP), two heptad repeats (HR1 and HR2), the cytoplasmic (CP) tail, and the transmembrane (TM) domain, is crucial for viral fusion. In infected cells, newly produced spike trimers are cleaved at the S1-S2 interface by host cell furin, and the two subunits remain non-covalently bound in a pre-fusion state. Upon successful binding to ACE2, spike undergoes additional proteolytical cleavage by TMPRSS2 at the S2′ site located proximally to the FP. This results in the dissociation (shedding) of the S1 subunits, enabling insertion of the FPs into the target cell membrane. Finally, refolding of the S2 HR regions into a highly stable and compact six-helical bundle induces fusion of the viral and host cell membranes (Gavor et al., 2020; Huang et al., 2020; Tay et al., 2020).

Numerous neutralizing epitopes have been identified on spike, including epitopes on RBD, NTD, and S2, and their modes of antibody recognition characterized at the molecular level (Castro Dopico et al., 2022; Corti et al., 2021; Yuan et al., 2021). In particular, four classes of RBD-targeting neutralizing antibodies have been identified including neutralizing antibodies recognizing the ACE2 RBM (Barnes et al., 2020; Brouwer et al., 2020; Robbiani et al., 2020), while a neutralization supersite has also been identified on NTD (Cerutti et al., 2021; Graham et al., 2021; McCallum et al., 2021; Rosa et al., 2021). Antibodies targeting several of these epitopes have been licensed for treating coronavirus 2019 (COVID-19) (Hurt and Wheatley, 2021; Taylor et al., 2021). Spike is the antigen used in most licensed COVID-19 vaccines (Krammer, 2020), arguing that a more in-depth understanding of the interactions between spike and neutralizing and non-neutralizing antibodies elicited following

This is an open access article under the CC BY license (http://creativecommons.org/licenses/by/4.0/).

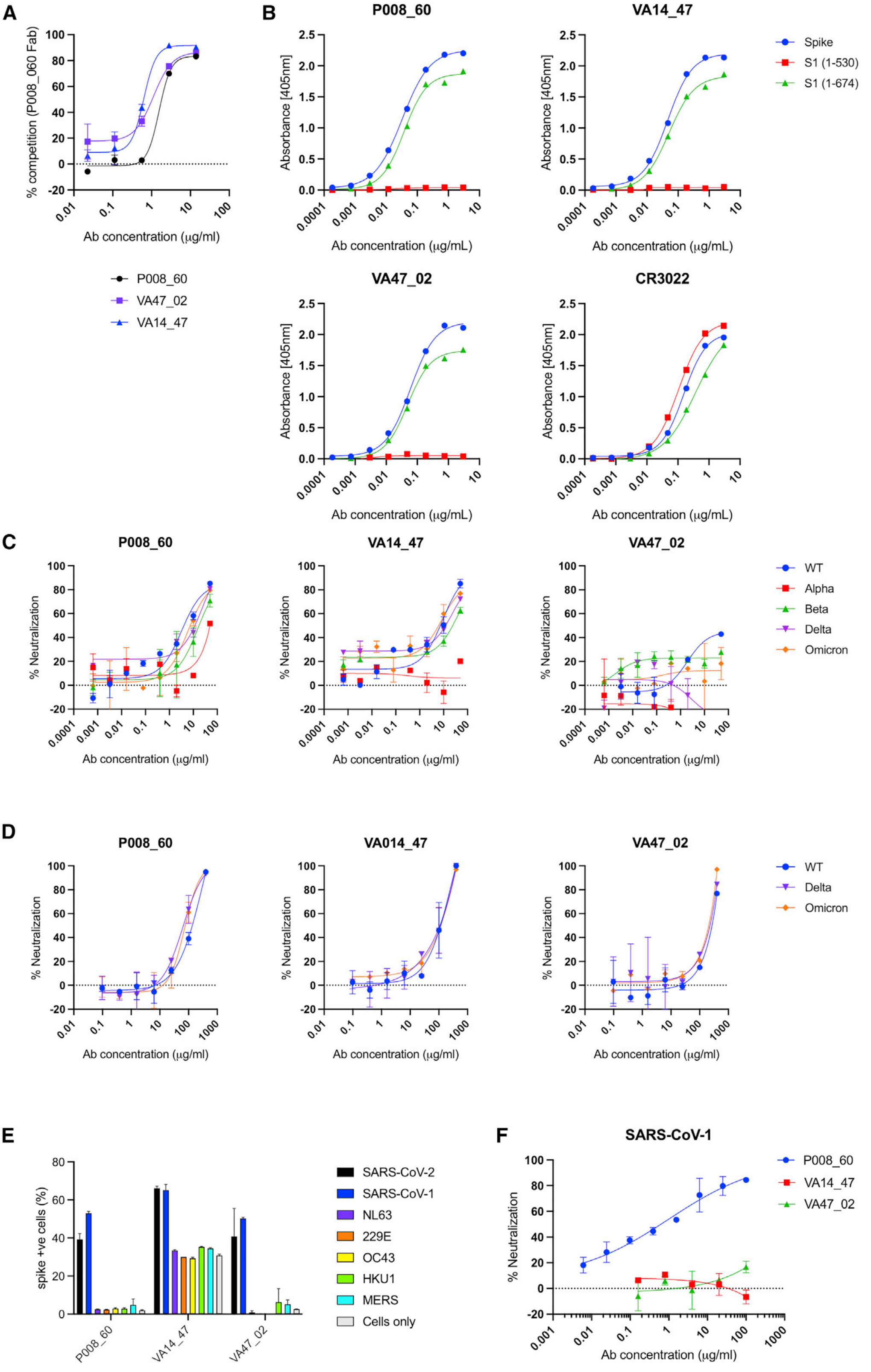

(legend on next page)

SARS-CoV-2 infection and COVID-19 vaccination is important for optimizing SARS-CoV-2 immunogens and antibody-based therapeutics, particularly in the face of emerging SARS-CoV-2 variants of concern (VOCs) carrying multiple mutations in spike.

Here, we identify a class of neutralizing antibodies that recognize the SD1 domain of the S1 subunit of SARS-CoV-2 and SARS-CoV-1 that have arisen following both SARS-CoV-2 infection and COVID-19 vaccination. Using cryo-electron microscopy (cryo-EM) and hydrogen-deuterium exchange coupled to mass spectrometry (HDX-MS), we identify a conserved neutralizing epitope on SD1 that is occluded on many SARS-CoV-2 spike structures. This study suggests that SD1 neutralizing antibodies engage a thus-far uncharacterized conformational state of the viral glycoprotein, which is sensitive to neutralization.

RESULTS

SD1 domain is a target for neutralizing antibodies

We have previously isolated three neutralizing monoclonal antibodies (mAbs) that bind to spike but not to RBD, NTD, or S2 (Graham et al., 2021; Seow et al., 2022). P008_60 was isolated from a convalescent donor infected in March 2020 (Graham et al., 2021), VA47_02 was isolated from a convalescent donor who was subsequently vaccinated with BNT162b2, and VA14_47 from an AZD1222-vaccinated individual (Seow et al., 2022). Competition ELISA revealed that these three mAbs compete for binding to spike (Figure 1A). To gain further insight into the epitope targeted by these mAbs, we measured their binding to S1(1–530), a construct lacking the SD1 and SD2 domains, and full-length S1, spanning residues 1–674 of the spike. All three mAbs bound strongly (Figure 1B) to the full-length, but not the truncated, construct, indicating the presence of a previously unidentified neutralizing epitope within the C-terminal portion of S1.

This class of mAbs displayed modest neutralization activity against HIV-1 viral particles pseudotyped with Wuhan-1 (wild-type [WT]) spike (half maximal inhibitory concentration [IC_{50}] 0.7–16.4 μg/mL) (Figure 1C) and against live virus (Figure 1D). In general, low neutralization plateaus and shallow neutralization curves were observed, suggesting incomplete neutralization (Graham et al., 2021). Neutralization against HIV-1 viral particles pseudotyped with current SARS-CoV-2 VOCs, including B.1.1.7 (Alpha), B.1.351 (Beta), B.1.617.2 (Delta), and BA.1 (Omicron) was reduced, but not completely abrogated, for P008_60 and VA14_47, whereas VA47_02 was unable to neutralize these VOCs. Cross-neutralization against Delta and Omicron (BA.1) was also detected using live virus (Figure 1D). Despite the low neutralization of Delta and Beta VOCs, all three mAbs were able to bind the recombinant VOC spikes in ELISA (Figure S1A).

All three neutralizing mAbs targeted to the C-terminal portion of S1 bound SARS-CoV-2 and SARS-CoV-1 spike expressed on the surface of HEK 293T cells (Figure 1E) but did not bind spike of other human coronaviruses (HCoVs) including 229E, NL63, OC43, HKU1, or Middle Eastern respiratory syndrome (MERS) (Figure 1E). Only P008_60 was able to neutralize SARS-CoV-1 pseudotyped viral particles (Figure 1F), indicating that binding is not sufficient for neutralization. Interestingly, VA14_47 also bound to non-transfected HEK 293T cells (Figure 1E).

Mode of P008_60 binding to SARS-CoV-2 spike glycoprotein

To structurally characterize the epitope of a neutralizing antibody targeting the C terminus of S1, we imaged single particles of the SARS-CoV-2 spike ectodomain in the presence of excess P008_60 Fab by cryo-EM (Figure S2A). Neither two-dimensional (2D) class averages nor 3D reconstructions of the trimeric spike suggested the presence of an associated Fab moiety (Figures S2B and S2C). By contrast, classification of spike monomers, also present in recombinant preparations of the viral glycoprotein construct (Rosa et al., 2021), yielded 2D class averages revealing features consistent with a bound Fab molecule (Figure S3A). Further image classification allowed us to reconstruct a 3D volume of the spike monomer with one molecule of Fab at 4.3 Å resolution (Figures S3B–S3F). Almost the entire S1 polypeptide, spanning residues 14–697 comprising the NTD, RBD, SD1, and SD2, could be docked into the cryo-EM map. The structure revealed that the Fab is bound to the SD1 (Figure 2A), making extensive contacts with the L3 loop of the subdomain (Figures 2B and S4A). While the resolution of the reconstruction was not sufficient to describe atomic details of the antibody-epitope interaction, the recognition of SD1 is almost exclusively mediated by the heavy chain of the mAb. The tyrosine-rich complementarity determining region (CDR) loops 1, 2, and 3 of P008_60 pinch the SD1 L3 loop (Figure 2B). The extended CDR3 makes extensive interactions with SD1 loop L5, reaching out to the glycan attached to Asn331, while CDR1 and CDR2 make contacts with SD1 L4. Approximately 950 Å^2 of molecular surface is buried upon formation of the SD1-Fab P008_60 complex, which corresponds to ~50% of average buried area for characterized epitope-paratope interfaces (Ramaraj et al., 2012).

Comparison of the S1-Fab P008_60 structure with the S1-ACE2 complex (Benton et al., 2020) reveals a small perturbation

Figure 1. P008_60, VA14_47, and VA47_02 bind an epitope on the SD1 domain of SARS-CoV-2 spike

(A) Competition spike ELISA between P008_60 F(ab')$_2$ and IgG of P008_60, VA14_47, and VA47_2 shows that the three mAbs bind an overlapping epitope.

(B) ELISA showing IgG binding to spike and S1 containing the SD1 domain (residues 1–674) but not to S1 (residues 1–530). SARS-CoV-1 RBD-specific mAb, CR3022, is used as a binding control.

(C) Neutralization of HIV-1 viral particles pseudotyped with Wuhan-1 (wild-type), Alpha, Beta, Delta, and Omicron/BA.1 spikes by SD1 mAbs.

(D) Neutralization of authentic SARS-CoV-2 virus (including Wuhan-1, Delta, and Omicron/BA.1) by SD1 mAbs. Data represent the average of two independent experiments performed in singlet.

(E) Binding of SD1 mAbs to SARS-CoV-2, SARS-CoV-1, NL63, 229E, OC43, HKU1, and MERS spikes expressed on the surface of HEK 293T cells.

(F) Neutralization of SARS-CoV-1 pseudotyped particles by SD1 mAbs.

Unless otherwise stated, experiments were performed in duplicate and performed at least twice. Representative datasets are shown. Error bars represent the range of the values for experiments performed in duplicate (not shown when smaller than symbol size).

See also Figure S1.

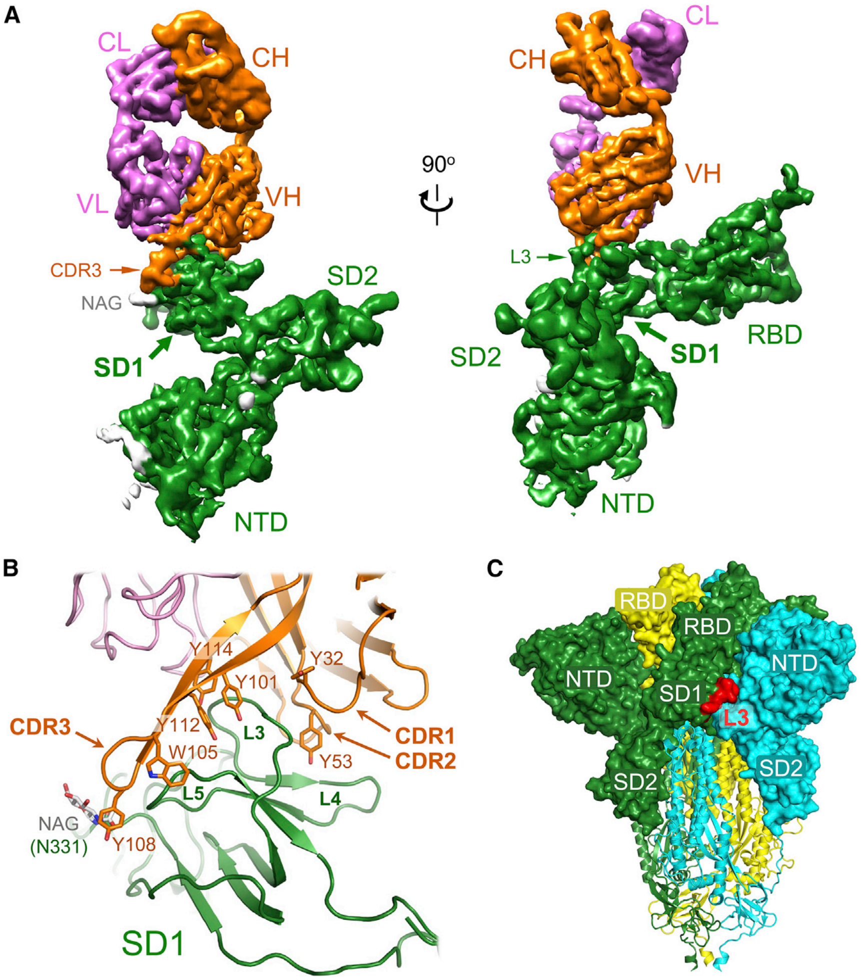

Figure 2. Characterization of the SD1 epitope recognized by P008_60

(A) Cryo-EM map of the S1-P008_60 Fab complex viewed from two orthogonal directions. The 3D reconstruction is colored by subunit: S1 protein is green (with sugars in gray); the antibody light and heavy chains are pink and orange, respectively. Protein domains are indicated: NTD, RBD, SD1, and SD2 are the spike N-terminal domain, receptor binding domain, subdomain 1, and subdomain 2, respectively; CL and CH are the constant regions of light and heavy chains; VL and VH are variable portions of light and heavy chains. NAG, CDR3, and L3 are N-Acetylglucosamine, complementarity determining region 3 loop, and the SD1 loop 3, respectively.

(B) Closeup view of the S1-P008_60 Fab interface with protein chains as cartoons. Selected side chains are shown as sticks and indicated.

(C) Trimeric SARS-CoV-2 spike ectodomain (PDB: 6ZGE) (Wrobel et al., 2020) with the three individual protein chains subunits color coded. The P008_60 epitope (L3) is highlighted in red. The S1 and S2 subunits are shown as surface and cartoons, respectively.

See also Figures S2–S4 and Table S1.

within the SD1, mostly localized to the L3 loop (Figure S4B), but no global conformational changes in S1 upon P008_60 binding (Figure S4C). Crucially, the structures do not suggest a competition between ACE2 and P008_60 for binding to monomeric S1. Although L3 loop is partially surface exposed on the structure of the spike trimer (Figure 2C), the epitope is not available for the interaction with the antibody, and the modeling predicts extensive steric clashes between P008_60 and the NTD of the neighboring S1 protomer, within closed (all RBDs down) or open (all RBDs up) trimer conformations (Figures S4D and S4E). This observation is fully consistent with the absence of the spike trimer-Fab P008_60 complexes on cryo-EM grids.

Both P008_60 and VA14_47 use the VH3-30 and VK3-20 germline genes, but they differ in their CDRH3 length (14 and 22 amino acids, respectively) (Figure S5A). The cryo-EM analysis indicates that the predominant antigen interactions are facilitated through tyrosine residues that are germline encoded or introduced through somatic hypermutation in the CDRH1, 2 and 3 regions of P008_60. VA14_47 has similar tyrosine residues in the CDRH1 and 2 regions, which presumably facilitate spike recognition. Although the CDRH3 of VA14_47 is shorter than P008_60, both are enriched in tyrosine residues (Figure S5B). The cross-neutralizing activity of P008_60 with SARS-CoV-1 is likely mediated by the SD1 contact residues R577 and E583 that are conserved between SARS-CoV-1 and SARS-CoV-2 spikes and the positively charged contact residue at position 568 (R and K, respectively). Indeed, mutation of R577 to alanine resulted in reduced binding of P008_60 to SARS-CoV-1 spike (Figure S1B) and loss of SARS-CoV-1 neutralizing activity (Figure S1C). Direct interactions with the protein backbone occur at positions where the amino acid sequence varies between SARS-CoV-1 and SARS-CoV-2.

HDX-MS reveals conformational impact of the SD1 mAb P008_60 on spike protein

To gain insights into the conformational impact of the SD1-binding neutralizing mAbs on spike and their neutralization mechanism, we conducted HDX-MS analysis on the spike alone and in complex with P008_60 immunoglobulin G (IgG). The combination of cryo-EM and HDX-MS has previously shown to be a powerful tool to describe the high-resolution structure of antigen-antibody assemblies and the conformational effects and allostery upon binding (Engen and Komives, 2020). The batch of recombinant spike protein used for HDX-MS was the same as that utilized for cryo-EM analysis. We carried out a differential temperature labeling experiment, expanding the HDX time window studied (ranging from 2 s to 100 min) to capture conformational events on a wide timescale (Coales et al., 2010; Goswami et al., 2013). We followed 321 peptides, spanning 81.5% of protein sequence (including 5 glycosylation sites) (Figure S6; Table S2), and overlapping peptides were used to spatially

CellPress
OPEN ACCESS

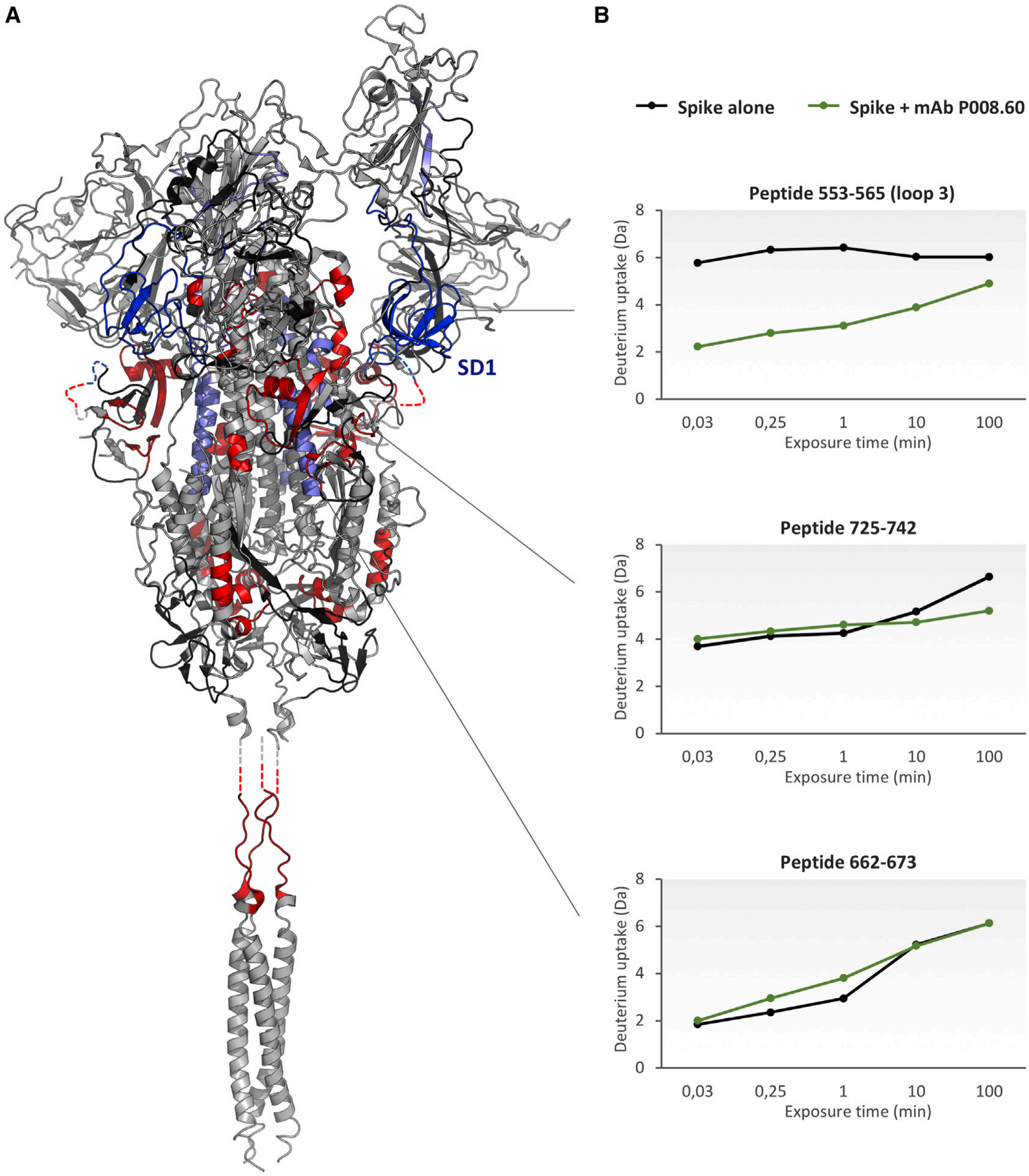

Figure 3. Conformational impact of the mAb P008_60 on the structure of spike protein

(A) Regions showing HDX effects are colored on the structure of spike glycoprotein with one RBD erected (PDB: 7BNN) and on the structure of the SARS-CoV-2 HR2 domain (PDB: 2FXP). Blue: decrease in HDX in the epitope and SD1 subdomain. Light blue: decrease in HDX caused by allosteric stabilization. Red: increase in HDX due to allosteric destabilization. Gray: no HDX effect. Black: no information. Protein segments not mapped in the structures are added as dashed lines.
(B) Examples of differential deuterium uptake plots. From top to bottom: a peptide spanning the epitope, a peptide manifesting allosteric stabilization upon binding, and a peptide manifesting destabilization upon binding. HDX-MS data supporting these findings (table containing deuterium uptake values and uptake plots) are available in Data S1 and S2.
See also Figures S4–S8 and Table S2.

resolve HDX effects (Figure S7). The conformational effects induced by the association to P008_60 were clearly visible across several spike regions, spanning both the S1 and S2 subunits (Figures 3, S7, and S8).

In detail, a pronounced decrease in HDX was seen in the region spanning residues 516–585, corresponding to the whole SD1 subdomain and including the epitope identified by cryo-EM (loops L3, L4, and L5). In this region, up to 5 Da of difference in HDX between the apo and bound states was observed and at several time points, indicating a stable association. Due to the presence of *N*-glycans, peptides encompassing Asn331 and Asn343 could not be identified (see STAR Methods). However,

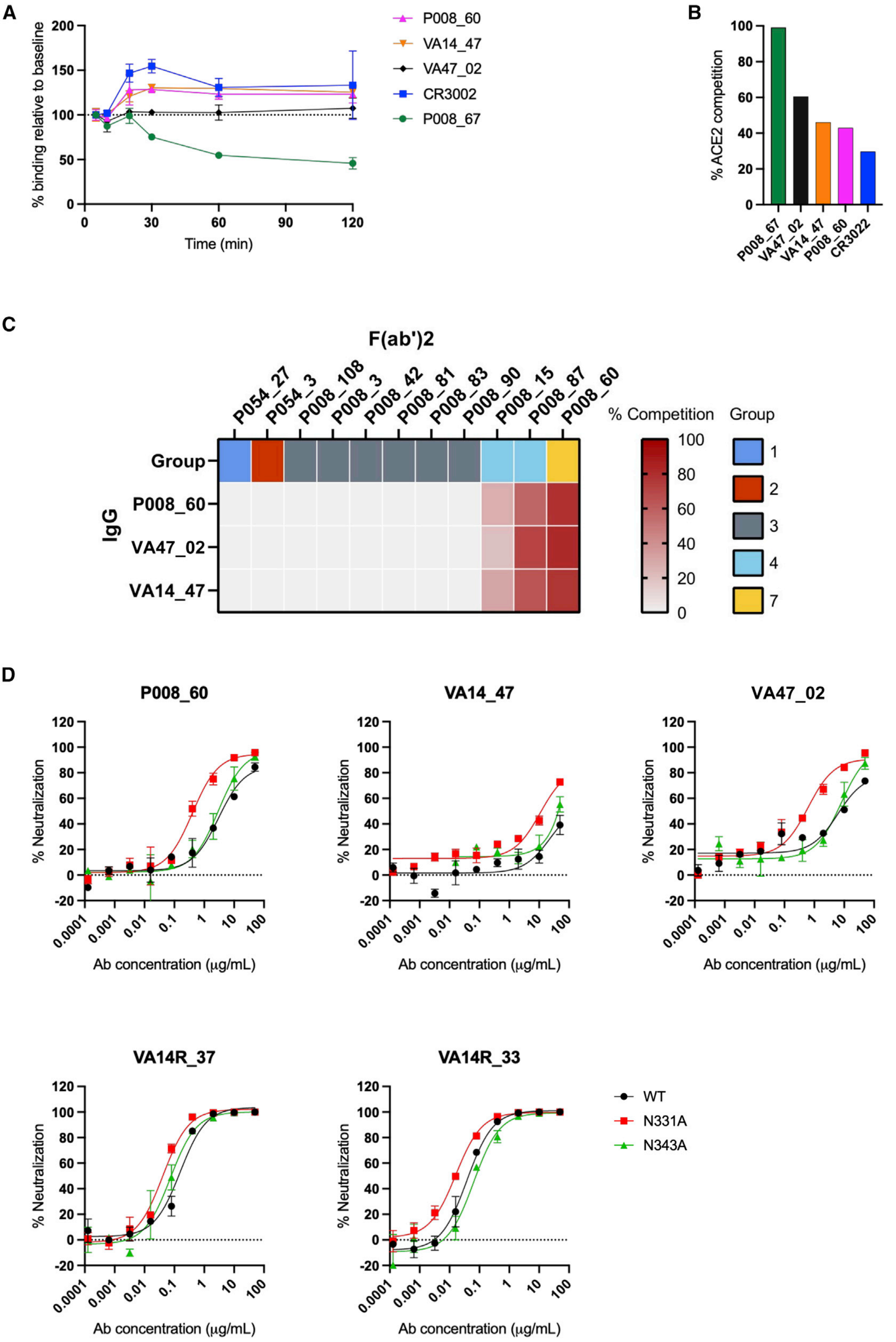

(legend on next page)

we were able to confirm that the *O*-glycans at Thr323 and Ser325, which are positioned very close to the S1 subunit, do not shield the P008_60 epitope, as a non-glycosylated copy of a peptide encompassing this region was detected and did not show any difference in HDX (Figures S6 and S7).

Transient and less pronounced decrease in HDX, a sign of potential allosteric effects of P008_60 mAb binding, was detected within the RBD (residues 348–361), the SD2 (residues 621–626), and S2 (residues 732–742 and 764–782) (Figures 3 and S7). In addition, P008_60 mAbs also induced widespread increase in HDX at regions scattered through the spike structure. Indicative of structural destabilization, increased HDX was observed in several segments along to the S2, including the region abutting the FP (residues 834–851 and 903–916) and HR1 (residues 923–931, 956-951, and 962–981), as well as the linker region of the HR2 (residues 962–981) (Figures 3 and S7), all involved in the transition to the post-fusion conformation (Wrapp et al., 2020). A decrease in HDX along the SD2 segment 621–626 may indicate transient stabilization of the RBD-up conformation (Zhang et al., 2021). Moreover, the observed higher flexibility (increased HDX) of residues 834–851 seems consistent with disruption of the salt bridge between Asp614 and Lys854, known to promote the transition to the RBD-up conformation (Benton et al., 2021; Zhang et al., 2021).

SD1 neutralizing antibody binding does not cause S1 shedding or compete with ACE2 binding

To further understand the mechanism by which this class of antibody neutralizes SARS-CoV-2, we measured the ability of mAbs to mediate S1 shedding from SARS-CoV-2 WT spike expressed on the surface of HEK 293T cells using flow cytometry. RBD mAbs P008_67 and CR3022 were used as positive and negative controls, respectively (Graham et al., 2021; Hurlburt et al., 2020). As expected, prolonged incubation with P008_67 resulted in progressive loss of antibody retention, which is consistent with induction of S1 shedding by this antibody. By contrast, P008_60 and CR3022 remained stably bound to the cells even after prolonged incubation, indicating that these antibodies do not induce S1 shedding in these conditions (Figure 4A).

Next, because our HDX-MS analysis suggested that P008_60 might stabilize the RBD-up conformation, we tested if the SD1 mAbs compete with the RBD-directed neutralizing antibodies, which bind the spike in the RBD-down conformation (P008_15 and P008_87). Reduced binding of this class of mAbs in the presence of SD1 mAbs supported the HDX-MS observation that P008_60 binding stabilizes the RBD-up conformation (Figure 4C). Further, we assessed competition with RBD neutralizing mAbs that recognize the ACE2 RBM (P008_108 and P008_042) and with ACE2, both binding to spike in RBD-up conformation (Graham et al., 2021). Expectedly, no competition was highlighted with RBM-targeting neutralizing mAbs (Figure 4C). By contrast with P008_67, which efficiently competed with ACE2 for the spike, P008_60 and CR3022 only weakly inhibited ACE2 binding (Figure 4B).

Glycan at position N331 partly occludes the SD1 epitope

Previous characterization of P008_60 showed that neutralization potency was sensitive to changes in the *N*-linked glycosylation of spike (Doores and Burton, 2010; Graham et al., 2021). In particular, neutralization potency was enhanced when pseudoviral particles were prepared in the presence of the glycosidase inhibitor swainsonine. As the compound leads to the spike being decorated in *N*-linked glycans that are smaller in size (Doores and Burton, 2010), this observation suggested larger glycans might restrict epitope accessibility. Indeed, our cryo-EM structure revealed that the *N*-linked glycan at position N331 is located in the immediate vicinity of the P008_60 epitope (Figure 2B). Therefore, to test the hypothesis that the glycan might partially occlude the SD1 neutralizing epitope, the neutralization potency of SD1 neutralizing mAbs was measured against SARS-CoV-2 pseudotyped particles containing the N331A or N343A substitution mutations to delete the *N*-linked glycan (Figure 4D). Deletion of the N331 glycan enhanced the neutralization potency of all three mAbs, supporting the hypothesis that N331 may partly occlude accessibility of the SD1 neutralizing epitope. By contrast, removal of the N343 glycan, located on the RBD and remote from the epitope, resulted in a slightly reduced neutralization potency. RBD specific mAbs VA14R_37 and VA14_33 showed minimal changes in neutralization potency against the glycan modified viruses.

DISCUSSION

Here, we demonstrate that the SD1 domain of SARS-CoV-2 is a target for neutralizing antibodies elicited following infection with ancestral SARS-CoV-2 (Graham et al., 2021) or following COVID-19 vaccination (AZD1222) (Seow et al., 2022). Two antibodies targeting SD1 have been previously reported (Tanaka et al.,

Figure 4. Mechanism of neutralization by SD1 mAbs

(A) Antibody mediated shedding of S1 from SARS-CoV-2 spike. HEK 293T cells expressing Wuhan-1 spike were incubated with SD1 mAbs binding measured by flow cytometry at 5, 10, 20, 30, and 60 min. mAb P008_67 was used as a positive control, and CR3022 was used as a negative control (Hurlburt et al., 2020). The spike protein did not contain the furin site mutation.

(B) Ability of mAbs to inhibit the interaction between cell surface ACE2 and soluble SARS-CoV-2 spike. mAbs (at 600 nM) were pre-incubated with fluorescently labeled spike before addition to HeLa-ACE2 cells. The percentage reduction in mean fluorescence intensity is reported. Experiments were performed in duplicate.

(C) Competition between P008_60 and RBD mAbs for spike binding. Inhibition of IgG binding to SARS-CoV-2 spike by $F(ab)_2$'s fragments was measured. The percentage competition was calculated using the reduction in IgG binding in the presence of $F(ab')_2$ (at 100 molar excess of the IC_{80}) as a percentage of the maximum IgG binding in the absence of $F(ab')_2$. Competition groups are as described in Graham et al. (2021) and Seow et al. (2022).

(D) Neutralization of HIV-1 viral particles pseudotyped with Wuhan-1 spike or Wuhan-1 spike containing an N331A or N343A mutation by SD1 mAbs. RBD nAbs VA14R_33 and VA14R_37 were used as neutralization controls (Seow et al., 2022).

All experiments were performed in duplicate and performed at least twice. Representative datasets are shown. Error bars represent the range of the values for experiments performed in duplicate (not shown when smaller than symbol size).

2022), but these were isolated from an engineered mRNA scFv library. N-612-004, which shows partial neutralization of the Wuhan variant, interacts at spike amino acid residues 556–563 and 567-69. In contradiction to the predictions from the previous study (Tanaka et al., 2022), we show that SD1 neutralizing epitopes are accessible on SARS-CoV-2 spike such that neutralizing antibodies targeting this region are elicited during infection or vaccination.

The SD1 epitope defined by P008_60 binding is structurally occluded in the available structures of stabilized trimeric SARS-CoV-2 spike constructs (Figures S4D and S4E). Given the ability of P008_60 to bind the native spike exposed on cell surface (Figure 1E) and neutralize the virus (Figures 1C and 1D), we conclude that the SD1 antibodies engage a thus-far uncharacterized conformational state of the viral glycoprotein, which is sensitive to neutralization. Such an epitope may be more exposed *in vivo*, on the native spike, where the S1 and S2 subunits are not covalently bound, or, alternatively, become exposed during the fusion process, possibly upon shedding of one or two S1 subunits. Indeed, binding of P008_60 to partially dissociated spike, lacking one of the S1 subunits (Gobeil et al., 2021) is predicted to cause no steric clashes (Figure S4F). Hence, the antibody may enhance dissociation of spike trimers, which is supported by enhancements of HDX detected throughout the spike structure in the presence of P008_60. Upon binding of SD1 neutralizing antibodies, the spike becomes more flexible in several regions of the S2 (Figures S7 and S8), thus less stable. This may induce the trimer to dissociate entirely and/or to irreversibly undergo inactivation. Our HDX data are consistent with the mAb stabilizing the RBD-up conformation. Indeed, competition assays with mAbs targeting the RBD-down conformation supports this hypothesis. The RBD-up conformation induced by SD1 neutralizing mAbs could expose otherwise occluded epitopes, including those in the ACE2 RBM, and enhance neutralization by antibodies directed against this epitope in the context of polyclonal sera.

RBD-directed neutralizing antibodies have been shown to compose a large proportion of the neutralizing activity in sera from SARS-CoV-2-infected and vaccinated individuals (Greaney et al., 2021; Piccoli et al., 2020). Therefore, antibodies targeting the SD1 neutralizing epitope likely represent a minor component of circulating neutralizing antibodies and likely have minimal selective immune pressure on SARS-CoV-2 spike evolution. Indeed, current SARS-CoV-2 VOCs (Alpha, Beta, Delta, and Omicron/BA.1) do not encode spike mutations in the P008_60 epitope. Concordantly, despite their relatively modest potency, both P008_60 and VA14_47 retain neutralization against these VOCs. Moreover, the SD1 epitope is further conserved within the SARS-CoV-1 spike. However, the observation that only P008_60 can neutralize SARS-CoV-1, and not VA14_47 and VA47_02, indicates that binding to membrane-tethered spike is not sufficient for neutralization by all mAbs targeting this previously unidentified epitope. Whether mAbs with more potent neutralizing activity against the SD1 epitope can be identified from convalescent or vaccinated individuals or engineered using rationale approaches warrants further investigation. Our cryo-EM analysis revealed that the predominant P008_60 antigen interactions are facilitated through tyrosine residues that are germline encoded or introduced through somatic hypermutation in the CDRH1, 2 and 3 regions. Both P008_60 and VA14_47 use the VH3-30 and VK3-20 germline genes. However, VH3-30 has been reported to be enriched in SARS-CoV-2-specific mAbs in general (Graham et al., 2021; Robbiani et al., 2020; Seow et al., 2022), and these antibodies target a number of different epitopes on spike (Graham et al., 2021; Robbiani et al., 2020; Seow et al., 2022), indicating that VH3-30 can be promiscuous in epitope recognition.

In conclusion, we identified a conserved epitope on SD1 that is targeted by neutralizing antibodies arising from infection with the ancestral SARS-CoV-2 or COVID-19 vaccination.

Limitations of the study

The specific mechanism by which SD1-targeted antibodies neutralize SARS-CoV-2 has not been fully elucidated. Despite broad neutralization activity, the *in vivo* protective efficacy of this class of neutralizing antibodies needs to be determined using animal models.

STAR★METHODS

Detailed methods are provided in the online version of this paper and include the following:

- KEY RESOURCES TABLE
- RESOURCE AVAILABILITY
  - Lead contact
  - Materials availability
  - Data and code availability
- EXPERIMENTAL MODEL AND SUBJECT DETAILS
  - Bacterial strains and cell culture
- METHOD DETAILS
  - Monoclonal antibody isolation
  - Protein expression and purification
  - Fab cloning and expression
  - ELISA (spike, S1 (1-530), S1+SD1 (1-674))
  - IgG digestion to generate $F(ab')_2$
  - $F(ab')_2$ and IgG competition ELISA
  - SARS-CoV-2 and SARS-CoV-1 pseudotyped virus preparation
  - Neutralization assay with SARS-CoV-2 and SARS-CoV-1 pseudotyped virus
  - SARS-CoV-2 microneutralization assay
  - Monoclonal antibody binding to spike using flow cytometry
  - ACE2 competition measured by flow cytometry
  - S1 shedding assay
  - Cryo-EM data collection, image processing and structure refinement
  - Hydrogen-deuterium exchange, LC-MS and data analysis
- QUANTIFICATION AND STATISTICAL ANALYSIS

SUPPLEMENTAL INFORMATION

Supplemental information can be found online at https://doi.org/10.1016/j.celrep.2022.111276.

CellPress
OPEN ACCESS

ACKNOWLEDGMENTS

We thank Wendy Barclay for providing the B.1.1.7., B.1.351, B.1.617.2, and Omicron/BA.1 spike plasmids and James Voss and Deli Huang for providing the Hela-ACE2 cells. This work was funded by a Huo Family Foundation Award to M.H.M. and K.J.D.; MRC Genotype-to-Phenotype UK National Virology Consortium (MR/W005611/1 to M.H.M. and K.J.D.); Fondation Dormeur, Vaduz for funding equipment to K.J.D.; Wellcome Trust Awards (106223/Z/14/Z and 222433/Z/21/Z to M.H.M.); Wellcome Trust Multi-User Equipment Grant (208354/Z/17/Z to M.H.M. and K.J.D.); and the Department of Health via a National Institute for Health Research comprehensive Biomedical Research Centre award to Guy's and St. Thomas' NHS Foundation Trust in partnership with King's College London and King's College Hospital NHS Foundation Trust. C.G. was supported by the MRC-KCL Doctoral Training Partnership in Biomedical Sciences (MR/N013700/1). This study is part of the EDCTP2 program supported by the European Union (grant number RIA2020EF-3008 COVAB, to K.J.D. and M.H.M.). The views and opinions of authors expressed herein do not necessarily state or reflect those of EDCTP. This project is supported by a joint initiative between the Botnar Research Centre for Child Health and the European & Developing Countries Clinical Trials Partnership (K.J.D.). The P.C. laboratory is funded by the Francis Crick Institute (FC001061), which receives its core funding from the Cancer Research UK (FC001061), the UK Medical Research Council (FC001061), and the Wellcome Trust (FC001061). Cryo-EM data were acquired at the LonCEM Facility, which is funded by the Wellcome Trust (206175/Z/17/Z to Professor Xiaodong Zhang). HDX-MS is funded by the Leverhulme Trust (RPG-2019-178) to A.P. A.P. is also supported by an EPSRC Research Fellowship (EP/V011715/1). This research was funded in whole, or in part, by the Wellcome Trust 106223/Z/14/Z, 222433/Z/21/Z, and 208354/Z/17/Z). For the purpose of open access, the author has applied a CC BY public copyright license to any author accepted manuscript version arising from this submission.

AUTHOR CONTRIBUTIONS

J.S., C.G., I.H., S.P., and K.J.D. isolated and characterized the mAbs. H.K. performed mechanistic studies. A.R. prepared samples for cryo-EM; N.B.C. acquired cryo-EM data; P.C. processed cryo-EM data and generated the 3D volume; P.C. and V.E.P., refined and analyzed the atomistic model of the S1-Fab complex. V.C. designed, performed, and analyzed HDX-MS experiments. A.P. supervised the HDX-MS work. K.J.D. and M.H.M. supervised the work. K.J.D., P.C., V.C., and A.P. wrote the paper, and all authors commented on the manuscript.

DECLARATION OF INTERESTS

The authors declare no competing interests.

Received: March 28, 2022
Revised: July 25, 2022
Accepted: August 5, 2022
Published: August 11, 2022

REFERENCES

Afonine, P.V., Poon, B.K., Read, R.J., Sobolev, O.V., Terwilliger, T.C., Urzhumtsev, A., and Adams, P.D. (2018). Real-space refinement in PHENIX for cryo-EM and crystallography. Acta Crystallogr. D Struct. Biol. *74*, 531–544.

Aricescu, A.R., Lu, W., and Jones, E.Y. (2006). A time- and cost-efficient system for high-level protein production in mammalian cells. Acta Crystallogr. D Biol. Crystallogr. *62*, 1243–1250.

Barnes, C.O., Jette, C.A., Abernathy, M.E., Dam, K.M.A., Esswein, S.R., Gristick, H.B., Malyutin, A.G., Sharaf, N.G., Huey-Tubman, K.E., Lee, Y.E., et al. (2020). SARS-CoV-2 neutralizing antibody structures inform therapeutic strategies. Nature *588*, 682–687.

Benton, D.J., Wrobel, A.G., Roustan, C., Borg, A., Xu, P., Martin, S.R., Rosenthal, P.B., Skehel, J.J., and Gamblin, S.J. (2021). The effect of the D614G substitution on the structure of the spike glycoprotein of SARS-CoV-2. Proc. Natl. Acad. Sci. USA *118*, e2022586118.

Benton, D.J., Wrobel, A.G., Xu, P., Roustan, C., Martin, S.R., Rosenthal, P.B., Skehel, J.J., and Gamblin, S.J. (2020). Receptor binding and priming of the spike protein of SARS-CoV-2 for membrane fusion. Nature *588*, 327–330.

Brouwer, P.J.M., Caniels, T.G., van der Straten, K., Snitselaar, J.L., Aldon, Y., Bangaru, S., Torres, J.L., Okba, N.M.A., Claireaux, M., Kerster, G., et al. (2020). Potent neutralizing antibodies from COVID-19 patients define multiple targets of vulnerability. Science *369*, 643–650.

Carter, M.J., Fish, M., Jennings, A., Doores, K.J., Wellman, P., Seow, J., Acors, S., Graham, C., Timms, E., Kenny, J., et al. (2020). Peripheral immunophenotypes in children with multisystem inflammatory syndrome associated with SARS-CoV-2 infection. Nat. Med. *26*, 1701–1707.

Castro Dopico, X., Ols, S., Loré, K., and Karlsson Hedestam, G.B. (2022). Immunity to SARS-CoV-2 induced by infection or vaccination. J. Intern. Med. *291*, 32–50.

Cerutti, G., Guo, Y., Zhou, T., Gorman, J., Lee, M., Rapp, M., Reddem, E.R., Yu, J., Bahna, F., Bimela, J., et al. (2021). Potent SARS-CoV-2 neutralizing antibodies directed against spike N-terminal domain target a single supersite. Cell Host Microbe *29*, 819–833.e7.

Coales, S.J., E, S.Y., Lee, J.E., Ma, A., Morrow, J.A., and Hamuro, Y. (2010). Expansion of time window for mass spectrometric measurement of amide hydrogen/deuterium exchange reactions. Rapid Commun. Mass Spectrom. *24*, 3585–3592.

Corti, D., Purcell, L.A., Snell, G., and Veesler, D. (2021). Tackling COVID-19 with neutralizing monoclonal antibodies. Cell *184*, 4593–4595.

Dixon, E.V. (2014). Mechanisms of immunoglobulin deactivation by Streptococcus pyogenes. PhD Thesis.

Doores, K.J., and Burton, D.R. (2010). Variable loop glycan dependency of the broad and potent HIV-1-neutralizing antibodies PG9 and PG16. J. Virol. *84*, 10510–10521.

Emsley, P., and Cowtan, K. (2004). Coot: model-building tools for molecular graphics. Acta Crystallogr. D Biol. Crystallogr. *60*, 2126–2132.

Engen, J.R., and Komives, E.A. (2020). Complementarity of hydrogen/deuterium exchange mass spectrometry and cryo-electron microscopy. Trends Biochem. Sci. *45*, 906–918.

Gavor, E., Choong, Y.K., Er, S.Y., Sivaraman, H., and Sivaraman, J. (2020). Structural basis of SARS-CoV-2 and SARS-CoV antibody interactions. Trends Immunol. *41*, 1006–1022.

Gobeil, S.M.C., Janowska, K., McDowell, S., Mansouri, K., Parks, R., Stalls, V., Kopp, M.F., Manne, K., Li, D., Wiehe, K., et al. (2021). Effect of natural mutations of SARS-CoV-2 on spike structure, conformation, and antigenicity. Science *373*, eabi6226.

Goswami, D., Devarakonda, S., Chalmers, M.J., Pascal, B.D., Spiegelman, B.M., and Griffin, P.R. (2013). Time window expansion for HDX analysis of an intrinsically disordered protein. J. Am. Soc. Mass Spectrom. *24*, 1584–1592.

Graham, C., Seow, J., Huettner, I., Khan, H., Kouphou, N., Acors, S., Winstone, H., Pickering, S., Galao, R.P., Dupont, L., et al. (2021). Neutralization potency of monoclonal antibodies recognizing dominant and subdominant epitopes on SARS-CoV-2 Spike is impacted by the B.1.1.7 variant. Immunity *54*, 1276–1289.e6.

Greaney, A.J., Loes, A.N., Crawford, K.H.D., Starr, T.N., Malone, K.D., Chu, H.Y., and Bloom, J.D. (2021). Comprehensive mapping of mutations in the SARS-CoV-2 receptor-binding domain that affect recognition by polyclonal human plasma antibodies. Cell Host Microbe *29*, 463–476.e6.

Grehan, K., Ferrara, F., and Temperton, N. (2015). An optimised method for the production of MERS-CoV spike expressing viral pseudotypes. MethodsX *2*, 379–384.

Huang, Y., Yang, C., Xu, X.F., Xu, W., and Liu, S.W. (2020). Structural and functional properties of SARS-CoV-2 spike protein: potential antivirus drug development for COVID-19. Acta Pharmacol. Sin. *41*, 1141–1149.

Huo, J., Mikolajek, H., Le Bas, A., Clark, J.J., Sharma, P., Kipar, A., Dormon, J., Norman, C., Weckener, M., Clare, D.K., et al. (2021). A potent SARS-CoV-2 neutralising nanobody shows therapeutic efficacy in the Syrian golden hamster model of COVID-19. Nat. Commun. *12*, 5469.

Hurlburt, N.K., Seydoux, E., Wan, Y.H., Edara, V.V., Stuart, A.B., Feng, J., Suthar, M.S., McGuire, A.T., Stamatatos, L., and Pancera, M. (2020). Structural basis for potent neutralization of SARS-CoV-2 and role of antibody affinity maturation. Nat. Commun. *11*, 5413.

Hurt, A.C., and Wheatley, A.K. (2021). Neutralizing antibody therapeutics for COVID-19. Viruses *13*, 628.

Kidmose, R.T., Juhl, J., Nissen, P., Boesen, T., Karlsen, J.L., and Pedersen, B.P. (2019). Namdinator - automatic molecular dynamics flexible fitting of structural models into cryo-EM and crystallography experimental maps. IUCrJ *6*, 526–531.

Krammer, F. (2020). SARS-CoV-2 vaccines in development. Nature *586*, 516–527.

Lan, J., Ge, J., Yu, J., Shan, S., Zhou, H., Fan, S., Zhang, Q., Shi, X., Wang, Q., Zhang, L., and Wang, X. (2020). Structure of the SARS-CoV-2 spike receptor-binding domain bound to the ACE2 receptor. Nature *581*, 215–220.

Lau, A.M., Claesen, J., Hansen, K., and Politis, A. (2021). Deuteros 2.0: peptide-level significance testing of data from hydrogen deuterium exchange mass spectrometry. Bioinformatics *37*, 270–272.

Lechmere, T., Snell, L.B., Graham, C., Seow, J., Shalim, Z.A., Charalampous, T., Alcolea-Medina, A., Batra, R., Nebbia, G., Edgeworth, J.D., et al. (2022). Broad neutralization of SARS-CoV-2 variants, including omicron, following breakthrough infection with delta in COVID-19-vaccinated individuals. mBio *13*, e0379821.

Masson, G.R., Burke, J.E., Ahn, N.G., Anand, G.S., Borchers, C., Brier, S., Bou-Assaf, G.M., Engen, J.R., Englander, S.W., Faber, J., et al. (2019). Recommendations for performing, interpreting and reporting hydrogen deuterium exchange mass spectrometry (HDX-MS) experiments. Nat. Methods *16*, 595–602.

McCallum, M., Marco, A., Lempp, F., Tortorici, M.A., Pinto, D., Walls, A.C., Beltramello, M., Chen, A., Liu, Z., Zatta, F., et al. (2021). N-terminal domain antigenic mapping reveals a site of vulnerability for SARS-CoV-2. Cell. https://doi.org/10.1016/j.cell.2021.03.028.

Min, X., Weiszmann, J., Johnstone, S., Wang, W., Yu, X., Romanow, W., Thibault, S., Li, Y., and Wang, Z. (2018). Agonistic beta-Klotho antibody mimics fibroblast growth factor 21 (FGF21) functions. J. Biol. Chem. *293*, 14678–14688.

Monin, L., Laing, A.G., Muñoz-Ruiz, M., McKenzie, D.R., Del Molino Del Barrio, I., Alaguthurai, T., Domingo-Vila, C., Hayday, T.S., Graham, C., Seow, J., et al. (2021). Safety and immunogenicity of one versus two doses of the COVID-19 vaccine BNT162b2 for patients with cancer: interim analysis of a prospective observational study. Lancet Oncol. *22*, 765–778.

Pettersen, E.F., Goddard, T.D., Huang, C.C., Couch, G.S., Greenblatt, D.M., Meng, E.C., and Ferrin, T.E. (2004). UCSF Chimera–a visualization system for exploratory research and analysis. J. Comput. Chem. *25*, 1605–1612.

Piccoli, L., Park, Y.J., Tortorici, M.A., Czudnochowski, N., Walls, A.C., Beltramello, M., Silacci-Fregni, C., Pinto, D., Rosen, L.E., Bowen, J.E., et al. (2020). Mapping neutralizing and immunodominant sites on the SARS-CoV-2 spike receptor-binding domain by structure-guided high-resolution serology. Cell *183*, 1024–1042.e21.

Pickering, S., Betancor, G., Galão, R.P., Merrick, B., Signell, A.W., Wilson, H.D., Kia Ik, M.T., Seow, J., Graham, C., Acors, S., et al. (2020). Comparative assessment of multiple COVID-19 serological technologies supports continued evaluation of point-of-care lateral flow assays in hospital and community healthcare settings. PLoS Pathog. *16*, e1008817.

Punjani, A., Rubinstein, J.L., Fleet, D.J., and Brubaker, M.A. (2017). cryoSPARC: algorithms for rapid unsupervised cryo-EM structure determination. Nat. Methods *14*, 290–296.

Ramaraj, T., Angel, T., Dratz, E.A., Jesaitis, A.J., and Mumey, B. (2012). Antigen-antibody interface properties: composition, residue interactions, and features of 53 non-redundant structures. Biochim. Biophys. Acta *1824*, 520–532.

Robbiani, D.F., Gaebler, C., Muecksch, F., Lorenzi, J.C.C., Wang, Z., Cho, A., Agudelo, M., Barnes, C.O., Gazumyan, A., Finkin, S., et al. (2020). Convergent antibody responses to SARS-CoV-2 in convalescent individuals. Nature *584*, 437–442.

Rogers, T.F., Zhao, F., Huang, D., Beutler, N., Burns, A., He, W.T., Limbo, O., Smith, C., Song, G., Woehl, J., et al. (2020). Isolation of potent SARS-CoV-2 neutralizing antibodies and protection from disease in a small animal model. Science *369*, 956–963.

Rosa, A., Pye, V.E., Graham, C., Muir, L., Seow, J., Ng, K.W., Cook, N.J., Rees-Spear, C., Parker, E., Dos Santos, M.S., et al. (2021). SARS-CoV-2 can recruit a haem metabolite to evade antibody immunity. Sci. Adv. *7*, eabg7607.

Rosenthal, P.B., and Henderson, R. (2003). Optimal determination of particle orientation, absolute hand, and contrast loss in single-particle electron cryomicroscopy. J. Mol. Biol. *333*, 721–745.

Sanchez-Garcia, R., Gomez-Blanco, J., Cuervo, A., Carazo, J.M., Sorzano, C.O.S., and Vargas, J. (2021). DeepEMhancer: a deep learning solution for cryo-EM volume post-processing. Commun. Biol. *4*, 874.

Scheres, S.H.W., and Chen, S. (2012). Prevention of overfitting in cryo-EM structure determination. Nat. Methods *9*, 853–854.

Scheres, S.H.W. (2020). Amyloid structure determination in RELION-3.1. Acta Crystallogr. D Struct. Biol. *76*, 94–101.

Seow, J., Graham, C., Hallett, S.R., Lechmere, T., Maguire, T.J.A., Huettner, I., Cox, D., Khan, H., Pickering, S., Roberts, R., et al. (2022). ChAdOx1 nCoV-19 vaccine elicits monoclonal antibodies with cross-neutralizing activity against SARS-CoV-2 viral variants. Cell Rep. *39*, 110757.

Seow, J., Graham, C., Merrick, B., Acors, S., Pickering, S., Steel, K.J.A., Hemmings, O., O'Byrne, A., Kouphou, N., Galao, R.P., et al. (2020). Longitudinal observation and decline of neutralizing antibody responses in the three months following SARS-CoV-2 infection in humans. Nat. Microbiol. *5*, 1598–1607.

Tan, Y.Z., Baldwin, P.R., Davis, J.H., Williamson, J.R., Potter, C.S., Carragher, B., and Lyumkis, D. (2017). Addressing preferred specimen orientation in single-particle cryo-EM through tilting. Nat. Methods *14*, 793–796.

Tanaka, S., Olson, C.A., Barnes, C.O., Higashide, W., Gonzalez, M., Taft, J., Richardson, A., Martin-Fernandez, M., Bogunovic, D., Gnanapragasam, P.N.P., et al. (2022). Rapid identification of neutralizing antibodies against SARS-CoV-2 variants by mRNA display. Cell Rep. *38*, 110348.

Tay, M.Z., Poh, C.M., Rénia, L., MacAry, P.A., and Ng, L.F.P. (2020). The trinity of COVID-19: immunity, inflammation and intervention. Nat. Rev. Immunol. *20*, 363–374.

Taylor, P.C., Adams, A.C., Hufford, M.M., de la Torre, I., Winthrop, K., and Gottlieb, R.L. (2021). Neutralizing monoclonal antibodies for treatment of COVID-19. Nat. Rev. Immunol. *21*, 382–393.

ter Meulen, J., van den Brink, E.N., Poon, L.L.M., Marissen, W.E., Leung, C.S.W., Cox, F., Cheung, C.Y., Bakker, A.Q., Bogaards, J.A., van Deventer, E., et al. (2006). Human monoclonal antibody combination against SARS coronavirus: synergy and coverage of escape mutants. PLoS Med. *3*, e237.

Terwilliger, T.C., Ludtke, S.J., Read, R.J., Adams, P.D., and Afonine, P.V. (2020). Improvement of cryo-EM maps by density modification. Nat. Methods *17*, 923–927.

Thompson, C.P., Grayson, N.E., Paton, R.S., Bolton, J.S., Lourenço, J., Penman, B.S., Lee, L.N., Odon, V., Mongkolsapaya, J., Chinnakannan, S., et al. (2020). Detection of neutralising antibodies to SARS-CoV-2 to determine population exposure in Scottish blood donors between March and May 2020. Euro Surveill. *25*.

van den Brink, E.N., Ter Meulen, J., Cox, F., Jongeneelen, M.A.C., Thijsse, A., Throsby, M., Marissen, W.E., Rood, P.M.L., Bakker, A.B.H., Gelderblom, H.R., et al. (2005). Molecular and biological characterization of human monoclonal antibodies binding to the spike and nucleocapsid proteins of severe acute respiratory syndrome coronavirus. J. Virol. *79*, 1635–1644.

CellPress
OPEN ACCESS

Williams, C.J., Headd, J.J., Moriarty, N.W., Prisant, M.G., Videau, L.L., Deis, L.N., Verma, V., Keedy, D.A., Hintze, B.J., Chen, V.B., et al. (2018). MolProbity: more and better reference data for improved all-atom structure validation. Protein Sci. *27*, 293–315.

Winstone, H., Lista, M.J., Reid, A.C., Bouton, C., Pickering, S., Galao, R.P., Kerridge, C., Doores, K.J., Swanson, C., and Neil, S. (2021). The polybasic cleavage site in the SARS-CoV-2 spike modulates viral sensitivity to Type I interferon and IFITM2. J. Virol. *95*, e02422-20.

Wrapp, D., Wang, N., Corbett, K.S., Goldsmith, J.A., Hsieh, C.L., Abiona, O., Graham, B.S., and McLellan, J.S. (2020). Cryo-EM structure of the 2019-nCoV spike in the prefusion conformation. Science *367*, 1260–1263.

Wrobel, A.G., Benton, D.J., Xu, P., Roustan, C., Martin, S.R., Rosenthal, P.B., Skehel, J.J., and Gamblin, S.J. (2020). SARS-CoV-2 and bat RaTG13 spike glycoprotein structures inform on virus evolution and furin-cleavage effects. Nat. Struct. Mol. Biol. *27*, 763–767.

Yuan, M., Liu, H., Wu, N.C., and Wilson, I.A. (2021). Recognition of the SARS-CoV-2 receptor binding domain by neutralizing antibodies. Biochem. Biophys. Res. Commun. *538*, 192–203.

Zhang, J., Cai, Y., Xiao, T., Lu, J., Peng, H., Sterling, S.M., Walsh, R.M., Jr., Rits-Volloch, S., Zhu, H., Woosley, A.N., et al. (2021). Structural impact on SARS-CoV-2 spike protein by D614G substitution. Science *372*, 525–530.

Zhang, K. (2016). Gctf: real-time CTF determination and correction. J. Struct. Biol. *193*, 1–12.

Zheng, S.Q., Palovcak, E., Armache, J.P., Verba, K.A., Cheng, Y., and Agard, D.A. (2017). MotionCor2: anisotropic correction of beam-induced motion for improved cryo-electron microscopy. Nat. Methods *14*, 331–332.

Zivanov, J., Nakane, T., and Scheres, S.H.W. (2019). A Bayesian approach to beam-induced motion correction in cryo-EM single-particle analysis. IUCrJ *6*, 5–17.

Zufferey, R., Nagy, D., Mandel, R.J., Naldini, L., and Trono, D. (1997). Multiply attenuated lentiviral vector achieves efficient gene delivery in vivo. Nat. Biotechnol. *15*, 871–875.

STAR★METHODS

KEY RESOURCES TABLE

REAGENT or RESOURCE	SOURCE	IDENTIFIER
**Antibodies**		
Goat-anti-human-Fc-AP	Jackson	RRID: AB_2337608 Cat#:109-055-098
horse-anti-mouse-IgG-HRP	Sigma	Cat#: A2554
Mouse-anti-human IgG Fc-PE	Biolegend	RRID: AB_10895907 Cat#: 409304
Streptavidin-APC	Thermofisher Scientific	Cat#: S32362
Murinized mAb CR3009	This manuscript and (van den Brink et al., 2005)	N/A
mAb CR3022	This manuscript and (ter Meulen et al., 2006)	N/A
mAbs P008_060, VA14_47 and VA14_02	This manuscript and (Graham et al., 2021; Seow et al., 2022)	N/A
**Bacterial and virus strains**		
NEB® Stable Competent *E. coli*	New England Biolabs	Cat#: C3040H
SARS-CoV-2 Strain BA.1	From Wendy Barclay (Imperial) and grown at KCL	N/A
SARS-CoV-2 Strain delta	From Wendy Barclay (Imperial) and grown at KCL	N/A
SARS-CoV-2 Strain England 2 (England 02/2020/407073)	Public Health England (PHE) and grown at KCL	N/A
**Chemicals, peptides, and recombinant proteins**		
Polyethylenimine, Linear, MW 25000 (PEI Max)	Polysciences, Inc	Cat#: 23966
Polyethylenimine Hydrochloride, Linear, MW 4,000	Polysciences, Inc	Cat#: 24885
TransIT-2020	Mirus Bio	Cat#: MIR5406
Recombinant S1 Wuhan (residues 1–530)	Peter Cherepanov (Crick) (Rosa et al., 2021) and this manuscript	N/A
Recombinant S1 Wuhan (residues 1–674)	Native Antigen Company	Cat#: REC31806-100
Recombinant Stabilized SARS-CoV-2 Spike ectodomain trimer for CryoEM and HDX	Peter Cherepanov (Crick) (Wrobel et al., 2020)	N/A
Recombinant Stabilized SARS-CoV-2 Spike for ELISA (Wuhan, beta and delta)	Marit van Gils (Amsterdam) (Brouwer et al., 2020) and this paper.	N/A
Recombinant SARS-CoV-2 Spike (biotinylated)	(Graham et al., 2021; Seow et al., 2022)	N/A
IdeS	Max Crispin (University of Southampton) (Dixon, 2014)	N/A
Protein G agarose	GE Healthcare	Cat#: Cytiva 17-0618-02
HiTrap IMAC columns	GE Healthcare	Cat#: Cytiva 17-0921-04
HILOAD 16/600 SUPERDEX 200 PG	GE Healthcare	Cat#: 28989335
**Critical commercial assays**		
Q5® Site-Directed Mutagenesis Kit	New England Biolabs	Cat#: E0554
Bright-Glo luciferase kit	Promega	Cat#: E2610
LIVE/DEAD Fixable Aqua Dead Cell Stain Kit	Thermofisher Scientific	Cat#: L34957
TrueBlue peroxidase substrate	SeraCare	Cat#: 50-78-02
Phosphatase substrate	Sigma Aldrich	Cat#: S0942-200TAB

(*Continued on next page*)

CellPress
OPEN ACCESS

Continued

REAGENT or RESOURCE	SOURCE	IDENTIFIER
Deposited data		
CryoEM data (EMDB and PDB)	This manuscript	Accession codes EMDB: EMD-14591 and PDB: 7ZBU
Experimental models: Cell lines		
FreeStyle™ 293F Cells	Thermofisher Scientific	Cat#: R79007
HEK293T/17	ATCC	ATCC® CRL-11268™
HeLa-ACE2	James Voss (Scripps), (Rogers et al., 2020)	N/A
Vero-E6 TMPRSS2 cells	Stuart Neil (KCL)	N/A
HEK293T	ATCC	ATCC® CRL-3216™
Oligonucleotides		
SARS-CoV-1 Spike mutagenesis primers	This manuscript	N/A
Recombinant DNA		
Biotinylated Spike (pHLSec)	This manuscript and (Seow et al., 2022)	N/A
Pre-fusion, stabilized and uncleaved SARS-CoV-2 Spike (pcDNA3.1+) (WT, delta and beta)	Marit van Gils (Amsterdam) (Brouwer et al., 2020) and this paper	N/A
Truncated SARS-CoV-2 Wuhan Spike (pcDNA3.1+)	Wendy Barclay (Imperial)	N/A
Truncated B.1.1.7 (alpha) variant Spike (pcDNA3.1+)	Wendy Barclay (Imperial)	N/A
Truncated B.1.351 (beta) variant Spike (pcDNA3.1+)	Wendy Barclay (Imperial)	N/A
Truncated B.1.617.2 (delta) variant Spike (pcDNA3.1+)	Wendy Barclay (Imperial)	N/A
Truncated B.1.1.529 (omicron/BA.1) variant Spike (pcDNA3.1+)	Wendy Barclay (Imperial)	N/A
Full-length SARS-CoV-1 Spike (pcDNA3.1+)	This manuscript and (Winstone et al., 2021)	N/A
Full-length NL63 Spike (pcDNA3.1+)	This manuscript	N/A
Full-length 229E Spike (pcDNA3.1+)	This manuscript	N/A
Full-length OC43 Spike (pcDNA3.1+)	This manuscript	N/A
Full-length HKU1 Spike (pcDNA3.1+)	This manuscript	N/A
Full-length MERS Spike (pcDNA3.1+)	This manuscript	N/A
Full-length SARS-CoV-2 Spike (pcDNA3.1+)	(Seow et al., 2020)	N/A
pHIV-Luc (constructed by replacing GFP in pHR'SIN-SEW (PMID: 11975847) with HA-luciferase)	Luis Apolonia (KCL)	N/A
HIV 8.91 gag/pol packaging construct	p8.91 (Zufferey et al., 1997)	N/A
Stabilized trimeric Wuhan Spike ectodomain (pcDNA3.1+) used in cryo-EM experiments	Antoni Wrobel (Crick Isntitute) (Wrobel et al., 2020)	N/A
Software and algorithms		
FlowJo	Tree Star	https://www.flowjo.com
Prism	Graphpad	https://www.graphpad.com/scientific-software/prism/
IMGT/V-QUEST	IMGT	http://www.imgt.org/IMGT_vquest/vquest
AID EliSpot 8.0 software	Autoimmun Diagnostika GmbH	N/A
MotionCor2	(Zheng et al., 2017)	https://emcore.ucsf.edu/ucsf-software
Gautomatch	Kai Zhang, MRC Laboratory of Molecular Biology (Cambridge, UK)	http://www.mrc-lmb.cam.ac.uk/kzhang
Relion version 3.1	(Scheres, 2020)	https://relion.readthedocs.io/en/release-3.1/
cryoSPARC	(Punjani et al., 2017)	https://cryosparc.com/
3DFSC	(Tan et al., 2017)	https://github.com/LyumkisLab/3DFSC

(*Continued on next page*)

Continued

REAGENT or RESOURCE	SOURCE	IDENTIFIER
DeepEMhancer	(Sanchez-Garcia et al., 2021)	https://github.com/rsanchezgarc/deepEMhancer
Coot	(Emsley and Cowtan, 2004)	https://www2.mrc-lmb.cam.ac.uk/personal/pemsley/coot/
Namdinator	(Kidmose et al., 2019)	https://namdinator.au.dk/
Phenix, version	(Afonine et al., 2018); (Terwilliger et al., 2020)	https://phenix-online.org/
MolProbity	(Williams et al., 2018)	https://molprobity.biochem.duke.edu
UCSF Chimera	(Pettersen et al., 2004)	https://www.cgl.ucsf.edu/chimera/
PyMOL Molecular Graphics System, version 2.0	Schrödinger, LLC	https://www.pymol.org/
DynamX version 3.0	Waters	N/A
ProteinLynX Global Server (PLGS) version 3.0	Waters	N/A
Deuteros version 2.0	(Lau et al., 2021)	https://github.com/andymlau/Deuteros_2.0
Other		
FACS Melody	BD Biosciences	N/A
Victor™ X3 multilabel reader	Perkin Elmer	N/A
EliSpot reader	Autoimmun Diagnostika GmbH	N/A
Vitrobot Mark IV	Thermo Fisher Scientific	N/A
K2 Summit direct electron detector	Gatan	N/A
GIF BioQuantum energy filter	Gatan	N/A
Titan Krios G3i cryo-electron microscope	Thermo Fisher Scientific	N/A
LEAP PAL system Automation technology	Trajan	N/A
nanoACQUITY UPLC	Waters	N/A
Synapt G2-Si mass spectrometer	Waters	N/A

RESOURCE AVAILABILITY

Lead contact

Further information and requests for resources and reagents should be directed to and will be fulfilled by the Lead Contact, Katie J Doores (katie.doores@kcl.ac.uk).

Materials availability

Reagents generated in this study are available from the Lead contact with a completed Materials Transfer Agreement.

Data and code availability

- The cryo-EM reconstruction and refined model of the S1-P008_60 Fab complex have been deposited with the EMDB and the PDB under accession codes EMDB: EMD-14591 and PDB: 7ZBU.
- Following the community-based recommendations (Masson et al., 2019), HDX-MS data supporting these findings (table containing deuterium uptake values and uptake plots) can be found in Data S1 and S2. HDX-MS raw data are also available from A.P. and V.C. upon request.
- This paper does not report original code.
- Any additional information required to reanalyze the data reported in this paper is available from the lead contact upon request.

EXPERIMENTAL MODEL AND SUBJECT DETAILS

Bacterial strains and cell culture

SARS-CoV-2 and SARS-CoV-1 pseudotypes were produced by transfection of HEK293T/17 cells (ATCC® CRL-11268™) and neutralization activity assayed using HeLa cells stably expressing ACE2 (kind gift from James E Voss). Expression of monoclonal antibodies and Fab fragments was performed in 293 Freestyle cells (Thermofisher Scientific, R79007). Experiments with surface

expressed Spike glycoproteins were performed using HEK293T cells (ATCC® CRL-3216™). Bacterial transformations were performed with NEB® Stable Competent *E. coli*.

METHOD DETAILS

Monoclonal antibody isolation

P008_60 (Graham et al., 2021) and VA14_47 (Seow et al., 2022) were isolated previously. VA047_02 was isolated from a donor P008 after receiving 1-dose of BNT162b2 vaccine using the methods described in Graham et al. and Seow et al. (Graham et al., 2021; Seow et al., 2022).

Protein expression and purification

Recombinant Spike and S1 (residues 1–530) for ELISA were expressed and purified as previously described (Pickering et al., 2020; Rosa et al., 2021; Seow et al., 2020). S1 protein containing the SD1 domain (residues 1–674) was obtained from Native Antigen Company (Cat number: REC31806-100).

For CryoEM and HDX: Trimeric SARS-CoV-2 Spike ectodomain (corresponding to residues 1–1208 of the Wuhan isolate Spike, UniProt ID YP_009724390) with amino acid substitutions stabilizing the pre-fusion conformation (K986P and V987P), with disrupted furin cleavage site, and carboxy-terminal foldon followed by a hexahistidine tag (Wrobel et al., 2020) was produced by expression in a stable cell line and purified as previously described (Rosa et al., 2021).

IgG1 antibody heavy and light plasmids were co-transfected at a 1:1 ratio into HEK-293F cells (Thermofisher) using PEI Max (1 mg/mL, Polysciences, Inc.) at a 3:1 ratio (PEI Max:DNA). Antibody supernatants were harvested five days following transfection, filtered and purified using protein G affinity chromatography following the manufacturer's protocol (GE Healthcare).

Fab cloning and expression

P008_60 variable heavy and light chain domains were cloned into the pHLsec vector (Aricescu et al., 2006) containing the CH1 or CK1 domains, respectively. Antibody heavy and light plasmids were co-transfected at a 1:1 ratio into HEK-293F cells (Thermofisher) using PEI Max (1 mg/mL, Polysciences, Inc.) at a 3:1 ratio (PEI Max:DNA). Antibody supernatants were harvested five days following transfection, filtered and purified using immobilized metal affinity chromatography and size-exclusion chromatography.

ELISA (spike, S1 (1-530), S1+SD1 (1-674))

96-well plates (Corning, 3690) were coated with antigen at 3 μg/mL overnight at 4°C. The plates were washed (5 times with PBS/0.05% Tween-20, PBS-T), blocked with blocking buffer (5% skimmed milk in PBS-T) for 1 h at room temperature. Serial dilutions of plasma, mAb or supernatant in blocking buffer were added and incubated for 2 h at room temperature. Plates were washed (5 times with PBS-T) and secondary antibody was added and incubated for 1 h at room temperature. IgG was detected using Goat-anti-human-Fc-AP (alkaline phosphatase) (1:1,000) (Jackson: 109-055-098). Plates were washed (5 times with PBS-T) and developed with AP substrate (Sigma) and read at 405 nm.

IgG digestion to generate F(ab')$_2$

IgG were incubated with IdeS (Dixon, 2014) (4 μg of IdeS per 1 mg of IgG) in PBS for 1 h at 37°C. The Fc and IdeS A were removed using a mix of Protein A Sepharose® Fast Flow (250 μL per 1 mg digested mAb; GE Healthcare Life Sciences) and Ni Sepharose™ 6 Fast Flow (50 μL per 1 mg digested mAb; GE Healthcare Life Sciences) which were washed twice with PBS before adding to the reaction mixture. After exactly 10 min the beads were removed from the F(ab')$_2$-dilution by filtration in Spin-X tube filters (Costar®) and the filtrate was concentrated in Amicon® Ultra Filters (10k, Millipore). Purified F(ab')$_2$ fragments were analyzed by SDS-PAGE.

F(ab')$_2$ and IgG competition ELISA

96-well half area high bind microplates (Corning®) were coated with Spike protein at 3μg/mL in PBS overnight at 4°C. Plates were washed (5 times with PBS/0.05% Tween 20, PBS-T) and blocked with 5% milk in PBS/T for 2 h at room temperature. Serial dilutions (5-fold) of F(ab')$_2$, starting at 100-molar excess of the IC_{80} of Spike binding, were added to the plates and incubated for 1 h at room temperature. Plates were washed (5 times with PBS-T) before competing IgG was added at their IC_{80} of Spike binding and incubated at room temperature for 1 h. Plates were washed (5 times with PBS-T) and Goat-anti-human-Fc-AP (alkaline phosphatase) (1:1,000) (Jackson: 109-055-098) was added and incubated for 45 min at room temperature. The plates were washed (5 times with PBS-T) and AP substrate (Sigma) was added. Optical density was measured at 405 nm in 5-min intervals. The percentage competition was calculated as the reduction in IgG binding in the presence of F(ab')$_2$ (at 100-molar excess of the IC_{80}) as a percentage of the maximum IgG binding in the absence of F(ab')$_2$. Competition groups were then arranged according to binding epitopes.

SARS-CoV-2 and SARS-CoV-1 pseudotyped virus preparation

SARS-CoV-2 Spike plasmids were obtained from Wendy Barclay (Imperial). SARS-CoV-1 Spike R577A mutant was generated using site-directed mutagenesis (Forward primer: CAGATTCTGTGGCGGACCCCAAGACCAGC, Reverse primer: GCTGGTCTTGGGGTC CGCCACAGAATCTG). Pseudotyped HIV-1 virus incorporating either the SARS-Cov-2 Wuhan, B.1.1.7, B.1.351, B.1.617.2, BA.1

truncated Spike (final 19 amino acids were removed using a K1255* mutation) (Lechmere et al., 2022) or SARS-CoV-1 full-length Spike were produced in a 10 cm dish seeded the day prior with 5 × 10^6 HEK293T/17 cells in 10 mL of complete Dulbecco's Modified Eagle's Medium (DMEM-C, 10% fetal bovine serum (FBS) and 1% Pen/Strep (100 IU/mL penicillin and 100 mg/mL streptomycin)). Cells were transfected using 90 μg of PEI-Max (1 mg/mL, Polysciences) with: 15 μg of HIV-luciferase plasmid, 10 μg of HIV 8.91 gag/pol plasmid (Zufferey et al., 1997) and 5 μg of SARS-CoV-2 Spike protein plasmid (Grehan et al., 2015; Thompson et al., 2020). Pseudotyped virus was harvested after 72 h, filtered through a 0.45mm filter and stored at −80°C until required.

Neutralization assay with SARS-CoV-2 and SARS-CoV-1 pseudotyped virus

Neutralization assays were conducted as previously described (Carter et al., 2020; Monin et al., 2021; Seow et al., 2020). Serial dilutions of serum samples (heat inactivated at 56°C for 30mins) or mAbs were prepared with DMEM-C media and incubated with pseudotyped virus for 1 h at 37°C in 96-well plates. Next, HeLa cells stably expressing the ACE2 receptor (provided by Dr James Voss, Scripps Research, La Jolla, CA) were added (12,500 cells/50μL per well) and the plates were left for 72 h. The amount of infection was assessed in lysed cells with the Bright-Glo luciferase kit (Promega), using a Victor™ X3 multilabel reader (PerkinElmer). Measurements were performed in duplicate and duplicates used to calculate the ID_{50}.

SARS-CoV-2 microneutralization assay

Vero-E6-TMPRSS2 cells were seeded in 96-well plates at a density of 30,000 cells per well the day prior to infection, in DMEM supplemented with 10% FCS and 1% pen/strep. Serial dilutions of mAbs were performed in 96-well plates in DMEM supplemented with 2% FCS. 50 μL of virus was added to 50 μl of diluted antibody and incubated at 37°C for 1 h. Media was removed from the cells and replaced with 100 μL of antibody-virus mixture and incubated at 37°C for 1 h. Pre-warmed carboxymethylcellulose (Sigma-Aldrich, C4888) overlay was added, without removing the inoculum, to a final concentration of 0.5%. Plates were incubated for a further 14–21 h at 37°C before removing media and fixing with 4% formaldehyde in PBS for 30 min at room temperature. Virus inputs were optimized to ensure 100–200 discrete plaques per well in virus-only control wells. Incubation times of between 15 and 22 h post addition of virus were determined for each variant to ensure optimal plaque size.

Foci were stained with an anti-SARS-CoV-2 nucleocapsid antibody using Triton X-100 permeabilization as detailed previously (Seow et al., 2020). Briefly, the fixed monolayer was permeabilised with 0.2% Triton X-100 in PBS, blocked in 3% milk and incubated with murinized CR3009 (2 μg/mL) for 45 min at room temperature. Cells were washed twice in PBS then incubated with goat anti-mouse IgG (Fc-specific)-peroxidase antibody (Sigma A2554; 2 mg/mL) for 30 min at room temperature. After two further washes, 50 μL per well of TrueBlue peroxidase substrate (SeraCare, 50-78-02) was added and incubated for 20–40 min at room temperature. Plates were then air-dried and foci counted on an EliSpot reader (Autoimmun Diagnostika GmbH) using AID EliSpot 8.0 software.

Monoclonal antibody binding to spike using flow cytometry

HEK293T cells were plated in a 6-well plate (2 × 10^6 cells/well). Cells were transfected with 1 μg of plasmid encoding full-length SARS-CoV-1, SARS-CoV-2, HKU1, OC43, NL63, 229E and MERS full-length Spike and incubated for 48h at 37°C. Post incubation cells were resuspended in PBS and plated in 96-well plates (1 × 10^5 cells/well). Monoclonal antibodies were diluted in FACS buffer (1x PBS, 2% FBS, 1 mM EDTA) to 25 μg/mL and incubated with cells on ice for 1 h. The plates were washed twice in FACS buffer and stained with 50 μL/well of 1:200 dilution of PE-conjugated mouse anti-human IgG Fc antibody (BioLegend, 409,304) on ice in dark for 1 h. After another two washes, stained cells were analyzed using flow cytometry, and the binding data were generated by calculating the percent (%) PE-positive cells using FlowJo 10 software.

ACE2 competition measured by flow cytometry

To prepare the fluorescent probe, Streptavidin-APC (Thermofisher Scientific, S32362) was added to biotinylated SARS-CoV-2 Spike (3.5 times molar excess of Spike) on ice. Additions were staggered over 5 steps with 30 min incubation times between each addition. Purified mAbs were mixed with PEAPCconjugated SARS-CoV-2 spike in a molar ratio of 4:1 in FACS buffer (2% FBS in PBS) on ice for 1 h. HeLa-ACE2 and HeLa cells were washed once with PBS and detached using PBS containing 5mM EDTA. Detached cells were washed and resuspended in FACS buffer. 0.5 million HeLa-ACE2 cells were added to each mAb-spike complex and incubated on ice for 30 m. The cells were washed with PBS and resuspended in 1 mL FACS buffer with 1 μL of LIVE/DEAD Fixable Aqua Dead Cell Stain Kit (Invitrogen). HeLa-ACE2 cells alone and with SARS-CoV-2 Spike only were used as background and positive controls, respectively. The geometric mean fluorescence for PE was measured from the gate of singlet and live cells. The ACE2 binding inhibition percentage was calculated as described previously (Graham et al., 2021; Rogers et al., 2020).

$$\%\ ACE2\ \ binding\ inhibition\ =\ 100 * \left(1 - \frac{sample\ geometric\ mean\ -\ geomatric\ mean\ of\ background}{geometric\ mean\ of\ positive\ control\ -\ geometric\ mean\ of\ background}\right)$$

S1 shedding assay

HEK293T cells were plated in a 6-well plate (2 × 10^6 cells/well). Cells were transfected with 1 μg of plasmid encoding full-length SARS-CoV-2 Spikes using 3 μL of TransIT-2020 (Mirus Bio) and incubated for 48h at 37°C. Post incubation cells were resuspended in PBS and plated in U-bottom 96-well plates (1 × 10^5cells/well). Monoclonal antibodies were diluted in FACS buffer (1x PBS, 2%

FBS, 1 mM EDTA) to 25 μg/mL and incubated with cells on ice for 60, 30, 20, 10 or 5 min. The plates were washed twice in FACS buffer and stained with 50 μL/well of 1:200 dilution of PE-conjugated mouse anti-human IgG Fc antibody (BioLegend, 409,304) on ice in dark for 1 h. After another two washes, stained cells were analyzed using flow cytometry, and the binding data were generated by calculating the percent (%) PE-positive cells using FlowJo 10 software. Mean fluorescence intensity (MFI) for each sample was determined at each time point and each sample was normalized to the MFI at the 5 min time point (MFI/MFI 5 min × 100).

Cryo-EM data collection, image processing and structure refinement

Four μl SARS-CoV-2 Spike ectodomain (0.5 mg/mL), supplemented 0.2 mg/mL P008_60 Fab and 0.1% n-octyl glucoside in 150 mM NaCl, 20 mM Tris-HCl, pH8.0, was applied onto glow-discharged 200-mesh copper holey carbon R2/2 grids (Quantifoil) for 1 min, under 100% humidity at 20°C. The grids, blotted for 3–4 s, were plunge-frozen in liquid ethane using a Vitrobot Mark IV (Thermo Fisher Scientific). Cryo-EM data were collected on a Titan Krios G3i cryo-electron microscope (Thermo Fisher Scientific) equipped with a K3 Summit direct electron detector (Gatan) and a Gatan GIF BioQuantum energy filter. A total of 16,624 micrograph movie stacks were acquired in dose-fractionation mode, at a calibrated magnification of 82,000, corresponding to 1.1 Å per physical detector pixel (0.55 Å per super-resolution pixel). A total electron exposure of 50 e/Å^2 was fractionated across 40 movie frames; a 20-eV energy slit and a defocus range of −0.7 to −3.6 μm were used for the data collection (Table S1).

The micrograph stacks were aligned, binned to the physical pixel size of 1.1 Å and summed, with dose weighting applied, as implemented in MotionCor2 (Zheng et al., 2017) (Figure S2A). Contrast transfer function (CTF) parameters were estimated using Gctf-v1.06 (Zhang, 2016), and 644 images showing evidence of crystalline ice contamination were discarded. Initially, particles were picked with Gautomatch (http://www.mrc-lmb.cam.ac.uk/kzhangusing 2D class averages of the trimeric Spike (Rosa et al., 2021), low-pass filtered to 20 Å resolution, as templates. The resulting 1,740,430 particles, extracted in Relion-3.1 and binned to a pixel size of 4.4 Å, were subjected to two rounds of reference-free 2D classification in cryoSPARC-2 (Punjani et al., 2017). 283,956 particles belonging to well-defined 2D classes (Figure S2A) were subjected to classification into twelve 3D classes in Relion-3.1 (Scheres, 2020). Neither the 2D nor 3D class averages of the trimeric Spike revealed features attributable to a bound Fab molecule (Figures S2B and S2C). Next, 3,772,722 particles were picked using 2D class averages of the dissociated Spikes (Rosa et al., 2021). Following two rounds of 2D classification in cryoSPARC-2 (Figure S3A), 753,837 particles, re-extracted with pixel size of 2.2 Å, were subjected to 3D classification in Relion-3.1 into 9 classes using an initial model obtained by *ab*-initio reconstruction in cryoSPARC-2. The procedure revealed a single well-defined 3D class containing 208,343 particles (27.4%) of S1 protein with a bound Fab molecule (Figure S3B). The particles, re-extracted without binning (with a pixel size 1.1 Å), were subjected to two rounds of 3D classification using *ab*-initio reconstruction in cryoSPARC-2 with 2 classes and class similarity set to 0. At the end of each round, the most populated class was selected, resulting in the final set of 166,619 particles. Following Baesian polishing (Zivanov et al., 2019) in Relion-3.1, the particles were used for non-uniform refinement in cryoSPARC-2 to generate the final 3D reconstruction (Figure 2). The resolution reported is according to the gold-standard Fourier shell correlation (FSC) 0.143 criterion (Rosenthal and Henderson, 2003; Scheres and Chen, 2012) (Figure S3C and Table S1). The particles displayed a strong preferential orientation (Figure S3D); concordantly, analysis of the 3D reconstruction with 3DFSC program (Tan et al., 2017) revealed considerable anisotropy (Figure S4E). Local resolution was estimated in cryoSPARC (Figure S4F). For real-space refinement, the map was post-processed using density modification procedure in Phenix (Terwilliger et al., 2020). To aid in model building and for illustration purposes, the map was filtered and sharpened using deepEMhancer (Sanchez-Garcia et al., 2021).

The atomistic models of monomeric SARS-CoV-2 S1 protein and a Fab molecule, derived from PDB entries 7A92 (Benton et al., 2020) and 5WI9 (Min et al., 2018), were docked in the cryo-EM map using Chimera (Pettersen et al., 2004). The NTD and the RBD of the Spike subunit were replaced with the crystal structures from PDB entries 7B62 (Rosa et al., 2021) and 7OAO (Huo et al., 2021), respectively. Guided by the cryo-EM map, the model was fitted and extended interactively in Coot (Emsley and Cowtan, 2004), this model was subjected to automated flexible fitting using Namdinator (Kidmose et al., 2019), regions that were improved in terms of geometry and fit by Namdinator were used to update the model. The model was refined using phenix.real_space_refine (version dev-4213) (Afonine et al., 2018). The quality of the final model was assessed using MolProbity (Williams et al., 2018) (Table S1). The final cryo-EM reconstruction and refined model have been deposited with the EMDB and the PDB under accession codes EMDB-14591 and 7ZBU.

Hydrogen-deuterium exchange, LC-MS and data analysis

Prior to conducting HDX-MS experiments, peptides were identified by digesting undeuterated Spike using the same protocol and identical LC gradient as detailed below and performing MS^E analysis with a Synapt G2-Si mass spectrometer (Waters), applying collision energy ramping from 20–30 kV. Sodium iodide was used for calibration and Leucine Enkephalin was applied for mass accuracy correction. MS^E runs were analyzed with ProteinLynx Global Server (PLGS) 3.0 (Waters) and peptides identified in 3 out of 4 runs, with at least 0.2 fragments per amino acid (at least 2 fragments in total) and mass error below 10 ppm were selected in DynamX 3.0 (Waters). Peptides were further visually inspected to exclude those of insufficient quality. To note, post-quenching deglycosylation was not performed and no attempt to identify glycosylated peptides was made, causing partial sequence coverage in proximity to glycan sites. Coverage in 5 out 23 glycosylation sites arose from non-glycosylated peptides of Spike molecules of incomplete glycan occupancy. Deuterium labeling was performed at two different temperatures, at 20 and 0°C. HDX samples at 20°C were labeled with LEAP PAL system Automation technology (Trajan), directly coupled to a nanoACQUITY UPLC. The Spike was pre-incubated with PBS or

with mAb P008_60 at ratio 1:3 Spike trimer: mAb (ratio 1:1 Spike monomer: mAb). The exchange reactions were initiated by 6-fold dilution into deuterated PBS buffer (pH_{read} 7.6), yielding to 83.3% of final deuterium content in the reaction mixture. The reaction was allowed to proceed for 15 s, 1 min, 10 min and 100 min at 20°C. Samples were quenched by 1:1 dilution into cold (0°C) 100 mM phosphate buffer containing 4 M Urea and 0.5 M TCEP (final pH_{read} 2.3), held for 30 s at 0°C and directly injected into the LC system. HDX samples at 0°C were manually labeled on ice, at identical molar ratio as for the automated labeling conducted at 20°C. The exchange reactions were initiated by 6-fold dilution in ice-cold deuterated PBS buffer and allowed to proceed for 20 s on ice (corresponding to approximately 2 s at 20°C). Samples were quenched 1:1 with ice-cold quench buffer, held for 30 s on ice, snap-frozen in liquid nitrogen and kept at −80°C until LC-MS analysis. Frozen samples labeled on ice were quickly thawed and injected into the LC system. Triplicates were performed for time points of 15 s (20°C) and 20 s on ice. A maximally labeled sample was performed by labeling Spike in 6 M deuterated Urea in D_2O and 20 mM TCEP, resulting in a final deuterium content as for the other labeled samples. The maximally labeled control was quenched after 6 h by 1:1 dilution with ice-cold 100 mM phosphate buffer (final pH_{read} 2.3), held for 30 s on ice, snap-frozen in liquid nitrogen and kept at −80°C until LC-MS analysis. Protein samples, each containing 22.5 pmol of Spike (monomer), were quickly on-line digested at 20°C into a dual protease column (Pepsin-Type XIII protease) and trapped/desalted for 3 min at 200 μL/min and at 0°C through an Acquity BEH C18 1.7 μm VanGuard pre-column with Solvent A (0.23% formic acid in MilliQ water, pH 2.5). Peptides were eluted into an Acquity UPLC BEH C18 1.7 μm analytical column with a linear gradient raising from 8 to 40% of Solvent B (0.23% formic acid in acetonitrile) at a flow rate of 40 μL/min and at 0°C. Then, peptides went through electrospray ionization in positive mode and underwent MS analysis with ion mobility separation. In order to eliminate peptide carryover, the protease column was washed between injections using a wash buffer at 1.5 M Gu-HCl in 100 mM phosphate buffer (pH 2.5) and a wash run with a saw-tooth gradient was carried out between each run of deuterated samples. Data were analyzed with DynamX 3.0 and statistical analysis was performed with Deuteros 2.0 (Lau et al., 2021), applying 99.9% of confidence interval.

HDX-MS data supporting these findings (table containing deuterium uptake values and uptake plots) are available and incorporated as supplemental information (Data S1 and S2).

QUANTIFICATION AND STATISTICAL ANALYSIS

All neutralization and ELISA experiments were performed in duplicate. The 50% inhibitory concentrations/dilutions (IC/ID_{50}) were calculated using GraphPad Prism software. For HDX-MS analysis, a difference in HDX between states “spike alone” and “mAb-bound spike” was considered significant if exceeded a threshold of 0.44 Da. The threshold was calculated with 99.9% of confidence interval by Deuteros 2.0 (Lau et al., 2021), which uses the pooled average standard deviation of peptide deuterium uptake at time points performed in triplicates. Triplicates labeling experiments and LC-MS runs (n = 3) were performed at time points: 20 s on ice and 15 s at 20°C. Average standard deviation in deuterium uptake was calculated by Deuteros software as 0.0169 for state “spike alone” and 0.0151 for state “mAb-bound spike”. This is reported in the HDX summary table (Table S2). The threshold of significance is indicated in Figures S6 and S7.

Cell Reports, Volume *40*

Supplemental information

A neutralizing epitope on the SD1 domain of SARS-CoV-2 spike targeted following infection and vaccination

Jeffrey Seow, Hataf Khan, Annachiara Rosa, Valeria Calvaresi, Carl Graham, Suzanne Pickering, Valerie E. Pye, Nora B. Cronin, Isabella Huettner, Michael H. Malim, Argyris Politis, Peter Cherepanov, and Katie J. Doores

A neutralizing epitope on the SD1 domain of SARS-CoV-2 Spike targeted following infection and vaccination.

Jeffrey Seow[1]*, Hataf Khan[1]*, Annachiara Rosa[2]*, Valeria Calvaresi[3]*, Carl Graham[1], Suzanne Pickering[1], Valerie E. Pye[2], Nora B. Cronin[4], Isabella Huettner[1], Michael H. Malim[1], Argyris Politis[3]#, Peter Cherepanov[2,5]#, Katie J. Doores[1,6]#

[1] Department of Infectious Diseases, School of Immunology & Microbial Sciences, King's College London, London, UK.
[2]Chromatin Structure and Mobile DNA Laboratory, The Francis Crick Institute, London, UK
[3] Department of Chemistry, King's College London, London, UK.
[4]LonCEM Facility, The Francis Crick Institute, London, UK;
[5]Department of Infectious Disease, St-Mary's Campus, Imperial College London, London, UK.
[6]Lead author

***** These authors contributed equally

# To whom correspondence should be addressed (katie.doores@kcl.ac.uk, Peter.Cherepanov@crick.ac.uk, argyris.politis@kcl.ac.uk)

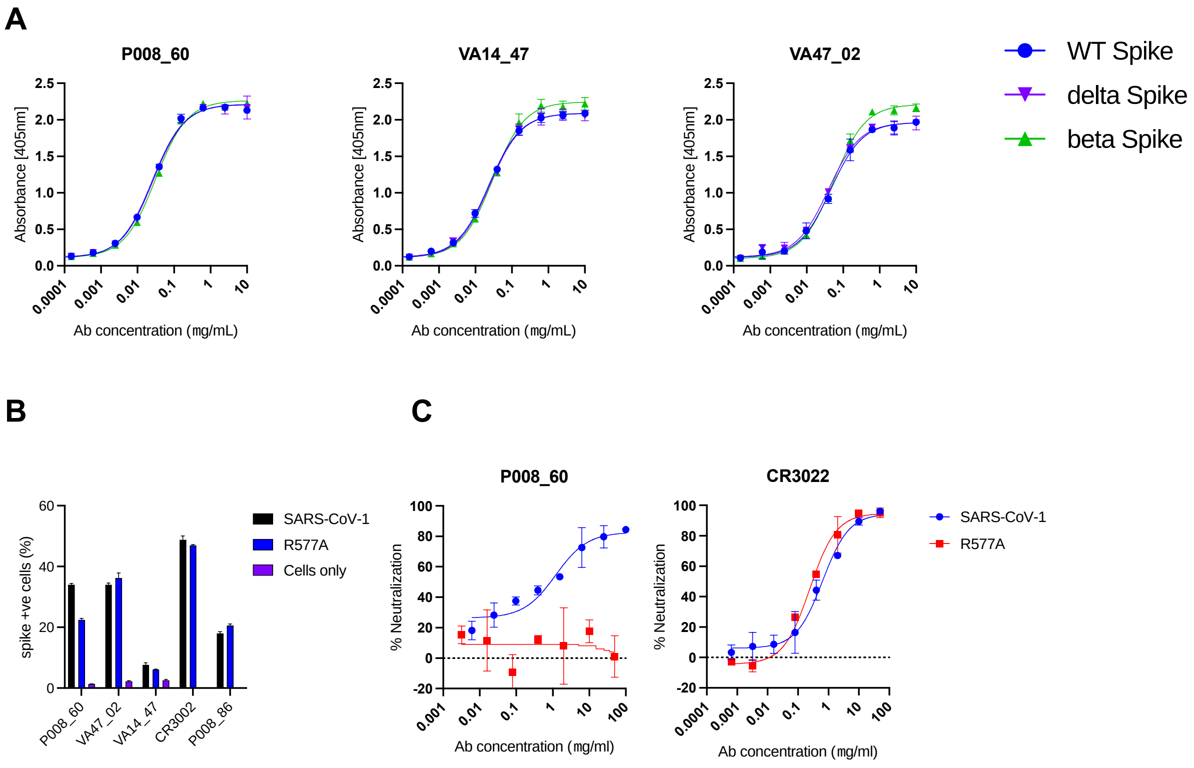

Figure S1. SD1 mAb binding to SARS-CoV-2 variants of concern and SARS-CoV-1. A) Binding of P008_60, VA14_47 and VA47_2 to WT, delta and beta recombinant Spike by ELISA. **B)** SD1 mAbs bind to SARS-CoV-1 Spike and R577A mutant expressed on the surface of HEK 293T cells. CR3022 and P008_86 were used as controls. **C)** Neutralization of SARS-CoV-1 WT and R577A pseudotyped particles by P008_60. CR3022 is used as a control. Experiments were performed in duplicate and performed at least twice. Representative data sets are shown. Error bars represent the range of the values for experiments performed in duplicate (not shown when smaller than symbol size). Related to **Figure 1**.

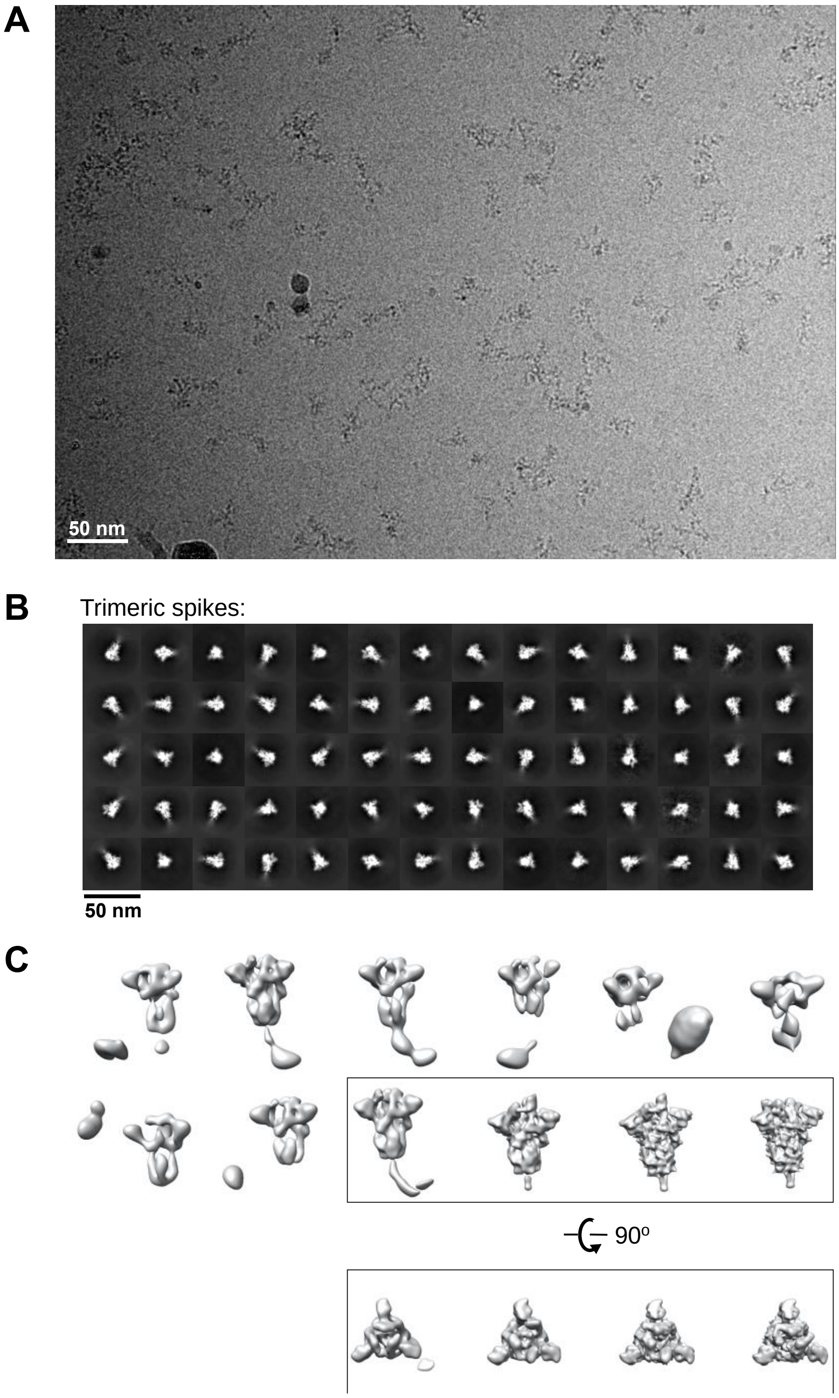

Figure S2. Preliminary cryo-EM data analysis. (A) Example of a micrograph of a vitrified sample containing SARS-CoV-2 spike supplemented with excess Fab P008_60; the scale bar is 50 nm. **(B)** 2D class averages corresponding to trimeric spike ectodomain **(C)** Result of 3D classification of trimeric spikes into 12 classes. The best 4 classes (boxed) are shown in two orthogonal views. Note that features corresponding to bound Fab molecules are absent in 2D and 3D class averages. Related to **Figure 2**.

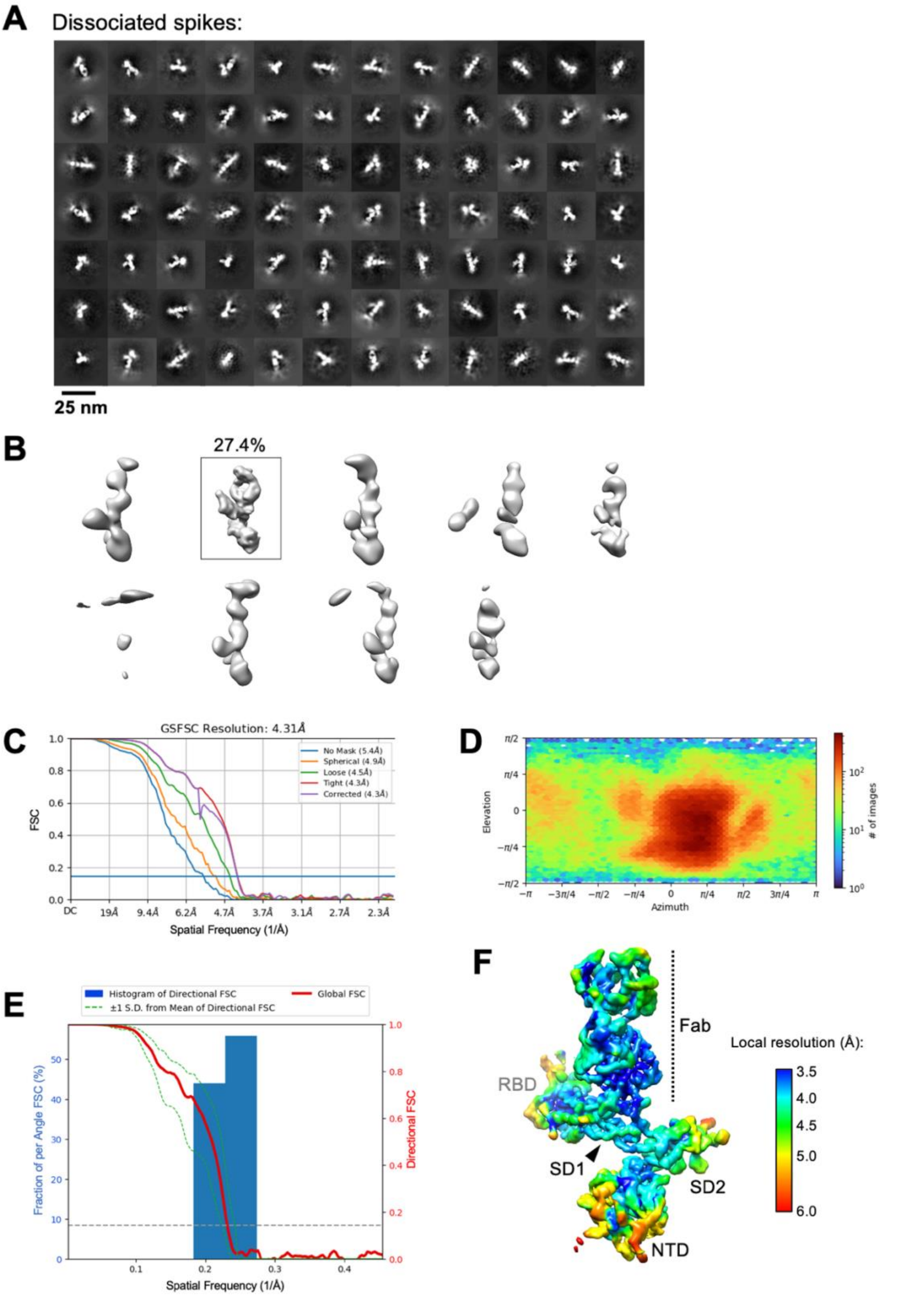

Figure S3. Cryo-EM reconstruction of the S1-Fab P008_60 complex. (A) 2D class averages corresponding to dissociated spikes. **(B)** Results of 3D classification into 9 classes. Particles contributing to the single well-defined 3D class (boxed, 27.4% particles) were retained for downstream processing. **(C, D)** Half-map Fourier shell correlations (FSCs, C) and distribution of the refined particle orientations for the final reconstruction, as implemented in cryoSPARC (D). **(E)** Directional resolution metrics generated by 3DFSC software (12) showing the global FSC curve (thick red line), boundaries of the directional FSCs (± 1 standard deviation, dotted green

lines), and a histogram of directional FSC values (blue bars). **(E)** The final 3D reconstruction, colored by local resolution; the color scheme corresponds to the key on the right. Related to **Figure 2**.

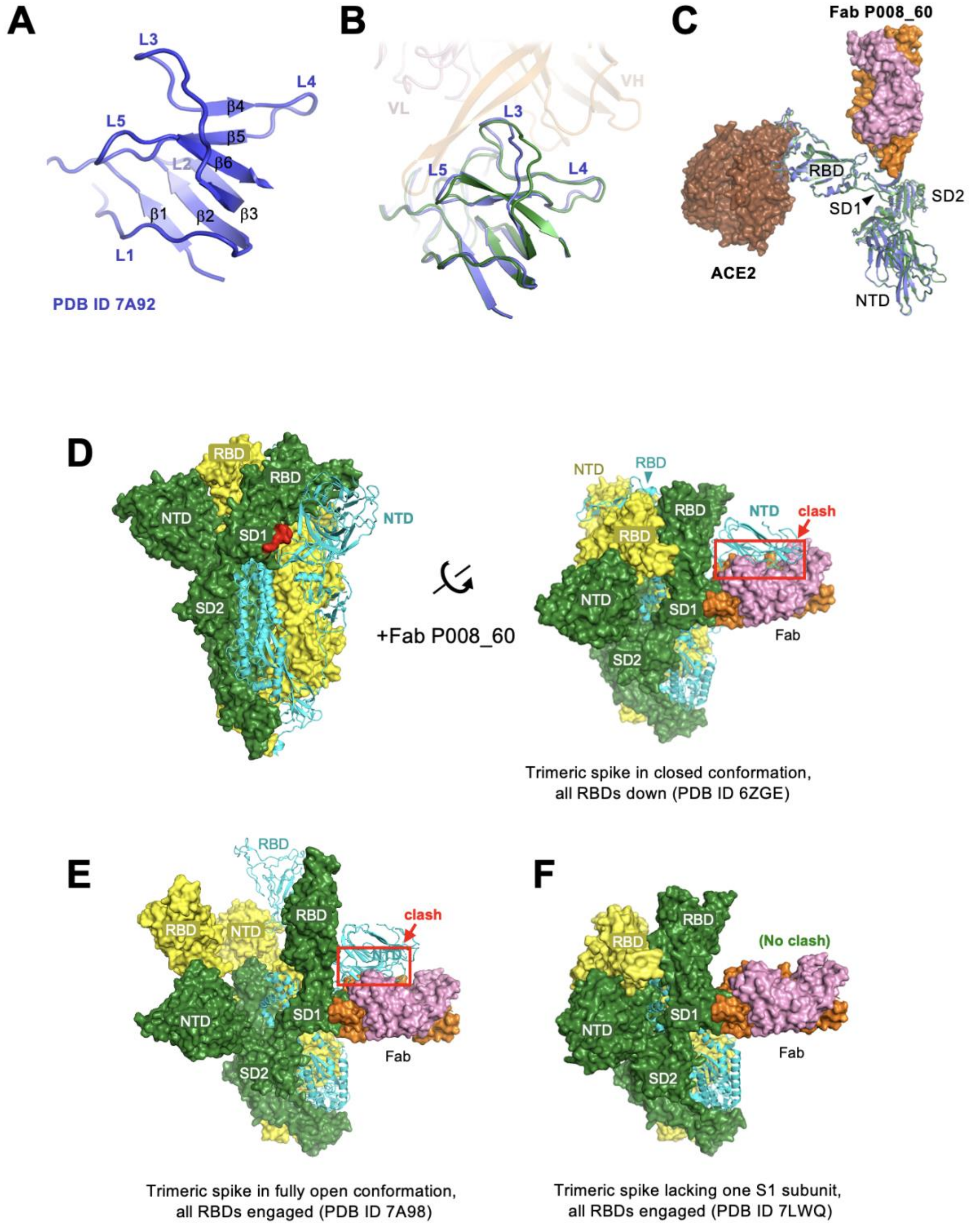

Figure S4. (A) Structure of SD1 shown in cartoons, with individual β-strands and loops indicated; the structure shown is from PDB entry 7A92 (3). **(B)** Superposition of SD1 structures without (blue, PDB entry 7A92 (3)) and with (green) Fab P008_60 bound; the Fab is shown as semi-transparent cartoons. **(C)** Superposition of the ACE2- and P008_60 Fab- bound S1 structures. Fab (pink and orange) and ACE2 (yellow) are shown as surface representations, and S1 as cartoons. **(D)** Left: models of the trimeric spike in fully closed (left, PDB entry 6ZGE (4)) in the orientation and colors as in **Fig. 2C**, with two spike subunits shown as green and yellow surface representations (including S2 portions) and one as cyan cartoons. Right: model of the closed trimeric spike trimer in complex with P008_60, bound to the SD1 of the green spike subunit. Note the extensive clashes (indicated with red box and arrowhead) with the NTD of the neighboring (cyan) S1 subunit. **(E,F)** Models of

fully open trimeric spike **(E)** (PDB entry 7A98 (3)) and partially dissociated trimeric spike, lacking one of the S1 subunits **(F)** (PDB entry 7LWQ (21)) in complex with P008_60. For clarity, ACE2 molecules (present in PDB entry 7A98) are hidden; Fab and two spike subunits are shown as surface representation and one spike chain is shown as cartoons. Related to **Figure 2**.

A

	VH	%	IGHJ	%	IGHD	CDRH3	VK	%	IGKJ	%	CDRL3
**P008_60**	IGHV3-30	95.5	IGHJ4	97.9	IGHD1-14	CARDTPDLETYYFDCW	IGKV3-20	97.9	IGKJ2	87.2	CQQYGDSPRGSF
**VA14_47**	IGHV3-30	97.9	IGHJ6	90.3	IGHD3-3	CTKADYYDFWSGYQKTYYYYMDVW	IGKV3-20	97.9	IGKJ5	100.0	CQQYGSSPQITF
**VA47_2**	IGHV4-61	94.2	IGHJ3	94.0	IGHD3-22	CARVDPYYYSSSGYWTNAFDIW	IGKV3-20	95.0	IGKJ1	94.7	CHQYDNLWTF

B

Heavy chain alignment

```
47_2        QVQLQESGPGLVKPSQTLSLTCTVSGGSISNTNYFWNWIRQPAGKGLEWIGHTYTSGS-T 59
14_47       EVQLVESGGGVVQPGRSLRLSCGGSGFTFS--SHAMHWVRQAPGKGLEWVAVISYDGSYQ 58
8_60        EVQLVESGGGVVQPGRSLRLTCAASGFIFS--SYGMHWVRQAPGKGLEWVAVISYDGSYK 58
            :*** *** *:*:*.::* *:*  **  :*  .:  :*:**  ******:.    .**

47_2        NYNPSLKSRVTISIDTSRNQFSLKLSSVTATDTAVYYCARVDPYYYSS--SGYWTNAFDI 117
14_47       YYADSVKGRFTISRDNSKNALYLQMNSLRVEDTAIYYCARDT--------PDLETYYFDC 110
8_60        YYADSVKGRFTISRDNSKNTLYLQMNSLRAEDTAVYYCTKADYYDFWSGYQKTYYYYMDV 118
             *  *:*.*.*** *.*:* : *::.*: . ***:***::                 :*

47_2        WGQGTMVTVSS  128
14_47       WGQGTLVTVSS  121
8_60        WGKGTTVTISS  129
            **:** **:**
```

Light chain alignment

```
14_47       EIVMTQSPGTLSLSPGERATLSCRASQSVSSSYLAWYQQKPGQAPRLLIYSACSRATGIP 60
8_60        DIQLTQSPGTLSLSPGERATLSCRASQSVSSSYLAWYQQKPGQAPRLLIYGTSSRATGIP 60
47_02       EIVMTQSPGTLXSSPGERVTLSCRASQSVSSNYLAWYQQKPGQAPRLLIYGASKRATGIP 60
            :* :*******  *****.************.******************.:..******

14_47       DRFSGSGSGTDFTLTISRLEPEDFAVYYCQQYGDSPRGSFGQGTKVDIK    109
8_60        DRFSGSGSGTDFTLTISRLEPEDFAVYYCQQYGSSPQITFGQGTRLEIK    109
47_02       DRFSGGGSGTDFTLTISRLEPEDFAIYYCHQYDN--LWTFGQGTKLEIK    107
            *****.*******************:***:**..    :*****:::**
```

Figure S5: SD1 mAb germline characteristics. A) Germline gene usage and level of somatic hypermutation**. B)** SD1-specific mAb heavy and light chain alignment. Related to **Figure 1** and **Figure 2**.

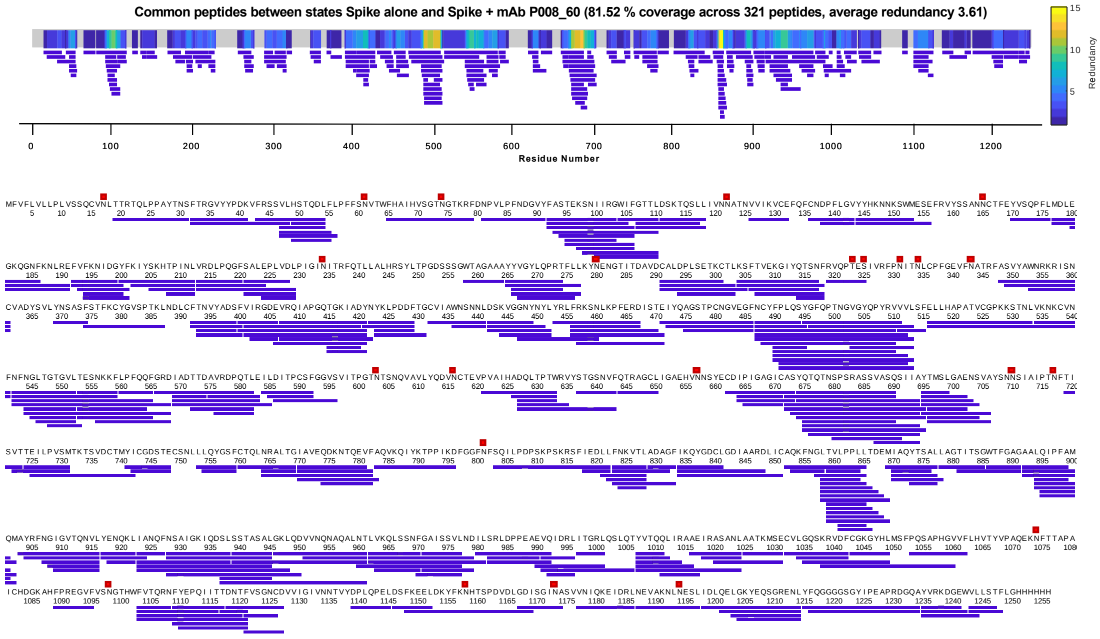

Fig. S6. Sequence coverage of peptides followed by HDX-MS. The blue segments indicate the peptides followed and the red squares indicate the glycosylation sites. Related to **Figure 3**.

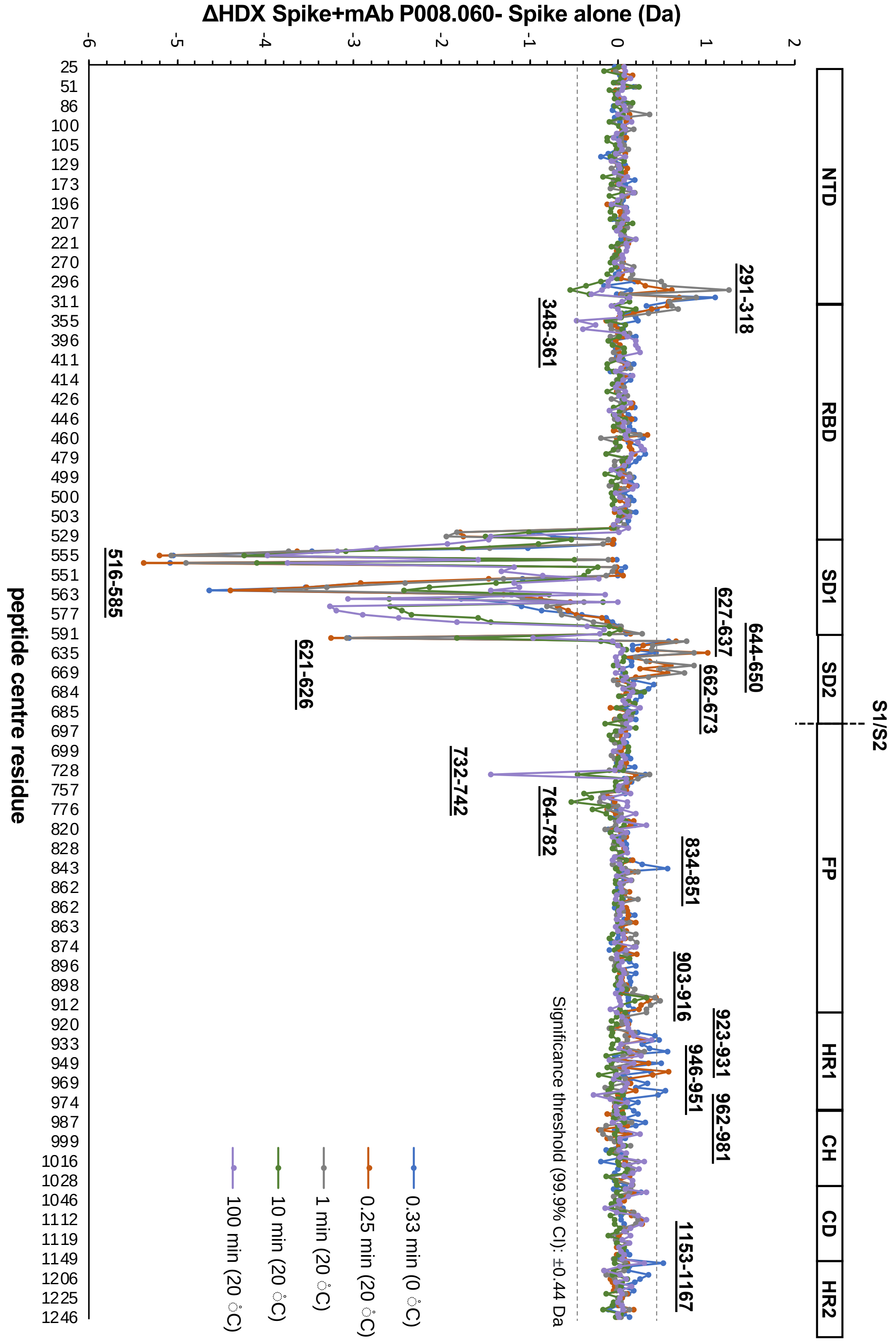

Fig. S7. Difference plot illustrates the differences in HDX over the measured time points between Spike + mAb P008_60 and Spike alone. Residues comprising a region with a statistically significant difference in HDX are indicated. The peptides are arranged according to their position from N- to C-terminus. The various subdomains of the Spike protein are indicated. NTD: N-terminal domain, RBD: receptor binding domain, SD1: subdomain 1, SD2: subdomain 2, FP: fusion peptide, HR1: heptad repeat 1, CH: central helix, CD: connector domain, HR2: heptad repeat 2. A dotted line denotes the furin cleavage site (separating S1 and S2 domains). Related to **Figure 3**.

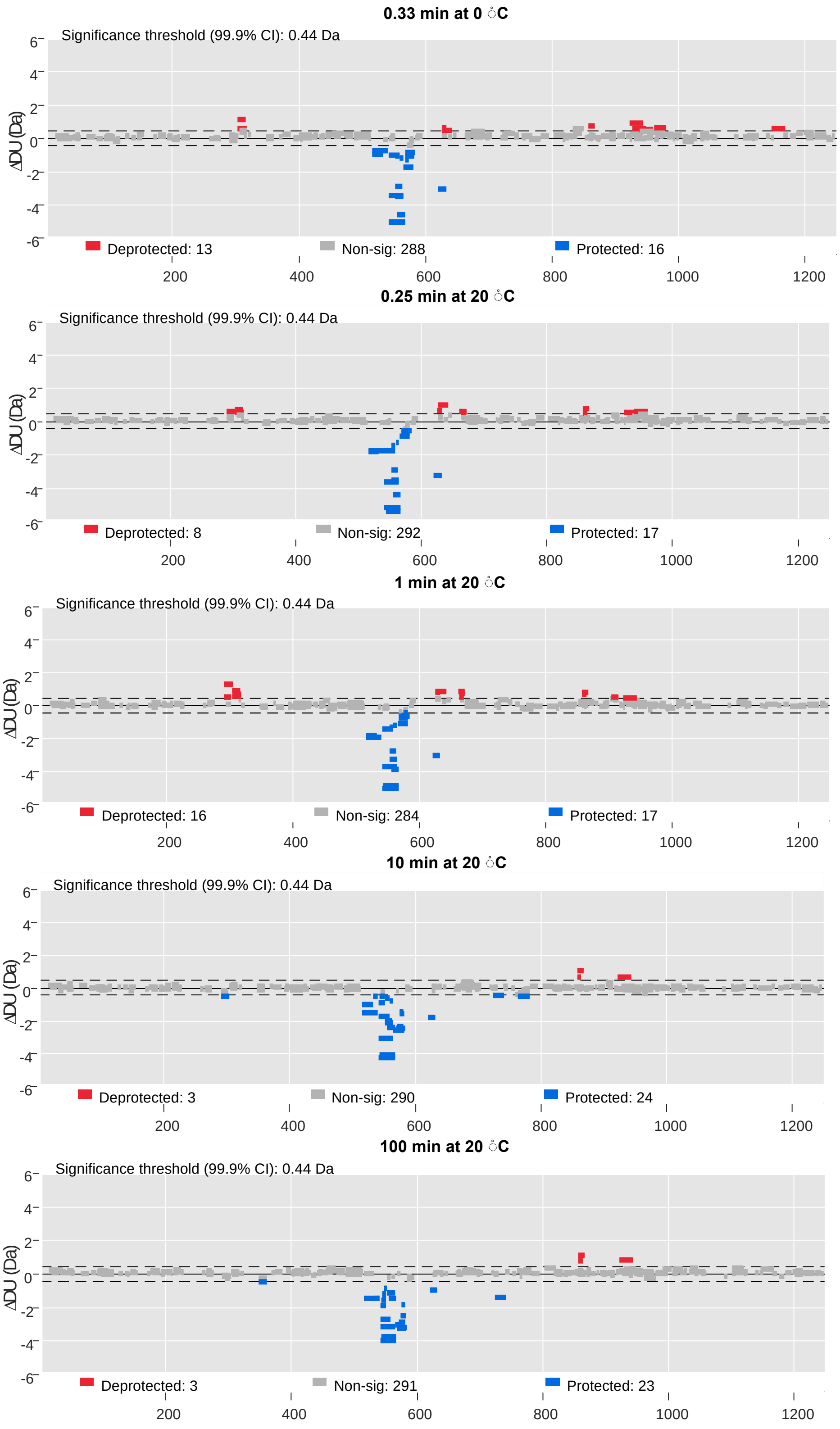

Fig S8. Statistically significant and not significant peptides grouped by time point and arranged according to their position from the N- to the C-terminus. Figures generated by Deuteros software (version 2.0). Related to **Figure 3**.

Table S1. Cryo-EM data collection, 3D volume reconstruction, and model refinement. Related to **Figure 2**.

**Database accession codes**	
EMDB	EMDB-14591
RCSB	7ZBU
**Data collection**	
Microscope	Titan Krios G3i
Operating voltage (kV)	300
Detector	Gatan K3
Physical pixel size (Å)	1.1
Defocus range (μm)	-0.7-3.6
Number of frames per movie	40
Total electron dose (e/Å^2)	50
Total movies acquired/used	16,624/15,980
Movie alignment software	MotionCor2
**3D Reconstruction**	
Software for 2D classification	cryoSPARC-2
Software for 3D classification	Relion-3.1
Software for reconstruction	Relion-3.1
Number of extracted particles	3,772,722
Number of refined particles	166,619
Symmetry imposed	C1
Map resolution (Å) [a]	4.31
Map 3D FSC sphericity	0.885
**Model refinement**	
Software for real-space refinement	Phenix version dev-4213
Number of atoms	8,529
Real-space correlation coefficient	0.68
Mean B-factor (Å^2)	133
R.m.s. deviations	
Bonds (Å)	0.003
Angles (°)	0.679
Validation	
MolProbity score	1.73
Clash score	10.09
Rotamers outliers (%)	0.21
Ramachandran plot quality (%)	
Favored	96.69
Disallowed	0

[a] Based on the FSC of 0.143 between half-sets.

Table S2: HDX summary table. Related to **Figure 3**.

States	Spike protein alone; Spike protein + mAb P008_60
HDX reaction details	PBS buffer (137 mM NaCl, 2.7 mM KCl, 8 mM Na_2HPO_4, and 2 mM KH_2PO_4); pH/D_{read}=7.6; 83.3% D_2O
HDX time course (min)	0.33 at 0 ºC (on ice); 0.25, 1, 10 and 100 at 20 ºC
HDX control samples	Maximally labelled sample
Back-exchange (mean / IQR)	29.12% / 10.55%
# of Peptides	321
Sequence coverage	81.52%
Average peptide length / redundancy	11.55 / 3.61
Replicates	3 (technical)
Repeatability (average SD)	0.0169 (Spike protein alone); 0.0151 (Spike protein + mAb P008_60)
